# Whole-genome phylogenies of the family *Bacillaceae *and expansion of the sigma factor gene family in the *Bacillus cereus *species-group

**DOI:** 10.1186/1471-2164-12-430

**Published:** 2011-08-24

**Authors:** Timothy R Schmidt, Edgar J Scott, David W Dyer

**Affiliations:** 1 Department of Microbiology and Immunology, Oklahoma University Health Sciences Center, Oklahoma City, OK 73117 USA

## Abstract

**Background:**

The *Bacillus cereus sensu lato *group consists of six species (*B. anthracis*, *B. cereus*, *B. mycoides*, *B. pseudomycoides*, *B. thuringiensis*, and *B. weihenstephanensis*). While classical microbial taxonomy proposed these organisms as distinct species, newer molecular phylogenies and comparative genome sequencing suggests that these organisms should be classified as a single species (thus, we will refer to these organisms collectively as the *Bc *species-group). How do we account for the underlying similarity of these phenotypically diverse microbes? It has been established for some time that the most rapidly evolving and evolutionarily flexible portions of the bacterial genome are regulatory sequences and transcriptional networks. Other studies have suggested that the sigma factor gene family of these organisms has diverged and expanded significantly relative to their ancestors; sigma factors are those portions of the bacterial transcriptional apparatus that control RNA polymerase recognition for promoter selection. Thus, examining sigma factor divergence in these organisms would concurrently examine both regulatory sequences and transcriptional networks important for divergence. We began this examination by comparison to the sigma factor gene set of *B. subtilis*.

**Results:**

Phylogenetic analysis of the *Bc *species-group utilizing 157 single-copy genes of the family *Bacillaceae *suggests that several taxonomic revisions of the genus *Bacillus *should be considered. Within the *Bc *species-group there is little indication that the currently recognized species form related sub-groupings, suggesting that they are members of the same species. The sigma factor gene family encoded by the *Bc *species-group appears to be the result of a dynamic gene-duplication and gene-loss process that in previous analyses underestimated the true heterogeneity of the sigma factor content in the *Bc *species-group.

**Conclusions:**

Expansion of the sigma factor gene family appears to have preferentially occurred within the extracytoplasmic function (ECF) sigma factor genes, while the primary alternative (PA) sigma factor genes are, in general, highly conserved with those found in *B. subtilis*. Divergence of the sigma-controlled transcriptional regulons among various members of the *Bc *species-group likely has a major role in explaining the diversity of phenotypic characteristics seen in members of the *Bc *species-group.

## Background

The genus *Bacillus *consists of a heterogeneous group of Gram-positive heterotrophic aerobic or facultative anaerobic bacilli with the ability to form environmentally resistant, metabolically inert spores [1]. These soil-borne organisms are ubiquitous throughout the world, and occupy surprisingly diverse environments [2,3]. Within this large genus, the *B. cereus sensu lato *group consists of six species [*B. anthracis *(*Ba*), *B. cereus *(*Bc*), *B. mycoides*, *B. pseudomycoides*, *B. thuringiensis *(*Bt*), and *B. weihenstephanensis*], based on classical microbial taxonomy [4]. However, newer molecular phylogenies and comparative genome sequencing suggests that these organisms should be classified as a single species [5]. On the surface, this conclusion seems difficult to reconcile with the varied biological characteristics of these organisms. Some *Bc *strains are thermophiles [6], while *B. weihenstephanensis *is psychrophilic [7]. By contrast, many members of this group are mesophiles, and can be found in a variety of locales including soil, on plant surfaces and in the mammalian gastrointestinal microflora [8]. Some members of this group appear to be nonpathogenic, while others cause diverse diseases including gastroenteritis, food poisoning [8], endophthalmitis [9], tissue abscesses [10,11], and anthrax [2]. *Bt *strains have the capacity to cause disease in insects [12,13] and possibly nematodes [14-16], while some evidence suggests that *Bc *strains are part of the normal insect gut flora [8,17]. Nevertheless, whole genome comparisons between these organisms reveal a surprising similarity in gene content, and Han *et al*. [18] have concluded "that differential regulation [of gene content] modulates virulence rather than simple acquisition of virulence factor genes", a conclusion confirmed by other studies [19]. Consequently, we will refer to these organisms as the *Bc *species-group, to reflect the extremely close phylogenetic relationships between these organisms.

How do we account for the underlying genomic similarity of these phenotypically diverse microbes? It has been established for some time that the most rapidly evolving and evolutionarily flexible portions of the bacterial genome are regulatory sequences and transcriptional networks [20-22]. Thus, it is no surprise that major differences between *Bc *species-group organisms reside in the regulation of gene expression rather than gene content. A prime example of this divergence is the PlcR-PapR quorum-sensing operon, present in all *Bc *species-group organisms, but harboring point mutations that differentiate group members from one another [23,24]. The *papR *locus encodes a quorum-sensing signal (a secreted peptide) that is internalized and binds to PlcR, a transcriptional activator that controls gene expression and is important for *Bc *virulence. There are four distinct phylogenetic groups of the PapR peptide, each with point mutations that result in a unique quorum-sensing 'pherotype' [23]. The PlcR sensor in each pherotype has co-evolved to exclusively bind only its cognate PapR peptide, and each PlcR pherotype is consequently 'blind' to the quorum sensing signals secreted by other *Bc *pherotypes. *Ba *strains (and a low percentage of *Bc *strains) [24] have taken PlcR-PapR divergence a step further. These organisms carry a unique nonsense mutation in PlcR that inactivates the quorum-sensing function entirely. Since PlcR and the global virulence regulator AtxA on the virulence plasmid pXO1 appear to antagonize one another [24], PlcR inactivation after *Ba *acquired pXO1 appears necessary for full virulence of *Ba*.

This is not to say that horizontal gene transfer and genome reduction have not been important in remodeling genomes within the *Bc *species-group. For instance, the virulence plasmids pXO1 and pXO2 in *Ba *appear to have been acquired by horizontal gene transfer [25], and represent 52% of the unique coding capacity found in the *Ba *genome. Although these genes have a significant impact on the *Ba *pathogenic phenotype, this plasmid gene content comprises only 176 genes, representing a small fraction of the total coding capacity of the *Ba *genome. Genome reduction has played a modest role in divergence of the *Bc *species-group [26], likely being responsible for the reduced genome size of *Bc *NVH391-98. However, genome reduction is probably more important for *speciation *events; e.g., the *M. leprae *genome is fully 26% smaller than that of *M. tuberculosis*, and carries over 1100 pseudogenes with functional orthologs in *M. tuberculosis*. GR has essentially eliminated 50% of the coding capacity of the *M. leprae *genome [27]. Thus, subtler genome alterations within the *Bc *species-group, such as gene duplication, divergence and point mutations probably have contributed as much or more than horizontal gene transfer and genome reduction to the unique niche adaptations of individuals within the *Bc *species-group.

Anderson *et al*. [28] first noted that the genomes of *Bc *species-group organisms appeared to harbor an overabundance of sigma factors, compared to *B. subtilis *strain 168. Bacterial sigma factors bind RNA polymerase and allow the holoenzyme to recognize promoter sequences 5' to the site of initiation of transcription [29]. Typically, bacteria encode several different sigma factors, each of which is responsible for controlling a suite of genes by activating transcription at a unique set of sigma factor specific promoter sequences. Sigma factors generally belong to two primary categories, the sigma^54 ^and the sigma^70 ^families [29]. The sigma^54 ^proteins encoded by the *Bc *species-group are very highly conserved, and ubiquitously present as a single copy gene. Therefore, a phylogenetic analysis of these proteins in the *Bc *species-group was not particularly revealing (data not shown). We consequently focused further efforts on the sigma^70 ^proteins. Sigma^70 ^proteins can be further differentiated into primary alternative (PA) sigma factors and extracytoplasmic function sigma factors (ECF) [30]. In general, PA sigma factors control expression of many housekeeping functions of the cell (e.g., *B. subtilis *SigA), and allow the organism to respond to specific environmental stimuli such as heat-shock (e.g., SigB) [31,32]; in *B. subtilis*, several PA sigma factors are integral to the sporulation developmental pathway [33,34]. ECF sigma factors typically activate gene expression in response to extracellular signals such as the availability of specific iron sources [35,36] and commonly are essential for disease pathogenesis [37-39]. The activity of a PA or (more commonly) an ECF sigma is often controlled by an anti-sigma factor that renders the sigma factor in a state unable to bind RNA polymerase. Activation of the sigma factor for RNA polymerase binding and transcription initiation is triggered by a signal (ligand binding, covalent modification or proteolysis) that inactivates the anti-sigma factor [40].

Thus, sigma factors activate transcription in response to environmental or developmental signals, and selectively activate transcription by recognizing different consensus promoter sequences to tailor gene expression to those signals [41]. This suggested to us that many of the phenotypic differences between members of the *Bc *species-group organisms might be a consequence of the sigma factor gene expansion [28], accompanied by divergence among the sigma factor regulons of these organisms. Consequently, we began to explore the phylogeny of the sigma factors found in various *Bc *species-group members, by comparison to the experimentally well-understood model organism *B. subtilis*. To place these studies in context, we began by constructing a phylogeny of the *Bacillaceae *using whole-genome single copy genes. This phylogeny suggested that the current taxonomic affiliation of many members of the *Bacillaceae *should be reconsidered. Using this phylogeny as a basis, we then examined the phylogenetic relationships of the sigma factors encoded by members of the *Bc *species-group. We find that the overabundance of sigma factors encoded by the *Bc *species-group organisms is specifically in the ECF sigma factors, rather than in the sigma factor group as a whole. The sigma factor gene family encoded by the *Bc *species-group is the end-product of a dynamic gene-duplication and gene-loss process that has, until now, underestimated the true heterogeneity of ECF sigma factor content in the *Bc *species-group. Further, the sigma factor content carried by any given member of the *Bc *species-group suggests that both shared and unique gene expression patterns have evolved during the divergence of this group of organisms from a common ancestor.

## Results and Discussion

Whole-genome single copy-gene phylogeny of the family *Bacillaceae*

**Figure 1:**
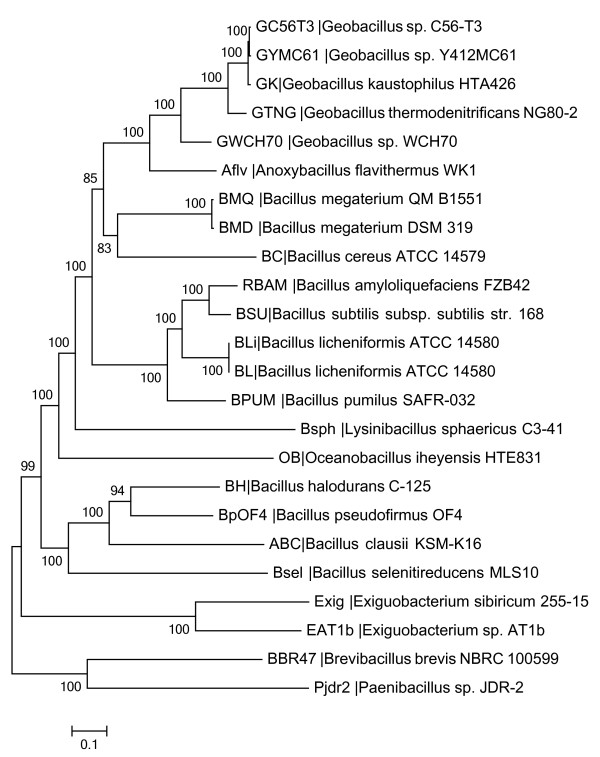
**Whole genome single-copy gene phylogeny of the family *Bacillaceae *and the *Bc *species-group**. Relationships among members of the family *Bacillaceae *based on the results obtained from a maximum-likelihood analysis of 157 single-copy genes found in each of the 43 genomes included in the analysis, using the genomes of *Paenibacillus *JDR-2 and *Brevibacillus brevis *NBRC-100599 to root the analysis. Numbers along the internodes are the number of times that node was supported in 100 bootstrap replicates. This is a phylogram that displays the relationships of all of the *Bacillaceae*; the legend denotes substitutions per nucleotide.

Phylogenetic analysis of 157 single copy genes (Additional file 1) of 41 *Bacillaceae *genomes (Table 1), using *Paenibacillus *and *Brevibacillus *as outgroups, indicate that there are five main lineages and suggest four modifications to the taxonomy of the family (Figure 1). The initial divergence within the *Bacillaceae *was between *Exiguobacterium*, an aerobic, asporogenous, and irregularly shaped Gram-positive bacterium recently linked to bacteraemia [42], and the bulk of the family. Subsequent to this, *B*. *halodurans*, *B*. *clausii*, *B. selenitireducens, and B. pseudofirmus *(the *B. halodurans *group) diverged from the rest of the family, followed by the divergence of *Oceanobacillus *and *Lysinibacillus*. Within the remaining *Bacillus *genera, there is a multichotomous split between the *B. subtilis *group (including *B. subtilis*, *B. amyloliquefaciens*, *B. licheniformis*, and *B. pumilus*), the *Bc *species-group, *B*. *megaterium*, and a group that includes strains of *Geobacillus *and *Anoxybacillus *(*G. kaustophilus*, *G. thermodenitrificans*, *Geobacillus *WCH-70, and *Anoxybacillus flavithermus*). Although results from the maximum likelihood analysis indicate a lack of resolution between these four groups, the inclusion of *Geobacillus *and *Anoxybacillus *within *Bacillus *has strong support (particularly relative to the *B*. *halodurans *group). This indicates that *Oceanobacillus*, *Lysinibacillus*, *Geobacillus*, and *Anoxybacillus *are more closely related to some *Bacillus *spp. than are members of the *B*. *halodurans *group, and that, if one wishes the taxonomy of the group to reflect evolutionary history, should be subsumed within *Bacillus*.

**Table 1:** Genome sequences used in this study

Organism	Locus tag	Size (bp)	Source of isolation	Accession
Anoxybacillus flavithermus WK1	Aflv	2846746	Geothermal waste-water drain	NC_011567
Exiguobacterium AT1b	EAT1B	2999895	Yellowstone Nat'l Park	NC_012673
Exiguobacterium sibiricum 255-15	Exig	3040786	Siberian permafrost	NC_010556
Geobacillus WCH70	GWCH70	3508804	Wood chip composter heap	NC_012793
Bacillus selenitireducens MLS10	Bsel	3592487	Axonic lake mud	NC_014219
Geobacillus kaustophilus HTA426	GK	3592666	Deep-sea sediment	NC_006510
Geobacillus thermodenitrificans NG80-2	GTNG	3608012	Deep oil reservoir	NC_009328
Oceanobacillus iheyensis HTE831	OB	3630528	Deep-sea sediment	NC_004193
Geobacillus C56-T3	GC56T3	3650813	Hot spring	NC_014206
Geobacillus Y412MC61	GYMC61	3667901	Hot spring	NC_013411
Bacillus pumilus SAFR-032	BPUM	3704465	JPL spacecraft assembly facility	NC_009848
Bacillus amyloliquefaciens FZB42	RBAM	3918589	soil	NC_009725
Bacillus cereus cytotoxis NVH 391-98	Bcer98	4094159	Food poisoning outbreak	NC_009674
Bacillus halodurans C-125	BH	4202352	Deep-sea sediment	NC_002570
Bacillus subtilis 168	BSU	4215606	Model organism	NC_000964
Bacillus licheniformis ATCC-14580	BL	4222597	soil	NC_006270
Bacillus licheniformis DSM-13	Bli	4222645	soil	NC_006322
Bacillus pseudofirmus OF4	BpOF4	4249248	soil	NC_013791
Bacillus clausii KSM-K16	ABC	4303871	soil	NC_006582
Lysinibacillus sphaericus C3-41	Bsph	4817463	soil	NC_010382
Bacillus megaterium DSM319	BMD	5097447	soil	NC_014103
Bacillus anthracis Ames	BA	5227293	Bovine carcass	NC_003997
Bacillus cereus 03BB102	BCA	5228663	Human blood isolate	NC_012472
Bacillus thuringiensis Al-Hakam	BALH	5313030	Iraq bioweapons facility	NC_008600
Bacillus thuringiensis konkukian	BT	5314794	Human tissue necrosis	NC_005957
Bacillus cereus biovar anthracis CI	BACI	5419036	Chimpanzee carcass	NC_014335
Bacillus cereus B4264	BCB	5427083	Bloodstream isolate from pneumonia patient	NC_011725
Bacillus cereus ATCC14579	BC	5432652	Dairy product	NC_004722
Bacillus cereus AH187	BCAH187	5449308	Food poisoning isolate	NC_011658
Bacillus anthracis str Sterne	BAS	5486649	Vaccine strain	NC_005945
Bacillus anthracis A0248	BAA	5503926	Human disease	NC_012659
Bacillus anthracis Ames-0581	GBAA	5503926	Bovine carcass	NC_007530
Bacillus cereus Q1	BCQ	5506207	Deep oil reservoir	NC_011969
Bacillus anthracis CDC 684	BAMEG	5506763	NA*	NC_012581
Bacillus megaterium QM-B1551	BMQ	5523192	soil	NC_014019
Bacillus cereus ATCC-10987	BCE	5588834	Cheese spoilage	NC_003909
Bacillus cereus AH820	BCAH820	5599857	Human periodontitis	NC_011773
Bacillus thuringiensis BMB171	BMB	5643051	soil	NC_014171
Bacillus cereus G9842	BCG	5736823	Stool sample from food poisoning outbreak	NC_011772
Bacillus cereus ZK	BCZK	5843235	Zebra carcass	NC_006274
Bacillus weihenstephanensis KBAB4	KBAB	5872743	soil	NC_010184

* NA: not available

These relationships are significantly different than those deduced by most other strategies, until recently. The family *Bacillaceae*, including the genus *Bacillus*, is a heterogeneous collection of gram-positive rod-shaped bacteria within the Firmicutes and includes both free-living and pathogenic species with a world-wide distribution. Their heterogeneity is reflected in a highly variable GC content ranging between 33 and 78% G+C. To date, the most commonly utilized phylogenetic strategy for examining these phylogenetic relationships has utilized rDNA sequences. Xu and Cote [43], for example, identified 10 groups within *Bacillaceae *on the basis of 16S-23S internal transcribed spacer sequences. Seven of those groups included members of the genus *Bacillus*. The ribosomal database project (RDB) [44] currently includes 13,359 sequences for members of *Bacillaceae *(as of 10/01/2010). However, recent study of relationships of members of *Bacillus *has begun to look beyond 16S rDNA sequences and has benefitted from the many whole-genome sequences becoming available. For example, Alcaraz et al. [45] examined twenty *Bacillus *genomes and, utilizing a core-genome conceptual data analysis, determined the phylogeny of known *Bacillus *spp. included in their study and identified four main lineages. Although their study employed different outgroups, methods, and genomes sampled, their conclusions were similar to ours and consistent with the idea that the taxonomic affiliation of these organisms needs to be reconsidered, in the light of whole-genome analyses. This is not to suggest that phylogenetic analyses based on 16S rDNA sequence should be supplanted by whole genome analyses, due to the obvious practical limitations of requiring the entire genome sequence of an isolate prior to phylogenetic analysis. However, whole genome phylogenetic methods such as that presented here, and by other groups such as Alcaraz et al. [45] indicate that the resolution of 16S phylogenies should be viewed with caution. Our results also are consistent with the conclusions of Tourasse et al. [46], who have recently described an extremely robust analysis of this group of organisms using a combination of MSLT, AFLP and MLEE genotyping. Again, these methodologies have the advantage of not requiring whole genome sequence for analysis. Nevertheless, the comprehensive nature of using whole genome sequences for phylogenetic comparisons is attractive due to the power of the technique, when the data is available.

**Figure 2:**
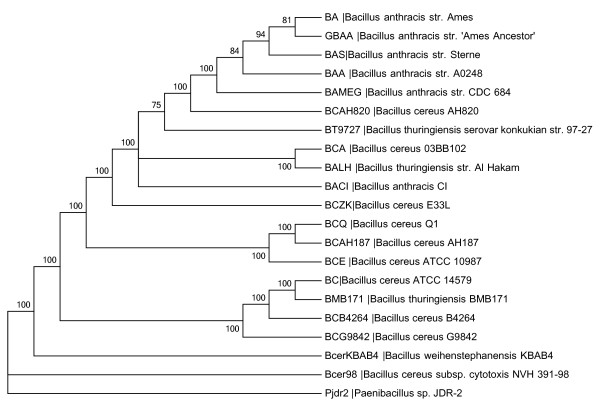
**Whole-genome single-copy gene phylogeny of the *Bc*-species group**. This analysis was performed as for Figure. 1, except that as the relationships between members of the *Bc *species-group were not resolved by this maximum iikelihood analysis (data not shown), Figure 2 is a cladogram that more clearly delineates the relationships within the *Bc *species-group.

Within the *Bc *species-group (Figure 2), *Bc *subsp. *cytotoxis *NVH 391-98 is the most distantly related of the *Bc *species-group, followed by *B. weihenstephanensis*. The remaining *Bc *strains form a paraphyletic assemblage that excludes *B*. *thuringiensis *and *B*. *anthracis*. While both the gene content and extent of divergence suggest that *Bc *subsp. *cytotoxis *and perhaps *B. weihenstephanensis *may warrant specific recognition, other organisms within the *Bc *species-group do not. For example, the three *Bt *strains did not group together. *Bt *Konkukian is most closely related to *Ba*, while the other two *Bt *strains are more distantly related. The closest relative of *Bt *Al Hakam is *Bc *03BB102, while *Bt *strain BMB171 is mostly nearly related to *Bc *strain ATCC14579. Preliminary results for two other *Bt *strains, *kurstaki *T03a001 and HD1, also fall within this region of the phylogeny (data not shown). *Ba *strains form a monophyletic lineage and could be a sub-species of *Bc*. While subsuming *Ba *and *Bt *within *Bc *may be problematic, there are definitively *Bc *strains (e.g. *Bc *AH820) that are significantly more closely related to *Ba *or *Bt *than they are to other strains of *Bc*. Thus, our phylogenetic assessment is consistent with other recent suggestions that the *Bc *group exhibits sufficiently high genetic similarity that these organisms could be members of a single species ([5,47-49]).

### Expansion of the sigma factor gene family in the *Bc *species-group of the *Bacillaceae*

#### Initial dataset containing the *Bc *species-group sigma factors

Iterative BLAST searches initiated from 18 *B*. *subtilis *sigma factors initially identified 515 potential sigma factors within the 20 strains of *Bc *species-group genomes (see Additional file 2). A total of 16 genes identified in the iterative BLAST searches were excluded from the final analysis due to either their short length (in some cases producing non-overlapping genes when aligned with all other sigma factor homologs), and/or lack of evidence from the Multiple Expectation Maximization for Motif Elicitation (MEME) analysis warranting their inclusion as a sigma factor (see below). TBLASTN searches to the nucleotide sequences of the *Bc *species-group identified 3 additional non-annotated sigma factors that are orthologs of BSU13450 (SigI - present in the BCAH187 *B. cereus *genome), and BAS5102 and BAS1035 (both present in the *B. thuringiensis *Al-Hakam genome), respectively.

The seven most informative motifs from MEME analysis proved useful in segregating functional sigma factors from sequences that bore superficial similarity to sigma factors (false positives), and allowed us to differentiate PA sigma factors from ECF sigma factors (Tables 2 and 3, also see Additional file 3 for the complete MEME results). Comparing these MEME motifs to previously identified regions of sequence conservation among sigma factors [50] also was informative. Motifs 1 and 5, which are located near or slightly to the N-terminal side of the -35 and -10 promoter binding sites (sigma factor regions 4 and 2), respectively, were present in most sigma factors. MEME motifs 2 and 7 also were identified within region 2 (the -10 binding site), and differentiate PA from ECF sigma factors. MEME motifs 3 and 6 are at the -35 binding site and are also representative of PA and ECF sigma factors, respectively. MEME motif 4, lying to the N-terminal region of the -10 binding site, is largely restricted to PA sigma factors but is also present in 2 ECF sigma factor paralogs. Aside from the well-documented differences in size between ECF and PA sigma factors, these data suggest that the principle functional difference between the two is directly associated with the binding of the protein to DNA recognition sites.

**Table 2:** MEME motifs found in PA sigma factors

PA Locus Tag	1	2	3	4	5	6	7	Orthologous BSU locus tag
**BAS4194**	+	+	+	+	+			**BSU25200 (SigA)**
**BAS0928**	+	+	+	+	+			**BSU04730 (SigB)**
	+	+	+		+			**BSU16470 (SigD)**
**BAS3755**	+	+	+	+	+			**BSU15320 (SigE)**
**BAS3983**	+	+	+	+	+			**BSU23450 (SigF)**
**BAS3754**	+	+	+	+	+			**BSU15330 (SigG)**
**BAS0093**	+				+			**BSU00980 (SigH)**
**BAS3231**		+			+			**BSU13450(SigI)**
**BAS4236**	+	+	+	+	+			**BSU25760, 26390 (SigK)**
**BAS3522**								**BSU12560 (Xpf)**
**BAS3823**	+	+			+			
**BAS5102**	+	+			+			
**Bcer98_2607**	+	+						
**BCG9842_0035**	+	+	+	+	+			
**BMB171_P0077**	+	+	+		+			

A '+' designates the presence of a motif in the PA sigma factor gene at the left. MEME motifs are presented here for a representative (from the BAS genome where available) genome for each of the PA sigma factors detected by the analyses.

**Table 3:** MEME motifs found in ECF sigma factors

ECF Locus Tag	1	2	3	4	5	6	7	Orthologous BSU locus tag
**BAS0964**	+					+	+	
**BAS2285**	+			+	+		+	
**BAS3082**	+					+	+	**BSU09520 (SigM)**
	+				+	+	+	**BSU27120 (SigV)**
	+				+	+	+	**BSU01730 (SigW)**
	+				+	+	+	**BSU23100 (SigX)**
	+				+	+	+	**BSU38700 (SigY)**
	+				+	+	+	**BSU26840 (SigZ)**
	+				+	+	+	**BSU14730 (YlaC)**
**BAS0171**	+					+	+	
**BAS0613**	+				+		+	
**BAS1035**	+				+	+	+	
**BAS1626**					+		+	
**BAS1658**	+				+	+	+	
**BAS1966**	+				+	+		
**BAS2323**	+				+	+	+	
**BAS2545**	+				+		+	
**BAS2600**					+	+	+	
**BAS2758**	+				+	+	+	
**BAS3383**					+	+	+	
**BAS4558**	+				+	+	+	
**BAS5212**	+				+	+	+	
**BALH_4199**	+				+	+	+	
**BCAH187_A3458**				+	+			
**BCAH820_1326**							+	
**BCE_1118**	+				+	+	+	
**BCE_5322**					+	+	+	
**Bcer98_3970**					+		+	
**BcerKBAB4_3133**	+				+			
**BcerKBAB4_4716**	+				+	+	+	
**BcerKBAB4_5577**	+				+	+		
**BCQ_1681**					+		+	

A '+' designates the presence of a motif in the ECF sigma factor gene at the left. MEME motifs are presented here for a representative (from the BAS genome where available; otherwise as the locus tag indicates) gene for each of the ECF sigma factors detected by the analyses.

#### Sigma factor genes in the *Bacillaceae*

**Figure 3:**
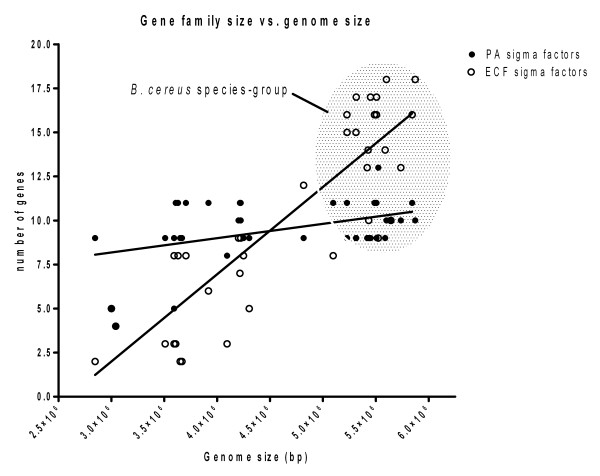
**Correlation of genome size with the number of PA and ECF sigma factors in *Bacillaceae***. The number of PA (black circles) and ECF (open circles) sigma factors genes identified in the genomes listed in Table 1 are plotted against genome size. The highlighted grey area is the observed number of PA and ECF sigma factor genes found for members of the Bc species-group. These results show that the number of ECF, but not PA, sigma factor genes is correlated with genome size.

Taken as a whole, the number of PA sigma factor genes found within the genomes of the *Bacillaceae *was roughly independent of the genome sizes of these organisms (Figure 3). By contrast, the numbers of ECF sigma factor genes found in the *Bacillaceae *increased in direct proportion to genome size. Thus, the overabundance of sigma factor genes earlier observed in the *Bc *species-group organisms [28] resulted from a preferential expansion in the ECF sigma factors, compared to the PA sigma factor genes. This might indicate that members of the *Bc *species-group have evolved a more sophisticated ability to sense and respond transcriptionally to extracellular signals, compared to other members of the *Bacillaceae *with smaller genomes and a relative paucity of ECF sigma factor genes. Alternatively, this may indicate that other regulatory regimes (e.g., two-component regulators) are preferentially used by members of the *Bacillaceae *with smaller genomes, for coordinating transcription with extracellular signals. Further work is necessary to differentiate between these possibilities.

#### Phylogenetic analysis of the *Bc *species-group sigma factors

**Figure 4:**
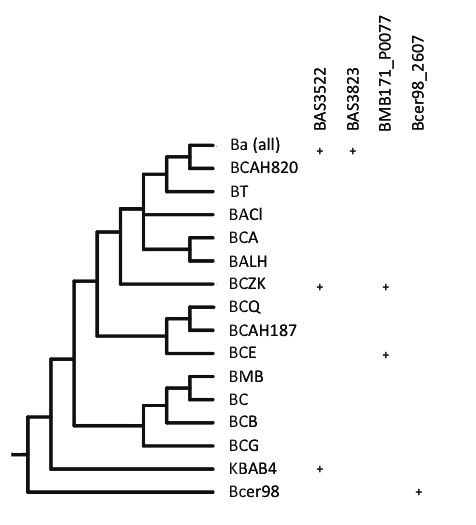
**Phylogenetic distribution of PA sigma factors in the *Bc *species-group**. Sigma factors genes found in fewer than all of the genomes listed in Table 1, mapped on a *Bc *species-group cladeogram similar to that shown in Figure 2. The five *Ba *strains in Table 1 have a gene content identical to strain *Ba *strain Sterne, and so are condensed to one line in this tree. A + indicates the presence of a gene, as listed in the column heading, in that genome. Genome abbreviations are as found in Table 1.

Within the *Bc *species-group, phylogenetic analysis of the sigma factors of the *Bc *species-group identified 41 paralogous sigma factor genes in these organisms (Tables 4, 5, and 6, Additional files 2 and 4). Any one genome contained at most 27 sigma factor genes, hinting at an extensive history of gene duplication and loss in these lineages. Of these 41 genes, 14 were PA sigma factors and 27 were ECF sigma factors. Four of the PA sigma factors genes and 21 ECF sigma factor genes were unique to the *Bc *species-group, indicating that the majority of sigma factor gene expansion within the *Bc *species-group is concentrated on the ECF sigma factor genes, as noted above. By comparison, 18 sigma factor genes were found for *B. subtilis*, 10 of which were PA sigma factors. The *Bc *species-group harbors 9 PA sigma factors that are orthologous to the more extensively studied sigma factors of *B. subtilis *and appear to be the most evolutionarily conserved. (Six of these PA sigma factors appear to be very highly conserved as they were present in all *Bacillus *species examined). At least one of these PA sigma factors, BAS0093, the ortholog of the *B. subtilis *SigH locus, is evolutionarily conserved amongst many of the *Firmicutes *[51]. Further, the location of these conserved PA sigma factors within their respective genomes was syntenic between genomes. Indeed, finding a PA sigma factor that was not present in all members of the *Bc *species-group was rare (Figure 4). One *B. subtilis *PA sigma factor, BSU16470 (SigD), lacked an orthologous sequence in all members of the *Bc *species-group. A second PA sigma factor, BSU12560 (Xpf), was uniformly found in all *Ba *strains but only in one other *Bc *strain (*Bc *ZK) and in *B. weiheinstephanensis*. Two (BAS0928 and BAS3231) were absent in *Bc *subsp. *cytotoxis*. In rare cases (e.g. plasmid-borne pE33L466_0212 of *Bc *ZK, with similarity to the SigA genes of *B. clausii *and *B. halodurans*), a few PAs appear to be the result of horizontal gene transfer from organisms outside of the *Bc *species-group. However these are the only data that we found indicative of horizontal transfer, suggesting indirectly that horizontal gene transfer has not been a significant contributor to sigma factor evolution in these organisms.

**Table 4:** PA and ECF sigma factor counts in *Bacillaceae *genomes

Genome	Locus Tag	PA	ECF	Total
***Bacillus cereus *species-group:**				
***B. anthracis *A0248**	**BAA**	11	16	27
***B. anthracis *Ames**	**BA**	11	16	27
***B. anthracis *Ames-0581**	**GBAA**	11	16	27
***B. anthracis *CDC 684**	**BAMEG**	11	16	27
***B. anthracis *Sterne**	**BAS**	11	16	27
***B. cereus *biovar *anthracis *CI**	**BACI**	9	13	22
***B. cereus *03BB102**	**BCA**	9	15	24
***B. cereus *AH187**	**BCAH187**	9	17	26
***B. cereus *AH820**	**BCAH820**	10	18	28
***B. cereus *ATCC-10987**	**BCE**	9	14	23
***B. cereus *ATCC14579**	**BCB**	9	10	19
***B. cereus *B4264**	**BCB**	9	14	23
***B. cereus *G9842**	**BCG**	10	13	23
***B. cereus *Q1**	**BCQ**	9	17	26
***B. cereus *ZK**	**BCZK**	11	16	27
***B. thuringiensis *Al-Hakam**	**BALH**	9	15	24
***B. thuringiensis *BMB171**	**BMB**	10	10	20
***B. thuringiensis *konkukian**	**BT**	9	17	26
***B. weihenstephanensis *KBAB4**	**KBAB**	10	18	28
***B. cereus cytotoxis *NVH 391-98**	**Bcer98**	8	3	11
***Bacillus subtilis *group:**				
***B. amyloliquefaciens *FZB42**	**RBAM**	11	6	17
***B. licheniformis *ATCC-14580**	**BL**	11	9	20
***B. licheniformis *DSM-13**	**Bli**	10	9	19
***B. pumilus *SAFR-032**	**BPUM**	11	8	19
***B. subtilis *168**	**BSU**	11	7	18
***Bacillus megaterium:***				
***B. megaterium *DSM319**	**BMD**	11	8	19
***B. megaterium *QM-B1551**	**BMQ**	13	9	22
***Geobacillus *group:**				
***Anoxybacillus flavithermus *WK1**	**Aflv**	9	2	11
***Geobacillus *C56-T3**	**GC56T3**	9	2	11
***G. kaustophilus *HTA426**	**GK**	9	3	12
***G. thermodenitrificans *NG80-2**	**GTNG**	11	3	14
***Geobacillus *WCH70**	**GWCH70**	9	3	12
***Geobacillus *Y412MC61**	**GYMC61**	9	2	11
**Other *Bacillaceae*:**				
***Lysinibacillus sphaericus *C3-41**	**Bsph**	9	12	21
***Oceanobacillus iheyensis *HTE831**	**OB**	11	8	19
***Bacillus halodurans *group:**				
***B. clausii *KSM-K16**	**ABC**	9	5	14
***B. halodurans *C-125**	**BH**	10	9	19
***B. pseudofirmus *OF4**	**BpOF4**	9	8	17
***B. selenitireducens *MLS10**	**Bsel**	5	8	13
***Exiguobacterium:***				
***Exiguobacterium *AT1b**	**EAT1B**	5	5	10
***E. sibiricum *255-15**	**Exig**	4	4	8
***Paenibacillaceae *Outgroups:**				
***Brevibacillus brevis *NBRC-100599**	**BBR**	11	41	52
***Paenibacillus *JDR 2**	**Pjdr2**	10	19	29

**Table 5:** PA sigma factor genes in the *Bc *species-group compared to *B. subtilis*

PA Locus tag	BAS	GBAA	BA	BAA	BAMEG	BCAH820	BACl	BT	BALH	BCA	BCZK	BCAH187	BMB	BCQ	BCE	BCG	BCB	BC	KBAB	Bcer98	BSU	Orthologous BSU locus tag
**BAS4194**	+	+	+	+	+	+	+	+	+	+	+	+	+	+	+	+	+	+	+	+	+	**BSU25200 (SigA)**
**BAS0928**	+	+	+	+	+	+	+	+	+	+	+	+	+	+	+	+	+	+	+		+	**BSU04730 (SigB)**
																					+	**BSU16470 (SigD)**
**BAS3755**	+	+	+	+	+	+	+	+	+	+	+	+	+	+	+	+	+	+	+	+	+	**BSU15320 (SigE)**
**BAS3983**	+	+	+	+	+	+	+	+	+	+	+	+	+	+	+	+	+	+	+	+	+	**BSU23450 (SigF)**
**BAS3754**	+	+	+	+	+	+	+	+	+	+	+	+	+	+	+	+	+	+	+	+	+	**BSU15330 (SigG)**
**BAS0093**	+	+	+	+	+	+	+	+	+	+	+	+	+	+	+	+	+	+	+	+	+	**BSU00980 (SigH)**
**BAS3231**	+	+	+	+	+	+	+	+	+	+	+	+	+	+	+	+	+	+	+		+	**BSU13450 (SigI)**
**BAS4236**	+	+	+	+	+	+	+	+	+	+	+	+	+	+	+	+	+	+	+	+	+	**BSU25760, BSU26390 (SigK)**
**BAS3522**	+	+	+	+	+						+								+		+	**BSU12560 (Xpf)**
**BAS3823**	+	+	+	+	+																	
**BAS5102**	+	+	+	+	+	+	+	+	+	+	+	+	+	+	+	+	+	+	+	+		
**Bcer98_2607**																				+		
**BCG9842_0035**																+						
**BMB171_P0077**											+		+									

A '+' designates the presence of a PA sigma factor ortholog group. The PA locus tag shown is from *B. anthracis *strain Sterne, unless this gene was not found in that organism. In those instances another locus tag was chosen as a representative. Genome abbreviations are as in Table 1.

**Table 6:** ECF sigma factor genes in the *Bc *species-group compared to *B. subtilis*

ECF Locus tag	BAS	GBAA	BA	BAA	BAMEG	BCAH820	BACl	BT	BALH	BCA	BCZK	BCAH187	BMB	BCQ	BCE	BCG	BCB	BC	KBAB	Bcer98	BSU	Orthologous BSU locus tag
**BAS0964**	+	+	+	+	+	+	+	+	+	+	+	+	+	+	+	+	+		+			
**BAS2285**	+	+	+	+	+	+	+	+	+	+	+	+	+	+		+	+	+	+			
**BAS3082**	+	+	+	+	+	+	+	+		+	+	+		+					+		+	**BSU09520 (SigM)**
**BAS0171**	+	+	+	+	+	+			+	+	+	+		+	+				+			
**BAS0613**	+	+	+	+	+	+		+	+	+	+	+		+	+	+	+	+	+			
**BAS1035**	+	+	+	+	+	+	+	+	+	+	+	+		+	+	+	+	+	+			
**BAS1626**	+	+	+	+	+	+	+	+	+	+	+	+	+	+	+	+	+	+	+			
**BAS1658**	+	+	+	+	+	+	+	+	+	+	+	+	+	+	+	+	+	+	+			
**BAS1966**	+	+	+	+	+	+	+	+	+	+	+	+	+	+	+	+	+	+	+			
**BAS2323**	+	+	+	+	+	+	+	+	+	+	+	+	+	+	+	+	+	+	+			
**BAS2545**	+	+	+	+	+	+	+	+	+	+	+			+								
**BAS2600**	+	+	+	+	+	+	+	+	+	+	+	+	+	+	+	+	+	+	+	+		
**BAS2758**	+	+	+	+	+	+																
**BAS3383**	+	+	+	+	+	+	+	+	+	+	+	+	+	+	+	+	+	+	+			
**BAS4558**	+	+	+	+	+	+	+		+		+	+		+	+		+		+			
**BAS5212**	+	+	+	+	+	+	+	+	+	+	+	+	+	+	+	+		+				
**BALH_4199**						+		+	+	+		+		+					+			
**BCAH187_A3458**												+										
**BCAH820_1326**						+																
**BCE_1118**															+							
**BCE_5322**															+							
**Bcer98_3970**																				+		
**BcerKBAB4_3133**													+			+	+		+	+		
**BcerKBAB4_4716**								+											+			
**BcerKBAB4_5577**																+			+			
**BCQ_1681**								+			+	+		+			+		+			
**BT9727_0859**								+														

The pattern of ECF sigma factor distribution was decidedly different and more complex. Of the 7 ECF sigma factors found in *B. subtilis*, 6 were not present in the *Bc *species-group. Thus, the divergence of the *Bc *species-group from *B. subtilis *resulted in a relatively stable set of PA sigma factor genes shared by both, with a regimen of gene expansion that resulted in additional ECF sigma factors encoded in the genomes of the *Bc *species-group. Interestingly, our analyses suggest that this pattern of expansion of ECF sigma factor genes within a given lineage may independently occur in another lineage of *Bacillales*. Our initial screen of sigma factors identified 52 sigma factors encoded in *Brevibacillus brevis *[52]. Of these 52 genes, 41 are ECF sigma factors. The *B. brevis *ECF sigma factor gene family may therefore represent an independent and dramatic expansion, comparing whole-genome phylogenetic analysis (see above) and the absence of sequence similarity of the *B. brevis *ECF sigma factors to those of the *Bc *species-group (data not shown).

**Figure 5:**
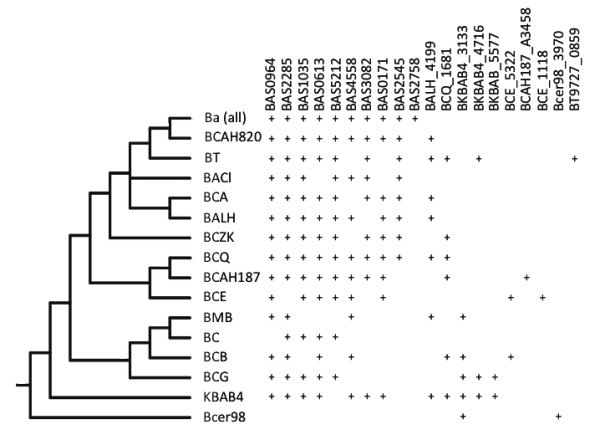
**Phylogenetic distribution of ECF sigma factors in the *Bc *species-group**. Presentation and analyses are as described for Figure 4.

In contrast to the relative conservation of the PA sigma factors, the patterns of gene duplication/loss among paralogous ECF sigma factors of the *Bc *species-group were difficult to deduce (Figure 5). No clear syntenic pattern was observed when comparing the location of these ECFs in the various genomes. Neighbor-joining (NJ) analysis (phylogenetic relationships of the 499 *Bc *species-group sigma factors can be found in Additional file 4) indicates some support for relationships between four groups of *Bc *species-group ECF sigma factors, including: 1) BAS0964 and BAS2600 (supported in 70 NJ bootstrap replicates), 2) a grouping of three paralogs including BAS2758 and BcerKBAB4-5577, followed by BAS1966 (supported in 90 and 93 NJ replicates, respectively), 3) BAS2285 and BAS0613 (supported in 83 NJ bootstrap replicates, and 4) BAS2545 and BcerKBAB4-3133 (supported in 100 NJ replicates). However, evidence of more recent common ancestry between any pair of sigma factor paralogs is the exception rather than the rule. The remaining 18 *Bc *species-group ECF sigma factor genes are of indeterminate relation to one another, and the preponderance of evidence seems to point to an active period of ECF sigma factor duplications in the ancestors of the *Bc *species-group. However, the evolutionary origin of many of the ECF sigma factors in the *Bc *species-group is difficult to discern, as the phylogenetic placement of these genes was more complex than for PA sigma factors. While it was relatively unusual to find PA sigma factors that were only encoded in some genomes, the pattern of ECF sigma factor genes harbored by some but not all *Bc *species-group organisms was complex (compare Figures 4 and 5).

## Conclusions

The preponderance of evidence presented here and elsewhere is that the ECF sigma factors of the *Bc *species-group have common ancestry with one another and they are the product of gene duplications, although at this time the bulk of that evidence is raw sequence similarity. Our hypothesis is that many of the ancestors of these genes regulated a larger sub-set of genes than their descendents do presently. Following duplication, each cognate descendent sigma factor was then free to specialize (fine-tune) for a smaller subset of genes and for a more specialized role, and in the process of evolving into this specialized niche these genes then become critically important in the survival of descendent generations and are retained in their respective genomes. This subfunctionalization [53] of gene regulation also is potentially reinforced by duplication and/or specialization of the genes which they regulate, which are likewise free from constraints that arise from being co-regulated with a larger set of genes. Interestingly, this suggests that, although our ability to discern relationships among paralogous ECF sigma factors at this time is, at best, murky, in the future these relationships may be deduced from genes that each sigma factor is found to regulate.

## Methods

### Whole-genome single copy-gene phylogeny

Our initial aim was to determine the sigma factor content of the ancestral *Bc *species-group genome and then to determine the changes that had subsequently occurred during divergence of these genomes. However, the genus *Bacillus *has undergone numerous and complex recent taxonomic revisions and been the subject of discordant phylogenetic results [1,43], making any definitive definition of the genus a potential complication. Consequently, we constructed a phylogenetic tree of the *Bacillaceae *that was independent of earlier efforts, but relied solely on whole genome sequences to discern relationships. Our efforts focused on the family *Bacillaceae *as defined by the ribosomal 16S rDNA sequences contained in the Ribosomal Database Project Release 10 [44], to direct our sampling of whole-genome data (Table 1) available at NCBI. This yielded a total dataset of 41 genomes. We purposely excluded draft genome sequences from this analysis to ensure that the absence of a given sigma factor was not an artifact of the incomplete sequence available for that organism. Two close relatives of the *Bacillaceae*, *Paenibacillus *and *Brevibacillus*, from the closely related family *Paenibacillaceae*, were used as outgroups for the purpose of rooting. We then performed phylogenetic analyses on the larger *Bacillaceae *to identify the closest relatives to the *Bc *species-group.

Determination of a gene's orthology is the most important complicating factor in identifying phylogenetic relationships derived from whole genome data. We avoided this problem by restricting our analysis to single-copy genes, for which determination of orthology versus paralogy is not needed [54]. Aligned amino acid sequences were used because the extent of divergence of the genes examined made alignments of DNA sequences unreliable in many cases. Single-copy genes were identified using BLAST searches of each annotated protein-coding gene of one genome to all other genomes listed in Table 1. Results of the BLAST were parsed to identify instances where a gene's BLAST result produced a hit for one and only one of each genome in the analysis. Qualifying genes (Additional file 1) were extracted from the dataset and aligned with ClustalW [55] and put into a concatenated cumulative dataset for phylogenetic analysis with PHYLIP [56]. Phylogenetic analysis of this data set with the Proml progam of PHYLIP utilized the maximum-likelihood algorithm and 100 bootstrap replicates.

### Identification of sigma factor genes and MEME analysis

Genes encoding prospective sigma factors of the *Bc *species-group were identified with an iterative automated BLAST search of amino acid sequences, using as an initial reference the annotated sigma factors of *B*. *subtilis*, the most studied of *Bacillus *genomes. The *B. subtilis *proteins were initially compared by BLAST to the predicted protein coding sequences of the *Bc *species-group. Proteins identified in this analysis were iteratively compared by BLAST against the *Bc *species-group until no additional prospective sigma factors were found. This process, while minimizing the possibility of false negative results (missed sigma factors), inevitably resulted in the inclusion of sequences that, although bearing superficial similarity to a known sigma factor, were likely not functional sigma factors (false positives). Consequently, this analysis was supplemented with MEME [57]] analysis using the zoops setting. The zoops setting does not require the presence of a motif since it is unlikely for these genes to have repeated motifs. All other MEME settings used the default parameters. We searched for up to 10 motifs, 7 of which proved informative for identifying these sigma factors, and differentiating between PA and ECF sigma factors (Tables 3 and 4 and Additional file 3). MEME motifs were utilized to segregate genes that most likely encoded functional sigma factors from those that were not. An additional benefit of the MEME analysis is that it provided independent evidence in addition to that of the BLAST analyses to segregate sigma^70 ^PA sigma factors from ECF sigma factors. This gene identification process also was vulnerable to variation in annotations between the published genomes, which could result in the omission of sigma factors that were not present in the original annotations. Thus, we used TBLASTN searches of the identified sigma factors against the complete nucleotide sequences of all genomes, which were consequently examined to see if any such cryptic non-annotated sigma factors were present in members of the *Bc *species-group. The presence/absence data reported here was updated to reflect these gaps in the publicly-available annotations. Lastly, sigma factor proteins identified in these analyses were aligned using ClustalW and phylogenetic relations among them were examined using the neighbor-joining algorithm of Molecular Evolutionary Genetics Analysis (MEGA) [58]. Other algorithms (such as maximum-likelihood) were computationally infeasible due to the large size of the data set (499 genes).

## Competing interests

The authors declare that they have no competing interests.

## Authors' contributions

TS performed the data analyses included in the manuscript, except for Figures 1A/B, which were analyzed together by TS and ES. All authors read and approved the final manuscript.

## Supplementary Material

Additional file 1 **Single-copy genes used in the phylogenetic analysis of the *Bacillaceae***. Annotations for each of the single-copy gene are from the *Paenibacillus *genome as submitted to Genbank, one of the outgroups included in the analysis. Click here for file

Additional file 2 **Sigma factor genes identified in this study**. Locus tags for genes found in each genome follow the locus tag identifier or sigma factor identifier for each ortholog. Click here for file

Additional file 3 **Results of MEME analysis of the sigma factor genes identified in iterative BLAST searches**. MEME results for 10 motifs (nmotifs = 10) are shown, 7 of which follow phylogenetic patterns that differentiate PA from ECF sigma factors (Tables 2 and 3). Click here for file

Additional file 4 **Results of phylogenetic analysis of the sigma factors identified in Additional file **2. Phylogenetic analysis utilized the neighbor-joining algorithm of MEGA (see text). Click here for file
