# Supplementary material for: Whole-genome phylogenies of the family Bacillaceae and expansion of the sigma factor gene family in the Bacillus cereus species-group
Source: BMC Genomics. 2011 Aug 24;12:430. doi: 10.1186/1471-2164-12-430 (PMC3171730; doi:10.1186/1471-2164-12-430)
Supplement: Additional file 2 — Sigma factor genes identified in this study. Locus tags for genes found in each genome follow the locus tag identifier or sigma factor identifier for each ortholog. [file 1471-2164-12-430-S2.DOC]

**Gene list**

**BAS2323**

BA2502

BAA_2557

GBAA2502

BAMEG_2101

BAS2323

BCAH820_2517

BACI_c24720

BCA_2587

BALH_2250

BCZK2243

BCQ_2413

BCE_2534

BC2469

BMB171_C2230

BCAH187_A2590

BCG9842_B2824

BCB4264_A2499

BcerKBAB4_2327

BT9727_2287

**BAS1035**

BcerKBAB4_1020

GBAA1113

BA1113

BAMEG_3466

BAA_1198

BAS1035

BCZK1013

BCAH187_A1270

BCQ_1177

BCB4264_A1169

BC1114

BCG9842_B4172

BCE_1215

BACI_c11460

BCA_1159

BCAH820_1193

BT9727_1012

**SigM**

BcerKBAB4_3069

BCAH820_3302

BACI_c32210

BT9727_3026

BCQ_2255

BCAH187_A2429

BCZK2972

BCA_3356

BAMEG_1301

BAS3082

BA3324

BAA_3359

GBAA3324

BSU09520

**SigC**

BA1032

BAS0964

GBAA1032

BAMEG_3540

BAA_1124

BCAH820_1110

BCZK0942

BACI_c10700

BCA_1069

BT9727_0953

BALH_0922

BCQ_1107

BCAH187_A1202

BCB4264_A1071

BCG9842_B4230

BcerKBAB4_0953

BCE_1131

BMB171_C0915

**BAS2600**

Bcer98_1911

BcerKBAB4_2594

BCQ_2632

BCE_2820

BCZK2519

BC2794

BCB4264_A2799

BMB171_C2498

BCG9842_B2493

BCAH820_2796

BCAH187_A2841

BALH_2506

BCA_2873

BT9727_2552

BACI_c27530

BA2789

BAMEG_1807

BAA_2852

BAS2600

GBAA2789

**BAS3383**

BA3649

GBAA3649

BAMEG_0984

BAA_3677

BAS3383

BCG9842_B1617

BCZK3297

BCAH187_A3615

BCE_3607

BCQ_3384

BT9727_3349

BACI_c35350

BCAH820_3600

BALH_3229

BCA_3683

BC3589

BCB4264_A3700

BMB171_C3266

BcerKBAB4_3275

**BcerKBAB4_4716**

BT9727_4602

BcerKBAB4_4716

**BAS5212**

BCA_5512

BALH_4859

GBAA5610

BAA_5636

BAMEG_5654

BAS5212

BA5610

BCZK5060

BACI_c53580

BT9727_5044

BCAH820_5454

BCQ_5203

BCAH187_A5541

BC5363

BMB171_C4957

BCE_5492

BCG9842_B5466

**BAS4558**

BAA_4924

BA4913

GBAA4913

BAS4558

BAMEG_4945

BCQ_4476

BCAH820_4778

BACI_c46590

BCAH187_A4795

BcerKBAB4_4490

BCB4264_A4772

BCZK4409

BCE_4798

BALH_4240

**BAS2758**

GBAA2970

BAA_3022

BA2970

BAMEG_1635

BAS2758

BCAH820_2966

**BcerKBAB4_5577**

BCG9842_B0273

BcerKBAB4_5577

**BAS1966**

BCAH187_A2266

BCQ_2091

BCE_2195

BCG9842_B3190

BcerKBAB4_1958

BC2108

BMB171_C1895

BCB4264_A2124

BCZK1921

BACI_c20780

BT9727_1945

BCA_2202

BALH_1878

BCAH820_2148

BAS1966

BAMEG_2477

BAA_2180

GBAA2114

BA2114

**SigJ**

BAA_2511

GBAA2454

BA2454

BAMEG_2147

BAS2285

BCQ_2373

BCAH187_A2551

BACI_c23980

BCZK2206

BCAH820_2472

BT9727_2248

BCA_2518

BALH_2188

BcerKBAB4_2263

BCB4264_A2411

BMB171_C2146

BCG9842_B2919

BC2386

**BAS0613**

BcerKBAB4_0560

BC0647

BCZK0557

BCAH187_A0775

BCG9842_B4656

BCE_0714

BCAH820_0703

BCB4264_A0682

BCQ_0714

BALH_0588

BT9727_0558

BCA_0684

GBAA0646

BAS0613

BAMEG_3941

BA0646

BAA_0728

**BAS1658**

BACI_c17850

BALH_1573

GBAA1789

BA1789

BAS1658

BAA_1861

BAMEG_2801

BCAH820_1838

BT9727_1636

BCA_1798

BCQ_1798

BCAH187_A1912

BCB4264_A1801

BC1731

BMB171_C1597

BCZK1605

BCE_1861

BCG9842_B3540

BcerKBAB4_1655

**BALH_4199**

BCQ_4424

BCAH187_A4747

BCAH820_4736

BT9727_4352

BALH_4199

BCA_4732

BcerKBAB4_4446

**BAS0171**

BcerKBAB4_0156

BCQ_0193

BCAH187_A0213

BAS0171

BCE_0193

BCZK0162

BCA_0212

BALH_0169

BCAH820_0191

BAA_0200

GBAA0169

BA0169

BAMEG_0200

**BCQ_1681**

BCZK1493

BT9727_1502

BCAH187_A1792

BCQ_1681

BCB4264_A1685

BcerKBAB4_1533

**BCE_5322**

BCB4264_A4639

BCE_5322

**BAS1626**

BcerKBAB4_1626

BCAH820_1803

BT9727_1587

BCQ_1771

BCAH187_A1886

BCZK1578

BCB4264_A1768

BC1698

BMB171_C1565

BCG9842_B3574

BCE_1829

BALH_1540

BCA_1765

BACI_c17530

BAMEG_2835

BAS1626

GBAA1753

BA1753

BAA_1827

**SigI**

GBAA3483

BA3483

BAS3231

BCAH820_3445

BAA_3517

BAMEG_1143

BT9727_3204

BCZK3137

BCQ_3232

BACI_c33840

BCA_3504

BALH_3081

BCE_3441

BMB171_C3111

BCG9842_B1813

BC3426

BCB4264_A3433

BcerKBAB4_3122

BSU13450

**SigH**

BA0093

BALH_0093

BCAH187_A0124

BAMEG_0109

GBAA0093

BACI_c01200

BAA_0109

BAS0093

BT9727_0090

BCZK0089

BCAH820_0104

BCA_0122

BCQ_0107

BCG9842_B5212

BCE_0093

BC0114

BCB4264_A0114

BMB171_C0090

BcerKBAB4_0088

Bcer98_0088

BSU00980

**BAS5102**

BCA_5390

BACI_c52440

BCZK4947

BAA_5520

BAMEG_5539

BCAH820_5342

BT9727_4932

GBAA5493

BAS5102

BA5493

BCE_5370

BCQ_5081

BCAH187_A5424

BCG9842_B5580

BCB4264_A5376

BC5251

BMB171_C4847

BcerKBAB4_5045

Bcer98_3783

**SigA**

BCA_4403

BA4515

BAS4194

BT9727_4032

BCZK4042

BALH_3885

BAA_4536

BCAH820_4314

GBAA4515

BAMEG_4554

BCG9842_B0827

BcerKBAB4_4144

BMB171_C3957

BC4289

BCB4264_A4409

BCAH187_A4426

BCE_4372

BCQ_4079

BACI_c42620

Bcer98_3019

BSU25200

**BMB171_P0077**

pE33L466_0212

BMB171_P0077

**SigE**

BCB4264_A4005

BALH_3534

BCA_4008

BMB171_C3568

BAMEG_0584

BCG9842_B1236

BC3904

BCE_3949

BCZK3663

BAA_4069

BAS3755

BCQ_3690

BA4043

BT9727_3646

GBAA4043

BCAH187_A3957

BCAH820_3918

BACI_c38600

BcerKBAB4_3731

Bcer98_2553

BSU15320

**SigK**

BCAH820_4418

BMB171_C4002

Bcer98_3065

BALH_3926

BCE_4421

BACI_c43070

BCQ_4125

BC4336

GBAA4566

BAS4236

BCB4264_A4459

BCG9842_B0778

BcerKBAB4_4190

BCA_4450

BCAH187_A4472

BCZK4084

BAMEG_4602

BAA_4584

BA4566

BT9727_4074

**BAS3823**

BAS3823

BAMEG_0515

BAA_4139

GBAA4115

BA4115

**SigB**

BACI_c10250

BALH_0889

BCAH820_1068

BT9727_0913

BCA_1029

BA0992

BAMEG_3579

BAA_1086

BAS0928

GBAA0992

BCZK0896

BCG9842_B4270

BcerKBAB4_0903

BCB4264_A1033

BC1004

BMB171_C0879

BCE_1086

BCAH187_A1159

BCQ_1068

BSU04730

**SigF**

BCE_4142

BAS3983

BCAH820_4094

BCZK3829

BAMEG_4334

BC4072

BALH_3690

BA4294

BCB4264_A4182

BMB171_C3737

BCQ_3863

BACI_c40400

BCA_4185

BAA_4316

BCG9842_B1056

GBAA4294

BCAH187_A4205

BT9727_3813

BcerKBAB4_3903

Bcer98_2771

BSU23450

**SigG**

BSU15330

Bcer98_2552

BCG9842_B1237

BMB171_C3567

BC3903

BcerKBAB4_3730

BCE_3948

BACI_c38590

BAS3754

BCQ_3689

BALH_3533

BCZK3662

BT9727_3645

BAMEG_0585

BCAH187_A3955

BCAH820_3917

GBAA4042

BCA_4007

BAA_4068

BCB4264_A4004

BA4042

**BAS2545**

GBAA2732

BAS2545

BA2732

BCA_2814

BT9727_2501

BAA_2796

BAMEG_1863

BALH_2454

BCAH820_2740

BACI_c26970

BCQ_2581

BCZK2466

**BcerKBAB4_3133**

Bcer98_3939

BMB171_C0869

BcerKBAB4_3133

BCB4264_A1023

BCG9842_B4279

**BAS3522**

BAA_3825

BA3803

BAS3522

BAMEG_0833

GBAA3803

BCZK3438

BcerKBAB4_3444

pE33L466_0103

BCQ_PT45

BSU12560

**Solo_genes**

BSU14730

Bcer98_3970

BCAH820_1326

BCAH187_A3458

BT9727_0859

BCE_1118

BCG9842_0035

Bcer98_2607

SigD - BSU16470

SigV - BSU27120

SigW - BSU01730

SigX - BSU23100

SigY - BSU38700

SigZ - BSU26840
