# Supplementary material for: Whole-genome phylogenies of the family Bacillaceae and expansion of the sigma factor gene family in the Bacillus cereus species-group
Source: BMC Genomics. 2011 Aug 24;12:430. doi: 10.1186/1471-2164-12-430 (PMC3171730; doi:10.1186/1471-2164-12-430)
Supplement: Additional file 3 — Results of MEME analysis of the sigma factor genes identified in iterative BLAST searches. MEME results for 10 motifs (nmotifs = 10) are shown, 7 of which follow phylogenetic patterns that differentiate PA from ECF sigma factors (Tables 2 and 3). [file 1471-2164-12-430-S3.HTML]

 
 
 
 
 MEME 
 
  TD.invisible { color: '#D5F0FF'; }
  TD.c0 { background: aqua; color: black; }
  TD.cw0 { background: aqua; color: black; font: 50% sans-serif; }
  TD.c1 { background: blue; color: white; }
  TD.cw1 { background: blue; color: white; font: 50% sans-serif; }
  TD.c2 { background: red; color: white; }
  TD.cw2 { background: red; color: white; font: 50% sans-serif; }
  TD.c3 { background: fuchsia; color: black; }
  TD.cw3 { background: fuchsia; color: black; font: 50% sans-serif; }
  TD.c4 { background: yellow; color: black; }
  TD.cw4 { background: yellow; color: black; font: 50% sans-serif; }
  TD.c5 { background: lime; color: black; }
  TD.cw5 { background: lime; color: black; font: 50% sans-serif; }
  TD.c6 { background: teal; color: white; }
  TD.cw6 { background: teal; color: white; font: 50% sans-serif; }
  TD.c7 { background: #444444; color: white; }
  TD.cw7 { background: #444444; color: white; font: 50% sans-serif; }
  TD.c8 { background: green; color: white; }
  TD.cw8 { background: green; color: white; font: 50% sans-serif; }
  TD.c9 { background: silver; color: black; }
  TD.cw9 { background: silver; color: black; font: 50% sans-serif; }
  TD.c10 { background: purple; color: white; }
  TD.cw10 { background: purple; color: white; font: 50% sans-serif; }
  TD.c11 { background: olive; color: black; }
  TD.cw11 { background: olive; color: black; font: 50% sans-serif; }
  TD.c12 { background: navy; color: white; }
  TD.cw12 { background: navy; color: white; font: 50% sans-serif; }
  TD.c13 { background: maroon; color: white; }
  TD.cw13 { background: maroon; color: white; font: 50% sans-serif; }
  TD.c14 { background: black; color: white; }
  TD.cw14 { background: black; color: white; font: 50% sans-serif; }
  TD.c15 { background: white; color: black; }
  TD.cw15 { background: white; color: black; font: 50% sans-serif; }
  B.red { color: red; }
  TD.red { color: red; }
  TH.red { color: red; }
  B.blue { color: blue; }
  TD.blue { color: blue; }
  TH.blue { color: blue; }
  B.orange { color: orange; }
  TD.orange { color: orange; }
  TH.orange { color: orange; }
  B.green { color: green; }
  TD.green { color: green; }
  TH.green { color: green; }
  B.black { color: black; }
  TD.black { color: black; }
  TH.black { color: black; }
  B.magenta { color: magenta; }
  TD.magenta { color: magenta; }
  TH.magenta { color: magenta; }
  B.pink { color: pink; }
  TD.pink { color: pink; }
  TH.pink { color: pink; }
  B.yellow { color: yellow; }
  TD.yellow { color: yellow; }
  TH.yellow { color: yellow; }
  B.turquoise { color: turquoise; }
  TD.turquoise { color: turquoise; }
  TH.turquoise { color: turquoise; }
 
 
 
   
  
     Command line  
     Training Set  
     First Motif  
     Summary of Motifs  
     Termination  
     Explanation   
 

 
 
   Search sequence databases with these motifs using  MAST. 
   Submit these motifs to  BLOCKS multiple alignment processor. 
   Build and use a motif-based hidden Markov model (HMM) using  Meta-MEME. 
  
    
      MEME - Motif discovery tool  
    
 
MEME version 3.5.7 (Release date: 2007-12-17 16:56:19 -0800 (Mon, 17 Dec 2007))
 
For further information on how to interpret these results or to get
a copy of the MEME software please access  http://meme.nbcr.net. 
 
This file may be used as input to the MAST algorithm for searching
sequence databases for matches to groups of motifs.  MAST is available
for interactive use and downloading at  http://meme.nbcr.net. 

    
      REFERENCE  
    
 
If you use this program in your research, please cite:
 
Timothy L. Bailey and Charles Elkan,
"Fitting a mixture model by expectation maximization to discover
motifs in biopolymers", Proceedings of the Second International
Conference on Intelligent Systems for Molecular Biology, pp. 28-36,
AAAI Press, Menlo Park, California, 1994.

    
      TRAINING SET  
    
 
DATAFILE= secondpass.twoline.faa
ALPHABET= ACDEFGHIKLMNPQRSTVWY
Sequence name            Weight Length  Sequence name            Weight Length  
-------------            ------ ------  -------------            ------ ------  
gi|157691589|BPUM_0807|Y 1.0000    200  gi|163940708|BcerKBAB4_2 1.0000     35  
gi|239827399|GWCH70_2031 1.0000    831  gi|229916702|EAT1b_0975| 1.0000    180  
gi|229917217|EAT1b_1492| 1.0000    101  gi|56964615|ABC2851|YP_1 1.0000    220  
gi|49477012|BT9727_0859| 1.0000    199  gi|261409914|GYMC10_6143 1.0000    524  
gi|56963278|ABC1513|YP_1 1.0000    151  gi|261407639|GYMC10_3840 1.0000    582  
gi|163119564|BL05276|YP_ 1.0000     82  gi|169827149|Bsph_1579|Y 1.0000    271  
gi|15615679|BH3117|NP_24 1.0000    230  gi|52080136|BL02256|YP_0 1.0000    259  
gi|52082396|BL00940|YP_0 1.0000    177  gi|295696140|Btus_1522|Y 1.0000    242  
gi|296504786|BMB171_C395 1.0000    375  gi|154686587|RBAM_021560 1.0000    255  
gi|288555607|BpOF4_12995 1.0000    235  gi|295702333|BMD_0117|YP 1.0000    216  
gi|301056170|BACI_c46590 1.0000    181  gi|295695225|Btus_0554|Y 1.0000    209  
gi|239826640|GWCH70_1138 1.0000    254  gi|169829522|Bsph_4082|Y 1.0000    180  
gi|52783954|BLi00116|YP_ 1.0000    223  gi|138895828|GTNG_2187|Y 1.0000    181  
gi|154685119|RBAM_006640 1.0000    165  gi|15613235|BH0672|NP_24 1.0000    285  
gi|296502433|BMB171_C159 1.0000    112  gi|301055395|BACI_c38600 1.0000    238  
gi|52786504|BLi02769|YP_ 1.0000    241  gi|169828833|Bsph_3367|Y 1.0000    172  
gi|296501752|BMB171_C091 1.0000    206  gi|169828654|Bsph_3171|Y 1.0000    154  
gi|261409324|GYMC10_5549 1.0000    256  gi|56964009|ABC2244|YP_1 1.0000    254  
gi|15615785|BH3223|NP_24 1.0000    242  gi|169827019|Bsph_1443|Y 1.0000    204  
gi|295695669|Btus_1025|Y 1.0000    206  gi|15613939|BH1376|NP_24 1.0000    372  
gi|229917220|EAT1b_1495| 1.0000    186  gi|261405575|GYMC10_1726 1.0000    182  
gi|212638647|Aflv_0804|Y 1.0000    129  gi|297529299|GC56T3_0955 1.0000    245  
gi|301053373|BACI_c17850 1.0000    160  gi|296503062|BMB171_C223 1.0000    177  
gi|301053967|BACI_c23980 1.0000    289  gi|261419324|GYMC61_1900 1.0000    239  
gi|261409365|GYMC10_5592 1.0000    229  gi|67078088|pE33L466_021 1.0000    323  
gi|261409537|GYMC10_5766 1.0000    214  gi|23100228|OB2773|NP_69 1.0000    188  
gi|239826355|GWCH70_0832 1.0000    245  gi|297530579|GC56T3_2307 1.0000    251  
gi|261404502|GYMC10_0633 1.0000    176  gi|295706363|BMD_4258|YP 1.0000    239  
gi|288556053|BpOF4_15235 1.0000    176  gi|15616444|BH3882|NP_24 1.0000    154  
gi|15614178|BH1615|NP_24 1.0000    176  gi|295695629|Btus_0984|Y 1.0000    246  
gi|297530706|GC56T3_2446 1.0000    239  gi|296501706|BMB171_C086 1.0000    176  
gi|295696809|Btus_2226|Y 1.0000    195  gi|295695448|Btus_0783|Y 1.0000    379  
gi|261405990|GYMC10_2143 1.0000    251  gi|23098557|OB1102|NP_69 1.0000    242  
gi|301055076|BACI_c35350 1.0000    177  gi|169830054|Bsph_4638|Y 1.0000    215  
gi|172056123|Exig_0079|Y 1.0000    213  gi|138896056|GTNG_2419|Y 1.0000    375  
gi|56965809|ABC4051|YP_1 1.0000    179  gi|169829726|Bsph_4295|Y 1.0000    261  
gi|169826679|Bsph_1097|Y 1.0000    180  gi|52787569|BLi03891|YP_ 1.0000    201  
gi|15613848|BH1285|NP_24 1.0000    235  gi|297529579|GC56T3_1250 1.0000    181  
gi|52080250|BL01246|YP_0 1.0000    254  gi|294499031|BMQ_2272|YP 1.0000    159  
gi|229918653|EAT1b_2940| 1.0000    256  gi|16079737|BSU26840|NP_ 1.0000    176  
gi|261417590|GYMC61_0090 1.0000    238  gi|169828091|Bsph_2568|Y 1.0000    177  
gi|56961818|ABC0036|YP_1 1.0000    246  gi|294501280|BMQ_4542|YP 1.0000    377  
gi|15612678|BH0115|NP_24 1.0000    217  gi|23098724|OB1269|NP_69 1.0000    174  
gi|52785627|BLi01868|YP_ 1.0000    254  gi|261406063|GYMC10_2217 1.0000    197  
gi|297585316|Bsel_3049|Y 1.0000    175  gi|52078665|BL02699|YP_0 1.0000    187  
gi|23097558|OB0103|NP_69 1.0000    217  gi|294499631|BMQ_2875|YP 1.0000    176  
gi|297528524|GC56T3_0148 1.0000    190  gi|169827296|Bsph_1728|Y 1.0000    173  
gi|154685410|RBAM_009760 1.0000    163  gi|169826467|Bsph_0879|Y 1.0000    182  
gi|261406207|GYMC10_2363 1.0000    178  gi|23099399|OB1944|NP_69 1.0000    380  
gi|261406707|GYMC10_2870 1.0000    200  gi|301054766|BACI_c32210 1.0000    183  
gi|154684616|RBAM_001230 1.0000    218  gi|154685892|RBAM_014590 1.0000    173  
gi|301056717|BACI_c52440 1.0000    156  gi|301054926|BACI_c33840 1.0000    240  
gi|169826213|Bsph_0618|Y 1.0000    182  gi|56964116|ABC2351|YP_1 1.0000    237  
gi|296503330|BMB171_C249 1.0000    228  gi|229917493|EAT1b_1768| 1.0000    173  
gi|23099294|OB1839|NP_69 1.0000    250  gi|297582861|Bsel_0539|Y 1.0000    268  
gi|261417856|GYMC61_0374 1.0000    252  gi|295705020|BMD_2904|YP 1.0000    176  
gi|296504831|BMB171_C400 1.0000    196  gi|295695806|Btus_1170|Y 1.0000    238  
gi|295696440|Btus_1834|Y 1.0000    256  gi|301054311|BACI_c27530 1.0000    228  
gi|261405623|GYMC10_1774 1.0000    377  gi|301051831|BACI_c01200 1.0000    218  
gi|297582431|Bsel_0096|Y 1.0000    184  gi|261405539|GYMC10_1690 1.0000    233  
gi|261419325|GYMC61_1901 1.0000    259  gi|138895880|GTNG_2239|Y 1.0000    252  
gi|295706259|BMD_4154|YP 1.0000    253  gi|297582645|Bsel_0319|Y 1.0000    166  
gi|261405165|GYMC10_1312 1.0000    180  gi|23100115|OB2660|NP_69 1.0000    187  
gi|239826532|GWCH70_1030 1.0000    239  gi|52784408|BLi00595|YP_ 1.0000    165  
gi|15615117|BH2554|NP_24 1.0000    261  gi|261407370|GYMC10_3567 1.0000    183  
gi|218896653|BCG9842_B36 1.0000     87  gi|157694260|BPUM_3514|Y 1.0000    176  
gi|261406097|GYMC10_2252 1.0000    204  gi|23099454|OB1999|NP_69 1.0000    234  
gi|52081127|BL02107|YP_0 1.0000    241  gi|288554248|BpOF4_06145 1.0000    170  
gi|172058539|Exig_2532|Y 1.0000    181  gi|157691684|BPUM_0902|Y 1.0000    163  
gi|172057862|Exig_1853|Y 1.0000    257  gi|229917375|EAT1b_1650| 1.0000    213  
gi|15613183|BH0620|NP_24 1.0000    177  gi|261419010|GYMC61_1575 1.0000    193  
gi|239827600|GWCH70_2250 1.0000    250  gi|56962021|ABC0239|YP_1 1.0000    187  
gi|288556276|BpOF4_16370 1.0000    184  gi|294509114|BMQ_pBM5002 1.0000    176  
gi|294501013|BMQ_4269|YP 1.0000    259  gi|261404792|GYMC10_0928 1.0000    216  
gi|297582696|Bsel_0370|Y 1.0000    187  gi|169829980|Bsph_4562|Y 1.0000    181  
gi|157691562|BPUM_0780|Y 1.0000    188  gi|172057556|Exig_1543|Y 1.0000    175  
gi|23098930|OB1475|NP_69 1.0000    239  gi|261405674|GYMC10_1825 1.0000    260  
gi|295702393|BMD_0187|YP 1.0000    187  gi|296504397|BMB171_C356 1.0000    259  
gi|138894662|GTNG_0992|Y 1.0000    239  gi|52786447|BLi02712|YP_ 1.0000    373  
gi|297529524|GC56T3_1195 1.0000    252  gi|52080819|BL00651|YP_0 1.0000    194  
gi|297582547|Bsel_0215|Y 1.0000    185  gi|218848158|BCG9842_003 1.0000    295  
gi|261406353|GYMC10_2510 1.0000    406  gi|23100811|OB3356|NP_69 1.0000    195  
gi|157691239|BPUM_0446|Y 1.0000    262  gi|157690881|BPUM_0083|Y 1.0000    218  
gi|261404258|GYMC10_0386 1.0000    299  gi|297531625|GC56T3_3410 1.0000    256  
gi|295695216|Btus_0545|Y 1.0000    186  gi|295702435|BMD_0229|YP 1.0000    264  
gi|294498927|BMQ_2164|YP 1.0000    164  gi|261404968|GYMC10_1112 1.0000    308  
gi|154685949|RBAM_015160 1.0000    260  gi|23097683|OB0228|NP_69 1.0000    187  
gi|294509181|BMQ_pBM5009 1.0000    220  gi|295706676|BMD_4577|YP 1.0000    242  
gi|52079432|BL02851|YP_0 1.0000    163  gi|261404314|GYMC10_0444 1.0000    170  
gi|138896469|GTNG_2832|Y 1.0000    143  gi|157692422|BPUM_1642|Y 1.0000     77  
gi|157690957|BPUM_0160|Y 1.0000    195  gi|56963560|ABC1795|YP_1 1.0000    254  
gi|288555708|BpOF4_13500 1.0000    176  gi|296505675|BMB171_C484 1.0000    156  
gi|23099768|OB2313|NP_69 1.0000    155  gi|172056208|Exig_0164|Y 1.0000    181  
gi|295705661|BMD_3546|YP 1.0000    170  gi|218897389|BCG9842_B29 1.0000     37  
gi|218235233|BCB4264_A18 1.0000    112  gi|261417650|GYMC61_0150 1.0000    190  
gi|15616194|BH3632|NP_24 1.0000    320  gi|297528465|GC56T3_0089 1.0000    227  
gi|157694452|BPUM_3710|Y 1.0000    164  gi|154684695|RBAM_002260 1.0000    187  
gi|157693076|BPUM_2309|Y 1.0000    289  gi|296502728|BMB171_C189 1.0000    192  
gi|157692810|BPUM_2042|Y 1.0000    202  gi|154686555|RBAM_021240 1.0000    194  
gi|52079008|BL02208|YP_0 1.0000    263  gi|261407775|GYMC10_3981 1.0000    316  
gi|52785264|BLi01499|YP_ 1.0000    251  gi|288553151|BpOF4_00625 1.0000    237  
gi|296506459|BMB171_P007 1.0000    323  gi|52786234|BLi02495|YP_ 1.0000    255  
gi|15615778|BH3216|NP_24 1.0000    193  gi|56963737|ABC1972|YP_1 1.0000    176  
gi|52786190|BLi02449|YP_ 1.0000    194  gi|296503941|BMB171_C311 1.0000    235  
gi|172056180|Exig_0136|Y 1.0000    187  gi|52080135|BL02255|YP_0 1.0000    239  
gi|261408152|GYMC10_4361 1.0000    244  gi|172056869|Exig_0832|Y 1.0000    361  
gi|301053658|BACI_c20780 1.0000    192  gi|23099037|OB1582|NP_69 1.0000    258  
gi|172058807|Exig_2804|Y 1.0000    265  gi|288556282|BpOF4_16400 1.0000    178  
gi|229917800|EAT1b_2078| 1.0000    266  gi|163119552|BL03682|YP_ 1.0000    373  
gi|239827809|GWCH70_2471 1.0000    236  gi|52784380|BLi00560|YP_ 1.0000    264  
gi|295696452|Btus_1846|Y 1.0000    261  gi|261409476|GYMC10_5703 1.0000    188  
gi|154685948|RBAM_015150 1.0000    239  gi|301055785|BACI_c42620 1.0000    375  
gi|295706482|BMD_4377|YP 1.0000    253  gi|52785509|BLi01750|YP_ 1.0000    239  
gi|52785449|BLi01689|YP_ 1.0000    171  gi|157691378|BPUM_0588|Y 1.0000    181  
gi|56963382|ABC1617|YP_1 1.0000    235  gi|296504398|BMB171_C356 1.0000    239  
gi|294500640|BMQ_3893|YP 1.0000    166  gi|169829158|Bsph_3702|Y 1.0000    375  
gi|52082452|BL01923|YP_0 1.0000    182  gi|52785510|BLi01751|YP_ 1.0000    259  
gi|297584063|Bsel_1770|Y 1.0000    253  gi|23098931|OB1476|NP_69 1.0000    260  
gi|296502978|BMB171_C214 1.0000    289  gi|294501329|BMQ_4591|YP 1.0000    242  
gi|261418447|GYMC61_0982 1.0000    245  gi|261404205|GYMC10_0333 1.0000    162  
gi|294497070|BMQ_0235|YP 1.0000    264  gi|288555754|BpOF4_13730 1.0000    372  
gi|154686781|RBAM_023510 1.0000    373  gi|294501135|BMQ_4391|YP 1.0000    253  
gi|154687094|RBAM_026660 1.0000    171  gi|52784792|BLi01020|YP_ 1.0000    163  
gi|157693020|BPUM_2253|Y 1.0000    373  gi|15615924|BH3362|NP_24 1.0000    183  
gi|295704098|BMD_1970|YP 1.0000    188  gi|261405228|GYMC10_1376 1.0000    172  
gi|169827960|Bsph_2435|Y 1.0000    159  gi|301053341|BACI_c17530 1.0000    188  
gi|294500554|BMQ_3807|YP 1.0000    224  gi|16080921|BSU38700|NP_ 1.0000    178  
gi|296504567|BMB171_C373 1.0000    252  gi|138894925|GTNG_1263|Y 1.0000    173  
gi|288553039|BpOF4_00065 1.0000    257  gi|297582430|Bsel_0095|Y 1.0000    214  
gi|288554854|BpOF4_09205 1.0000    261  gi|23100547|OB3092|NP_69 1.0000    179  
gi|154685755|RBAM_013220 1.0000    248  gi|261406734|GYMC10_2897 1.0000    191  
gi|15615119|BH2556|NP_24 1.0000    237  gi|297582837|Bsel_0515|Y 1.0000    178  
gi|15614101|BH1538|NP_24 1.0000    252  gi|30019875|BC1731|NP_83 1.0000    112  
gi|229917320|EAT1b_1595| 1.0000    191  gi|23098703|OB1248|NP_69 1.0000    163  
gi|295704351|BMD_2228|YP 1.0000    159  gi|301055830|BACI_c43070 1.0000    181  
gi|239826131|GWCH70_0591 1.0000    186  gi|157693526|BPUM_2772|Y 1.0000    172  
gi|15612826|BH0263|NP_24 1.0000    187  gi|52078593|BL03273|YP_0 1.0000    223  
gi|288554775|BpOF4_08800 1.0000    187  gi|296500928|BMB171_C009 1.0000    219  
gi|229916320|EAT1b_0589| 1.0000    364  gi|261409306|GYMC10_5531 1.0000    185  
gi|138893768|GTNG_0089|Y 1.0000    216  gi|295694818|Btus_0134|Y 1.0000    221  
gi|56420261|GK1726|YP_14 1.0000    161  gi|294497991|BMQ_1224|YP 1.0000    236  
gi|296504096|BMB171_C326 1.0000    177  gi|261404455|GYMC10_0586 1.0000    166  
gi|239827752|GWCH70_2414 1.0000    374  gi|52785094|BLi01326|YP_ 1.0000    167  
gi|296505785|BMB171_C495 1.0000    175  gi|15613092|BH0529|NP_24 1.0000    261  
gi|52784027|BLi00199|YP_ 1.0000    187  gi|138894663|GTNG_0993|Y 1.0000    264  
gi|56963455|ABC1690|YP_1 1.0000    373  gi|56964115|ABC2350|YP_1 1.0000    261  
gi|295703274|BMD_1138|YP 1.0000    163  gi|229916805|EAT1b_1078| 1.0000    147  
gi|288555447|BpOF4_12185 1.0000    182  gi|294500913|BMQ_4167|YP 1.0000    253  
gi|294500560|BMQ_3813|YP 1.0000    216  gi|52787848|BLi04171|YP_ 1.0000    189  
gi|157692018|BPUM_1237|Y 1.0000    251  gi|295706362|BMD_4257|YP 1.0000    259  
gi|42780194|BCE_1118|NP_ 1.0000    180  gi|301052754|BACI_c11460 1.0000    161  
gi|261406105|GYMC10_2260 1.0000    163  gi|294497028|BMQ_0193|YP 1.0000    187  
gi|301055569|BACI_c40400 1.0000    252  gi|152977630|Bcer98_3970 1.0000    183  
gi|138893826|GTNG_0147|Y 1.0000    162  gi|239825735|GWCH70_0154 1.0000    183  
gi|295705913|BMD_3805|YP 1.0000    216  gi|288553150|BpOF4_00620 1.0000    261  
gi|296502401|BMB171_C156 1.0000    188  gi|52788043|BLi04371|YP_ 1.0000    285  
gi|239826533|GWCH70_1031 1.0000    259  gi|23098085|OB0630|NP_69 1.0000    263  
gi|294498777|BMQ_2014|YP 1.0000    188  gi|301052678|BACI_c10700 1.0000    177  
gi|261406442|GYMC10_2604 1.0000    193  gi|295705907|BMD_3799|YP 1.0000    221  
gi|138896107|GTNG_2470|Y 1.0000    237  gi|261408790|GYMC10_5011 1.0000    234  
gi|154685132|RBAM_006770 1.0000    177  gi|67077986|pE33L466_010 1.0000    162  
gi|138894489|GTNG_0819|Y 1.0000    246  gi|169827020|Bsph_1444|Y 1.0000    257  
gi|52080075|BL02968|YP_0 1.0000    171  gi|169827905|Bsph_2380|Y 1.0000    172  
gi|294496966|BMQ_0119|YP 1.0000    216  gi|239825676|GWCH70_0094 1.0000    216  
gi|261404562|GYMC10_0696 1.0000    172  gi|297582968|Bsel_0647|Y 1.0000    185  
gi|239827551|GWCH70_2200 1.0000    182  gi|218902444|BCAH820_132 1.0000    179  
gi|152976319|Bcer98_2607 1.0000    242  gi|52080862|BL00778|YP_0 1.0000    255  
gi|218232506|BCB4264_A54 1.0000     59  gi|56962590|ABC0816|YP_1 1.0000    261  
gi|288556517|BpOF4_17595 1.0000    163  gi|169829956|Bsph_4538|Y 1.0000    157  
gi|15614589|BH2026|NP_24 1.0000    179  gi|294500314|BMQ_3558|YP 1.0000    170  
gi|23098753|OB1298|NP_69 1.0000    175  gi|169827282|Bsph_1714|Y 1.0000    195  
gi|169829306|Bsph_3856|Y 1.0000    228  gi|288555440|BpOF4_12150 1.0000    168  
gi|15614994|BH2431|NP_24 1.0000    256  gi|157692327|BPUM_1546|Y 1.0000    255  
gi|52079036|BL05045|YP_0 1.0000    165  gi|154686064|RBAM_016310 1.0000    254  
gi|261420836|GYMC61_3488 1.0000    256  gi|294497918|BMQ_1151|YP 1.0000    163  
gi|261417657|GYMC61_0157 1.0000    375  gi|297583869|Bsel_1572|Y 1.0000    184  
gi|297529352|GC56T3_1008 1.0000    375  gi|288554708|BpOF4_08465 1.0000    215  
gi|301054038|BACI_c24720 1.0000    177  gi|297531020|GC56T3_2772 1.0000    193  
gi|138897001|GTNG_3372|Y 1.0000    258  gi|52079892|BL03529|YP_0 1.0000    251  
gi|154684976|RBAM_005070 1.0000    262  gi|157692208|BPUM_1427|Y 1.0000    259  
gi|261406319|GYMC10_2476 1.0000    263  gi|157692844|BPUM_2076|Y 1.0000    255  
gi|296501716|BMB171_C087 1.0000    257  gi|295696987|Btus_2417|Y 1.0000    188  
gi|295703345|BMD_1209|YP 1.0000    236  gi|261404422|GYMC10_0553 1.0000    180  
gi|295694956|Btus_0276|Y 1.0000    197  gi|154686834|RBAM_024040 1.0000    240  
gi|56961915|ABC0133|YP_1 1.0000    220  gi|295705993|BMD_3885|YP 1.0000    167  
gi|261405039|GYMC10_1184 1.0000    155  gi|261405673|GYMC10_1824 1.0000    240  
gi|52082178|BL04030|YP_0 1.0000    201  gi|42779274|BCE_0193|NP_ 1.0000    114  
gi|288555973|BpOF4_14835 1.0000    256  gi|301052635|BACI_c10250 1.0000    258  
gi|261409947|GYMC10_6177 1.0000    162  gi|218905476|BCAH820_436 1.0000    100  
gi|295706627|BMD_4528|YP 1.0000    377  gi|301055394|BACI_c38590 1.0000    289  
gi|52079725|BL03831|YP_0 1.0000    167  gi|295696453|Btus_1847|Y 1.0000    242  
gi|52787786|BLi04109|YP_ 1.0000    177  gi|42783741|BCE_4695|NP_ 1.0000     81  
gi|261419446|GYMC61_2030 1.0000    251  gi|297584635|Bsel_2346|Y 1.0000    371  
gi|295694973|Btus_0293|Y 1.0000    185  gi|169829988|Bsph_4570|Y 1.0000    187  
gi|297530705|GC56T3_2445 1.0000    259  gi|301056831|BACI_c53580 1.0000    175  
gi|52082644|BL00106|YP_0 1.0000    285  gi|261407955|GYMC10_4162 1.0000    262  
gi|301054256|BACI_c26970 1.0000    176  gi|261407440|GYMC10_3639 1.0000    286  
gi|297582728|Bsel_0402|Y 1.0000    181  gi|16078537|BSU14730|NP_ 1.0000    173  
gi|294500199|BMQ_3443|YP 1.0000    177  gi|157692207|BPUM_1426|Y 1.0000    239  
gi|261404261|GYMC10_0389 1.0000    177  gi|294501014|BMQ_4270|YP 1.0000    239  
gi|138894766|GTNG_1100|Y 1.0000    253  gi|261417910|GYMC61_0428 1.0000    181  
gi|261406462|GYMC10_2625 1.0000    197  gi|138894123|GTNG_0449|Y 1.0000    180  
gi|295704249|BMD_2121|YP 1.0000    164  gi|217960836|BCAH187_A34 1.0000    872  
gi|154685691|RBAM_012580 1.0000    170  gi|23097664|OB0209|NP_69 1.0000    170  
gi|56419662|GK1127|YP_14 1.0000    239  gi|49186524|BAS3522|YP_0 1.0000    167  
gi|56420843|GK2308|YP_14 1.0000    250  gi|218897485|BCG9842_B28 1.0000    177  
gi|49186821|BAS3823|YP_0 1.0000    314  gi|161611198|GBAA2970|YP 1.0000    192  
gi|255767137|BSU04730|NP 1.0000    262  gi|218901842|BCAH820_070 1.0000    185  
gi|49478442|BT9727_3646| 1.0000    239  gi|118479803|BALH_4240|Y 1.0000    181  
gi|218902920|BCAH820_180 1.0000    188  gi|49187552|BAS4558|YP_0 1.0000    181  
gi|118479123|BALH_3533|Y 1.0000    289  gi|52143630|BCZK1605|YP_ 1.0000    160  
gi|218232753|BCB4264_A21 1.0000    192  gi|163941644|BcerKBAB4_3 1.0000    239  
gi|225862624|BCA_0684|YP 1.0000    185  gi|229604251|BAA_4924|YP 1.0000    181  
gi|163938900|BcerKBAB4_0 1.0000    257  gi|229601980|BAA_3517|YP 1.0000    235  
gi|229602836|BAA_1861|YP 1.0000    160  gi|229602282|BAA_3022|YP 1.0000    192  
gi|49481307|BT9727_4032| 1.0000    373  gi|163940993|BcerKBAB4_3 1.0000    166  
gi|218234226|BCB4264_A16 1.0000    179  gi|30263538|BA3649|NP_84 1.0000    169  
gi|229603990|BAA_4316|YP 1.0000    252  gi|152976482|Bcer98_2771 1.0000    252  
gi|222095431|BCQ_1771|YP 1.0000    188  gi|163941054|BcerKBAB4_3 1.0000    176  
gi|229604408|BAA_2852|YP 1.0000    228  gi|47530932|GBAA5610|YP_ 1.0000    175  
gi|163941815|BcerKBAB4_3 1.0000    252  gi|227813430|BAMEG_0833| 1.0000    167  
gi|52141585|BCZK3662|YP_ 1.0000    289  gi|218899057|BCG9842_B12 1.0000    259  
gi|118478833|BALH_3229|Y 1.0000    177  gi|49183986|BAS0964|YP_0 1.0000    206  
gi|227817068|BAMEG_4554| 1.0000    373  gi|229601988|BAA_2511|YP 1.0000    289  
gi|118475937|BALH_0169|Y 1.0000    164  gi|49186754|BAS3755|YP_0 1.0000    239  
gi|30022159|BC4072|NP_83 1.0000    252  gi|218234749|BCB4264_A40 1.0000    239  
gi|47529862|GBAA4566|YP_ 1.0000    237  gi|225866273|BCA_4403|YP 1.0000    373  
gi|229604897|BAA_2557|YP 1.0000    177  gi|49188088|BAS5102|YP_0 1.0000    146  
gi|222094400|BCQ_0714|YP 1.0000    185  gi|52141944|BCZK3297|YP_ 1.0000    177  
gi|218234415|BCB4264_A46 1.0000    181  gi|118477262|BALH_1573|Y 1.0000    160  
gi|49479940|BT9727_1587| 1.0000    188  gi|229604778|BAA_1124|YP 1.0000    177  
gi|49187190|BAS4194|YP_0 1.0000    373  gi|218897120|BCG9842_B31 1.0000    192  
gi|118476332|BALH_0588|Y 1.0000    185  gi|217960990|BCAH187_A36 1.0000    177  
gi|218901372|BCAH820_019 1.0000    163  gi|47529811|GBAA4515|YP_ 1.0000    373  
gi|47530811|GBAA5493|YP_ 1.0000    146  gi|227815388|BAMEG_2801| 1.0000    160  
gi|118479762|BALH_4199|Y 1.0000    169  gi|52144286|BCZK0942|YP_ 1.0000    177  
gi|163938949|BcerKBAB4_0 1.0000    177  gi|217961783|BCAH187_A44 1.0000    375  
gi|163938569|BcerKBAB4_0 1.0000    185  gi|218896095|BCG9842_B42 1.0000    177  
gi|30263235|BA3324|NP_84 1.0000    166  gi|162382776|BALH_3534|Y 1.0000    239  
gi|222096033|BCQ_2373|YP 1.0000    289  gi|42780162|BCE_1086|NP_ 1.0000    257  
gi|52143656|BCZK1578|YP_ 1.0000    188  gi|52141808|BCZK3438|YP_ 1.0000    167  
gi|222098834|BCQ_5203|YP 1.0000    177  gi|218896772|BCG9842_B35 1.0000    160  
gi|30260357|BA0169|NP_84 1.0000    163  gi|52141167|BCZK4084|YP_ 1.0000    237  
gi|222094769|BCQ_1107|YP 1.0000    156  gi|229601723|BAA_5636|YP 1.0000    175  
gi|49183638|BAS0613|YP_0 1.0000    185  gi|218905890|BCAH820_477 1.0000    171  
gi|49186087|BAS3082|YP_0 1.0000    166  gi|227813739|BAMEG_1143| 1.0000    235  
gi|229604117|BAA_3359|YP 1.0000    166  gi|218897813|BCG9842_B24 1.0000    228  
gi|225866598|BCA_4732|YP 1.0000    169  gi|218903912|BCAH820_279 1.0000    228  
gi|47778165|GBAA3483|YP_ 1.0000    240  gi|49184640|BAS1626|YP_0 1.0000    188  
gi|30018830|BC0647|NP_83 1.0000    185  gi|49477092|BT9727_1012| 1.0000    161  
gi|52144214|BCZK1013|YP_ 1.0000    161  gi|49183127|BAS0093|YP_0 1.0000    218  
gi|49186981|BAS3983|YP_0 1.0000    252  gi|218898683|BCG9842_B16 1.0000    177  
gi|49183950|BAS0928|YP_0 1.0000    258  gi|52140841|BCZK4409|YP_ 1.0000    171  
gi|222096292|BCQ_2632|YP 1.0000    228  gi|222098082|BCQ_4424|YP 1.0000    169  
gi|229601998|BAA_3677|YP 1.0000    177  gi|16078710|BSU16470|NP_ 1.0000    254  
gi|47525427|GBAA0169|YP_ 1.0000    163  gi|218903588|BCAH820_247 1.0000    289  
gi|218903264|BCAH820_214 1.0000    192  gi|212639538|Aflv_1712|Y 1.0000    243  
gi|30263977|BA4115|NP_84 1.0000    299  gi|16078597|BSU15330|NP_ 1.0000    260  
gi|225864098|BCA_2202|YP 1.0000    192  gi|222093937|BCQ_0193|YP 1.0000    163  
gi|30262761|BA2789|NP_84 1.0000    228  gi|52140312|BCZK4947|YP_ 1.0000    156  
gi|218231966|BCB4264_A10 1.0000    177  gi|163942391|BcerKBAB4_4 1.0000    181  
gi|16078321|BSU12560|NP_ 1.0000    169  gi|49480184|BT9727_0913| 1.0000    258  
gi|30265385|BA5610|NP_84 1.0000    175  gi|56420789|GK2254|YP_14 1.0000    181  
gi|49186753|BAS3754|YP_0 1.0000    289  gi|222096241|BCQ_2581|YP 1.0000    176  
gi|30261806|BA1753|NP_84 1.0000    188  gi|222097523|BCQ_3863|YP 1.0000    252  
gi|30021993|BC3904|NP_83 1.0000    239  gi|49476711|BT9727_0090| 1.0000    218  
gi|218234550|BCB4264_A41 1.0000    252  gi|49477520|BT9727_1945| 1.0000    192  
gi|30023393|BC5363|NP_83 1.0000    176  gi|42784415|BCE_5370|NP_ 1.0000    156  
gi|118479464|BALH_3885|Y 1.0000    373  gi|212638699|Aflv_0856|Y 1.0000    372  
gi|49479242|BT9727_2287| 1.0000    179  gi|16077241|BSU01730|NP_ 1.0000    187  
gi|225864413|BCA_2518|YP 1.0000    289  gi|49481088|BT9727_1636| 1.0000    160  
gi|218904561|BCAH820_344 1.0000    235  gi|42784367|BCE_5322|NP_ 1.0000    190  
gi|217959667|BCAH187_A22 1.0000    192  gi|218233483|BCB4264_A24 1.0000    289  
gi|163939932|BcerKBAB4_1 1.0000    192  gi|218902955|BCAH820_183 1.0000    160  
gi|163939613|BcerKBAB4_1 1.0000    182  gi|42780290|BCE_1215|NP_ 1.0000    161  
gi|49478518|BT9727_3813| 1.0000    252  gi|217959293|BCAH187_A18 1.0000    188  
gi|227813895|BAMEG_1301| 1.0000    166  gi|217958581|BCAH187_A11 1.0000    257  
gi|217958232|BCAH187_A07 1.0000    185  gi|49186236|BAS3231|YP_0 1.0000    240  
gi|52143741|BCZK1493|YP_ 1.0000    179  gi|52140200|BCZK5060|YP_ 1.0000    177  
gi|222097349|BCQ_3689|YP 1.0000    289  gi|30261838|BA1789|NP_84 1.0000    160  
gi|163938169|BcerKBAB4_0 1.0000    163  gi|227813580|BAMEG_0984| 1.0000    177  
gi|30264150|BA4294|NP_84 1.0000    252  gi|225862220|BCA_0212|YP 1.0000    164  
gi|222094730|BCQ_1068|YP 1.0000    257  gi|225865247|BCA_3356|YP 1.0000    166  
gi|16077166|BSU00980|NP_ 1.0000    218  gi|227817453|BAMEG_4945| 1.0000    171  
gi|163941195|BcerKBAB4_3 1.0000    177  gi|30260800|BA0646|NP_84 1.0000    185  
gi|163940227|BcerKBAB4_2 1.0000    289  gi|222096073|BCQ_2413|YP 1.0000    177  
gi|218232155|BCB4264_A06 1.0000    185  gi|118480394|BALH_4859|Y 1.0000    207  
gi|163942610|BcerKBAB4_4 1.0000    176  gi|42783467|BCE_4421|NP_ 1.0000    265  
gi|217958692|BCAH187_A12 1.0000    161  gi|47778237|GBAA4042|YP_ 1.0000    259  
gi|218899234|BCG9842_B10 1.0000    252  gi|218231038|BCB4264_A01 1.0000    219  
gi|227812842|BAMEG_0200| 1.0000    163  gi|30262499|BA2502|NP_84 1.0000    177  
gi|229601447|BAA_4069|YP 1.0000    239  gi|163938101|BcerKBAB4_0 1.0000    219  
gi|225865393|BCA_3504|YP 1.0000    239  gi|42782657|BCE_3607|NP_ 1.0000    169  
gi|16079402|BSU23450|NP_ 1.0000    255  gi|30264411|BA4566|NP_84 1.0000    237  
gi|152977451|Bcer98_3783 1.0000    156  gi|218895700|BCG9842_B46 1.0000    185  
gi|30020915|BC2794|NP_83 1.0000    228  gi|229601909|BAA_0109|YP 1.0000    218  
gi|118477845|BALH_2188|Y 1.0000    289  gi|212639788|Aflv_1962|Y 1.0000    174  
gi|152975676|Bcer98_1911 1.0000    227  gi|163941043|BcerKBAB4_3 1.0000    240  
gi|225863696|BCA_1798|YP 1.0000    160  gi|152976769|Bcer98_3065 1.0000    237  
gi|49478708|BT9727_4352| 1.0000    169  gi|222097044|BCQ_3384|YP 1.0000    169  
gi|161486563|BA2970|NP_8 1.0000    192  gi|30021529|BC3426|NP_83 1.0000    239  
gi|225865885|BCA_4007|YP 1.0000    259  gi|47777889|GBAA1032|YP_ 1.0000    177  
gi|30020599|BC2469|NP_83 1.0000    179  gi|218904082|BCAH820_296 1.0000    192  
gi|30019159|BC1004|NP_83 1.0000    258  gi|49185332|BAS2323|YP_0 1.0000    179  
gi|222097350|BCQ_3690|YP 1.0000    239  gi|218897390|BCG9842_B29 1.0000    232  
gi|42781261|BCE_2195|NP_ 1.0000    192  gi|218900489|BCG9842_B54 1.0000    175  
gi|218902186|BCAH820_106 1.0000    257  gi|255767573|BSU25200|NP 1.0000    371  
gi|222095915|BCQ_2255|YP 1.0000    166  gi|30022417|BC4336|NP_83 1.0000    240  
gi|225866059|BCA_4185|YP 1.0000    252  gi|225864707|BCA_2814|YP 1.0000    176  
gi|218232533|BCB4264_A11 1.0000    161  gi|218900375|BCG9842_B55 1.0000    156  
gi|56421017|GK2482|YP_14 1.0000    375  gi|218905033|BCAH820_391 1.0000    259  
gi|30260284|BA0093|NP_84 1.0000    218  gi|47527079|GBAA1789|YP_ 1.0000    160  
gi|49186388|BAS3383|YP_0 1.0000    177  gi|163942050|BcerKBAB4_4 1.0000    375  
gi|217958624|BCAH187_A12 1.0000    177  gi|218904716|BCAH820_360 1.0000    177  
gi|163943299|BcerKBAB4_5 1.0000    194  gi|217959947|BCAH187_A25 1.0000    289  
gi|218895229|BCG9842_B52 1.0000    219  gi|217959319|BCAH187_A19 1.0000    160  
gi|118479504|BALH_3926|Y 1.0000    271  gi|49183204|BAS0171|YP_0 1.0000    163  
gi|222094839|BCQ_1177|YP 1.0000    161  gi|42780931|BCE_1861|NP_ 1.0000    160  
gi|163942935|BcerKBAB4_5 1.0000    156  gi|163940286|BcerKBAB4_2 1.0000    177  
gi|47527408|GBAA2114|YP_ 1.0000    192  gi|227816114|BAMEG_3540| 1.0000    177  
gi|118479276|BALH_3690|Y 1.0000    252  gi|218899507|BCG9842_B07 1.0000    237  
gi|49477062|BT9727_0953| 1.0000    177  gi|49477843|BT9727_2501| 1.0000    176  
gi|163940540|BcerKBAB4_2 1.0000    214  gi|217961326|BCAH187_A39 1.0000    239  
gi|227816040|BAMEG_3466| 1.0000    161  gi|217962148|BCAH187_A47 1.0000    171  
gi|30021992|BC3903|NP_83 1.0000    259  gi|49477707|BT9727_2248| 1.0000    289  
gi|47525925|GBAA0646|YP_ 1.0000    185  gi|163939015|BcerKBAB4_1 1.0000    161  
gi|52145050|BCZK0162|YP_ 1.0000    163  gi|222095751|BCQ_2091|YP 1.0000    192  
gi|49185764|BAS2758|YP_0 1.0000    192  gi|118477553|BALH_1878|Y 1.0000    201  
gi|229602177|BAA_4068|YP 1.0000    259  gi|225863070|BCA_1159|YP 1.0000    161  
gi|30020245|BC2108|NP_83 1.0000    192  gi|225862980|BCA_1069|YP 1.0000    177  
gi|52145123|BCZK0089|YP_ 1.0000    218  gi|52144667|BCZK0557|YP_ 1.0000    185  
gi|152976724|Bcer98_3019 1.0000    373  gi|42784537|BCE_5492|NP_ 1.0000    175  
gi|42783844|BCE_4798|NP_ 1.0000    181  gi|227816852|BAMEG_4334| 1.0000    252  
gi|30022370|BC4289|NP_83 1.0000    375  gi|47528023|GBAA2732|YP_ 1.0000    176  
gi|229601254|BAA_0200|YP 1.0000    163  gi|16079766|BSU27120|NP_ 1.0000    166  
gi|212637996|Aflv_0147|Y 1.0000    187  gi|222095458|BCQ_1798|YP 1.0000    160  
gi|218231149|BCB4264_A44 1.0000    237  gi|42782997|BCE_3949|NP_ 1.0000    239  
gi|163942095|BcerKBAB4_4 1.0000    237  gi|52144331|BCZK0896|YP_ 1.0000    258  
gi|49481227|BT9727_4932| 1.0000    146  gi|118476614|BALH_0889|Y 1.0000    258  
gi|47529410|GBAA4115|YP_ 1.0000    299  gi|52143032|BCZK2206|YP_ 1.0000    289  
gi|217959200|BCAH187_A17 1.0000    179  gi|47526271|GBAA0992|YP_ 1.0000    257  
gi|229603576|BAA_5520|YP 1.0000    156  gi|30261116|BA0992|NP_84 1.0000    257  
gi|47529588|GBAA4294|YP_ 1.0000    252  gi|217961324|BCAH187_A39 1.0000    259  
gi|42782494|BCE_3441|NP_ 1.0000    235  gi|225866320|BCA_4450|YP 1.0000    237  
gi|222098712|BCQ_5081|YP 1.0000    144  gi|229603088|BAA_2796|YP 1.0000    176  
gi|218233874|BCB4264_A10 1.0000    176  gi|255767497|BSU23100|NP 1.0000    194  
gi|229602244|BAA_1827|YP 1.0000    188  gi|52141583|BCZK3663|YP_ 1.0000    239  
gi|229601154|BAA_4536|YP 1.0000    373  gi|47527745|GBAA2454|YP_ 1.0000    289  
gi|56419467|GK0932|YP_14 1.0000    246  gi|217962852|BCAH187_A55 1.0000    177  
gi|227815422|BAMEG_2835| 1.0000    188  gi|218898488|BCG9842_B18 1.0000    239  
gi|217962100|BCAH187_A47 1.0000    169  gi|217961829|BCAH187_A44 1.0000    237  
gi|227817116|BAMEG_4602| 1.0000    237  gi|49478801|BT9727_4602| 1.0000    176  
gi|30262453|BA2454|NP_84 1.0000    289  gi|227812767|BAMEG_0109| 1.0000    218  
gi|42783188|BCE_4142|NP_ 1.0000    252  gi|218905848|BCAH820_473 1.0000    169  
gi|227818128|BAMEG_5654| 1.0000    175  gi|229600567|BAA_4139|YP 1.0000    314  
gi|218230792|BCB4264_A17 1.0000    188  gi|30018364|BC0114|NP_82 1.0000    219  
gi|52142720|BCZK2519|YP_ 1.0000    228  gi|30264734|BA4913|NP_84 1.0000    181  
gi|225867344|BCA_5512|YP 1.0000    175  gi|218903856|BCAH820_274 1.0000    176  
gi|217962737|BCAH187_A54 1.0000    156  gi|229601329|BAA_3825|YP 1.0000    167  
gi|218231851|BCB4264_A24 1.0000    177  gi|229604478|BAA_1086|YP 1.0000    257  
gi|30263905|BA4042|NP_84 1.0000    259  gi|227816152|BAMEG_3579| 1.0000    257  
gi|152977601|Bcer98_3939 1.0000    176  gi|212638845|Aflv_1004|Y 1.0000    247  
gi|47528609|GBAA3324|YP_ 1.0000    166  gi|218905429|BCAH820_431 1.0000    373  
gi|218899458|BCG9842_B08 1.0000    375  gi|49478441|BT9727_3645| 1.0000    289  
gi|222097738|BCQ_4079|YP 1.0000    375  gi|229602397|BAA_1198|YP 1.0000    161  
gi|30262135|BA2114|NP_84 1.0000    192  gi|42779174|BCE_0093|NP_ 1.0000    219  
gi|52141420|BCZK3829|YP_ 1.0000    252  gi|225862940|BCA_1029|YP 1.0000    257  
gi|42780207|BCE_1131|NP_ 1.0000    177  gi|225862146|BCA_0122|YP 1.0000    218  
gi|47527789|GBAA2502|YP_ 1.0000    177  gi|49476853|BT9727_0558| 1.0000    185  
gi|218902311|BCAH820_119 1.0000    161  gi|52142270|BCZK2972|YP_ 1.0000    166  
gi|212639647|Aflv_1821|Y 1.0000    239  gi|227814227|BAMEG_1635| 1.0000    192  
gi|218905533|BCAH820_441 1.0000    149  gi|227813117|BAMEG_0515| 1.0000    314  
gi|163941361|BcerKBAB4_3 1.0000    167  gi|218896046|BCG9842_B42 1.0000    176  
gi|212638503|Aflv_0658|Y 1.0000    166  gi|56419663|GK1128|YP_14 1.0000    259  
gi|152976264|Bcer98_2552 1.0000    259  gi|118477234|BALH_1540|Y 1.0000    188  
gi|218233191|BCB4264_A47 1.0000    171  gi|47528934|GBAA3649|YP_ 1.0000    169  
gi|218904418|BCAH820_330 1.0000    166  gi|218901296|BCAH820_010 1.0000    218  
gi|163941643|BcerKBAB4_3 1.0000    292  gi|52142997|BCZK2243|YP_ 1.0000    179  
gi|30263679|BA3803|NP_84 1.0000    167  gi|56419781|GK1246|YP_14 1.0000    251  
gi|30265276|BA5493|NP_84 1.0000    146  gi|227814399|BAMEG_1807| 1.0000    228  
gi|218231054|BCB4264_A34 1.0000    239  gi|30020517|BC2386|NP_83 1.0000    289  
gi|221316967|BCQ_PT45|YP 1.0000    166  gi|218900006|BCG9842_B02 1.0000    137  
gi|217961566|BCAH187_A42 1.0000    252  gi|227814737|BAMEG_2147| 1.0000    289  
gi|227813187|BAMEG_0585| 1.0000    259  gi|218903633|BCAH820_251 1.0000    177  
gi|163939640|BcerKBAB4_1 1.0000    181  gi|56421070|GK2535|YP_14 1.0000    237  
gi|118478099|BALH_2454|Y 1.0000    176  gi|225865571|BCA_3683|YP 1.0000    177  
gi|218896055|BCG9842_B42 1.0000    257  gi|30264362|BA4515|NP_84 1.0000    373  
gi|222098134|BCQ_4476|YP 1.0000    181  gi|47529336|GBAA4043|YP_ 1.0000    239  
gi|217959986|BCAH187_A25 1.0000    177  gi|225865886|BCA_4008|YP 1.0000    239  
gi|217957669|BCAH187_A01 1.0000    218  gi|52142105|BCZK3137|YP_ 1.0000    240  
gi|30021690|BC3589|NP_83 1.0000    177  gi|47525348|GBAA0093|YP_ 1.0000    218  
gi|222096892|BCQ_3232|YP 1.0000    235  gi|118478149|BALH_2506|Y 1.0000    231  
gi|49477300|BT9727_1502| 1.0000    179  gi|47530207|GBAA4913|YP_ 1.0000    181  
gi|30263906|BA4043|NP_84 1.0000    239  gi|222095341|BCQ_1681|YP 1.0000    179  
gi|218906540|BCAH820_545 1.0000    175  gi|16078017|BSU09520|NP_ 1.0000    163  
gi|49184672|BAS1658|YP_0 1.0000    160  gi|218233406|BCB4264_A40 1.0000    259  
gi|227818013|BAMEG_5539| 1.0000    156  gi|218896738|BCG9842_B35 1.0000    188  
gi|225863663|BCA_1765|YP 1.0000    188  gi|52143316|BCZK1921|YP_ 1.0000    192  
gi|222097784|BCQ_4125|YP 1.0000    263  gi|227814691|BAMEG_2101| 1.0000    177  
gi|218905035|BCAH820_391 1.0000    239  gi|118475864|BALH_0093|Y 1.0000    218  
gi|49185294|BAS2285|YP_0 1.0000    289  gi|217959830|BCAH187_A24 1.0000    166  
gi|163942349|BcerKBAB4_4 1.0000    169  gi|218232670|BCB4264_A10 1.0000    257  
gi|42783418|BCE_4372|NP_ 1.0000    375  gi|52142774|BCZK2466|YP_ 1.0000    176  
gi|42781877|BCE_2820|NP_ 1.0000    228  gi|42781595|BCE_2534|NP_ 1.0000    177  
gi|218899058|BCG9842_B12 1.0000    239  gi|49185553|BAS2545|YP_0 1.0000    176  
gi|49187232|BAS4236|YP_0 1.0000    237  gi|227814455|BAMEG_1863| 1.0000    176  
gi|225864766|BCA_2873|YP 1.0000    228  gi|42779792|BCE_0714|NP_ 1.0000    185  
gi|30023283|BC5251|NP_83 1.0000    156  gi|49188198|BAS5212|YP_0 1.0000    175  
gi|49478105|BT9727_3026| 1.0000    166  gi|152973941|Bcer98_0088 1.0000    220  
gi|152976265|Bcer98_2553 1.0000    239  gi|49481728|BT9727_3204| 1.0000    240  
gi|49185607|BAS2600|YP_0 1.0000    228  gi|47529087|GBAA3803|YP_ 1.0000    167  
gi|47526385|GBAA1113|YP_ 1.0000    161  gi|225867222|BCA_5390|YP 1.0000    156  
gi|49481469|BT9727_4074| 1.0000    237  gi|52141207|BCZK4042|YP_ 1.0000    373  
gi|227813186|BAMEG_0584| 1.0000    239  gi|218905210|BCAH820_409 1.0000    252  
gi|218896153|BCG9842_B41 1.0000    161  gi|218234396|BCB4264_A27 1.0000    228  
gi|229603531|BAA_0728|YP 1.0000    185  gi|229602619|BAA_2180|YP 1.0000    192  
gi|42782996|BCE_3948|NP_ 1.0000    289  gi|217960231|BCAH187_A28 1.0000    228  
gi|49184977|BAS1966|YP_0 1.0000    192  gi|218231535|BCB4264_A37 1.0000    177  
gi|227816484|BAMEG_3941| 1.0000    185  gi|222093864|BCQ_0107|YP 1.0000    218  
gi|49479125|BT9727_2552| 1.0000    228  gi|56421959|GK3424|YP_14 1.0000    256  
gi|118478695|BALH_3081|Y 1.0000    248  gi|163939522|BcerKBAB4_1 1.0000    181  
gi|212639646|Aflv_1820|Y 1.0000    259  gi|118477905|BALH_2250|Y 1.0000    180  
gi|49184055|BAS1035|YP_0 1.0000    161  gi|16078409|BSU13450|NP_ 1.0000    251  
gi|218906428|BCAH820_534 1.0000    156  gi|30019842|BC1698|NP_83 1.0000    188  
gi|225864482|BCA_2587|YP 1.0000    177  gi|218232261|BCB4264_A44 1.0000    375  
gi|30261223|BA1113|NP_84 1.0000    161  gi|255767354|BSU15320|NP 1.0000    239  
gi|47528082|GBAA2789|YP_ 1.0000    228  gi|212637937|Aflv_0088|Y 1.0000    223  
gi|218902228|BCAH820_111 1.0000    177  gi|30262705|BA2732|NP_84 1.0000    176  
gi|30261155|BA1032|NP_84 1.0000    177  gi|30019269|BC1114|NP_83 1.0000    161  
gi|30263387|BA3483|NP_84 1.0000    240  gi|56418624|GK0089|YP_14 1.0000    216  
gi|218233984|BCB4264_A53 1.0000    156  gi|212638144|Aflv_0295|Y 1.0000    159  
gi|118476646|BALH_0922|Y 1.0000    177  gi|49480428|BT9727_5044| 1.0000    176  
gi|217957742|BCAH187_A02 1.0000    164  gi|56418685|GK0150|YP_14 1.0000    187  
gi|42780899|BCE_1829|NP_ 1.0000    188  gi|227815066|BAMEG_2477| 1.0000    192  
gi|49479249|BT9727_3349| 1.0000    177  gi|47527044|GBAA1753|YP_ 1.0000    188  
gi|229603939|BAA_4584|YP 1.0000    237  
 
 
 
 

    
      COMMAND LINE SUMMARY  
    
 
This information can also be useful in the event you wish to report a
problem with the MEME software.

command: meme secondpass.twoline.faa -protein -mod zoops -nmotifs 10 -wg 3 -ws 1 -noendgaps -maxsize 200000 -dir /root 

model:  mod=         zoops    nmotifs=        10    evt=           inf
object function=  E-value of product of p-values
width:  minw=            8    maxw=           50    minic=        0.00
width:  wg=              3    ws=              1    endgaps=        no
nsites: minsites=        2    maxsites=      871    wnsites=       0.8
theta:  prob=            1    spmap=         pam    spfuzz=        120
em:     prior=       megap    b=          944665    maxiter=        50
        distance=    1e-05
data:   n=          188933    N=             871

sample: seed=            0    seqfrac=         1
Dirichlet mixture priors file: prior30.plib
Letter frequencies in dataset:
A 0.057 C 0.006 D 0.061 E 0.105 F 0.034 G 0.040 H 0.020 I 0.079 K 0.088 
L 0.103 M 0.028 N 0.036 P 0.022 Q 0.047 R 0.065 S 0.056 T 0.043 V 0.061 
W 0.009 Y 0.042 
Background letter frequencies (from dataset with add-one prior applied):
A 0.057 C 0.006 D 0.061 E 0.105 F 0.034 G 0.040 H 0.020 I 0.079 K 0.088 
L 0.103 M 0.028 N 0.036 P 0.022 Q 0.047 R 0.065 S 0.056 T 0.043 V 0.061 
W 0.009 Y 0.042 
 
 

    
   
     P  
     N        MOTIF  1       width = 15         sites = 707        llr = 14099        E-value = 5.0e-3215 
    
 
    Simplified  A  : : 1 1 : : 1 : : : 1 : : 4 :
    pos.-specific  C  : : : : : : : 1 : : : : : : :
    probability  D  2 9 : : : 1 : : : : : : : : :
    matrix  E  6 1 : : : 5 : : : : : : : : :
    F  : : : 1 : : : 4 : : : 1 : : :
    G  : : : : : : 5 : : 5 : : : : :
    H  : : : : : : : : : : : : : 1 :
    I  : : 3 3 : 2 : : 6 : : 2 : : 3
    K  : : : : : : : : : 2 : : 5 : :
    L  1 : 5 : : : : 1 1 : 6 2 : : 2
    M  : : : 1 : : : 1 1 : : 1 : : :
    N  : : : : : : : 1 : : : : : 1 :
    P  : : : : : : : : : : : : : : :
    Q  : : : : 8 : : : : 1 : : : : :
    R  : : : : : : : : : 1 : : 1 : :
    S  : : : : 1 : : : : : : : : 3 :
    T  : : : 1 : : 2 1 : 1 : : : : :
    V  : : 1 3 : 1 2 : 1 : 1 : : : 2
    W  : : : : : : : : : : : 1 : : :
    Y  : : : : : : : 1 1 : : 3 : : 1
  . 
               bits      7.3                
                      6.6                
                      5.9                
                      5.1                
      Information   4.4                
      content   3.7                  
    (28.8 bits) 2.9                  
                      2.2                      
                      1.5                             
                      0.7                               
                      0.0    
  . 
    Multilevel                 E      D      L      V      Q      E      G      F      I      G      L      Y      K      A      I   
    consensus                 D      I    I        T        K          S    L  
    sequence                                              V  
                                                        
                                                        

  . 
  NAME       START   P-VALUE           SITES   
  gi|212637937|Aflv_0088|Y    66  4.09e-16 
    RSYFLVGADR     E      D      I      V      Q      E      G      M      I      G      L      Y      K      A      I       RDFKEDKQSS 
  gi|294496966|BMQ_0119|YP    59  4.09e-16 
    RSYFLIGADR     E      D      I      V      Q      E      G      M      I      G      L      Y      K      A      I       RDFREDKLTS 
  gi|295705913|BMD_3805|YP    59  4.09e-16 
    RKYFLIGAEK     E      D      I      V      Q      E      G      M      I      G      L      Y      K      A      I       RDFNEDKLTS 
  gi|294500560|BMQ_3813|YP    59  4.09e-16 
    RKYFLIGAEK     E      D      I      V      Q      E      G      M      I      G      L      Y      K      A      I       RDFNEDKLTS 
  gi|295696440|Btus_1834|Y    60  4.09e-16 
    QRFLGRGYEP     E      D      L      F      Q      I      G      C      I      G      L      M      K      A      I       DKFDLSYDVK 
  gi|15612678|BH0115|NP_24    60  4.09e-16 
    RSYFLIGADH     E      D      I      V      Q      E      G      M      I      G      L      Y      K      A      I       RDFKGDKLSS 
  gi|169830054|Bsph_4638|Y    59  4.09e-16 
    RSYFLIGADK     E      D      I      V      Q      E      G      M      I      G      L      Y      K      A      I       RDFKGDKLAS 
  gi|261409537|GYMC10_5766    59  4.09e-16 
    RSYFLIGADR     E      D      I      V      Q      E      G      M      I      G      L      Y      K      A      I       RDFKGDKLSS 
  gi|295702333|BMD_0117|YP    59  4.09e-16 
    RSYFLIGADR     E      D      I      V      Q      E      G      M      I      G      L      Y      K      A      I       RDFREDKLTS 
  gi|56418624|GK0089|YP_14    59  5.52e-16 
    RSYFLVGADR     E      D      I      V      Q      E      G      M      I      G      L      Y      K      A      V       RDFKGDKLSS 
  gi|56961915|ABC0133|YP_1    63  5.52e-16 
    RSYFLIGADH     E      D      I      V      Q      E      G      M      I      G      L      Y      K      A      V       RDFNVDKLSS 
  gi|288554708|BpOF4_08465    58  5.52e-16 
    RSYFLIGADH     E      D      I      V      Q      E      G      M      I      G      L      Y      K      A      V       RDFKGDKLAS 
  gi|138893768|GTNG_0089|Y    59  5.52e-16 
    RSYFLVGADR     E      D      I      V      Q      E      G      M      I      G      L      Y      K      A      V       RDFKGDKLSS 
  gi|297528465|GC56T3_0089    70  5.52e-16 
    RSYFLVGADR     E      D      I      V      Q      E      G      M      I      G      L      Y      K      A      V       RDFKGDKLSS 
  gi|261417590|GYMC61_0090    70  5.52e-16 
    RSYFLVGADR     E      D      I      V      Q      E      G      M      I      G      L      Y      K      A      V       RDFKGDKLSS 
  gi|16077166|BSU00980|NP_    61  7.36e-16 
    RSYFLIGADR     E      D      I      V      Q      E      G      M      I      G      L      Y      K      S      I       RDFKEDKLTS 
  gi|295694818|Btus_0134|Y    61  7.36e-16 
    RSYFLIGADR     E      D      I      V      Q      E      G      M      I      G      L      Y      K      S      I       RDFRNDKLVS 
  gi|52078593|BL03273|YP_0    66  7.36e-16 
    RSYFLIGADR     E      D      I      V      Q      E      G      M      I      G      L      Y      K      S      I       RDFREDKLTS 
  gi|157690881|BPUM_0083|Y    61  7.36e-16 
    RSYFLIGADR     E      D      I      V      Q      E      G      M      I      G      L      Y      K      S      I       RDFREDKLTS 
  gi|154684616|RBAM_001230    61  7.36e-16 
    RSYFLIGADR     E      D      I      V      Q      E      G      M      I      G      L      Y      K      S      I       RDFREDKLTS 
  gi|52783954|BLi00116|YP_    66  7.36e-16 
    RSYFLIGADR     E      D      I      V      Q      E      G      M      I      G      L      Y      K      S      I       RDFREDKLTS 
  gi|212638845|Aflv_1004|Y    56  9.66e-16 
    QRFLNRGYEP     E      D      L      F      Q      I      G      C      I      G      L      M      K      S      V       DKFDLSYDVK 
  gi|23097558|OB0103|NP_69    60  9.66e-16 
    RTYFLVGADK     E      D      I      I      Q      E      G      M      I      G      L      Y      K      A      I       RDYDVDKLSS 
  gi|239825676|GWCH70_0094    59  1.27e-15 
    RSYFLVGADR     E      D      I      I      Q      E      G      M      I      G      L      Y      K      A      V       RDFKGDKLSS 
  gi|172056123|Exig_0079|Y    56  1.27e-15 
    RSYFLIGADR     E      D      I      I      Q      E      G      M      I      G      L      Y      K      A      V       RDYRTDKLAS 
  gi|222093864|BCQ_0107|YP    59  1.65e-15 
    RSYFLVGADR     E      D      I      V      Q      E      G      M      I      G      L      F      K      A      I       RDYKEDKLSS 
  gi|152973941|Bcer98_0088    59  1.65e-15 
    RSYFLVGADR     E      D      I      V      Q      E      G      M      I      G      L      F      K      A      I       RDYKEDKLSS 
  gi|118475864|BALH_0093|Y    59  1.65e-15 
    RSYFLVGADR     E      D      I      V      Q      E      G      M      I      G      L      F      K      A      I       RDYKEDKLSS 
  gi|47525348|GBAA0093|YP_    59  1.65e-15 
    RSYFLVGADR     E      D      I      V      Q      E      G      M      I      G      L      F      K      A      I       RDYKEDKLSS 
  gi|217957669|BCAH187_A01    59  1.65e-15 
    RSYFLVGADR     E      D      I      V      Q      E      G      M      I      G      L      F      K      A      I       RDYKEDKLSS 
  gi|218901296|BCAH820_010    59  1.65e-15 
    RSYFLVGADR     E      D      I      V      Q      E      G      M      I      G      L      F      K      A      I       RDYKEDKLSS 
  gi|225862146|BCA_0122|YP    59  1.65e-15 
    RSYFLVGADR     E      D      I      V      Q      E      G      M      I      G      L      F      K      A      I       RDYKEDKLSS 
  gi|42779174|BCE_0093|NP_    59  1.65e-15 
    RSYFLVGADR     E      D      I      V      Q      E      G      M      I      G      L      F      K      A      I       RDYKEDKLSS 
  gi|30018364|BC0114|NP_82    59  1.65e-15 
    RSYFLVGADR     E      D      I      V      Q      E      G      M      I      G      L      F      K      A      I       RDYKEDKLSS 
  gi|227812767|BAMEG_0109|    59  1.65e-15 
    RSYFLVGADR     E      D      I      V      Q      E      G      M      I      G      L      F      K      A      I       RDYKEDKLSS 
  gi|52145123|BCZK0089|YP_    59  1.65e-15 
    RSYFLVGADR     E      D      I      V      Q      E      G      M      I      G      L      F      K      A      I       RDYKEDKLSS 
  gi|218895229|BCG9842_B52    59  1.65e-15 
    RSYFLVGADR     E      D      I      V      Q      E      G      M      I      G      L      F      K      A      I       RDYKEDKLSS 
  gi|30260284|BA0093|NP_84    59  1.65e-15 
    RSYFLVGADR     E      D      I      V      Q      E      G      M      I      G      L      F      K      A      I       RDYKEDKLSS 
  gi|229601909|BAA_0109|YP    59  1.65e-15 
    RSYFLVGADR     E      D      I      V      Q      E      G      M      I      G      L      F      K      A      I       RDYKEDKLSS 
  gi|163938101|BcerKBAB4_0    59  1.65e-15 
    RSYFLVGADR     E      D      I      V      Q      E      G      M      I      G      L      F      K      A      I       RDYKGDKLSS 
  gi|218231038|BCB4264_A01    59  1.65e-15 
    RSYFLVGADR     E      D      I      V      Q      E      G      M      I      G      L      F      K      A      I       RDYKEDKLSS 
  gi|49476711|BT9727_0090|    59  1.65e-15 
    RSYFLVGADR     E      D      I      V      Q      E      G      M      I      G      L      F      K      A      I       RDYKEDKLSS 
  gi|49183127|BAS0093|YP_0    59  1.65e-15 
    RSYFLVGADR     E      D      I      V      Q      E      G      M      I      G      L      F      K      A      I       RDYKEDKLSS 
  gi|169827020|Bsph_1444|Y    64  1.65e-15 
    GRFAYRGEQA     D      D      L      F      Q      V      G      C      I      G      L      M      K      A      I       DHFDLKHNVR 
  gi|296500928|BMB171_C009    59  1.65e-15 
    RSYFLVGADR     E      D      I      V      Q      E      G      M      I      G      L      F      K      A      I       RDYKEDKLSS 
  gi|295696452|Btus_1846|Y    65  1.65e-15 
    QRFNNRGENV     D      D      L      F      Q      V      G      C      I      G      L      M      K      A      I       DNFDLGQNVK 
  gi|261405674|GYMC10_1825    66  1.65e-15 
    QRFNNRGEFV     D      D      L      F      Q      V      G      C      I      G      L      M      K      A      I       DNFDLSQNVK 
  gi|301051831|BACI_c01200    59  1.65e-15 
    RSYFLVGADR     E      D      I      V      Q      E      G      M      I      G      L      F      K      A      I       RDYKEDKLSS 
  gi|212639646|Aflv_1820|Y    65  2.75e-15 
    QRFNNRGEYV     D      D      L      F      Q      V      G      C      I      G      L      M      K      S      I       DNFDLSQNVK 
  gi|42782996|BCE_3948|NP_    95  2.75e-15 
    QRFNNRGEYV     D      D      L      F      Q      V      G      C      I      G      L      M      K      S      I       DNFDLGQNVK 
  gi|218233406|BCB4264_A40    65  2.75e-15 
    QRFNNRGEYV     D      D      L      F      Q      V      G      C      I      G      L      M      K      S      I       DNFDLGQNVK 
  gi|227813187|BAMEG_0585|    65  2.75e-15 
    QRFNNRGEYV     D      D      L      F      Q      V      G      C      I      G      L      M      K      S      I       DNFDLGQNVK 
  gi|163941643|BcerKBAB4_3    98  2.75e-15 
    QRFNNRGEYV     D      D      L      F      Q      V      G      C      I      G      L      M      K      S      I       DNFDLGQNVK 
  gi|152976264|Bcer98_2552    65  2.75e-15 
    QRFNNRGEFV     D      D      L      F      Q      V      G      C      I      G      L      M      K      S      I       DNFDLSQNVK 
  gi|56419663|GK1128|YP_14    65  2.75e-15 
    QRFNNRGEFV     D      D      L      F      Q      V      G      C      I      G      L      M      K      S      I       DNFDLNQNVK 
  gi|49478441|BT9727_3645|    95  2.75e-15 
    QRFNNRGEYV     D      D      L      F      Q      V      G      C      I      G      L      M      K      S      I       DNFDLGQNVK 
  gi|30263905|BA4042|NP_84    65  2.75e-15 
    QRFNNRGEYV     D      D      L      F      Q      V      G      C      I      G      L      M      K      S      I       DNFDLGQNVK 
  gi|217961324|BCAH187_A39    65  2.75e-15 
    QRFNNRGEYV     D      D      L      F      Q      V      G      C      I      G      L      M      K      S      I       DNFDLGQNVK 
  gi|229602177|BAA_4068|YP    65  2.75e-15 
    QRFNNRGEYV     D      D      L      F      Q      V      G      C      I      G      L      M      K      S      I       DNFDLGQNVK 
  gi|30021992|BC3903|NP_83    65  2.75e-15 
    QRFNNRGEYV     D      D      L      F      Q      V      G      C      I      G      L      M      K      S      I       DNFDLGQNVK 
  gi|218905033|BCAH820_391    65  2.75e-15 
    QRFNNRGEYV     D      D      L      F      Q      V      G      C      I      G      L      M      K      S      I       DNFDLGQNVK 
  gi|225865885|BCA_4007|YP    65  2.75e-15 
    QRFNNRGEYV     D      D      L      F      Q      V      G      C      I      G      L      M      K      S      I       DNFDLGQNVK 
  gi|47778237|GBAA4042|YP_    65  2.75e-15 
    QRFNNRGEYV     D      D      L      F      Q      V      G      C      I      G      L      M      K      S      I       DNFDLGQNVK 
  gi|222097349|BCQ_3689|YP    95  2.75e-15 
    QRFNNRGEYV     D      D      L      F      Q      V      G      C      I      G      L      M      K      S      I       DNFDLGQNVK 
  gi|49186753|BAS3754|YP_0    95  2.75e-15 
    QRFNNRGEYV     D      D      L      F      Q      V      G      C      I      G      L      M      K      S      I       DNFDLGQNVK 
  gi|16078597|BSU15330|NP_    66  2.75e-15 
    QRFNNRGEYV     D      D      L      F      Q      V      G      C      I      G      L      M      K      S      I       DNFDLSHNVK 
  gi|218899057|BCG9842_B12    65  2.75e-15 
    QRFNNRGEYV     D      D      L      F      Q      V      G      C      I      G      L      M      K      S      I       DNFDLGQNVK 
  gi|52141585|BCZK3662|YP_    95  2.75e-15 
    QRFNNRGEYV     D      D      L      F      Q      V      G      C      I      G      L      M      K      S      I       DNFDLGQNVK 
  gi|118479123|BALH_3533|Y    95  2.75e-15 
    QRFNNRGEYV     D      D      L      F      Q      V      G      C      I      G      L      M      K      S      I       DNFDLGQNVK 
  gi|297530705|GC56T3_2445    65  2.75e-15 
    QRFNNRGEFV     D      D      L      F      Q      V      G      C      I      G      L      M      K      S      I       DNFDLNQNVK 
  gi|301055394|BACI_c38590    95  2.75e-15 
    QRFNNRGEYV     D      D      L      F      Q      V      G      C      I      G      L      M      K      S      I       DNFDLGQNVK 
  gi|157692208|BPUM_1427|Y    66  2.75e-15 
    QRFNNRGEYV     D      D      L      F      Q      V      G      C      I      G      L      M      K      S      I       DNFDLSHNVR 
  gi|239826533|GWCH70_1031    65  2.75e-15 
    QRFNNRGEFV     D      D      L      F      Q      V      G      C      I      G      L      M      K      S      I       DNFDLNQNVK 
  gi|288553150|BpOF4_00620    65  2.75e-15 
    QRFNNRGEYV     D      D      L      F      Q      V      G      C      I      G      L      M      K      S      I       DNFDLSQNVK 
  gi|295706362|BMD_4257|YP    65  2.75e-15 
    QRFNNRGEFV     D      D      L      F      Q      V      G      C      I      G      L      M      K      S      I       DNFDLSQNVK 
  gi|56964115|ABC2350|YP_1    65  2.75e-15 
    QRFNNRGENV     D      D      L      F      Q      V      G      C      I      G      L      M      K      S      I       DNFDLSQNVK 
  gi|138894663|GTNG_0993|Y    70  2.75e-15 
    QRFNNRGEFV     D      D      L      F      Q      V      G      C      I      G      L      M      K      S      I       DNFDLNQNVK 
  gi|23098931|OB1476|NP_69    66  2.75e-15 
    QRFNNRGEYV     D      D      L      F      Q      V      G      C      I      G      L      M      K      S      I       DNFDLSHNVR 
  gi|52785510|BLi01751|YP_    66  2.75e-15 
    QRFNNRGEYV     D      D      L      F      Q      V      G      C      I      G      L      M      K      S      I       DNFDLSHNVK 
  gi|154685949|RBAM_015160    66  2.75e-15 
    QRFNNRGEYV     D      D      L      F      Q      V      G      C      I      G      L      M      K      S      I       DNFDLSHNVK 
  gi|296504397|BMB171_C356    65  2.75e-15 
    QRFNNRGEYV     D      D      L      F      Q      V      G      C      I      G      L      M      K      S      I       DNFDLGQNVK 
  gi|294501013|BMQ_4269|YP    65  2.75e-15 
    QRFNNRGEFV     D      D      L      F      Q      V      G      C      I      G      L      M      K      S      I       DNFDLSQNVK 
  gi|15615117|BH2554|NP_24    65  2.75e-15 
    QRFNNRGEYV     D      D      L      F      Q      V      G      C      I      G      L      M      K      S      I       DNFDLSQNVK 
  gi|261419325|GYMC61_1901    65  2.75e-15 
    QRFNNRGEFV     D      D      L      F      Q      V      G      C      I      G      L      M      K      S      I       DNFDLNQNVK 
  gi|52080136|BL02256|YP_0    66  2.75e-15 
    QRFNNRGEYV     D      D      L      F      Q      V      G      C      I      G      L      M      K      S      I       DNFDLSHNVK 
  gi|288555973|BpOF4_14835    62  3.52e-15 
    QRFMNRGYEA     D      D      L      F      Q      I      G      C      I      G      L      I      K      S      V       DKFDLSYDVK 
  gi|15614101|BH1538|NP_24    60  3.52e-15 
    QRFLNRGYEA     D      D      L      F      Q      I      G      C      I      G      L      I      K      S      V       DKFDLSYDVK 
  gi|56963560|ABC1795|YP_1    60  3.52e-15 
    QRFLNRGYEA     D      D      L      F      Q      I      G      C      I      G      L      I      K      S      V       DKFDLSYDVK 
  gi|218905210|BCAH820_409    60  7.20e-15 
    QRFLNRGYEP     D      D      L      F      Q      I      G      C      I      G      L      L      K      S      V       DKFDLSFDVK 
  gi|217961566|BCAH187_A42    60  7.20e-15 
    QRFLNRGYEP     D      D      L      F      Q      I      G      C      I      G      L      L      K      S      V       DKFDLSFDVK 
  gi|52141420|BCZK3829|YP_    60  7.20e-15 
    QRFLNRGYEP     D      D      L      F      Q      I      G      C      I      G      L      L      K      S      V       DKFDLSFDVK 
  gi|42783188|BCE_4142|NP_    60  7.20e-15 
    QRFLNRGYEP     D      D      L      F      Q      I      G      C      I      G      L      L      K      S      V       DKFDLSFDVK 
  gi|47529588|GBAA4294|YP_    60  7.20e-15 
    QRFLNRGYEP     D      D      L      F      Q      I      G      C      I      G      L      L      K      S      V       DKFDLSFDVK 
  gi|227816852|BAMEG_4334|    60  7.20e-15 
    QRFLNRGYEP     D      D      L      F      Q      I      G      C      I      G      L      L      K      S      V       DKFDLSFDVK 
  gi|118479276|BALH_3690|Y    60  7.20e-15 
    QRFLNRGYEP     D      D      L      F      Q      I      G      C      I      G      L      L      K      S      V       DKFDLSFDVK 
  gi|225866059|BCA_4185|YP    60  7.20e-15 
    QRFLNRGYEP     D      D      L      F      Q      I      G      C      I      G      L      L      K      S      V       DKFDLSFDVK 
  gi|16079402|BSU23450|NP_    60  7.20e-15 
    QRFLNRGYEP     D      D      L      F      Q      I      G      C      I      G      L      L      K      S      V       DKFDLTYDVR 
  gi|218899234|BCG9842_B10    60  7.20e-15 
    QRFLNRGYEP     D      D      L      F      Q      I      G      C      I      G      L      L      K      S      V       DKFDLSFDVK 
  gi|30264150|BA4294|NP_84    60  7.20e-15 
    QRFLNRGYEP     D      D      L      F      Q      I      G      C      I      G      L      L      K      S      V       DKFDLSFDVK 
  gi|49478518|BT9727_3813|    60  7.20e-15 
    QRFLNRGYEP     D      D      L      F      Q      I      G      C      I      G      L      L      K      S      V       DKFDLSFDVK 
  gi|218234550|BCB4264_A41    60  7.20e-15 
    QRFLNRGYEP     D      D      L      F      Q      I      G      C      I      G      L      L      K      S      V       DKFDLSFDVK 
  gi|222097523|BCQ_3863|YP    60  7.20e-15 
    QRFLNRGYEP     D      D      L      F      Q      I      G      C      I      G      L      L      K      S      V       DKFDLSFDVK 
  gi|49186981|BAS3983|YP_0    60  7.20e-15 
    QRFLNRGYEP     D      D      L      F      Q      I      G      C      I      G      L      L      K      S      V       DKFDLSFDVK 
  gi|30022159|BC4072|NP_83    60  7.20e-15 
    QRFLNRGYEP     D      D      L      F      Q      I      G      C      I      G      L      L      K      S      V       DKFDLSFDVK 
  gi|163941815|BcerKBAB4_3    60  7.20e-15 
    QRFLNRGYEP     D      D      L      F      Q      I      G      C      I      G      L      L      K      S      V       DKFDLSFDVK 
  gi|152976482|Bcer98_2771    60  7.20e-15 
    QRFLNRGYEP     D      D      L      F      Q      I      G      C      I      G      L      L      K      S      V       DKFDLSFDVK 
  gi|229603990|BAA_4316|YP    60  7.20e-15 
    QRFLNRGYEP     D      D      L      F      Q      I      G      C      I      G      L      L      K      S      V       DKFDLSFDVK 
  gi|56420843|GK2308|YP_14    58  7.20e-15 
    QRFLNRGYEA     D      D      L      F      Q      I      G      C      I      G      L      L      K      S      V       DKFDLSYDVK 
  gi|157692844|BPUM_2076|Y    60  7.20e-15 
    QRFLNRGYEP     D      D      L      F      Q      I      G      C      I      G      L      L      K      S      V       DKFDLSYDVK 
  gi|52080862|BL00778|YP_0    60  7.20e-15 
    QRFLNRGYEP     D      D      L      F      Q      I      G      C      I      G      L      L      K      S      V       DKFDLSYDVR 
  gi|301055569|BACI_c40400    60  7.20e-15 
    QRFLNRGYEP     D      D      L      F      Q      I      G      C      I      G      L      L      K      S      V       DKFDLSFDVK 
  gi|296504567|BMB171_C373    60  7.20e-15 
    QRFLNRGYEP     D      D      L      F      Q      I      G      C      I      G      L      L      K      S      V       DKFDLSFDVK 
  gi|294501135|BMQ_4391|YP    60  7.20e-15 
    QRFINRGYEP     D      D      L      F      Q      I      G      C      I      G      L      L      K      S      V       DKFDLSYDVK 
  gi|295706482|BMD_4377|YP    60  7.20e-15 
    QRFINRGYEP     D      D      L      F      Q      I      G      C      I      G      L      L      K      S      V       DKFDLSYDVK 
  gi|52786234|BLi02495|YP_    60  7.20e-15 
    QRFLNRGYEP     D      D      L      F      Q      I      G      C      I      G      L      L      K      S      V       DKFDLSYDVR 
  gi|297529524|GC56T3_1195    60  7.20e-15 
    QRFLNRGYEA     D      D      L      F      Q      I      G      C      I      G      L      L      K      S      V       DKFDLSYDVK 
  gi|239827600|GWCH70_2250    58  7.20e-15 
    QRFLNRGYEP     D      D      L      F      Q      I      G      C      I      G      L      L      K      S      V       DKFDLSYDVK 
  gi|138895880|GTNG_2239|Y    60  7.20e-15 
    QRFLNRGYEA     D      D      L      F      Q      I      G      C      I      G      L      L      K      S      V       DKFDLSYDVK 
  gi|261417856|GYMC61_0374    60  7.20e-15 
    QRFLNRGYEA     D      D      L      F      Q      I      G      C      I      G      L      L      K      S      V       DKFDLSYDVK 
  gi|261405990|GYMC10_2143    59  7.20e-15 
    QRFMNRGYEP     D      D      L      F      Q      I      G      C      I      G      L      L      K      S      V       DKFDLSYDVK 
  gi|154686587|RBAM_021560    60  7.20e-15 
    QRFLNRGYEP     D      D      L      F      Q      I      G      C      I      G      L      L      K      S      V       DKFDLSYDVR 
  gi|229917375|EAT1b_1650|    56  1.09e-13 
    RSYFLIGADH     E      D      I      V      Q      E      G      M      I      G      L      Y      K      G      V       RDYKDERLAS 
  gi|297582430|Bsel_0095|Y    55  2.30e-13 
    RSYFLIGADH     E      D      I      L      Q      E      G      M      I      G      L      Y      K      A      I       RDFKQGKESS 
  gi|23098085|OB0630|NP_69    58  6.54e-13 
    RKYSKNSAIH     E      D      L      V      Q      V      G      M      I      G      L      L      A      A      V       RRYDPTYGKS 
  gi|295696453|Btus_1847|Y    88  7.73e-13 
    RKFENTGIYI     E      D      L      V      S      I      G      T      I      G      L      I      K      A      V       NTFDPQKRIK 
  gi|288555708|BpOF4_13500    34  7.73e-13 
    AAFTYDQNEV     E      D      L      T      Q      E      V      F      I      R      L      F      K      S      L       SSFRGESELK 
  gi|229603939|BAA_4584|YP    76  1.07e-12 
    KKFENTGEDA     E      D      L      I      S      I      G      T      I      G      L      I      K      A      I       ESYSAGKGTK 
  gi|49481469|BT9727_4074|    76  1.07e-12 
    KKFENTGEDA     E      D      L      I      S      I      G      T      I      G      L      I      K      A      I       ESYSAGKGTK 
  gi|49187232|BAS4236|YP_0    76  1.07e-12 
    KKFENTGEDA     E      D      L      I      S      I      G      T      I      G      L      I      K      A      I       ESYSAGKGTK 
  gi|222097784|BCQ_4125|YP    102  1.07e-12 
    KKFENTGEDA     E      D      L      I      S      I      G      T      I      G      L      I      K      A      I       ESYSAGKGTK 
  gi|56421070|GK2535|YP_14    77  1.07e-12 
    KKFENTGEEV     E      D      L      I      S      I      G      T      I      G      L      I      K      A      I       ESYSPGKGTK 
  gi|218905533|BCAH820_441    76  1.07e-12 
    KKFENTGEDA     E      D      L      I      S      I      G      T      I      G      L      I      K      A      I       ESYSAGKGTK 
  gi|227817116|BAMEG_4602|    76  1.07e-12 
    KKFENTGEDA     E      D      L      I      S      I      G      T      I      G      L      I      K      A      I       ESYSAGKGTK 
  gi|217961829|BCAH187_A44    76  1.07e-12 
    KKFENTGEDA     E      D      L      I      S      I      G      T      I      G      L      I      K      A      I       ESYSAGKGTK 
  gi|225866320|BCA_4450|YP    76  1.07e-12 
    KKFENTGEDA     E      D      L      I      S      I      G      T      I      G      L      I      K      A      I       ESYSAGKGTK 
  gi|163942095|BcerKBAB4_4    76  1.07e-12 
    KKFENTGEDA     E      D      L      I      S      I      G      T      I      G      L      I      K      A      I       ESYSAGKGTK 
  gi|218231149|BCB4264_A44    76  1.07e-12 
    KKFENTGEDA     E      D      L      I      S      I      G      T      I      G      L      I      K      A      I       ESYSAGKGTK 
  gi|218899507|BCG9842_B07    76  1.07e-12 
    KKFENTGEDA     E      D      L      I      S      I      G      T      I      G      L      I      K      A      I       ESYSAGKGTK 
  gi|118479504|BALH_3926|Y    110  1.07e-12 
    KKFENTGEDA     E      D      L      I      S      I      G      T      I      G      L      I      K      A      I       ESYSAGKGTK 
  gi|30022417|BC4336|NP_83    79  1.07e-12 
    KKFENTGEDA     E      D      L      I      S      I      G      T      I      G      L      I      K      A      I       ESYSAGKGTK 
  gi|152976769|Bcer98_3065    76  1.07e-12 
    KKFENTGEDA     E      D      L      I      S      I      G      T      I      G      L      I      K      A      I       ESYSAGKGTK 
  gi|30264411|BA4566|NP_84    76  1.07e-12 
    KKFENTGEDA     E      D      L      I      S      I      G      T      I      G      L      I      K      A      I       ESYSAGKGTK 
  gi|42783467|BCE_4421|NP_    104  1.07e-12 
    KKFENTGEDA     E      D      L      I      S      I      G      T      I      G      L      I      K      A      I       ESYSAGKGTK 
  gi|52141167|BCZK4084|YP_    76  1.07e-12 
    KKFENTGEDA     E      D      L      I      S      I      G      T      I      G      L      I      K      A      I       ESYSAGKGTK 
  gi|47529862|GBAA4566|YP_    76  1.07e-12 
    KKFENTGEDA     E      D      L      I      S      I      G      T      I      G      L      I      K      A      I       ESYSAGKGTK 
  gi|138896107|GTNG_2470|Y    77  1.07e-12 
    KKFENTGEEV     E      D      L      I      S      I      G      T      I      G      L      I      K      A      I       ESYSPNKGTK 
  gi|301055830|BACI_c43070    20  1.07e-12 
    KKFENTGEDA     E      D      L      I      S      I      G      T      I      G      L      I      K      A      I       ESYSAGKGTK 
  gi|261418447|GYMC61_0982    85  1.07e-12 
    KKFENTGEEV     E      D      L      I      S      I      G      T      I      G      L      I      K      A      I       ESYSPGKGTK 
  gi|294501329|BMQ_4591|YP    77  1.07e-12 
    KKFENTGEDA     E      D      L      I      S      I      G      T      I      G      L      I      K      A      I       ESYSQGKGTK 
  gi|56963382|ABC1617|YP_1    77  1.07e-12 
    KKFENTREDV     E      D      L      I      S      I      G      T      I      G      L      I      K      A      I       ESYSEGKGTK 
  gi|239827809|GWCH70_2471    77  1.07e-12 
    KKFENTGEDA     E      D      L      I      S      I      G      T      I      G      L      I      K      A      I       ESYSPGKGTK 
  gi|157693076|BPUM_2309|Y    125  1.07e-12 
    KKFENTGEDA     E      D      L      I      S      I      G      T      I      G      L      I      K      A      I       ESYSSGKGTK 
  gi|295706676|BMD_4577|YP    77  1.07e-12 
    KKFENTGEDA     E      D      L      I      S      I      G      T      I      G      L      I      K      A      I       ESYSQGKGTK 
  gi|52081127|BL02107|YP_0    77  1.07e-12 
    KKFENTGEDA     E      D      L      I      S      I      G      T      I      G      L      I      K      A      I       ESYSAGKGTK 
  gi|261405539|GYMC10_1690    77  1.07e-12 
    KKFDNTGEDL     E      D      L      I      S      I      G      T      I      G      L      I      K      A      I       ESFRPNKGTK 
  gi|295695806|Btus_1170|Y    77  1.07e-12 
    KKFENTGEDT     E      D      L      I      S      I      G      T      I      G      L      I      K      A      I       ESFQPHKGTK 
  gi|296504831|BMB171_C400    35  1.07e-12 
    KKFENTGEDA     E      D      L      I      S      I      G      T      I      G      L      I      K      A      I       ESYSAGKGTK 
  gi|297582861|Bsel_0539|Y    64  1.07e-12 
    RKFSRGQRHD     E      D      L      V      Q      V      G      M      I      G      L      L      A      A      L       RRFDPSFGRS 
  gi|15613848|BH1285|NP_24    77  1.07e-12 
    KKFENTREHT     E      D      L      I      S      I      G      T      I      G      L      I      K      A      I       ESFSEGKGTK 
  gi|297529299|GC56T3_0955    85  1.07e-12 
    KKFENTGEEV     E      D      L      I      S      I      G      T      I      G      L      I      K      A      I       ESYSPGKGTK 
  gi|212638647|Aflv_0804|Y    77  1.07e-12 
    KKFENTGEDP     E      D      L      I      S      I      G      T      I      G      L      I      K      A      I       ESYSTGKGTK 
  gi|52786504|BLi02769|YP_    77  1.07e-12 
    KKFENTGEDA     E      D      L      I      S      I      G      T      I      G      L      I      K      A      I       ESYSAGKGTK 
  gi|288555607|BpOF4_12995    77  1.07e-12 
    KKFENTREDT     E      D      L      I      S      I      G      T      I      G      L      I      K      A      I       ESYSDGKGTK 
  gi|255767354|BSU15320|NP    85  1.26e-12 
    RKFENTGINI     E      D      L      I      S      I      G      T      I      G      L      I      K      A      V       NTFNPEKKIK 
  gi|227813186|BAMEG_0584|    85  1.26e-12 
    RKFENTGINI     E      D      L      I      S      I      G      T      I      G      L      I      K      A      V       NTFNPEKKIK 
  gi|152976265|Bcer98_2553    85  1.26e-12 
    RKFENTGINI     E      D      L      I      S      I      G      T      I      G      L      I      K      A      V       NTFNPEKKIK 
  gi|218899058|BCG9842_B12    85  1.26e-12 
    RKFENTGINI     E      D      L      I      S      I      G      T      I      G      L      I      K      A      V       NTFNPEKKIK 
  gi|218905035|BCAH820_391    85  1.26e-12 
    RKFENTGINI     E      D      L      I      S      I      G      T      I      G      L      I      K      A      V       NTFNPEKKIK 
  gi|30263906|BA4043|NP_84    85  1.26e-12 
    RKFENTGINI     E      D      L      I      S      I      G      T      I      G      L      I      K      A      V       NTFNPEKKIK 
  gi|225865886|BCA_4008|YP    85  1.26e-12 
    RKFENTGINI     E      D      L      I      S      I      G      T      I      G      L      I      K      A      V       NTFNPEKKIK 
  gi|47529336|GBAA4043|YP_    85  1.26e-12 
    RKFENTGINI     E      D      L      I      S      I      G      T      I      G      L      I      K      A      V       NTFNPEKKIK 
  gi|212639647|Aflv_1821|Y    85  1.26e-12 
    RKFENTGINI     E      D      L      I      S      I      G      T      I      G      L      I      K      A      V       NTFNPEKKIK 
  gi|52141583|BCZK3663|YP_    85  1.26e-12 
    RKFENTGINI     E      D      L      I      S      I      G      T      I      G      L      I      K      A      V       NTFNPEKKIK 
  gi|42782997|BCE_3949|NP_    85  1.26e-12 
    RKFENTGINI     E      D      L      I      S      I      G      T      I      G      L      I      K      A      V       NTFNPEKKIK 
  gi|217961326|BCAH187_A39    85  1.26e-12 
    RKFENTGINI     E      D      L      I      S      I      G      T      I      G      L      I      K      A      V       NTFNPEKKIK 
  gi|222097350|BCQ_3690|YP    85  1.26e-12 
    RKFENTGINI     E      D      L      I      S      I      G      T      I      G      L      I      K      A      V       NTFNPEKKIK 
  gi|229601447|BAA_4069|YP    85  1.26e-12 
    RKFENTGINI     E      D      L      I      S      I      G      T      I      G      L      I      K      A      V       NTFNPEKKIK 
  gi|30021993|BC3904|NP_83    85  1.26e-12 
    RKFENTGINI     E      D      L      I      S      I      G      T      I      G      L      I      K      A      V       NTFNPEKKIK 
  gi|162382776|BALH_3534|Y    85  1.26e-12 
    RKFENTGINI     E      D      L      I      S      I      G      T      I      G      L      I      K      A      V       NTFNPEKKIK 
  gi|218234749|BCB4264_A40    85  1.26e-12 
    RKFENTGINI     E      D      L      I      S      I      G      T      I      G      L      I      K      A      V       NTFNPEKKIK 
  gi|49186754|BAS3755|YP_0    85  1.26e-12 
    RKFENTGINI     E      D      L      I      S      I      G      T      I      G      L      I      K      A      V       NTFNPEKKIK 
  gi|163941644|BcerKBAB4_3    85  1.26e-12 
    RKFENTGINI     E      D      L      I      S      I      G      T      I      G      L      I      K      A      V       NTFNPEKKIK 
  gi|49478442|BT9727_3646|    85  1.26e-12 
    RKFENTGINI     E      D      L      I      S      I      G      T      I      G      L      I      K      A      V       NTFNPEKKIK 
  gi|56419662|GK1127|YP_14    85  1.26e-12 
    RKFENTGIHI     E      D      L      I      S      I      G      T      I      G      L      I      K      A      V       NTFNPEKKIK 
  gi|294501014|BMQ_4270|YP    85  1.26e-12 
    RKFENTGINI     E      D      L      I      S      I      G      T      I      G      L      I      K      A      V       NTFNPEKKIK 
  gi|157692207|BPUM_1426|Y    85  1.26e-12 
    RKFENTGINI     E      D      L      I      S      I      G      T      I      G      L      I      K      A      V       NTFNPEKKIK 
  gi|15615119|BH2556|NP_24    83  1.26e-12 
    RKFENTGINI     E      D      L      I      S      I      G      T      I      G      L      I      K      A      V       NTFNPEKKIK 
  gi|296504398|BMB171_C356    85  1.26e-12 
    RKFENTGINI     E      D      L      I      S      I      G      T      I      G      L      I      K      A      V       NTFNPEKKIK 
  gi|52785509|BLi01750|YP_    85  1.26e-12 
    RKFENTGINI     E      D      L      I      S      I      G      T      I      G      L      I      K      A      V       NTFNPEKKIK 
  gi|154685948|RBAM_015150    85  1.26e-12 
    RKFENTGINI     E      D      L      I      S      I      G      T      I      G      L      I      K      A      V       NTFNPEKKIK 
  gi|52080135|BL02255|YP_0    85  1.26e-12 
    RKFENTGINI     E      D      L      I      S      I      G      T      I      G      L      I      K      A      V       NTFNPEKKIK 
  gi|288553151|BpOF4_00625    83  1.26e-12 
    RKFENTGINI     E      D      L      I      S      I      G      T      I      G      L      I      K      A      V       NTFNPEKKIK 
  gi|138894662|GTNG_0992|Y    85  1.26e-12 
    RKFENTGIHI     E      D      L      I      S      I      G      T      I      G      L      I      K      A      V       NTFNPEKKIK 
  gi|23098930|OB1475|NP_69    85  1.26e-12 
    RKFENTGINI     E      D      L      I      S      I      G      T      I      G      L      I      K      A      V       NTFNPEKKIK 
  gi|239826532|GWCH70_1030    85  1.26e-12 
    RKFENTGINI     E      D      L      I      S      I      G      T      I      G      L      I      K      A      V       NTFNPEKKIK 
  gi|56964116|ABC2351|YP_1    83  1.26e-12 
    RKFENTGINI     E      D      L      I      S      I      G      T      I      G      L      I      K      A      V       NTFNPEKKIK 
  gi|297530706|GC56T3_2446    85  1.26e-12 
    RKFENTGIHI     E      D      L      I      S      I      G      T      I      G      L      I      K      A      V       NTFNPEKKIK 
  gi|295706363|BMD_4258|YP    85  1.26e-12 
    RKFENTGINI     E      D      L      I      S      I      G      T      I      G      L      I      K      A      V       NTFNPEKKIK 
  gi|261419324|GYMC61_1900    85  1.26e-12 
    RKFENTGIHI     E      D      L      I      S      I      G      T      I      G      L      I      K      A      V       NTFNPEKKIK 
  gi|301055395|BACI_c38600    84  1.26e-12 
    RKFENTGINI     E      D      L      I      S      I      G      T      I      G      L      I      K      A      V       NTFNPEKKIK 
  gi|229917800|EAT1b_2078|    58  2.03e-12 
    RKFSRGRAIH     D      D      L      V      Q      V      G      M      I      G      L      L      A      A      L       RRFDPEFGRS 
  gi|172058807|Exig_2804|Y    58  2.03e-12 
    RKFSRGRPIH     D      D      L      V      Q      V      G      M      I      G      L      L      A      A      L       RRFDKEFGRS 
  gi|227816484|BAMEG_3941|    37  3.22e-12 
    YSILNNIQQA     E      D      A      V      Q      E      T      F      I      T      L      Y      K      N      L       EKLHNLNIQE 
  gi|229603531|BAA_0728|YP    37  3.22e-12 
    YSILNNIQQA     E      D      A      V      Q      E      T      F      I      T      L      Y      K      N      L       EKLHNLNIQE 
  gi|42779792|BCE_0714|NP_    37  3.22e-12 
    YSILNNIQQA     E      D      A      V      Q      E      T      F      I      T      L      Y      K      N      L       AKLYSLSTEE 
  gi|52144667|BCZK0557|YP_    37  3.22e-12 
    YSILNNIQQA     E      D      A      V      Q      E      T      F      I      T      L      Y      K      N      L       EKLHSLATEE 
  gi|47525925|GBAA0646|YP_    37  3.22e-12 
    YSILNNIQQA     E      D      A      V      Q      E      T      F      I      T      L      Y      K      N      L       EKLHNLNIQE 
  gi|255767573|BSU25200|NP    161  3.22e-12 
    KRYVGRGMLF     L      D      L      I      Q      E      G      N      M      G      L      M      K      A      V       EKFDYRKGYK 
  gi|218895700|BCG9842_B46    37  3.22e-12 
    YSILNNIQQA     E      D      A      V      Q      E      T      F      I      T      L      Y      K      N      L       EKLHSLSTEE 
  gi|218232155|BCB4264_A06    37  3.22e-12 
    YSILNNIQQA     E      D      A      V      Q      E      T      F      I      T      L      Y      K      N      L       EKLHSLATQE 
  gi|30260800|BA0646|NP_84    37  3.22e-12 
    YSILNNIQQA     E      D      A      V      Q      E      T      F      I      T      L      Y      K      N      L       EKLHNLNIQE 
  gi|30018830|BC0647|NP_83    37  3.22e-12 
    YSILNNIQQA     E      D      A      V      Q      E      T      F      I      T      L      Y      K      N      L       EKLHSLSTEE 
  gi|49183638|BAS0613|YP_0    37  3.22e-12 
    YSILNNIQQA     E      D      A      V      Q      E      T      F      I      T      L      Y      K      N      L       EKLHNLNIQE 
  gi|163938569|BcerKBAB4_0    37  3.22e-12 
    YSILNNIQQA     E      D      A      V      Q      E      T      F      I      T      L      Y      K      N      L       KKLHSLNTEE 
  gi|118476332|BALH_0588|Y    37  3.22e-12 
    YSILNNIQQA     E      D      A      V      Q      E      T      F      I      T      L      Y      K      N      L       EKLHNLNIQE 
  gi|222094400|BCQ_0714|YP    37  3.22e-12 
    YSILNNIQQA     E      D      A      V      Q      E      T      F      I      T      L      Y      K      N      L       EKLHSLSTEE 
  gi|225862624|BCA_0684|YP    37  3.22e-12 
    YSILNNIQQA     E      D      A      V      Q      E      T      F      I      T      L      Y      K      N      L       EKLHNLNIQE 
  gi|218901842|BCAH820_070    37  3.22e-12 
    YSILNNIQQA     E      D      A      V      Q      E      T      F      I      T      L      Y      K      N      L       EKLHSLATQE 
  gi|157693020|BPUM_2253|Y    163  3.22e-12 
    KRYVGRGMLF     L      D      L      I      Q      E      G      N      M      G      L      M      K      A      V       EKFDYRKGYK 
  gi|154686781|RBAM_023510    163  3.22e-12 
    KRYVGRGMLF     L      D      L      I      Q      E      G      N      M      G      L      M      K      A      V       EKFDYRKGYK 
  gi|163119552|BL03682|YP_    163  3.22e-12 
    KRYVGRGMLF     L      D      L      I      Q      E      G      N      M      G      L      M      K      A      V       EKFDYRKGYK 
  gi|52786447|BLi02712|YP_    163  3.22e-12 
    KRYVGRGMLF     L      D      L      I      Q      E      G      N      M      G      L      M      K      A      V       EKFDYRKGYK 
  gi|218232261|BCB4264_A44    165  3.74e-12 
    KRYVGRGMLF     L      D      L      I      Q      E      G      N      M      G      L      I      K      A      V       EKFDYRKGFK 
  gi|52141207|BCZK4042|YP_    163  3.74e-12 
    KRYVGRGMLF     L      D      L      I      Q      E      G      N      M      G      L      I      K      A      V       EKFDYRKGFK 
  gi|42783418|BCE_4372|NP_    165  3.74e-12 
    KRYVGRGMLF     L      D      L      I      Q      E      G      N      M      G      L      I      K      A      V       EKFDYRKGFK 
  gi|30264362|BA4515|NP_84    163  3.74e-12 
    KRYVGRGMLF     L      D      L      I      Q      E      G      N      M      G      L      I      K      A      V       EKFDYRKGFK 
  gi|218899458|BCG9842_B08    165  3.74e-12 
    KRYVGRGMLF     L      D      L      I      Q      E      G      N      M      G      L      I      K      A      V       EKFDYRKGFK 
  gi|218905429|BCAH820_431    163  3.74e-12 
    KRYVGRGMLF     L      D      L      I      Q      E      G      N      M      G      L      I      K      A      V       EKFDYRKGFK 
  gi|229601154|BAA_4536|YP    163  3.74e-12 
    KRYVGRGMLF     L      D      L      I      Q      E      G      N      M      G      L      I      K      A      V       EKFDYRKGFK 
  gi|30022370|BC4289|NP_83    165  3.74e-12 
    KRYVGRGMLF     L      D      L      I      Q      E      G      N      M      G      L      I      K      A      V       EKFDYRKGFK 
  gi|152976724|Bcer98_3019    163  3.74e-12 
    KRYVGRGMLF     L      D      L      I      Q      E      G      N      M      G      L      I      K      A      V       EKFDYRKGFK 
  gi|163942050|BcerKBAB4_4    165  3.74e-12 
    KRYVGRGMLF     L      D      L      I      Q      E      G      N      M      G      L      I      K      A      V       EKFDYRKGFK 
  gi|56421017|GK2482|YP_14    165  3.74e-12 
    KRYVGRGMLF     L      D      L      I      Q      E      G      N      M      G      L      I      K      A      V       EKFDYRKGYK 
  gi|212638699|Aflv_0856|Y    162  3.74e-12 
    KRYVGRGMLF     L      D      L      I      Q      E      G      N      M      G      L      I      K      A      V       EKFDYRKGYK 
  gi|118479464|BALH_3885|Y    163  3.74e-12 
    KRYVGRGMLF     L      D      L      I      Q      E      G      N      M      G      L      I      K      A      V       EKFDYRKGFK 
  gi|217961783|BCAH187_A44    165  3.74e-12 
    KRYVGRGMLF     L      D      L      I      Q      E      G      N      M      G      L      I      K      A      V       EKFDYRKGFK 
  gi|47529811|GBAA4515|YP_    163  3.74e-12 
    KRYVGRGMLF     L      D      L      I      Q      E      G      N      M      G      L      I      K      A      V       EKFDYRKGFK 
  gi|49187190|BAS4194|YP_0    163  3.74e-12 
    KRYVGRGMLF     L      D      L      I      Q      E      G      N      M      G      L      I      K      A      V       EKFDYRKGFK 
  gi|225866273|BCA_4403|YP    163  3.74e-12 
    KRYVGRGMLF     L      D      L      I      Q      E      G      N      M      G      L      I      K      A      V       EKFDYRKGFK 
  gi|227817068|BAMEG_4554|    163  3.74e-12 
    KRYVGRGMLF     L      D      L      I      Q      E      G      N      M      G      L      I      K      A      V       EKFDYRKGFK 
  gi|49481307|BT9727_4032|    163  3.74e-12 
    KRYVGRGMLF     L      D      L      I      Q      E      G      N      M      G      L      I      K      A      V       EKFDYRKGFK 
  gi|297584635|Bsel_2346|Y    161  3.74e-12 
    KRYVGRGMLF     L      D      L      I      Q      E      G      N      M      G      L      I      K      A      V       EKFDYNKGFK 
  gi|295706627|BMD_4528|YP    167  3.74e-12 
    KRYVGRGMLF     L      D      L      I      Q      E      G      N      M      G      L      I      K      A      V       EKFDYRKGYK 
  gi|297529352|GC56T3_1008    165  3.74e-12 
    KRYVGRGMLF     L      D      L      I      Q      E      G      N      M      G      L      I      K      A      V       EKFDYRKGYK 
  gi|261417657|GYMC61_0157    165  3.74e-12 
    KRYVGRGMLF     L      D      L      I      Q      E      G      N      M      G      L      I      K      A      V       EKFDYRKGYK 
  gi|56963455|ABC1690|YP_1    163  3.74e-12 
    KRYVGRGMLF     L      D      L      I      Q      E      G      N      M      G      L      I      K      A      V       EKFDYDKGFK 
  gi|239827752|GWCH70_2414    164  3.74e-12 
    KRYVGRGMLF     L      D      L      I      Q      E      G      N      M      G      L      I      K      A      V       EKFDYRKGYK 
  gi|229916320|EAT1b_0589|    154  3.74e-12 
    KRYVGRGMLF     L      D      L      I      Q      E      G      N      M      G      L      I      K      A      V       EKFDYMKGFK 
  gi|288555754|BpOF4_13730    162  3.74e-12 
    KRYVGRGMLF     L      D      L      I      Q      E      G      N      M      G      L      I      K      A      V       EKFDYEKGYK 
  gi|169829158|Bsph_3702|Y    165  3.74e-12 
    KRYVGRGMLF     L      D      L      I      Q      E      G      N      M      G      L      I      K      A      V       EKFDYRKGFK 
  gi|301055785|BACI_c42620    165  3.74e-12 
    KRYVGRGMLF     L      D      L      I      Q      E      G      N      M      G      L      I      K      A      V       EKFDYRKGFK 
  gi|172056869|Exig_0832|Y    151  3.74e-12 
    KRYVGRGMLF     L      D      L      I      Q      E      G      N      M      G      L      I      K      A      V       EKFDYTKGYK 
  gi|261407775|GYMC10_3981    106  3.74e-12 
    RRYAGRGMQF     L      D      L      I      Q      E      G      N      M      G      L      I      K      A      V       EKFDYSKGFK 
  gi|261405623|GYMC10_1774    167  3.74e-12 
    KRYVGRGMLF     L      D      L      I      Q      E      G      N      M      G      L      I      K      A      V       EKFDYKKGFK 
  gi|23099399|OB1944|NP_69    170  3.74e-12 
    KRYVGRGMLF     L      D      L      I      Q      E      G      N      M      G      L      I      K      A      V       EKFDYRKGFK 
  gi|294501280|BMQ_4542|YP    167  3.74e-12 
    KRYVGRGMLF     L      D      L      I      Q      E      G      N      M      G      L      I      K      A      V       EKFDYRKGYK 
  gi|138896056|GTNG_2419|Y    165  3.74e-12 
    KRYVGRGMLF     L      D      L      I      Q      E      G      N      M      G      L      I      K      A      V       EKFDYRKGYK 
  gi|15613939|BH1376|NP_24    162  3.74e-12 
    KRYVGRGMLF     L      D      L      I      Q      E      G      N      M      G      L      I      K      A      V       EKFDYNKGFK 
  gi|296504786|BMB171_C395    165  3.74e-12 
    KRYVGRGMLF     L      D      L      I      Q      E      G      N      M      G      L      I      K      A      V       EKFDYRKGFK 
  gi|297582645|Bsel_0319|Y    29  5.03e-12 
    LVYYTRRDDV     E      D      L      V      Q      E      T      F      I      R      A      L      R      S      I       DGFEGRSAAK 
  gi|294500199|BMQ_3443|YP    44  6.72e-12 
    YSYVNSVAVA     E      D      L      T      Q      E      I      F      I      K      C      Y      K      N      L       YRYSGKSKFR 
  gi|294497070|BMQ_0235|YP    57  7.75e-12 
    RRYSKGRSLH     E      D      I      V      Q      V      G      M      L      G      L      L      G      A      I       RRYDASFGRS 
  gi|295702435|BMD_0229|YP    57  7.75e-12 
    RRYSKGRSLH     E      D      I      V      Q      V      G      M      L      G      L      L      G      A      I       RRYDASFGRS 
  gi|295695448|Btus_0783|Y    169  8.93e-12 
    KRYVGRGMLF     L      D      L      I      Q      E      G      N      L      G      L      I      K      A      V       EKFDFRKGFK 
  gi|218232670|BCB4264_A10    57  1.18e-11 
    YRYSKGGPMH     E      D      I      I      Q      V      G      M      L      G      L      L      G      A      I       RRYDYSIGNA 
  gi|218896055|BCG9842_B42    57  1.18e-11 
    YRYSKGGPMH     E      D      I      I      Q      V      G      M      L      G      L      L      G      A      I       RRYDYSIGNA 
  gi|225862940|BCA_1029|YP    57  1.18e-11 
    YRYSKGGPMH     E      D      I      I      Q      V      G      M      L      G      L      L      G      A      I       RRYDYSIGNA 
  gi|227816152|BAMEG_3579|    57  1.18e-11 
    YRYSKGGPMH     E      D      I      I      Q      V      G      M      L      G      L      L      G      A      I       RRYDYSIGNA 
  gi|229604478|BAA_1086|YP    57  1.18e-11 
    YRYSKGGPMH     E      D      I      I      Q      V      G      M      L      G      L      L      G      A      I       RRYDYSIGNA 
  gi|30261116|BA0992|NP_84    57  1.18e-11 
    YRYSKGGPMH     E      D      I      I      Q      V      G      M      L      G      L      L      G      A      I       RRYDYSIGNA 
  gi|47526271|GBAA0992|YP_    57  1.18e-11 
    YRYSKGGPMH     E      D      I      I      Q      V      G      M      L      G      L      L      G      A      I       RRYDYSIGNA 
  gi|118476614|BALH_0889|Y    58  1.18e-11 
    YRYSKGGPMH     E      D      I      I      Q      V      G      M      L      G      L      L      G      A      I       RRYDYSIGNA 
  gi|52144331|BCZK0896|YP_    58  1.18e-11 
    YRYSKGGPMH     E      D      I      I      Q      V      G      M      L      G      L      L      G      A      I       RRYDYSIGNA 
  gi|218902186|BCAH820_106    57  1.18e-11 
    YRYSKGGPMH     E      D      I      I      Q      V      G      M      L      G      L      L      G      A      I       RRYDYSIGNA 
  gi|30019159|BC1004|NP_83    58  1.18e-11 
    YRYSKGGPMH     E      D      I      I      Q      V      G      M      L      G      L      L      G      A      I       RRYDYSIGNA 
  gi|222094730|BCQ_1068|YP    57  1.18e-11 
    YRYSKGGPMH     E      D      I      I      Q      V      G      M      L      G      L      L      G      A      I       RRYDYSIGNA 
  gi|217958581|BCAH187_A11    57  1.18e-11 
    YRYSKGGPMH     E      D      I      I      Q      V      G      M      L      G      L      L      G      A      I       RRYDYSIGNA 
  gi|49480184|BT9727_0913|    58  1.18e-11 
    YRYSKGGPMH     E      D      I      I      Q      V      G      M      L      G      L      L      G      A      I       RRYDYSIGNA 
  gi|49183950|BAS0928|YP_0    58  1.18e-11 
    YRYSKGGPMH     E      D      I      I      Q      V      G      M      L      G      L      L      G      A      I       RRYDYSIGNA 
  gi|42780162|BCE_1086|NP_    57  1.18e-11 
    YRYSKGGPMH     E      D      I      I      Q      V      G      M      L      G      L      L      G      A      I       RRYDYSIGNA 
  gi|163938900|BcerKBAB4_0    57  1.18e-11 
    YRYSKGGPMH     E      D      I      I      Q      V      G      M      L      G      L      L      G      A      I       RRYDYSIGNA 
  gi|301052635|BACI_c10250    58  1.18e-11 
    YRYSKGGPMH     E      D      I      I      Q      V      G      M      L      G      L      L      G      A      I       RRYDYSIGNA 
  gi|296501716|BMB171_C087    57  1.18e-11 
    YRYSKGGPMH     E      D      I      I      Q      V      G      M      L      G      L      L      G      A      I       RRYDYSIGNA 
  gi|152976319|Bcer98_2607    47  1.35e-11 
    AIKDDTGLEF     E      D      L      V      Q      C      G      M      I      A      L      I      K      A      R       EQFKPEMGFK 
  gi|169829726|Bsph_4295|Y    57  1.35e-11 
    RKYSNGKSYY     D      D      I      V      Q      V      G      M      L      G      L      L      G      A      I       RRFDPNVGRS 
  gi|52784380|BLi00560|YP_    58  1.55e-11 
    KKYSKGKSFH     E      D      L      C      Q      V      G      M      L      G      L      L      G      A      I       KRYDPDVGKS 
  gi|52079008|BL02208|YP_0    57  1.55e-11 
    KKYSKGKSFH     E      D      L      C      Q      V      G      M      L      G      L      L      G      A      I       KRYDPDVGKS 
  gi|23099294|OB1839|NP_69    58  1.55e-11 
    QRFINRGYDP     D      D      L      F      Q      I      G      S      I      G      L      I      K      S      I       DKFDLSYDVK 
  gi|169829306|Bsph_3856|Y    70  2.32e-11 
    KKFHPKHEQL     D      D      Y      I      S      I      G      T      I      G      L      M      K      A      V       ESYTPDKKTR 
  gi|163940286|BcerKBAB4_2    40  2.65e-11 
    FIITKDPHLS     D      D      I      T      Q      D      V      F      I      K      V      F      K      H      W       NSFRKESSIK 
  gi|255767137|BSU04730|NP    57  3.01e-11 
    KKYSKGKSFH     E      D      L      R      Q      V      G      M      I      G      L      L      G      A      I       KRYDPVVGKS 
  gi|154686834|RBAM_024040    77  3.01e-11 
    KKFENTGEDA     E      D      L      I      S      I      G      T      I      G      L      I      K      G      I       ESYSAGKGTK 
  gi|154684976|RBAM_005070    57  3.01e-11 
    KKYSKGKSFH     E      D      L      R      Q      V      G      M      I      G      L      L      G      A      I       KRYDPEVGKS 
  gi|157691239|BPUM_0446|Y    57  3.01e-11 
    KKYSKGKSFH     E      D      L      R      Q      V      G      M      I      G      L      L      G      A      I       KRYDPLVGKS 
  gi|42780207|BCE_1131|NP_    44  3.43e-11 
    YSYVNNKEVA     E      D      L      T      Q      D      I      F      V      K      C      Y      K      S      L       HTYKGNSNVK 
  gi|217958624|BCAH187_A12    44  3.43e-11 
    YSYVNNKEVA     E      D      L      T      Q      D      I      F      V      K      C      Y      K      S      L       HTYKGNSNLK 
  gi|222094769|BCQ_1107|YP    23  3.43e-11 
    YSYVNNKEVA     E      D      L      T      Q      D      I      F      V      K      C      Y      K      S      L       HTYKGNSNLK 
  gi|163938949|BcerKBAB4_0    44  3.43e-11 
    YSYVNNKEVA     E      D      L      T      Q      D      I      F      V      K      C      Y      K      S      L       HTYKGKSNVK 
  gi|52144286|BCZK0942|YP_    44  3.43e-11 
    YSYVNNKEVA     E      D      L      T      Q      D      I      F      V      K      C      Y      K      S      L       HTYKGNSNLK 
  gi|23099454|OB1999|NP_69    77  3.43e-11 
    KKFENTGEDM     E      D      L      I      S      I      G      T      I      G      L      I      K      G      V       ESFSTDKGTK 
  gi|225864482|BCA_2587|YP    40  3.90e-11 
    FIITKDPHLS     D      D      I      T      Q      D      V      F      I      K      V      F      K      N      W       NSFRKESSIK 
  gi|118477905|BALH_2250|Y    43  3.90e-11 
    FIITKDPHLS     D      D      I      T      Q      D      V      F      I      K      V      F      K      N      W       NSFRKESSIK 
  gi|42781595|BCE_2534|NP_    40  3.90e-11 
    FIITKDPHLS     D      D      I      T      Q      D      V      F      I      K      V      F      K      N      W       NSFRKESSIK 
  gi|217959986|BCAH187_A25    40  3.90e-11 
    FIITKDPHLS     D      D      I      T      Q      D      V      F      I      K      V      F      K      N      W       NSFRKESSIK 
  gi|218903633|BCAH820_251    40  3.90e-11 
    FIITKDPHLS     D      D      I      T      Q      D      V      F      I      K      V      F      K      N      W       NSFRRESSIK 
  gi|52142997|BCZK2243|YP_    42  3.90e-11 
    FIITKDPHLS     D      D      I      T      Q      D      V      F      I      K      V      F      K      N      W       NSFRKESSIK 
  gi|218231851|BCB4264_A24    40  3.90e-11 
    FIITKDPHLS     D      D      I      T      Q      D      V      F      I      K      V      F      K      N      W       NSFRKESSIK 
  gi|30020599|BC2469|NP_83    42  3.90e-11 
    FIITKDPHLS     D      D      I      T      Q      D      V      F      I      K      V      F      K      N      W       NSFRKESSIK 
  gi|222096073|BCQ_2413|YP    40  3.90e-11 
    FIITKDPHLS     D      D      I      T      Q      D      V      F      I      K      V      F      K      N      W       NSFRKESSIK 
  gi|49479242|BT9727_2287|    42  3.90e-11 
    FIITKDPHLS     D      D      I      T      Q      D      V      F      I      K      V      F      K      N      W       NSFRKESSIK 
  gi|218897485|BCG9842_B28    40  3.90e-11 
    FIITKDPHLS     D      D      I      T      Q      D      V      F      I      K      V      F      K      N      W       NSFRKESSIK 
  gi|52787786|BLi04109|YP_    47  3.90e-11 
    LKLTLNPDLT     E      D      L      V      Q      E      T      M      L      K      A      Y      V      S      L       HQFQGNAKFS 
  gi|301054038|BACI_c24720    40  3.90e-11 
    FIITKDPHLS     D      D      I      T      Q      D      V      F      I      K      V      F      K      N      W       NSFRKESSIK 
  gi|261409476|GYMC10_5703    46  3.90e-11 
    YRMLSNRHEA     E      D      V      V      Q      E      T      F      L      R      V      Y      K      N      L       DRYDENQKFS 
  gi|296503062|BMB171_C223    40  3.90e-11 
    FIITKDPHLS     D      D      I      T      Q      D      V      F      I      K      V      F      K      N      W       NSFRKESSIK 
  gi|52082396|BL00940|YP_0    47  3.90e-11 
    LKLTLNPDLT     E      D      L      V      Q      E      T      M      L      K      A      Y      V      S      L       HQFQGNAKFS 
  gi|261405673|GYMC10_1824    86  5.02e-11 
    RKFENTGINI     E      D      L      V      S      I      G      A      I      G      L      I      K      A      V       NTFDPEKKIK 
  gi|288554854|BpOF4_09205    57  5.02e-11 
    RKFSKGREYD     E      D      L      Y      Q      V      G      M      V      G      L      L      A      A      L       TRFDSEFGRS 
  gi|49476853|BT9727_0558|    37  8.21e-11 
    YSILNNIQQA     E      D      T      V      Q      E      T      F      I      T      L      Y      K      N      L       EKLHNLNIQE 
  gi|169827019|Bsph_1443|Y    49  8.21e-11 
    RRFDNTGTPI     E      D      L      I      S      I      G      S      I      G      L      I      K      A      I       ETFNTDKNIK 
  gi|118476646|BALH_0922|Y    44  1.32e-10 
    YSYVNNKEVA     E      D      V      T      Q      D      I      F      V      K      C      Y      K      S      L       HTYKGNSNLK 
  gi|30261155|BA1032|NP_84    44  1.32e-10 
    YSYVNNKEVA     E      D      V      T      Q      D      I      F      V      K      C      Y      K      S      L       HTYKGNSNLK 
  gi|218902228|BCAH820_111    44  1.32e-10 
    YSYVNNKEVA     E      D      V      T      Q      D      I      F      V      K      C      Y      K      S      L       HTYKGNSNLK 
  gi|225862980|BCA_1069|YP    44  1.32e-10 
    YSYVNNKEVA     E      D      V      T      Q      D      I      F      V      K      C      Y      K      S      L       HTYKGNSNLK 
  gi|49477062|BT9727_0953|    44  1.32e-10 
    YSYVNNKEVA     E      D      V      T      Q      D      I      F      V      K      C      Y      K      S      L       HTYKGNSNLK 
  gi|227816114|BAMEG_3540|    44  1.32e-10 
    YSYVNNKEVA     E      D      V      T      Q      D      I      F      V      K      C      Y      K      S      L       HTYKGNSNLK 
  gi|47777889|GBAA1032|YP_    44  1.32e-10 
    YSYVNNKEVA     E      D      V      T      Q      D      I      F      V      K      C      Y      K      S      L       HTYKGNSNLK 
  gi|218231966|BCB4264_A10    44  1.32e-10 
    YSYVNNKEVA     E      D      V      T      Q      D      I      F      V      K      C      Y      K      S      L       HTYKGKSNVK 
  gi|218896095|BCG9842_B42    44  1.32e-10 
    YSYVNNKEIA     E      D      V      T      Q      D      I      F      V      K      C      Y      K      S      L       HTYKGKSNVK 
  gi|229604778|BAA_1124|YP    44  1.32e-10 
    YSYVNNKEVA     E      D      V      T      Q      D      I      F      V      K      C      Y      K      S      L       HTYKGNSNLK 
  gi|49183986|BAS0964|YP_0    73  1.32e-10 
    YSYVNNKEVA     E      D      V      T      Q      D      I      F      V      K      C      Y      K      S      L       HTYKGNSNLK 
  gi|301052678|BACI_c10700    44  1.32e-10 
    YSYVNNKEVA     E      D      V      T      Q      D      I      F      V      K      C      Y      K      S      L       HTYKGNSNLK 
  gi|296501752|BMB171_C091    73  1.32e-10 
    YSYVNNKEVA     E      D      V      T      Q      D      I      F      V      K      C      Y      K      S      L       HTYKGKSNVK 
  gi|212638144|Aflv_0295|Y    33  1.49e-10 
    YVYMRNEHDA     E      D      V      F      Q      D      V      F      I      K      L      Y      E      H      M       GRIQSEEHMK 
  gi|56962590|ABC0816|YP_1    57  1.49e-10 
    RKFSKGREYD     E      D      L      Y      Q      V      G      M      V      G      L      L      A      A      M       KRYNEDYGRS 
  gi|295696809|Btus_2226|Y    47  1.49e-10 
    RRLTGNRDDA     L      D      L      T      Q      E      T      F      I      R      A      F      R      H      L       DRFQPGRPFK 
  gi|16077241|BSU01730|NP_    46  1.87e-10 
    YRMLGNVHEA     E      D      I      A      Q      E      A      F      I      R      A      Y      V      N      I       DSFDINRKFS 
  gi|52784027|BLi00199|YP_    46  1.87e-10 
    YRMLGNAHEA     E      D      I      A      Q      E      A      F      I      R      A      Y      V      N      I       DSFDINRKFS 
  gi|154684695|RBAM_002260    46  1.87e-10 
    YRMLGNVHEA     E      D      I      A      Q      E      A      F      I      R      A      Y      V      N      I       DSFDINRKFS 
  gi|157690957|BPUM_0160|Y    54  1.87e-10 
    YRMLGNAHEA     E      D      I      A      Q      E      A      F      I      R      A      Y      V      N      I       ESFDVNRKFS 
  gi|52078665|BL02699|YP_0    46  1.87e-10 
    YRMLGNAHEA     E      D      I      A      Q      E      A      F      I      R      A      Y      V      N      I       DSFDINRKFS 
  gi|261409365|GYMC10_5592    45  1.87e-10 
    TQRLADRQKA     Q      D      I      V      Q      E      T      F      I      R      L      I      R      H      L       KQHGTLEHVR 
  gi|217958232|BCAH187_A07    37  2.10e-10 
    YSILNNIQQA     E      D      S      V      Q      E      T      F      I      T      L      Y      K      N      L       EKLHSLSTEE 
  gi|52786190|BLi02449|YP_    31  2.10e-10 
    FYMVKDKNQA     E      D      L      V      Q      E      V      Y      I      R      V      L      H      S      Y       ETFEGRSSEK 
  gi|157692810|BPUM_2042|Y    31  2.10e-10 
    FYMVKDKNQA     E      D      L      V      Q      E      V      Y      I      R      V      L      H      S      Y       KTFEGRSSEK 
  gi|52080819|BL00651|YP_0    31  2.10e-10 
    FYMVKDKNQA     E      D      L      V      Q      E      V      Y      I      R      V      L      H      S      Y       ETFEGRSSEK 
  gi|239827551|GWCH70_2200    31  2.63e-10 
    FYMVRNREQA     E      D      L      V      Q      E      V      Y      I      K      V      L      R      S      Y       KRFKGESSEK 
  gi|49480428|BT9727_5044|    32  2.94e-10 
    YKLAKTKEDA     E      D      I      F      Q      E      T      W      I      K      V      F      S      S      R       HQLSYVENYK 
  gi|49188198|BAS5212|YP_0    31  2.94e-10 
    YKLAKTKEDA     E      D      I      F      Q      E      T      W      I      K      V      F      S      S      R       HQLSYVENYK 
  gi|218906540|BCAH820_545    31  2.94e-10 
    YKLAKTKEDA     E      D      I      F      Q      E      T      W      I      K      V      F      S      S      R       HQLSYVENYK 
  gi|222097738|BCQ_4079|YP    165  2.94e-10 
    KRYVGRGMLF     L      D      L      I      Q      E      G      N      M      G      L      I      K      A      G       EKFDYRKGFK 
  gi|225867344|BCA_5512|YP    31  2.94e-10 
    YKLAKTKEDA     E      D      I      F      Q      E      T      W      I      K      V      F      S      S      R       HQLSYVENYK 
  gi|227818128|BAMEG_5654|    31  2.94e-10 
    YKLAKTKEDA     E      D      I      F      Q      E      T      W      I      K      V      F      S      S      R       HQLSYVENYK 
  gi|217962852|BCAH187_A55    31  2.94e-10 
    YKLAKTKEDA     E      D      I      F      Q      E      T      W      I      K      V      F      S      S      R       HQLSYVENYK 
  gi|42784537|BCE_5492|NP_    31  2.94e-10 
    YKLAKTKEDA     E      D      I      F      Q      E      T      W      I      K      V      F      S      S      R       HQLSYVENHK 
  gi|218900489|BCG9842_B54    31  2.94e-10 
    YKLAKTKEDA     E      D      I      F      Q      E      T      W      I      K      V      F      S      S      R       HQLSYVDNYK 
  gi|118480394|BALH_4859|Y    63  2.94e-10 
    YKLAKTKEDA     E      D      I      F      Q      E      T      W      I      K      V      F      S      S      R       HQLSYVENYK 
  gi|52140200|BCZK5060|YP_    31  2.94e-10 
    YKLAKTKEDA     E      D      I      F      Q      E      T      W      I      K      V      F      S      S      R       HQLSYVENYK 
  gi|30023393|BC5363|NP_83    32  2.94e-10 
    YKLAKTKEDA     E      D      I      F      Q      E      T      W      I      K      V      F      S      S      R       HQLSYVENYK 
  gi|30265385|BA5610|NP_84    31  2.94e-10 
    YKLAKTKEDA     E      D      I      F      Q      E      T      W      I      K      V      F      S      S      R       HQLSYVENYK 
  gi|229601723|BAA_5636|YP    31  2.94e-10 
    YKLAKTKEDA     E      D      I      F      Q      E      T      W      I      K      V      F      S      S      R       HQLSYVENYK 
  gi|222098834|BCQ_5203|YP    31  2.94e-10 
    YKLAKTKEDA     E      D      I      F      Q      E      T      W      I      K      V      F      S      S      R       HQLSYVENYK 
  gi|47530932|GBAA5610|YP_    31  2.94e-10 
    YKLAKTKEDA     E      D      I      F      Q      E      T      W      I      K      V      F      S      S      R       HQLSYVENYK 
  gi|301056831|BACI_c53580    31  2.94e-10 
    YKLAKTKEDA     E      D      I      F      Q      E      T      W      I      K      V      F      S      S      R       HQLSYVENYK 
  gi|296505785|BMB171_C495    31  2.94e-10 
    YKLAKTKEDA     E      D      I      F      Q      E      T      W      I      K      V      F      S      S      R       HQLSYVENYK 
  gi|261406063|GYMC10_2217    47  3.29e-10 
    LKSSHMELMA     E      D      L      C      S      E      T      F      Y      K      A      F      R      S      L       HSFREVDASF 
  gi|30019269|BC1114|NP_83    33  3.67e-10 
    LSLTGDSHYA     E      D      L      M      Q      E      T      F      Y      R      M      L      V      H      I       DYYKGEEIRP 
  gi|30261223|BA1113|NP_84    33  3.67e-10 
    LSLTGDSHFA     E      D      L      M      Q      E      T      F      Y      R      M      L      V      H      I       DYYKGEEIRP 
  gi|49184055|BAS1035|YP_0    33  3.67e-10 
    LSLTGDSHFA     E      D      L      M      Q      E      T      F      Y      R      M      L      V      H      I       DYYKGEEIRP 
  gi|218896153|BCG9842_B41    33  3.67e-10 
    LSLTGDSPYA     E      D      L      M      Q      E      T      F      Y      R      M      L      V      H      I       DYYKGEEIRP 
  gi|47526385|GBAA1113|YP_    33  3.67e-10 
    LSLTGDSHFA     E      D      L      M      Q      E      T      F      Y      R      M      L      V      H      I       DYYKGEEIRP 
  gi|218902311|BCAH820_119    33  3.67e-10 
    LSLTGDSHFA     E      D      L      M      Q      E      T      F      Y      R      M      L      V      H      I       DYYKGEEIRP 
  gi|229602397|BAA_1198|YP    33  3.67e-10 
    LSLTGDSHFA     E      D      L      M      Q      E      T      F      Y      R      M      L      V      H      I       DYYKGEEIRP 
  gi|225863070|BCA_1159|YP    33  3.67e-10 
    LSLTGDSHFA     E      D      L      M      Q      E      T      F      Y      R      M      L      V      H      I       DYYKGEEIRP 
  gi|163939015|BcerKBAB4_1    33  3.67e-10 
    LSLTGDSHSA     E      D      L      M      Q      E      T      F      Y      R      M      L      V      H      I       DYYKGEEIRP 
  gi|227816040|BAMEG_3466|    33  3.67e-10 
    LSLTGDSHFA     E      D      L      M      Q      E      T      F      Y      R      M      L      V      H      I       DYYKGEEIRP 
  gi|222094839|BCQ_1177|YP    33  3.67e-10 
    LSLTGDSHFA     E      D      L      M      Q      E      T      F      Y      R      M      L      V      H      I       DYYKGEEIRP 
  gi|218232533|BCB4264_A11    33  3.67e-10 
    LSLTGDSHYA     E      D      L      M      Q      E      T      F      Y      R      M      L      V      H      I       DYYKGEEIRP 
  gi|217958692|BCAH187_A12    33  3.67e-10 
    LSLTGDSHFA     E      D      L      M      Q      E      T      F      Y      R      M      L      V      H      I       DYYKGEEIRP 
  gi|42780290|BCE_1215|NP_    33  3.67e-10 
    LSLTGDSHFA     E      D      L      M      Q      E      T      F      Y      R      M      L      V      H      I       DYYKGEEIRP 
  gi|52144214|BCZK1013|YP_    33  3.67e-10 
    LSLTGDCHYA     E      D      L      M      Q      E      T      F      Y      R      M      L      V      H      I       DYYKGEEIRP 
  gi|49477092|BT9727_1012|    33  3.67e-10 
    LSLTGDSHFA     E      D      L      M      Q      E      T      F      Y      R      M      L      V      H      I       DYYKGEEIRP 
  gi|138893826|GTNG_0147|Y    21  3.67e-10 
    YRMLGNRHEA     E      D      A      A      Q      E      A      F      I      R      A      Y      V      H      I       DTYNPNMKFS 
  gi|301052754|BACI_c11460    33  3.67e-10 
    LSLTGDSHFA     E      D      L      M      Q      E      T      F      Y      R      M      L      V      H      I       DYYKGEEIRP 
  gi|15613092|BH0529|NP_24    57  3.67e-10 
    RKFSRSRAHD     D      D      L      I      Q      V      G      M      V      G      L      L      A      A      F       ERYDPKYGRS 
  gi|261404455|GYMC10_0586    32  3.67e-10 
    YSLSYDHHTA     E      D      L      V      Q      E      T      F      Y      R      A      Y      L      Y      L       EDWKEERIKP 
  gi|261408152|GYMC10_4361    38  3.67e-10 
    LSLTNSSWDS     D      D      L      V      Q      D      T      W      I      K      A      L      E      H      L       QQRSHANPEA 
  gi|169827149|Bsph_1579|Y    69  3.67e-10 
    SAGLPKSVSR     D      D      L      T      S      L      G      M      I      G      L      F      D      A      L       NKFDINRDLK 
  gi|227815066|BAMEG_2477|    48  4.09e-10 
    HFLIEDKTDV     D      D      V      V      Q      E      V      Y      I      Q      L      Y      E      S      L       RKYDSEKPFR 
  gi|49184977|BAS1966|YP_0    48  4.09e-10 
    HFLIEDKTDV     D      D      V      V      Q      E      V      Y      I      Q      L      Y      E      S      L       RKYDSEKPFR 
  gi|229602619|BAA_2180|YP    48  4.09e-10 
    HFLIEDKTDV     D      D      V      V      Q      E      V      Y      I      Q      L      Y      E      S      L       RKYDSEKPFR 
  gi|30262135|BA2114|NP_84    48  4.09e-10 
    HFLIEDKTDV     D      D      V      V      Q      E      V      Y      I      Q      L      Y      E      S      L       RKYDSEKPFR 
  gi|30020245|BC2108|NP_83    48  4.09e-10 
    HFLIEDKTDV     D      D      V      V      Q      E      V      Y      I      Q      L      Y      E      S      L       RKYDSEKPFR 
  gi|47527408|GBAA2114|YP_    48  4.09e-10 
    HFLIEDKTDV     D      D      V      V      Q      E      V      Y      I      Q      L      Y      E      S      L       RKYDSEKPFR 
  gi|218232753|BCB4264_A21    48  4.09e-10 
    HFLIEDKTDV     D      D      V      V      Q      E      V      Y      I      Q      L      Y      E      S      L       RKYDSEKPFR 
  gi|296502728|BMB171_C189    48  4.09e-10 
    HFLIEDKTDV     D      D      V      V      Q      E      V      Y      I      Q      L      Y      E      S      L       RKYDSGKPFR 
  gi|157692327|BPUM_1546|Y    53  4.56e-10 
    SIGLPKSVHK     E      D      L      I      S      L      G      M      L      G      L      Y      D      A      L       EKFDPGRDLK 
  gi|261406105|GYMC10_2260    23  4.56e-10 
    YSVLRDAKEA     E      D      A      S      Q      E      V      F      I      Q      V      Y      K      A      L       PQYRSEGFKT 
  gi|295695216|Btus_0545|Y    35  4.56e-10 
    RSLIAAPDAV     E      D      L      A      Q      E      V      F      V      R      L      Y      R      H      P       PDDPSKVAPW 
  gi|212637996|Aflv_0147|Y    46  5.07e-10 
    YRMLGNRHEA     E      D      A      A      Q      E      A      F      I      R      A      Y      V      N      I       HTYDPTMKFS 
  gi|239825735|GWCH70_0154    42  5.07e-10 
    YRMLGNRHEA     E      D      A      A      Q      E      A      F      I      R      A      Y      V      N      I       HTYNPNMRFS 
  gi|52785627|BLi01868|YP_    53  5.07e-10 
    AVGLPKSVHK     D      D      L      V      S      L      G      M      L      G      L      Y      D      A      L       EKFDPGRDLK 
  gi|52080250|BL01246|YP_0    53  5.07e-10 
    AVGLPKSVHK     D      D      L      V      S      L      G      M      L      G      L      Y      D      A      L       EKFDPGRDLK 
  gi|261406462|GYMC10_2625    57  5.65e-10 
    YYILKNHAET     E      D      A      V      Q      E      I      F      I      R      A      Y      E      H      L       HLYKRQVSFS 
  gi|23097683|OB0228|NP_69    46  5.65e-10 
    YRMLGNKHEA     E      D      I      A      Q      E      A      F      I      R      A      Y      I      N      I       DSFDEKRKFS 
  gi|218233984|BCB4264_A53    31  6.28e-10 
    IKKLGIRKDY     E      E      Y      Y      Q      C      G      L      I      G      L      W      H      A      Y       ERFDEKKGYF 
  gi|225867222|BCA_5390|YP    31  6.28e-10 
    IKKLGIYQDY     E      E      Y      Y      Q      C      G      L      I      G      L      W      H      A      Y       ERYDAKKGSF 
  gi|30023283|BC5251|NP_83    31  6.28e-10 
    IKKLGIRKDY     E      E      Y      Y      Q      C      G      L      I      G      L      W      H      A      Y       ERFDEKKGYF 
  gi|227814691|BAMEG_2101|    40  6.28e-10 
    FIITKDPHLS     G      D      I      T      Q      D      V      F      I      K      V      F      K      N      W       NSFRRESSIK 
  gi|227818013|BAMEG_5539|    31  6.28e-10 
    IKKLGIYQDY     E      E      Y      Y      Q      C      G      L      I      G      L      W      H      A      Y       ERYDAKKGSF 
  gi|30265276|BA5493|NP_84    21  6.28e-10 
    IKKLGIYQDY     E      E      Y      Y      Q      C      G      L      I      G      L      W      H      A      Y       ERYDAKKGSF 
  gi|47527789|GBAA2502|YP_    40  6.28e-10 
    FIITKDPHLS     G      D      I      T      Q      D      V      F      I      K      V      F      K      N      W       NSFRRESSIK 
  gi|217962737|BCAH187_A54    31  6.28e-10 
    IKKLGIYQDY     E      E      Y      Y      Q      C      G      L      I      G      L      W      H      A      Y       EKFDAEKGSF 
  gi|222098712|BCQ_5081|YP    31  6.28e-10 
    IKKLGIYQDY     E      E      Y      Y      Q      C      G      L      I      G      L      W      H      A      Y       EKFDAEKGSF 
  gi|229603576|BAA_5520|YP    31  6.28e-10 
    IKKLGIYQDY     E      E      Y      Y      Q      C      G      L      I      G      L      W      H      A      Y       ERYDAKKGSF 
  gi|49481227|BT9727_4932|    21  6.28e-10 
    IKKLGIYQDY     E      E      Y      Y      Q      C      G      L      I      G      L      W      H      A      Y       ERYDAKKGSF 
  gi|163942935|BcerKBAB4_5    31  6.28e-10 
    IKRLNIYQDY     E      E      Y      Y      Q      C      G      L      I      G      L      W      H      A      Y       ERYEEEKGSF 
  gi|218900375|BCG9842_B55    31  6.28e-10 
    IKKLGIRQDY     E      E      Y      Y      Q      C      G      L      I      G      L      W      H      A      Y       ERFDAKKGYF 
  gi|49185332|BAS2323|YP_0    42  6.28e-10 
    FIITKDPHLS     G      D      I      T      Q      D      V      F      I      K      V      F      K      N      W       NSFRRESSIK 
  gi|30262499|BA2502|NP_84    40  6.28e-10 
    FIITKDPHLS     G      D      I      T      Q      D      V      F      I      K      V      F      K      N      W       NSFRRESSIK 
  gi|42784415|BCE_5370|NP_    31  6.28e-10 
    IKRLGIYQDY     E      E      Y      Y      Q      C      G      L      I      G      L      W      H      A      Y       EKFDAEKGSF 
  gi|52140312|BCZK4947|YP_    31  6.28e-10 
    IKKLGIYQDY     E      E      Y      Y      Q      C      G      L      I      G      L      W      H      A      Y       ERYDAKKGSF 
  gi|47530811|GBAA5493|YP_    21  6.28e-10 
    IKKLGIYQDY     E      E      Y      Y      Q      C      G      L      I      G      L      W      H      A      Y       ERYDAKKGSF 
  gi|49188088|BAS5102|YP_0    21  6.28e-10 
    IKKLGIYQDY     E      E      Y      Y      Q      C      G      L      I      G      L      W      H      A      Y       ERYDAKKGSF 
  gi|229604897|BAA_2557|YP    40  6.28e-10 
    FIITKDPHLS     G      D      I      T      Q      D      V      F      I      K      V      F      K      N      W       NSFRRESSIK 
  gi|295704249|BMD_2121|YP    33  6.28e-10 
    LSLTRRKESA     E      D      L      M      Q      E      T      F      Y      R      A      L      L      H      L       ETYKGEEVKP 
  gi|296505675|BMB171_C484    31  6.28e-10 
    IKKLGIRKDY     E      E      Y      Y      Q      C      G      L      I      G      L      W      H      A      Y       ERFDAKKGYF 
  gi|294498927|BMQ_2164|YP    33  6.28e-10 
    LSLTRRKESA     E      D      L      M      Q      E      T      F      Y      R      A      L      L      H      L       ETYKGEEVKP 
  gi|301056717|BACI_c52440    31  6.28e-10 
    IKKLGIYQDY     E      E      Y      Y      Q      C      G      L      I      G      L      W      H      A      Y       ERYDAKKGSF 
  gi|15613235|BH0672|NP_24    30  6.28e-10 
    YRLLGSVTDA     E      D      L      V      Q      E      T      F      L      R      A      Y      Q      A      E       EREINNKKAY 
  gi|288556053|BpOF4_15235    43  6.97e-10 
    FQLGAKESSV     E      D      I      T      Q      E      V      F      I      K      V      Y      R      F      L       SKHTRGKFTT 
  gi|227814227|BAMEG_1635|    48  7.74e-10 
    FLVGSQNQDV     D      D      I      V      N      E      V      Y      I      K      M      W      K      S      V       TNYDMNRSFR 
  gi|49185764|BAS2758|YP_0    48  7.74e-10 
    FLVGSQNQDV     D      D      I      V      N      E      V      Y      I      K      M      W      K      S      V       TNYDMNRSFR 
  gi|218904082|BCAH820_296    48  7.74e-10 
    FLVGSQNQDV     D      D      I      V      N      E      V      Y      I      K      M      W      K      S      V       TNYDMNRSFR 
  gi|161486563|BA2970|NP_8    48  7.74e-10 
    FLVGSQNQDV     D      D      I      V      N      E      V      Y      I      K      M      W      K      S      V       TNYDMNRSFR 
  gi|229602282|BAA_3022|YP    48  7.74e-10 
    FLVGSQNQDV     D      D      I      V      N      E      V      Y      I      K      M      W      K      S      V       TNYDMNRSFR 
  gi|161611198|GBAA2970|YP    48  7.74e-10 
    FLVGSQNQDV     D      D      I      V      N      E      V      Y      I      K      M      W      K      S      V       TNYDMNRSFR 
  gi|15615924|BH3362|NP_24    47  7.74e-10 
    YSYVKDKQIA     E      D      L      T      Q      E      V      F      V      R      A      F      Q      S      Y       HTYQTRSSAK 
  gi|49478105|BT9727_3026|    29  8.58e-10 
    FSLSRSHHVA     E      D      L      M      Q      E      T      F      Y      R      A      Y      L      Y      L       EDYENQKVKS 
  gi|218904418|BCAH820_330    29  8.58e-10 
    FSLSRSHHVA     E      D      L      M      Q      E      T      F      Y      R      A      Y      L      Y      L       EDYENQKVKS 
  gi|52142270|BCZK2972|YP_    29  8.58e-10 
    FSLSRSHHVA     E      D      L      M      Q      E      T      F      Y      R      A      Y      L      Y      L       EDYENQKVKS 
  gi|47528609|GBAA3324|YP_    29  8.58e-10 
    FSLSRSHHAA     E      D      L      M      Q      E      T      F      Y      R      A      Y      L      Y      L       EDYENQKVKS 
  gi|225865247|BCA_3356|YP    29  8.58e-10 
    FSLSRSHHAA     E      D      L      M      Q      E      T      F      Y      R      A      Y      L      Y      L       EDYENQKVKS 
  gi|227813895|BAMEG_1301|    29  8.58e-10 
    FSLSRSHHAA     E      D      L      M      Q      E      T      F      Y      R      A      Y      L      Y      L       EDYENQKVKS 
  gi|229604117|BAA_3359|YP    29  8.58e-10 
    FSLSRSHHAA     E      D      L      M      Q      E      T      F      Y      R      A      Y      L      Y      L       EDYENQKVKS 
  gi|49186087|BAS3082|YP_0    29  8.58e-10 
    FSLSRSHHAA     E      D      L      M      Q      E      T      F      Y      R      A      Y      L      Y      L       EDYENQKVKS 
  gi|30263235|BA3324|NP_84    29  8.58e-10 
    FSLSRSHHAA     E      D      L      M      Q      E      T      F      Y      R      A      Y      L      Y      L       EDYENQKVKS 
  gi|163940993|BcerKBAB4_3    29  8.58e-10 
    FSLSRSHHVA     E      D      L      M      Q      E      T      F      Y      R      A      Y      L      Y      L       EDYENQKVKS 
  gi|301054766|BACI_c32210    46  8.58e-10 
    FSLSRSHHVA     E      D      L      M      Q      E      T      F      Y      R      A      Y      L      Y      L       EDYENQKVKS 
  gi|56420261|GK1726|YP_14    23  9.51e-10 
    YRLTNDYHLA     E      D      L      L      Q      E      T      F      Y      K      V      Y      L      H      V       SGISDIAQIK 
  gi|23100547|OB3092|NP_69    46  9.51e-10 
    YAISKNKNDA     S      D      I      V      Q      E      T      F      I      R      V      F      R      N      I       DSYDINRPFR 
  gi|56964615|ABC2851|YP_1    37  9.51e-10 
    WSIRSSWFHY     S      D      L      I      S      V      G      T      I      G      L      I      N      A      V       ENYENKKGIT 
  gi|56418685|GK0150|YP_14    46  1.05e-09 
    YRMLGNRHEA     E      D      A      A      Q      E      A      F      I      R      A      Y      I      H      I       DTYNPEMKFS 
  gi|16078017|BSU09520|NP_    29  1.05e-09 
    LSMTKDKHLA     E      D      L      L      Q      E      T      F      M      R      A      Y      I      H      I       HSYDHSKVKP 
  gi|288553039|BpOF4_00065    54  1.05e-09 
    SVGLPKNVSK     D      D      L      T      S      H      G      M      L      G      L      Y      D      A      L       EKFNPNRDLK 
  gi|52784792|BLi01020|YP_    29  1.05e-09 
    LSMTKDKHLA     E      D      L      L      Q      E      T      F      M      R      A      Y      I      H      I       HSYDHSKVKP 
  gi|154686555|RBAM_021240    31  1.05e-09 
    FYMVKDKSQA     E      D      L      T      Q      E      V      Y      I      R      V      L      N      S      Y       HTFEGRSSEK 
  gi|261417650|GYMC61_0150    49  1.05e-09 
    YRMLGNRHEA     E      D      A      A      Q      E      A      F      I      R      A      Y      I      H      I       DTYNPEMKFS 
  gi|52079432|BL02851|YP_0    29  1.05e-09 
    LSMTKDKHLA     E      D      L      L      Q      E      T      F      M      R      A      Y      I      H      I       HSYDHSKVKP 
  gi|157691684|BPUM_0902|Y    29  1.05e-09 
    LSMTKDKHLA     E      D      L      L      Q      E      T      F      M      R      A      Y      I      H      I       HSYDHSKVKP 
  gi|154685410|RBAM_009760    29  1.05e-09 
    LSMTKDKHLA     E      D      L      L      Q      E      T      F      M      R      A      Y      I      H      I       HSYDHSKVKP 
  gi|297528524|GC56T3_0148    49  1.05e-09 
    YRMLGNRHEA     E      D      A      A      Q      E      A      F      I      R      A      Y      I      H      I       DTYNPEMKFS 
  gi|138895828|GTNG_2187|Y    31  1.05e-09 
    FYMVRNREHA     E      D      L      V      Q      E      V      Y      V      K      V      L      R      S      Y       KRFKGQCSEK 
  gi|16078710|BSU16470|NP_    53  1.17e-09 
    SVGLPKSVHK     D      D      L      M      S      L      G      M      L      G      L      Y      D      A      L       EKFDPSRDLK 
  gi|154686064|RBAM_016310    53  1.17e-09 
    SVGLPKSVHK     D      D      L      M      S      L      G      M      L      G      L      Y      D      A      L       EKFDPGRDLK 
  gi|294509181|BMQ_pBM5009    59  1.17e-09 
    KRYFLLGGDR     E      D      L      L      Q      E      A      R      L      G      L      Y      K      A      V       RDFKTEGMSS 
  gi|261404422|GYMC10_0553    44  1.29e-09 
    AAVTGSSAGA     A      D      V      V      Q      E      T      F      I      R      V      Y      R      N      L       GSYDLSRPFE 
  gi|217959830|BCAH187_A24    29  1.42e-09 
    FSLSRSHHVA     E      D      L      M      Q      E      T      F      Y      R      A      F      L      Y      L       EDYENQKVKS 
  gi|222095915|BCQ_2255|YP    29  1.42e-09 
    FSLSRSHHVA     E      D      L      M      Q      E      T      F      Y      R      A      F      L      Y      L       EDYENQKVKS 
  gi|56420789|GK2254|YP_14    31  1.42e-09 
    FYMVRNREQA     E      D      L      V      Q      E      V      Y      V      R      V      L      R      S      Y       KRFKGQCSEK 
  gi|261417910|GYMC61_0428    31  1.42e-09 
    FYMVRNREQA     E      D      L      V      Q      E      V      Y      V      R      V      L      R      S      Y       KRFKGQCSEK 
  gi|297529579|GC56T3_1250    31  1.42e-09 
    FYMVRNREQA     E      D      L      V      Q      E      V      Y      V      R      V      L      R      S      Y       KRFKGQCSEK 
  gi|261408790|GYMC10_5011    31  1.57e-09 
    MRISGDPWEA     E      D      L      L      Q      D      V      M      M      K      V      I      R      A      V       ETDPGKHLSN 
  gi|52143316|BCZK1921|YP_    48  1.74e-09 
    HFLIDDKADV     D      D      V      V      Q      E      I      Y      I      Q      L      Y      E      S      L       RKYDSEKPFR 
  gi|118477553|BALH_1878|Y    57  1.74e-09 
    HFLIEDKTDV     D      D      V      V      Q      E      I      Y      I      Q      L      Y      E      S      L       RKYDSEKPFR 
  gi|222095751|BCQ_2091|YP    48  1.74e-09 
    HFLIDDKTDV     D      D      V      V      Q      E      I      Y      I      Q      L      Y      E      S      L       RKYDSEKPFR 
  gi|42781261|BCE_2195|NP_    48  1.74e-09 
    HFLIDDKADV     D      D      V      V      Q      E      I      Y      I      Q      L      Y      E      S      L       RKYDSEKPFR 
  gi|163939932|BcerKBAB4_1    48  1.74e-09 
    HFLIEDKADI     D      D      V      V      Q      E      I      Y      I      Q      L      Y      E      S      L       RKYDSEKPFR 
  gi|217959667|BCAH187_A22    48  1.74e-09 
    HFLIDDKTDV     D      D      V      V      Q      E      I      Y      I      Q      L      Y      E      S      L       RKYDSEKPFR 
  gi|49477520|BT9727_1945|    48  1.74e-09 
    HFLIEDKTDV     D      D      V      V      Q      E      I      Y      I      Q      L      Y      E      S      L       RKYDSEKPFR 
  gi|225864098|BCA_2202|YP    48  1.74e-09 
    HFLIEDKTDV     D      D      V      V      Q      E      I      Y      I      Q      L      Y      E      S      L       RKYDSEKPFR 
  gi|218903264|BCAH820_214    48  1.74e-09 
    HFLIEDKTDV     D      D      V      V      Q      E      I      Y      I      Q      L      Y      E      S      L       RKYDSEKPFR 
  gi|218897120|BCG9842_B31    48  1.74e-09 
    HFLIEDKTDV     D      D      V      V      Q      E      I      Y      I      Q      L      Y      E      S      L       RKYDSEKPFR 
  gi|301053658|BACI_c20780    48  1.74e-09 
    HFLIDDKADV     D      D      V      V      Q      E      I      Y      I      Q      L      Y      E      S      L       RKYDSEKPFR 
  gi|15616194|BH3632|NP_24    46  1.74e-09 
    RYLTGSPWDG     E      D      L      Y      Q      E      T      L      V      K      A      F      G      S      L       YQRWQPTNPK 
  gi|138894123|GTNG_0449|Y    43  2.11e-09 
    AAVTGSSANA     A      D      A      V      Q      E      T      F      I      R      V      Y      D      H      I       GRFDPAKPFR 
  gi|288555447|BpOF4_12185    45  2.11e-09 
    FSYVHQQDIA     E      D      L      A      Q      E      V      F      V      K      V      Y      Q      K      I       DQFEQRASLK 
  gi|15614994|BH2431|NP_24    54  2.32e-09 
    SVGLPRSVSK     D      D      L      I      S      H      G      M      L      G      L      L      D      A      L       EKFNPDRDLK 
  gi|30262705|BA2732|NP_84    42  2.56e-09 
    IWIIKEQTVA     E      E      L      A      Q      E      V      F      L      Q      L      Y      H      S      D       WKAIENLRAW 
  gi|227814455|BAMEG_1863|    42  2.56e-09 
    IWIIKEQTVA     E      E      L      A      Q      E      V      F      L      Q      L      Y      H      S      D       WKAIENLRAW 
  gi|49185553|BAS2545|YP_0    42  2.56e-09 
    IWIIKEQTVA     E      E      L      A      Q      E      V      F      L      Q      L      Y      H      S      D       WKAIENLRAW 
  gi|52142774|BCZK2466|YP_    42  2.56e-09 
    IWIIKEQTVA     E      E      L      A      Q      E      V      F      L      Q      L      Y      H      S      D       WKSIENLRAW 
  gi|118478099|BALH_2454|Y    42  2.56e-09 
    IWIIKEQTVA     E      E      L      A      Q      E      V      F      L      Q      L      Y      H      S      D       WKAIENLRAW 
  gi|152977601|Bcer98_3939    42  2.56e-09 
    LWIVKNQPIA     E      E      L      A      Q      E      V      F      L      Q      L      Y      H      S      D       WKAIENIPGW 
  gi|218903856|BCAH820_274    42  2.56e-09 
    IWIIKEQTVA     E      E      L      A      Q      E      V      F      L      Q      L      Y      H      S      D       WKAIENLCAW 
  gi|229603088|BAA_2796|YP    42  2.56e-09 
    IWIIKEQTVA     E      E      L      A      Q      E      V      F      L      Q      L      Y      H      S      D       WKAIENLRAW 
  gi|47528023|GBAA2732|YP_    42  2.56e-09 
    IWIIKEQTVA     E      E      L      A      Q      E      V      F      L      Q      L      Y      H      S      D       WKAIENLRAW 
  gi|49477843|BT9727_2501|    42  2.56e-09 
    IWIIKEQTVA     E      E      L      A      Q      E      V      F      L      Q      L      Y      H      S      D       WKAIENLRAW 
  gi|225864707|BCA_2814|YP    42  2.56e-09 
    IWIIKEQTVA     E      E      L      A      Q      E      V      F      L      Q      L      Y      H      S      D       WKAIENLRAW 
  gi|222096241|BCQ_2581|YP    42  2.56e-09 
    IWIIKEQTVA     E      E      L      A      Q      E      V      F      L      Q      L      Y      H      S      D       WKAIENLRAW 
  gi|301054256|BACI_c26970    42  2.56e-09 
    IWIIKEQTVA     E      E      L      A      Q      E      V      F      L      Q      L      Y      H      S      D       WKAIENLRAW 
  gi|261409947|GYMC10_6177    30  2.56e-09 
    LSLSRDEHVA     E      E      L      T      Q      E      T      F      F      K      A      L      K      H      I       DSFQGTCKLS 
  gi|157691378|BPUM_0588|Y    44  2.56e-09 
    FLYTRNKEDS     L      D      A      V      Q      E      T      V      I      K      A      F      K      H      L       SGLKEPAYFS 
  gi|172056180|Exig_0136|Y    46  2.56e-09 
    FRVLRDRMEA     E      D      A      T      Q      E      A      F      I      R      V      F      T      K      I       ESYNAQWKFR 
  gi|217957742|BCAH187_A02    34  3.09e-09 
    LAKGAIKEDA     E      D      I      I      Q      N      T      F      Y      K      V      Y      T      L      L       DDLTENNIRP 
  gi|229601254|BAA_0200|YP    33  3.09e-09 
    LAKGAIKEDA     E      D      I      I      Q      N      T      F      Y      K      V      Y      T      L      L       DDLTENNIRP 
  gi|52145050|BCZK0162|YP_    33  3.09e-09 
    LAKGAIKEDA     E      D      I      I      Q      N      T      F      Y      K      V      Y      T      L      L       DSLTEDNIRP 
  gi|49183204|BAS0171|YP_0    33  3.09e-09 
    LAKGAIKEDA     E      D      I      I      Q      N      T      F      Y      K      V      Y      T      L      L       DDLTENNIRP 
  gi|227812842|BAMEG_0200|    33  3.09e-09 
    LAKGAIKEDA     E      D      I      I      Q      N      T      F      Y      K      V      Y      T      L      L       DDLTENNIRP 
  gi|225862220|BCA_0212|YP    34  3.09e-09 
    LAKGAIKEDA     E      D      I      I      Q      N      T      F      Y      K      V      Y      T      L      L       DSLTEDNIRP 
  gi|163938169|BcerKBAB4_0    33  3.09e-09 
    LAKGAIKEDA     E      D      I      I      Q      N      T      F      Y      K      V      Y      T      L      L       DDLTEGNIRP 
  gi|222093937|BCQ_0193|YP    33  3.09e-09 
    LAKGAIKEDA     E      D      I      I      Q      N      T      F      Y      K      V      Y      T      L      L       DDLTENNIRP 
  gi|47525427|GBAA0169|YP_    33  3.09e-09 
    LAKGAIKEDA     E      D      I      I      Q      N      T      F      Y      K      V      Y      T      L      L       DDLTENNIRP 
  gi|30260357|BA0169|NP_84    33  3.09e-09 
    LAKGAIKEDA     E      D      I      I      Q      N      T      F      Y      K      V      Y      T      L      L       DDLTENNIRP 
  gi|218901372|BCAH820_019    33  3.09e-09 
    LAKGAIKEDA     E      D      I      I      Q      N      T      F      Y      K      V      Y      T      L      L       DDLTENNIRP 
  gi|118475937|BALH_0169|Y    34  3.09e-09 
    LAKGAIKEDA     E      D      I      I      Q      N      T      F      Y      K      V      Y      T      L      L       DNLTEDNIRP 
  gi|169829988|Bsph_4570|Y    46  3.09e-09 
    YRMLGNKQES     E      D      I      A      Q      E      A      F      V      R      A      Y      M      N      L       HTFDQKRKFS 
  gi|42779274|BCE_0193|NP_    33  3.09e-09 
    LAKGAIKEDA     E      D      I      I      Q      N      T      F      Y      K      V      Y      T      L      L       DSLTEDNIRP 
  gi|288556517|BpOF4_17595    33  3.09e-09 
    LVRGAVKEDA     E      D      I      I      Q      N      T      F      Y      K      V      Y      T      L      L       DDLTESNLRP 
  gi|169827960|Bsph_2435|Y    35  3.09e-09 
    VHQTKNMSEA     E      D      I      V      Q      D      V      F      M      T      L      M      K      Q      R       KPFDHEDHLK 
  gi|261404968|GYMC10_1112    49  3.40e-09 
    YSVLGDFHEA     Q      D      A      V      Q      E      A      F      L      K      C      Y      R      N      L       HTLNDPAKLG 
  gi|261406319|GYMC10_2476    114  3.73e-09 
    LYVVRDHAAV     E      D      I      I      Q      E      S      F      I      K      V      I      T      S      K       PKFETESKMR 
  gi|218896046|BCG9842_B42    42  4.10e-09 
    IWIVKDQSIA     E      E      L      A      Q      E      V      F      L      Q      L      Y      R      S      D       WKGIENIPGW 
  gi|218233874|BCB4264_A10    42  4.10e-09 
    IWIVKDQSIA     E      E      L      A      Q      E      V      F      L      Q      L      Y      R      S      D       WKGIENIPGW 
  gi|163941054|BcerKBAB4_3    42  4.10e-09 
    IWIVKDQSIA     E      E      L      A      Q      E      V      F      L      Q      L      Y      R      S      D       WKVIENIPGW 
  gi|296501706|BMB171_C086    42  4.10e-09 
    IWIVKDQSIA     E      E      L      A      Q      E      V      F      L      Q      L      Y      R      S      D       WKGIKNIPGW 
  gi|218232506|BCB4264_A54    31  4.49e-09 
    YKLAKTKEDA     E      D      I      L      Q      E      T      W      I      K      V      F      S      S      R       HQLSYVDKRV 
  gi|296506459|BMB171_P007    97  4.49e-09 
    YKNKHPDIEF     D      D      L      V      Q      E      G      N      E      G      M      L      R      A      M       EDFNPDLGYC 
  gi|67078088|pE33L466_021    97  4.49e-09 
    YKNKHPDIEF     D      D      L      V      Q      E      G      N      E      G      M      L      R      A      M       EDFNPDLGYC 
  gi|261406207|GYMC10_2363    43  5.39e-09 
    YFLTKQHALA     D      D      I      A      Q      E      A      F      I      K      A      Y      Y      G      I       HTFRGQATLK 
  gi|294498777|BMQ_2014|YP    47  6.46e-09 
    YRLTNSSDIA     E      E      A      V      Q      E      V      F      I      K      L      W      R      G      K       GVYSDGKGKF 
  gi|294497028|BMQ_0193|YP    46  6.46e-09 
    YRMLGNREEA     Q      D      A      A      Q      E      A      F      I      R      A      Y      V      N      I       HSYDTSKKFS 
  gi|295704098|BMD_1970|YP    47  6.46e-09 
    YRLTNSSDIA     E      E      A      V      Q      E      V      F      I      K      L      W      R      G      K       GVYSDEKGKF 
  gi|295702393|BMD_0187|YP    46  6.46e-09 
    YRMLGNREEA     Q      D      A      A      Q      E      A      F      I      R      A      Y      V      N      I       HSYDTSKKFS 
  gi|255767497|BSU23100|NP    31  7.72e-09 
    FYMVKDKNQT     E      D      L      L      Q      E      V      Y      I      R      V      L      N      S      Y       HTFEGRSSEK 
  gi|157691562|BPUM_0780|Y    48  7.72e-09 
    YKMTGSRELA     E      E      V      L      Q      E      V      F      I      K      L      W      R      G      I       GEYHTDKGKF 
  gi|294509114|BMQ_pBM5002    42  7.72e-09 
    VWIVKNQMIA     E      E      L      A      Q      E      V      F      L      Q      L      Y      R      N      D       WKSIENVPGW 
  gi|227813117|BAMEG_0515|    54  8.44e-09 
    AIEQETNLNM     D      D      L      I      Q      S      G      M      L      G      L      V      T      A      R       RDFKTELGFK 
  gi|229600567|BAA_4139|YP    54  8.44e-09 
    AIEQETNLNM     D      D      L      I      Q      S      G      M      L      G      L      V      T      A      R       RDFKTELGFK 
  gi|47529410|GBAA4115|YP_    39  8.44e-09 
    AIEQETNLNM     D      D      L      I      Q      S      G      M      L      G      L      V      T      A      R       RDFKTELGFK 
  gi|30263977|BA4115|NP_84    39  8.44e-09 
    AIEQETNLNM     D      D      L      I      Q      S      G      M      L      G      L      V      T      A      R       RDFKTELGFK 
  gi|49186821|BAS3823|YP_0    54  8.44e-09 
    AIEQETNLNM     D      D      L      I      Q      S      G      M      L      G      L      V      T      A      R       RDFKTELGFK 
  gi|218906428|BCAH820_534    31  9.21e-09 
    IKKLGIYQDY     E      E      Y      Y      Q      C      G      L      I      G      L      W      Y      A      Y       ERYDAKKGSF 
  gi|261407370|GYMC10_3567    38  9.21e-09 
    FAITRNTHTA     D      D      V      A      Q      D      V      F      I      K      A      Y      Q      H      F       ASFRGEASVK 
  gi|15614178|BH1615|NP_24    43  9.21e-09 
    FQLGVSPFAV     E      D      I      S      Q      E      V      F      I      K      V      Y      R      F      L       HKHDRGKFTT 
  gi|154685892|RBAM_014590    33  1.19e-08 
    YRRTHHAETA     K      D      L      A      Q      D      T      F      M      K      A      F      N      G      L       DSFKGHSSIR 
  gi|261407639|GYMC10_3840    57  1.19e-08 
    RAITRESYLA     E      D      V      V      Q      D      A      L      I      R      A      F      M      H      L       GKLVDARRFL 
  gi|229917320|EAT1b_1595|    46  1.30e-08 
    YKILYDRGEA     E      D      A      A      Q      E      A      F      I      R      A      Y      T      R      I       DTYDAQYKFK 
  gi|56962021|ABC0239|YP_1    46  1.30e-08 
    YRMVGHVHEA     Q      D      I      A      Q      E      A      F      L      R      A      Y      T      N      L       DKFDTTRKFS 
  gi|23098724|OB1269|NP_69    36  1.42e-08 
    FNYVKDKETA     K      D      M      V      Q      E      T      F      I      K      C      Y      E      K      L       DEFRNESNLK 
  gi|15612826|BH0263|NP_24    46  1.54e-08 
    YRMVGNVHEA     Q      D      V      A      Q      E      A      F      L      R      A      Y      T      N      I       DTYDIQRKFS 
  gi|23100811|OB3356|NP_69    41  1.68e-08 
    YLLVKDHQLA     E      E      A      V      Q      D      T      F      I      T      A      F      E      K      I       HQLEDAAKLK 
  gi|15615679|BH3117|NP_24    29  1.68e-08 
    LFVTKSTWDA     E      D      L      A      Q      E      T      W      L      K      A      I      E      T      L       KKQGHQNPEA 
  gi|152977451|Bcer98_3783    31  1.82e-08 
    MKRLCLYKDY     E      E      F      Y      Q      C      G      L      I      G      L      W      M      A      Y       ERYEEEKGSF 
  gi|138894766|GTNG_1100|Y    56  1.82e-08 
    SATLPSSVPK     E      E      L      V      S      L      G      L      V      G      L      Y      D      A      L       EKFDPSRDLK 
  gi|294497918|BMQ_1151|YP    31  1.82e-08 
    IKKFHIYKDY     D      E      Y      Y      Q      I      A      L      I      G      L      W      R      A      Y       QKFDESKGSF 
  gi|295703274|BMD_1138|YP    31  1.82e-08 
    IKKFHIYKDY     D      E      Y      Y      Q      I      A      L      I      G      L      W      R      A      Y       QKFDENKGSF 
  gi|56963737|ABC1972|YP_1    44  1.82e-08 
    LKITLKPSLA     D      D      L      T      Q      E      T      M      T      K      A      I      E      K      I       HLYKGRSKFS 
  gi|261406097|GYMC10_2252    56  1.98e-08 
    WRLLNQRQDA     E      D      A      V      Q      E      I      M      V      K      A      Y      Q      K      L       ETYKPETSFS 
  gi|288554775|BpOF4_08800    46  2.15e-08 
    YRMVGNAHEA     Q      D      V      A      Q      E      A      F      L      R      A      Y      T      N      L       DRFDTNRKFS 
  gi|297582837|Bsel_0515|Y    31  2.15e-08 
    FYLVRHRETA     E      E      L      V      Q      E      V      Y      I      K      V      L      N      S      F       ETFEGKSTEK 
  gi|56419781|GK1246|YP_14    54  2.54e-08 
    SATLPSSVPK     D      E      L      V      S      L      G      L      V      G      L      Y      D      A      L       EKFDPSRDLK 
  gi|261419446|GYMC61_2030    54  2.54e-08 
    SATLPSSVPK     D      E      L      V      S      L      G      L      V      G      L      Y      D      A      L       EKFDPSRDLK 
  gi|52080075|BL02968|YP_0    35  2.54e-08 
    YRRTHQLETA     K      D      L      A      Q      D      T      F      L      K      A      F      A      G      L       ESFRGHSSIK 
  gi|52785449|BLi01689|YP_    35  2.54e-08 
    YRRTHQLETA     K      D      L      A      Q      D      T      F      L      K      A      F      A      G      L       ESFRGHSSIK 
  gi|218848158|BCG9842_003    72  2.54e-08 
    SFPCYGNCDL     M      E      M      I      Q      E      G      T      I      G      L      M      K      G      L       EDYDYTKGNK 
  gi|297530579|GC56T3_2307    54  2.54e-08 
    SATLPSSVPK     D      E      L      V      S      L      G      L      V      G      L      Y      D      A      L       EKFDPSRDLK 
  gi|49185294|BAS2285|YP_0    29  2.75e-08 
    YRILGSVMDA     E      D      I      V      Q      D      V      F      I      S      L      N      N      I      E       NLQSIENMKA 
  gi|227814737|BAMEG_2147|    29  2.75e-08 
    YRILGSVMDA     E      D      I      V      Q      D      V      F      I      S      L      N      N      I      E       NLQSIENMKA 
  gi|30020517|BC2386|NP_83    29  2.75e-08 
    YRILGSVMDA     E      D      I      V      Q      D      V      F      I      S      L      N      N      I      E       DPQSIENMKA 
  gi|30262453|BA2454|NP_84    29  2.75e-08 
    YRILGSVMDA     E      D      I      V      Q      D      V      F      I      S      L      N      N      I      E       NLQSIENMKA 
  gi|47527745|GBAA2454|YP_    29  2.75e-08 
    YRILGSVMDA     E      D      I      V      Q      D      V      F      I      S      L      N      N      I      E       NLQSIENMKA 
  gi|52143032|BCZK2206|YP_    29  2.75e-08 
    YRILGSVMDA     E      D      I      V      Q      D      V      F      I      S      L      N      N      I      E       DLQSIENMKA 
  gi|49477707|BT9727_2248|    29  2.75e-08 
    YRILGSVMDA     E      D      I      V      Q      D      V      F      I      S      L      N      N      I      E       DFQSIENMKA 
  gi|217959947|BCAH187_A25    29  2.75e-08 
    YRILGSVMDA     E      D      I      V      Q      D      V      F      I      S      L      N      N      I      E       DLQSIENMKA 
  gi|118477845|BALH_2188|Y    29  2.75e-08 
    YRILGSVMDA     E      D      I      V      Q      D      V      F      I      S      L      N      N      I      E       DLQSIENMKA 
  gi|218233483|BCB4264_A24    29  2.75e-08 
    YRILGSVMDA     E      D      I      V      Q      D      V      F      I      S      L      N      N      I      E       DPQSIENMKA 
  gi|225864413|BCA_2518|YP    29  2.75e-08 
    YRILGSVMDA     E      D      I      V      Q      D      V      F      I      S      L      N      N      I      E       DLQSIENMKA 
  gi|218903588|BCAH820_247    29  2.75e-08 
    YRILGSVMDA     E      D      I      V      Q      D      V      F      I      S      L      N      N      I      E       DFQSIENMKA 
  gi|222096033|BCQ_2373|YP    29  2.75e-08 
    YRILGSVMDA     E      D      I      V      Q      D      V      F      I      S      L      N      N      I      E       DLQSIENMKA 
  gi|229601988|BAA_2511|YP    29  2.75e-08 
    YRILGSVMDA     E      D      I      V      Q      D      V      F      I      S      L      N      N      I      E       NLQSIENMKA 
  gi|261405039|GYMC10_1184    29  2.75e-08 
    RSIAKHAYEA     D      D      L      V      Q      D      A      F      M      K      A      I      K      E      P       NLAELPLHKQ 
  gi|16080921|BSU38700|NP_    47  2.75e-08 
    LKLSLHPDLS     E      E      L      V      Q      E      T      F      L      K      G      Y      I      H      L       RSFQGRSKFS 
  gi|296502978|BMB171_C214    29  2.75e-08 
    YRILGSVMDA     E      D      I      V      Q      D      V      F      I      S      L      N      N      I      E       DPQSIENMKA 
  gi|301053967|BACI_c23980    29  2.75e-08 
    YRILGSVMDA     E      D      I      V      Q      D      V      F      I      S      L      N      N      I      E       DLQSIENMKA 
  gi|16078537|BSU14730|NP_    33  2.98e-08 
    FRRTHHLETA     K      D      L      A      Q      D      T      F      V      K      A      L      N      G      L       ASFRGHSSIR 
  gi|172056208|Exig_0164|Y    48  2.98e-08 
    YVYVKNEQDA     L      D      V      V      Q      E      T      V      Y      K      A      F      I      S      I       GQVKEPKYFV 
  gi|15615785|BH3223|NP_24    39  2.98e-08 
    KRLTHNKWES     E      D      L      F      Q      D      S      L      I      K      I      Y      L      A      M       KQQPDRKMTK 
  gi|23098753|OB1298|NP_69    32  3.50e-08 
    YQLSKSKQVA     E      D      L      T      Q      E      T      F      L      Q      A      T      I      H      L       HTFKDESVRS 
  gi|261406442|GYMC10_2604    45  3.50e-08 
    CRMLGCKQDA     Q      D      A      V      Q      D      I      F      I      K      A      F      T      K      L       YTYEAKVSFS 
  gi|297582696|Bsel_0370|Y    46  3.50e-08 
    YRMLGNMHES     Q      D      I      A      Q      E      A      F      L      R      A      Y      M      N      L       DSYDINRKFS 
  gi|229917220|EAT1b_1495|    44  3.50e-08 
    LQLTKDRDLA     E      E      A      M      Q      E      V      F      I      K      L      W      R      G      G       GVYDESKGKF 
  gi|212639788|Aflv_1962|Y    28  3.79e-08 
    IRKLHIRRHV     D      E      Y      V      Q      I      G      L      I      A      L      W      E      A      Y       ERYDETKGSF 
  gi|239827399|GWCH70_2031    598  3.79e-08 
    KKFSCETVPM     E      D      L      Y      Q      E      A      Y      F      A      L      H      K      A      Y       EKYNPELGGS 
  gi|297584063|Bsel_1770|Y    55  4.11e-08 
    GVHLPKNVSS     D      D      L      R      S      H      G      L      M      G      L      F      D      A      L       EKFDRKRELK 
  gi|157694260|BPUM_3514|Y    47  4.11e-08 
    MKLTLQQELA     E      E      I      M      Q      E      T      M      L      K      A      Y      M      K      L       SSFRGDSLFS 
  gi|49184672|BAS1658|YP_0    23  4.44e-08 
    FSILRHEEDA     K      D      V      T      Q      E      V      F      V      K      I      H      A      S      L       PNYQFRGLKT 
  gi|163939640|BcerKBAB4_1    44  4.44e-08 
    FSILRHEEDA     K      D      V      T      Q      E      V      F      V      K      I      H      A      S      L       PNYQFRGLKT 
  gi|222095458|BCQ_1798|YP    23  4.44e-08 
    FSILRHEEDA     K      D      V      T      Q      E      V      F      V      K      I      H      A      S      L       PNYQFRGLKT 
  gi|42780931|BCE_1861|NP_    23  4.44e-08 
    FSILRHEEDA     K      D      V      T      Q      E      V      F      V      K      I      H      A      S      L       PNYQFRGLKT 
  gi|217959319|BCAH187_A19    23  4.44e-08 
    FSILRHEEDA     K      D      V      T      Q      E      V      F      V      K      I      H      A      S      L       PNYQFRGLKT 
  gi|47527079|GBAA1789|YP_    23  4.44e-08 
    FSILRHEEDA     K      D      V      T      Q      E      V      F      V      K      I      H      A      S      L       PNYQFRGLKT 
  gi|225863696|BCA_1798|YP    23  4.44e-08 
    FSILRHEEDA     K      D      V      T      Q      E      V      F      V      K      I      H      A      S      L       PNYQFRGLKT 
  gi|30261838|BA1789|NP_84    23  4.44e-08 
    FSILRHEEDA     K      D      V      T      Q      E      V      F      V      K      I      H      A      S      L       PNYQFRGLKT 
  gi|218902955|BCAH820_183    23  4.44e-08 
    FSILRHEEDA     K      D      V      T      Q      E      V      F      V      K      I      H      A      S      L       PNYQFRGLKT 
  gi|49481088|BT9727_1636|    23  4.44e-08 
    FSILRHEEDA     K      D      V      T      Q      E      V      F      V      K      I      H      A      S      L       PNYQFRGLKT 
  gi|218896772|BCG9842_B35    23  4.44e-08 
    FSIVRHEEDA     K      D      V      T      Q      E      V      F      V      K      I      H      A      S      L       PNYQFRGLKT 
  gi|227815388|BAMEG_2801|    23  4.44e-08 
    FSILRHEEDA     K      D      V      T      Q      E      V      F      V      K      I      H      A      S      L       PNYQFRGLKT 
  gi|118477262|BALH_1573|Y    23  4.44e-08 
    FSILRHEEDA     K      D      V      T      Q      E      V      F      V      K      I      H      A      S      L       PNYQFRGLKT 
  gi|229602836|BAA_1861|YP    23  4.44e-08 
    FSILRHEEDA     K      D      V      T      Q      E      V      F      V      K      I      H      A      S      L       PNYQFRGLKT 
  gi|52143630|BCZK1605|YP_    23  4.44e-08 
    FSILRHEEDA     K      D      V      T      Q      E      V      F      V      K      I      H      A      S      L       PNYQFRGLKT 
  gi|301053373|BACI_c17850    23  4.44e-08 
    FSILRHEEDA     K      D      V      T      Q      E      V      F      V      K      I      H      A      S      L       PNYQFRGLKT 
  gi|295696140|Btus_1522|Y    37  4.44e-08 
    LKSRFKPAAM     E      D      L      V      S      F      G      M      L      G      L      L      E      A      A       RRYDPGQETP 
  gi|295694973|Btus_0293|Y    36  4.80e-08 
    YFYVRDRHMA     E      D      V      F      Q      D      T      F      L      R      V      Y      S      E      M       DKLRDEGAAK 
  gi|163942349|BcerKBAB4_4    32  5.61e-08 
    IKIGADRKEA     E      D      I      V      Q      D      T      L      Y      K      A      L      S      L      M       EEIPLEHLTP 
  gi|138894925|GTNG_1263|Y    39  5.61e-08 
    YFKVQNREEA     E      D      I      T      Q      E      T      Y      V      R      A      I      P      Y      L       QKNRVPPDKQ 
  gi|261407955|GYMC10_4162    56  6.06e-08 
    AIGLPKNVSK     G      D      L      A      S      N      G      V      M      G      L      I      D      A      I       EKFDYKRGLQ 
  gi|295696987|Btus_2417|Y    34  6.54e-08 
    YMVVRDTHLA     Q      D      V      V      Q      E      A      W      I      K      I      W      R      Q      I       KTLREPDRLG 
  gi|218905848|BCAH820_473    32  7.06e-08 
    IKIGANRKEA     E      D      I      V      Q      D      T      L      Y      K      A      L      L      L      M       EEIPLEHLTP 
  gi|217962100|BCAH187_A47    32  7.06e-08 
    IKIGANRKEA     E      D      I      V      Q      D      T      L      Y      K      A      L      L      L      M       EEIPLEHLTP 
  gi|49478708|BT9727_4352|    32  7.06e-08 
    IKIGANRKEA     E      D      I      V      Q      D      T      L      Y      K      A      L      L      L      M       EEIPLEHLTP 
  gi|222098082|BCQ_4424|YP    32  7.06e-08 
    IKIGANRKEA     E      D      I      V      Q      D      T      L      Y      K      A      L      L      L      M       EEIPLEHLTP 
  gi|225866598|BCA_4732|YP    32  7.06e-08 
    IKIGANRKEA     E      D      I      V      Q      D      T      L      Y      K      A      L      L      L      M       EEIPLEHLTP 
  gi|118479762|BALH_4199|Y    32  7.06e-08 
    IKIGADRKEA     E      D      I      V      Q      D      T      L      Y      K      A      L      L      L      M       EEIPLEHLTP 
  gi|294500913|BMQ_4167|YP    54  7.06e-08 
    SVGLPKNIHK     E      E      L      R      S      L      G      L      I      G      L      Y      D      A      L       EKFDYGRDLK 
  gi|295706259|BMD_4154|YP    54  7.06e-08 
    SVGLPKNIHK     E      E      L      R      S      L      G      L      I      G      L      Y      D      A      L       EKFDYGRDLK 
  gi|295705020|BMD_2904|YP    46  7.06e-08 
    LKLTLDEDLC     S      E      L      C      Q      E      A      M      M      K      C      Y      V      N      L       SSYKNEAKFS 
  gi|294499631|BMQ_2875|YP    46  7.06e-08 
    LKLTLDEDLC     S      E      L      C      Q      E      A      M      M      K      C      Y      V      N      L       SSYKNEAKFS 
  gi|169827296|Bsph_1728|Y    28  7.61e-08 
    IYLVKNRTLA     E      D      L      A      H      E      V      Y      V      R      V      L      R      S      Y       DQFAGNSSEK 
  gi|169826467|Bsph_0879|Y    31  9.53e-08 
    LKLTRNNVEA     E      D      L      M      Q      E      V      W      V      K      V      V      R      Y      E       DSVAEVEHIK 
  gi|23100228|OB2773|NP_69    32  9.53e-08 
    LHQMQIDDKD     E      E      Y      F      Q      E      G      L      V      A      M      W      K      A      F       ENYQPDKGPL 
  gi|295695225|Btus_0554|Y    45  1.11e-07 
    LAIVRNPEEA     A      D      V      A      Q      E      S      F      V      R      A      F      V      S      L       RSLKDDGAFP 
  gi|261409914|GYMC10_6143    42  1.11e-07 
    YDKLQDSHLA     E      D      A      V      Q      E      A      F      T      E      A      F      A      N      L       HKLQDPRRFP 
  gi|295705907|BMD_3799|YP    41  1.28e-07 
    RFLAQNAWDG     D      D      I      A      Q      E      T      F      L      K      A      Q      R      Y      K       GEEHKLSSAL 
  gi|294500554|BMQ_3807|YP    44  1.28e-07 
    RFLAQNVWDG     D      D      I      A      Q      E      T      F      L      K      A      Q      R      Y      K       SEEHNLSSAL 
  gi|239826640|GWCH70_1138    54  1.28e-07 
    AASLPKTISK     Q      E      L      I      S      L      G      L      M      G      L      Y      D      A      L       EKFDPSRDLK 
  gi|172058539|Exig_2532|Y    47  1.38e-07 
    HRFTNNDRLS     E      E      V      I      Q      E      V      W      M      K      I      W      N      G      R       VDFNTDKGKF 
  gi|297582728|Bsel_0402|Y    41  1.59e-07 
    VLLVKDRQLA     E      E      V      V      Q      D      T      F      I      T      A      F      R      K      S       HQLSSNDKIK 
  gi|23099037|OB1582|NP_69    54  1.83e-07 
    ASHLPNNVVK     D      D      I      R      S      F      G      I      L      G      L      Y      D      A      L       KKFDPSRDLK 
  gi|56965809|ABC4051|YP_1    44  1.83e-07 
    MKYTNQHNDA     L      D      A      I      Q      E      A      S      C      K      A      Y      L      S      I       HKLNHPQYFM 
  gi|157694452|BPUM_3710|Y    36  2.11e-07 
    YSYVKNPEDA     M      D      V      V      Q      E      A      I      H      K      A      L      K      S      V       HRLEDATKMH 
  gi|261405575|GYMC10_1726    44  2.11e-07 
    YYILNNEQDA     M      D      A      A      Q      E      A      L      I      R      V      Y      T      K      I       NSYEEKAQFK 
  gi|261409324|GYMC10_5549    57  2.11e-07 
    KIARNRPDLY     E      D      L      Y      Q      V      G      Q      M      A      L      I      R      L      L       QQYDISLGIP 
  gi|297582431|Bsel_0096|Y    43  2.43e-07 
    YMKVGNRQDA     E      E      L      L      Q      D      V      F      V      K      M      V      R      N      L       AGFSGKSTFR 
  gi|56421959|GK3424|YP_14    56  2.60e-07 
    KMARSRPDFY     E      D      L      F      Q      I      G      R      L      S      F      L      R      L      L       DHYDPSQGAS 
  gi|261420836|GYMC61_3488    56  2.60e-07 
    KMARSRPDFY     E      D      L      F      Q      I      G      R      L      S      F      L      R      L      L       DHYDPKQGTS 
  gi|42780194|BCE_1118|NP_    42  2.60e-07 
    KARLKNEEDI     G      D      A      I      Q      E      T      I      L      S      A      F      T      N      L       KKVKEPGYFK 
  gi|261404205|GYMC10_0333    38  2.60e-07 
    MVYLGNKQDV     E      E      A      I      Q      E      T      F      I      K      L      I      Y      K      A       PDFRDQEHKK 
  gi|297531625|GC56T3_3410    56  2.60e-07 
    KMARSRPDFY     E      D      L      F      Q      I      G      R      L      S      F      L      R      L      L       DHYDPKQGTS 
  gi|169826213|Bsph_0618|Y    44  2.60e-07 
    FQCGVHSNDL     A      D      V      S      Q      E      V      F      V      K      L      Y      R      F      L       HQFKQDRFTT 
  gi|138897001|GTNG_3372|Y    58  2.79e-07 
    KMARSRPDFY     E      D      L      F      Q      V      G      R      L      S      F      L      R      L      L       EHYDPNQGTS 
  gi|47530207|GBAA4913|YP_    48  2.99e-07 
    YRFNRDEQLS     I      D      L      V      Q      D      T      F      L      T      L      E      R      K      K       HMYEPEKGKI 
  gi|222098134|BCQ_4476|YP    48  2.99e-07 
    YRFNRDEQLS     I      D      L      V      Q      D      T      F      L      T      L      E      R      K      K       HMYEPEKGKI 
  gi|218233191|BCB4264_A47    38  2.99e-07 
    YRFNRDEQLS     I      D      L      V      Q      D      T      F      L      T      L      E      R      K      K       HMYELEKGKI 
  gi|30264734|BA4913|NP_84    48  2.99e-07 
    YRFNRDEQLS     I      D      L      V      Q      D      T      F      L      T      L      E      R      K      K       HMYEPEKGKI 
  gi|42783844|BCE_4798|NP_    48  2.99e-07 
    YRFNRDEQLS     I      D      L      V      Q      D      T      F      L      T      L      E      R      K      K       HMYELEKGKI 
  gi|217962148|BCAH187_A47    38  2.99e-07 
    YRFNRDEQLS     I      D      L      V      Q      D      T      F      L      T      L      E      R      K      K       HMYELEKGKI 
  gi|227817453|BAMEG_4945|    38  2.99e-07 
    YRFNRDEQLS     I      D      L      V      Q      D      T      F      L      T      L      E      R      K      K       HMYEPEKGKI 
  gi|52140841|BCZK4409|YP_    38  2.99e-07 
    YRFNRDEQLS     I      D      L      V      Q      D      T      F      L      T      L      E      R      K      K       HMYELEKGKI 
  gi|218905890|BCAH820_477    38  2.99e-07 
    YRFNRDEQLS     I      D      L      V      Q      D      T      F      L      T      L      E      R      K      K       HMYELEKGKI 
  gi|229604251|BAA_4924|YP    48  2.99e-07 
    YRFNRDEQLS     I      D      L      V      Q      D      T      F      L      T      L      E      R      K      K       HMYEPEKGKI 
  gi|49187552|BAS4558|YP_0    48  2.99e-07 
    YRFNRDEQLS     I      D      L      V      Q      D      T      F      L      T      L      E      R      K      K       HMYEPEKGKI 
  gi|118479803|BALH_4240|Y    48  2.99e-07 
    YRFNRDEQLS     I      D      L      V      Q      D      T      F      L      T      L      E      R      K      K       HMYELEKGKI 
  gi|301056170|BACI_c46590    48  2.99e-07 
    YRFNRDEQLS     I      D      L      V      Q      D      T      F      L      T      L      E      R      K      K       HMYELEKGKI 
  gi|49478801|BT9727_4602|    44  3.43e-07 
    YSYVRNEQDA     L      D      I      Y      Q      E      T      I      Y      E      A      Y      L      S      L       KKLKNPEKFQ 
  gi|163942610|BcerKBAB4_4    44  3.43e-07 
    YSYVRNEQDA     L      D      I      Y      Q      E      T      I      Y      E      A      Y      L      S      L       RKLKKPEKFQ 
  gi|295694956|Btus_0276|Y    45  3.43e-07 
    QRLLGRTEPA     E      D      L      V      Q      E      T      F      F      R      F      V      R      E      V       QRGRVPERVR 
  gi|229917493|EAT1b_1768|    42  3.43e-07 
    YVYVKNPDDA     L      D      V      V      Q      E      V      A      Y      Q      A      F      K      R      I       GTLREPQFLK 
  gi|169827905|Bsph_2380|Y    30  3.67e-07 
    YLYVKNKATA     E      D      I      V      Q      D      V      F      I      A      F      Y      Q      K      Q       GQFRQQASLR 
  gi|229916805|EAT1b_1078|    30  3.92e-07 
    RQLHLHPNEV     D      D      A      A      Q      T      A      R      I      A      L      W      R      A      I       QDGKTLGKTY 
  gi|169828654|Bsph_3171|Y    25  3.92e-07 
    YSLTKNHSAA     E      D      L      L      Q      D      T      F      T      K      A      H      I      T      L       LGQDIQDIKP 
  gi|261407440|GYMC10_3639    27  4.20e-07 
    YQMTGTVTDA     E      D      A      V      Q      D      V      F      V      K      V      Q      S      L      P       IKQIREPKAY 
  gi|163940540|BcerKBAB4_2    31  4.80e-07 
    TFLTKNKWDG     E      D      L      A      Q      E      A      I      C      K      V      L      Q      K      Y       SEKEICITLL 
  gi|261409306|GYMC10_5531    50  4.80e-07 
    LKVTMDPMLA     E      D      T      A      Q      D      T      I      V      R      C      M      E      N      M       NRYNGTSSFS 
  gi|261404261|GYMC10_0389    29  5.13e-07 
    YYMLRNAQDA     E      D      L      C      H      D      V      F      V      T      V      F      R      Q      D       WRSVEHTRAW 
  gi|169829956|Bsph_4538|Y    9  5.13e-07 
    MLRNAQDA     E      D      L      C      H      D      V      F      V      T      V      F      R      Q      D       WKSIEHTRAW 
  gi|163943299|BcerKBAB4_5    48  5.85e-07 
    FAIVKNGHDT     N      E      I      V      N      E      V      Y      F      Q      L      W      K      S      I       SKYDQSRPFL 
  gi|152975676|Bcer98_1911    45  5.85e-07 
    MFLTKNKWDG     E      D      L      A      Q      E      A      V      C      K      V      L      Q      K      Y       KEEDICMTLL 
  gi|212639538|Aflv_1712|Y    44  5.85e-07 
    ASSLPKSVKK     E      D      L      K      S      L      A      L      L      G      L      Y      D      A      L       EKFDPSRDLK 
  gi|261404314|GYMC10_0444    37  6.24e-07 
    YSYVRNKEDA     L      D      I      V      Q      D      A      I      H      K      A      F      L      S      M       EKLKQTGSLK 
  gi|15613183|BH0620|NP_24    42  6.66e-07 
    FTYVRNEHDA     L      D      V      V      Q      E      A      V      V      K      G      Y      E      A      Y       DRIKHLDYFS 
  gi|288555440|BpOF4_12150    32  7.58e-07 
    LKMGAVKEDA     E      D      I      I      Q      E      T      A      Y      Q      F      L      L      Y      M       DSVTIENVEG 
  gi|294500314|BMQ_3558|YP    45  7.58e-07 
    KHLGTLKSYE     E      E      C      V      N      D      V      L      L      A      V      W      H      H      I       DRFDSEKNTF 
  gi|295705661|BMD_3546|YP    45  7.58e-07 
    KHLGTLKSYE     E      E      C      V      N      D      V      L      L      A      V      W      H      H      I       DRFDSEKNTF 
  gi|16079766|BSU27120|NP_    37  1.04e-06 
    FSYVKNQDDA     L      D      I      V      Q      E      S      I      K      K      A      L      S      S      V       ETVRNPETIK 
  gi|154685119|RBAM_006640    36  1.04e-06 
    YSYVKNKDDA     L      D      I      V      Q      E      S      I      Q      K      A      L      T      S      V       ESVKNPDVIK 
  gi|52079036|BL05045|YP_0    36  1.11e-06 
    YSYVKNQEDA     L      D      I      V      Q      E      S      I      K      K      A      L      D      S      V       DSVRNPDTIK 
  gi|52784408|BLi00595|YP_    36  1.11e-06 
    YSYVKNQEDA     L      D      I      V      Q      E      S      I      K      K      A      L      D      S      V       DSVRNPDTIK 
  gi|218900006|BCG9842_B02    48  1.42e-06 
    FAIVKNRHDT     N      E      I      V      N      E      I      Y      F      Q      L      W      K      S      I       STYDQSRPFL 
  gi|23098703|OB1248|NP_69    36  1.51e-06 
    IGLTKSDELA     D      D      I      I      Q      E      L      F      T      K      I      L      V      S      P       SKITEVNYMK 
  gi|169829522|Bsph_4082|Y    40  2.18e-06 
    LTNFRIYKDY     D      Y      Y      R      Q      V      A      T      I      A      V      W      R      A      W       LKADPAKGQL 
  gi|295705993|BMD_3885|YP    37  2.45e-06 
    FSYVKNQEDA     L      D      I      M      Q      E      S      I      K      K      A      L      I      S      I       DRIKDIQSIK 
  gi|261404792|GYMC10_0928    48  2.60e-06 
    RLYLRREEDC     A      D      A      V      Q      E      A      I      V      K      S      F      A      A      M       STLKQPAYFK 
  gi|239826131|GWCH70_0591    45  3.48e-06 
    FTILRSHHDA     E      D      A      I      Q      N      S      L      L      E      A      Y      R      T      I       SENKEIHHFS 
  gi|163940227|BcerKBAB4_2    29  4.14e-06 
    YRILGSVMDA     E      D      I      V      H      D      V      F      I      S      F      N      N      M      E       DVQSIENMRA 
  gi|288554248|BpOF4_06145    31  6.49e-06 
    LKKLNLYRNH     E      H      F      Y      Q      V      G      V      C      A      L      W      E      A      Y       RKFEEGKGSF 
  gi|15615778|BH3216|NP_24    48  8.08e-06 
    LNILKDHSEA     E      D      V      S      H      D      V      F      L      E      V      F      Q      K      I       EHYDATKGSV 
  gi|163939522|BcerKBAB4_1    52  1.00e-05 
    LSTPIEKQYV     D      D      C      Y      N      D      V      F      T      V      I      W      F      N      I       DQFDIEKGNL 
  gi|218234226|BCB4264_A16    50  1.00e-05 
    LSTPIEKQYV     D      D      C      Y      N      D      V      F      T      V      I      W      F      N      I       DQFDTEKGNL 
  gi|23099768|OB2313|NP_69    26  1.24e-05 
    NTYVKNSNDT     Y      D      V      I      Q      E      T      A      Y      R      T      Y      E      K      L       PELRNPQYFK 
  gi|169826679|Bsph_1097|Y    45  1.38e-05 
    YCYVRNEADA     L      D      V      F      Q      Q      T      V      L      L      A      I      E      S      V       HQLREPKYFT 
  gi|169829980|Bsph_4562|Y    47  3.96e-05 
    RHLNYQQQDY     E      E      C      L      D      D      V      L      L      S      I      W      N      N      I       HSFDPKKNSF 
  gi|15616444|BH3882|NP_24    29  5.04e-05 
    RTISRNEQEA     N      D      L      I      Q      D      A      M      E      K      S      L      R      C      T       ELLDWPRHKQ 
  gi|15614589|BH2026|NP_24    44  1.57e-04 
    LTYMGNPHDA     M      D      A      M      E      D      M      I      V      T      L      Y      E      T      I       DQLQKEDAFY 
  gi|49479940|BT9727_1587|    45  1.14e-03 
    THKVLLPVQN     E      G      L      I      E      E      C      V      N      D      I      F      L      S      I       WNNANKFHGE 
  gi|163940708|BcerKBAB4_2    14  1.68e-03 
    VELKEWLYKM     E      S      G      D      Q      E      A      F      R      V      I      Y      K      L      T       SKDIYRQ 
  gi|16079737|BSU26840|NP_    90  2.45e-03 
    VLHFNDSAEE     E      N      F      T      K      E      A      T      V      C      I      R      S      T      I       KRLPEKYREA 
 
    
      	Motif 1 block diagrams  
    
 
  Name Lowest p-value    Motifs
 
     gi|212637937|Aflv_0088|Y
  4.1e-16
    
    
   1
    
   
  
 
     gi|294496966|BMQ_0119|YP
  4.1e-16
    
    
   1
    
   
  
 
     gi|295705913|BMD_3805|YP
  4.1e-16
    
    
   1
    
   
  
 
     gi|294500560|BMQ_3813|YP
  4.1e-16
    
    
   1
    
   
  
 
     gi|295696440|Btus_1834|Y
  4.1e-16
    
    
   1
    
   
  
 
     gi|15612678|BH0115|NP_24
  4.1e-16
    
    
   1
    
   
  
 
     gi|169830054|Bsph_4638|Y
  4.1e-16
    
    
   1
    
   
  
 
     gi|261409537|GYMC10_5766
  4.1e-16
    
    
   1
    
   
  
 
     gi|295702333|BMD_0117|YP
  4.1e-16
    
    
   1
    
   
  
 
     gi|56418624|GK0089|YP_14
  5.5e-16
    
    
   1
    
   
  
 
     gi|56961915|ABC0133|YP_1
  5.5e-16
    
    
   1
    
   
  
 
     gi|288554708|BpOF4_08465
  5.5e-16
    
    
   1
    
   
  
 
     gi|138893768|GTNG_0089|Y
  5.5e-16
    
    
   1
    
   
  
 
     gi|297528465|GC56T3_0089
  5.5e-16
    
    
   1
    
   
  
 
     gi|261417590|GYMC61_0090
  5.5e-16
    
    
   1
    
   
  
 
     gi|16077166|BSU00980|NP_
  7.4e-16
    
    
   1
    
   
  
 
     gi|295694818|Btus_0134|Y
  7.4e-16
    
    
   1
    
   
  
 
     gi|52078593|BL03273|YP_0
  7.4e-16
    
    
   1
    
   
  
 
     gi|157690881|BPUM_0083|Y
  7.4e-16
    
    
   1
    
   
  
 
     gi|154684616|RBAM_001230
  7.4e-16
    
    
   1
    
   
  
 
     gi|52783954|BLi00116|YP_
  7.4e-16
    
    
   1
    
   
  
 
     gi|212638845|Aflv_1004|Y
  9.7e-16
    
    
   1
    
   
  
 
     gi|23097558|OB0103|NP_69
  9.7e-16
    
    
   1
    
   
  
 
     gi|239825676|GWCH70_0094
  1.3e-15
    
    
   1
    
   
  
 
     gi|172056123|Exig_0079|Y
  1.3e-15
    
    
   1
    
   
  
 
     gi|222093864|BCQ_0107|YP
  1.7e-15
    
    
   1
    
   
  
 
     gi|152973941|Bcer98_0088
  1.7e-15
    
    
   1
    
   
  
 
     gi|118475864|BALH_0093|Y
  1.7e-15
    
    
   1
    
   
  
 
     gi|47525348|GBAA0093|YP_
  1.7e-15
    
    
   1
    
   
  
 
     gi|217957669|BCAH187_A01
  1.7e-15
    
    
   1
    
   
  
 
     gi|218901296|BCAH820_010
  1.7e-15
    
    
   1
    
   
  
 
     gi|225862146|BCA_0122|YP
  1.7e-15
    
    
   1
    
   
  
 
     gi|42779174|BCE_0093|NP_
  1.7e-15
    
    
   1
    
   
  
 
     gi|30018364|BC0114|NP_82
  1.7e-15
    
    
   1
    
   
  
 
     gi|227812767|BAMEG_0109|
  1.7e-15
    
    
   1
    
   
  
 
     gi|52145123|BCZK0089|YP_
  1.7e-15
    
    
   1
    
   
  
 
     gi|218895229|BCG9842_B52
  1.7e-15
    
    
   1
    
   
  
 
     gi|30260284|BA0093|NP_84
  1.7e-15
    
    
   1
    
   
  
 
     gi|229601909|BAA_0109|YP
  1.7e-15
    
    
   1
    
   
  
 
     gi|163938101|BcerKBAB4_0
  1.7e-15
    
    
   1
    
   
  
 
     gi|218231038|BCB4264_A01
  1.7e-15
    
    
   1
    
   
  
 
     gi|49476711|BT9727_0090|
  1.7e-15
    
    
   1
    
   
  
 
     gi|49183127|BAS0093|YP_0
  1.7e-15
    
    
   1
    
   
  
 
     gi|169827020|Bsph_1444|Y
  1.7e-15
    
    
   1
    
   
  
 
     gi|296500928|BMB171_C009
  1.7e-15
    
    
   1
    
   
  
 
     gi|295696452|Btus_1846|Y
  1.7e-15
    
    
   1
    
   
  
 
     gi|261405674|GYMC10_1825
  1.7e-15
    
    
   1
    
   
  
 
     gi|301051831|BACI_c01200
  1.7e-15
    
    
   1
    
   
  
 
     gi|212639646|Aflv_1820|Y
  2.7e-15
    
    
   1
    
   
  
 
     gi|42782996|BCE_3948|NP_
  2.7e-15
    
    
   1
    
   
  
 
     gi|218233406|BCB4264_A40
  2.7e-15
    
    
   1
    
   
  
 
     gi|227813187|BAMEG_0585|
  2.7e-15
    
    
   1
    
   
  
 
     gi|163941643|BcerKBAB4_3
  2.7e-15
    
    
   1
    
   
  
 
     gi|152976264|Bcer98_2552
  2.7e-15
    
    
   1
    
   
  
 
     gi|56419663|GK1128|YP_14
  2.7e-15
    
    
   1
    
   
  
 
     gi|49478441|BT9727_3645|
  2.7e-15
    
    
   1
    
   
  
 
     gi|30263905|BA4042|NP_84
  2.7e-15
    
    
   1
    
   
  
 
     gi|217961324|BCAH187_A39
  2.7e-15
    
    
   1
    
   
  
 
     gi|229602177|BAA_4068|YP
  2.7e-15
    
    
   1
    
   
  
 
     gi|30021992|BC3903|NP_83
  2.7e-15
    
    
   1
    
   
  
 
     gi|218905033|BCAH820_391
  2.7e-15
    
    
   1
    
   
  
 
     gi|225865885|BCA_4007|YP
  2.7e-15
    
    
   1
    
   
  
 
     gi|47778237|GBAA4042|YP_
  2.7e-15
    
    
   1
    
   
  
 
     gi|222097349|BCQ_3689|YP
  2.7e-15
    
    
   1
    
   
  
 
     gi|49186753|BAS3754|YP_0
  2.7e-15
    
    
   1
    
   
  
 
     gi|16078597|BSU15330|NP_
  2.7e-15
    
    
   1
    
   
  
 
     gi|218899057|BCG9842_B12
  2.7e-15
    
    
   1
    
   
  
 
     gi|52141585|BCZK3662|YP_
  2.7e-15
    
    
   1
    
   
  
 
     gi|118479123|BALH_3533|Y
  2.7e-15
    
    
   1
    
   
  
 
     gi|297530705|GC56T3_2445
  2.7e-15
    
    
   1
    
   
  
 
     gi|301055394|BACI_c38590
  2.7e-15
    
    
   1
    
   
  
 
     gi|157692208|BPUM_1427|Y
  2.7e-15
    
    
   1
    
   
  
 
     gi|239826533|GWCH70_1031
  2.7e-15
    
    
   1
    
   
  
 
     gi|288553150|BpOF4_00620
  2.7e-15
    
    
   1
    
   
  
 
     gi|295706362|BMD_4257|YP
  2.7e-15
    
    
   1
    
   
  
 
     gi|56964115|ABC2350|YP_1
  2.7e-15
    
    
   1
    
   
  
 
     gi|138894663|GTNG_0993|Y
  2.7e-15
    
    
   1
    
   
  
 
     gi|23098931|OB1476|NP_69
  2.7e-15
    
    
   1
    
   
  
 
     gi|52785510|BLi01751|YP_
  2.7e-15
    
    
   1
    
   
  
 
     gi|154685949|RBAM_015160
  2.7e-15
    
    
   1
    
   
  
 
     gi|296504397|BMB171_C356
  2.7e-15
    
    
   1
    
   
  
 
     gi|294501013|BMQ_4269|YP
  2.7e-15
    
    
   1
    
   
  
 
     gi|15615117|BH2554|NP_24
  2.7e-15
    
    
   1
    
   
  
 
     gi|261419325|GYMC61_1901
  2.7e-15
    
    
   1
    
   
  
 
     gi|52080136|BL02256|YP_0
  2.7e-15
    
    
   1
    
   
  
 
     gi|288555973|BpOF4_14835
  3.5e-15
    
    
   1
    
   
  
 
     gi|15614101|BH1538|NP_24
  3.5e-15
    
    
   1
    
   
  
 
     gi|56963560|ABC1795|YP_1
  3.5e-15
    
    
   1
    
   
  
 
     gi|218905210|BCAH820_409
  7.2e-15
    
    
   1
    
   
  
 
     gi|217961566|BCAH187_A42
  7.2e-15
    
    
   1
    
   
  
 
     gi|52141420|BCZK3829|YP_
  7.2e-15
    
    
   1
    
   
  
 
     gi|42783188|BCE_4142|NP_
  7.2e-15
    
    
   1
    
   
  
 
     gi|47529588|GBAA4294|YP_
  7.2e-15
    
    
   1
    
   
  
 
     gi|227816852|BAMEG_4334|
  7.2e-15
    
    
   1
    
   
  
 
     gi|118479276|BALH_3690|Y
  7.2e-15
    
    
   1
    
   
  
 
     gi|225866059|BCA_4185|YP
  7.2e-15
    
    
   1
    
   
  
 
     gi|16079402|BSU23450|NP_
  7.2e-15
    
    
   1
    
   
  
 
     gi|218899234|BCG9842_B10
  7.2e-15
    
    
   1
    
   
  
 
     gi|30264150|BA4294|NP_84
  7.2e-15
    
    
   1
    
   
  
 
     gi|49478518|BT9727_3813|
  7.2e-15
    
    
   1
    
   
  
 
     gi|218234550|BCB4264_A41
  7.2e-15
    
    
   1
    
   
  
 
     gi|222097523|BCQ_3863|YP
  7.2e-15
    
    
   1
    
   
  
 
     gi|49186981|BAS3983|YP_0
  7.2e-15
    
    
   1
    
   
  
 
     gi|30022159|BC4072|NP_83
  7.2e-15
    
    
   1
    
   
  
 
     gi|163941815|BcerKBAB4_3
  7.2e-15
    
    
   1
    
   
  
 
     gi|152976482|Bcer98_2771
  7.2e-15
    
    
   1
    
   
  
 
     gi|229603990|BAA_4316|YP
  7.2e-15
    
    
   1
    
   
  
 
     gi|56420843|GK2308|YP_14
  7.2e-15
    
    
   1
    
   
  
 
     gi|157692844|BPUM_2076|Y
  7.2e-15
    
    
   1
    
   
  
 
     gi|52080862|BL00778|YP_0
  7.2e-15
    
    
   1
    
   
  
 
     gi|301055569|BACI_c40400
  7.2e-15
    
    
   1
    
   
  
 
     gi|296504567|BMB171_C373
  7.2e-15
    
    
   1
    
   
  
 
     gi|294501135|BMQ_4391|YP
  7.2e-15
    
    
   1
    
   
  
 
     gi|295706482|BMD_4377|YP
  7.2e-15
    
    
   1
    
   
  
 
     gi|52786234|BLi02495|YP_
  7.2e-15
    
    
   1
    
   
  
 
     gi|297529524|GC56T3_1195
  7.2e-15
    
    
   1
    
   
  
 
     gi|239827600|GWCH70_2250
  7.2e-15
    
    
   1
    
   
  
 
     gi|138895880|GTNG_2239|Y
  7.2e-15
    
    
   1
    
   
  
 
     gi|261417856|GYMC61_0374
  7.2e-15
    
    
   1
    
   
  
 
     gi|261405990|GYMC10_2143
  7.2e-15
    
    
   1
    
   
  
 
     gi|154686587|RBAM_021560
  7.2e-15
    
    
   1
    
   
  
 
     gi|229917375|EAT1b_1650|
  1.1e-13
    
    
   1
    
   
  
 
     gi|297582430|Bsel_0095|Y
  2.3e-13
    
    
   1
    
   
  
 
     gi|23098085|OB0630|NP_69
  6.5e-13
    
    
   1
    
   
  
 
     gi|295696453|Btus_1847|Y
  7.7e-13
    
    
   1
    
   
  
 
     gi|288555708|BpOF4_13500
  7.7e-13
    
    
   1
    
   
  
 
     gi|229603939|BAA_4584|YP
  1.1e-12
    
    
   1
    
   
  
 
     gi|49481469|BT9727_4074|
  1.1e-12
    
    
   1
    
   
  
 
     gi|49187232|BAS4236|YP_0
  1.1e-12
    
    
   1
    
   
  
 
     gi|222097784|BCQ_4125|YP
  1.1e-12
    
    
   1
    
   
  
 
     gi|56421070|GK2535|YP_14
  1.1e-12
    
    
   1
    
   
  
 
     gi|218905533|BCAH820_441
  1.1e-12
    
    
   1
    
   
  
 
     gi|227817116|BAMEG_4602|
  1.1e-12
    
    
   1
    
   
  
 
     gi|217961829|BCAH187_A44
  1.1e-12
    
    
   1
    
   
  
 
     gi|225866320|BCA_4450|YP
  1.1e-12
    
    
   1
    
   
  
 
     gi|163942095|BcerKBAB4_4
  1.1e-12
    
    
   1
    
   
  
 
     gi|218231149|BCB4264_A44
  1.1e-12
    
    
   1
    
   
  
 
     gi|218899507|BCG9842_B07
  1.1e-12
    
    
   1
    
   
  
 
     gi|118479504|BALH_3926|Y
  1.1e-12
    
    
   1
    
   
  
 
     gi|30022417|BC4336|NP_83
  1.1e-12
    
    
   1
    
   
  
 
     gi|152976769|Bcer98_3065
  1.1e-12
    
    
   1
    
   
  
 
     gi|30264411|BA4566|NP_84
  1.1e-12
    
    
   1
    
   
  
 
     gi|42783467|BCE_4421|NP_
  1.1e-12
    
    
   1
    
   
  
 
     gi|52141167|BCZK4084|YP_
  1.1e-12
    
    
   1
    
   
  
 
     gi|47529862|GBAA4566|YP_
  1.1e-12
    
    
   1
    
   
  
 
     gi|138896107|GTNG_2470|Y
  1.1e-12
    
    
   1
    
   
  
 
     gi|301055830|BACI_c43070
  1.1e-12
    
    
   1
    
   
  
 
     gi|261418447|GYMC61_0982
  1.1e-12
    
    
   1
    
   
  
 
     gi|294501329|BMQ_4591|YP
  1.1e-12
    
    
   1
    
   
  
 
     gi|56963382|ABC1617|YP_1
  1.1e-12
    
    
   1
    
   
  
 
     gi|239827809|GWCH70_2471
  1.1e-12
    
    
   1
    
   
  
 
     gi|157693076|BPUM_2309|Y
  1.1e-12
    
    
   1
    
   
  
 
     gi|295706676|BMD_4577|YP
  1.1e-12
    
    
   1
    
   
  
 
     gi|52081127|BL02107|YP_0
  1.1e-12
    
    
   1
    
   
  
 
     gi|261405539|GYMC10_1690
  1.1e-12
    
    
   1
    
   
  
 
     gi|295695806|Btus_1170|Y
  1.1e-12
    
    
   1
    
   
  
 
     gi|296504831|BMB171_C400
  1.1e-12
    
    
   1
    
   
  
 
     gi|297582861|Bsel_0539|Y
  1.1e-12
    
    
   1
    
   
  
 
     gi|15613848|BH1285|NP_24
  1.1e-12
    
    
   1
    
   
  
 
     gi|297529299|GC56T3_0955
  1.1e-12
    
    
   1
    
   
  
 
     gi|212638647|Aflv_0804|Y
  1.1e-12
    
    
   1
    
   
  
 
     gi|52786504|BLi02769|YP_
  1.1e-12
    
    
   1
    
   
  
 
     gi|288555607|BpOF4_12995
  1.1e-12
    
    
   1
    
   
  
 
     gi|255767354|BSU15320|NP
  1.3e-12
    
    
   1
    
   
  
 
     gi|227813186|BAMEG_0584|
  1.3e-12
    
    
   1
    
   
  
 
     gi|152976265|Bcer98_2553
  1.3e-12
    
    
   1
    
   
  
 
     gi|218899058|BCG9842_B12
  1.3e-12
    
    
   1
    
   
  
 
     gi|218905035|BCAH820_391
  1.3e-12
    
    
   1
    
   
  
 
     gi|30263906|BA4043|NP_84
  1.3e-12
    
    
   1
    
   
  
 
     gi|225865886|BCA_4008|YP
  1.3e-12
    
    
   1
    
   
  
 
     gi|47529336|GBAA4043|YP_
  1.3e-12
    
    
   1
    
   
  
 
     gi|212639647|Aflv_1821|Y
  1.3e-12
    
    
   1
    
   
  
 
     gi|52141583|BCZK3663|YP_
  1.3e-12
    
    
   1
    
   
  
 
     gi|42782997|BCE_3949|NP_
  1.3e-12
    
    
   1
    
   
  
 
     gi|217961326|BCAH187_A39
  1.3e-12
    
    
   1
    
   
  
 
     gi|222097350|BCQ_3690|YP
  1.3e-12
    
    
   1
    
   
  
 
     gi|229601447|BAA_4069|YP
  1.3e-12
    
    
   1
    
   
  
 
     gi|30021993|BC3904|NP_83
  1.3e-12
    
    
   1
    
   
  
 
     gi|162382776|BALH_3534|Y
  1.3e-12
    
    
   1
    
   
  
 
     gi|218234749|BCB4264_A40
  1.3e-12
    
    
   1
    
   
  
 
     gi|49186754|BAS3755|YP_0
  1.3e-12
    
    
   1
    
   
  
 
     gi|163941644|BcerKBAB4_3
  1.3e-12
    
    
   1
    
   
  
 
     gi|49478442|BT9727_3646|
  1.3e-12
    
    
   1
    
   
  
 
     gi|56419662|GK1127|YP_14
  1.3e-12
    
    
   1
    
   
  
 
     gi|294501014|BMQ_4270|YP
  1.3e-12
    
    
   1
    
   
  
 
     gi|157692207|BPUM_1426|Y
  1.3e-12
    
    
   1
    
   
  
 
     gi|15615119|BH2556|NP_24
  1.3e-12
    
    
   1
    
   
  
 
     gi|296504398|BMB171_C356
  1.3e-12
    
    
   1
    
   
  
 
     gi|52785509|BLi01750|YP_
  1.3e-12
    
    
   1
    
   
  
 
     gi|154685948|RBAM_015150
  1.3e-12
    
    
   1
    
   
  
 
     gi|52080135|BL02255|YP_0
  1.3e-12
    
    
   1
    
   
  
 
     gi|288553151|BpOF4_00625
  1.3e-12
    
    
   1
    
   
  
 
     gi|138894662|GTNG_0992|Y
  1.3e-12
    
    
   1
    
   
  
 
     gi|23098930|OB1475|NP_69
  1.3e-12
    
    
   1
    
   
  
 
     gi|239826532|GWCH70_1030
  1.3e-12
    
    
   1
    
   
  
 
     gi|56964116|ABC2351|YP_1
  1.3e-12
    
    
   1
    
   
  
 
     gi|297530706|GC56T3_2446
  1.3e-12
    
    
   1
    
   
  
 
     gi|295706363|BMD_4258|YP
  1.3e-12
    
    
   1
    
   
  
 
     gi|261419324|GYMC61_1900
  1.3e-12
    
    
   1
    
   
  
 
     gi|301055395|BACI_c38600
  1.3e-12
    
    
   1
    
   
  
 
     gi|229917800|EAT1b_2078|
  2e-12
    
    
   1
    
   
  
 
     gi|172058807|Exig_2804|Y
  2e-12
    
    
   1
    
   
  
 
     gi|227816484|BAMEG_3941|
  3.2e-12
    
    
   1
    
   
  
 
     gi|229603531|BAA_0728|YP
  3.2e-12
    
    
   1
    
   
  
 
     gi|42779792|BCE_0714|NP_
  3.2e-12
    
    
   1
    
   
  
 
     gi|52144667|BCZK0557|YP_
  3.2e-12
    
    
   1
    
   
  
 
     gi|47525925|GBAA0646|YP_
  3.2e-12
    
    
   1
    
   
  
 
     gi|255767573|BSU25200|NP
  3.2e-12
    
    
   1
    
   
  
 
     gi|218895700|BCG9842_B46
  3.2e-12
    
    
   1
    
   
  
 
     gi|218232155|BCB4264_A06
  3.2e-12
    
    
   1
    
   
  
 
     gi|30260800|BA0646|NP_84
  3.2e-12
    
    
   1
    
   
  
 
     gi|30018830|BC0647|NP_83
  3.2e-12
    
    
   1
    
   
  
 
     gi|49183638|BAS0613|YP_0
  3.2e-12
    
    
   1
    
   
  
 
     gi|163938569|BcerKBAB4_0
  3.2e-12
    
    
   1
    
   
  
 
     gi|118476332|BALH_0588|Y
  3.2e-12
    
    
   1
    
   
  
 
     gi|222094400|BCQ_0714|YP
  3.2e-12
    
    
   1
    
   
  
 
     gi|225862624|BCA_0684|YP
  3.2e-12
    
    
   1
    
   
  
 
     gi|218901842|BCAH820_070
  3.2e-12
    
    
   1
    
   
  
 
     gi|157693020|BPUM_2253|Y
  3.2e-12
    
    
   1
    
   
  
 
     gi|154686781|RBAM_023510
  3.2e-12
    
    
   1
    
   
  
 
     gi|163119552|BL03682|YP_
  3.2e-12
    
    
   1
    
   
  
 
     gi|52786447|BLi02712|YP_
  3.2e-12
    
    
   1
    
   
  
 
     gi|218232261|BCB4264_A44
  3.7e-12
    
    
   1
    
   
  
 
     gi|52141207|BCZK4042|YP_
  3.7e-12
    
    
   1
    
   
  
 
     gi|42783418|BCE_4372|NP_
  3.7e-12
    
    
   1
    
   
  
 
     gi|30264362|BA4515|NP_84
  3.7e-12
    
    
   1
    
   
  
 
     gi|218899458|BCG9842_B08
  3.7e-12
    
    
   1
    
   
  
 
     gi|218905429|BCAH820_431
  3.7e-12
    
    
   1
    
   
  
 
     gi|229601154|BAA_4536|YP
  3.7e-12
    
    
   1
    
   
  
 
     gi|30022370|BC4289|NP_83
  3.7e-12
    
    
   1
    
   
  
 
     gi|152976724|Bcer98_3019
  3.7e-12
    
    
   1
    
   
  
 
     gi|163942050|BcerKBAB4_4
  3.7e-12
    
    
   1
    
   
  
 
     gi|56421017|GK2482|YP_14
  3.7e-12
    
    
   1
    
   
  
 
     gi|212638699|Aflv_0856|Y
  3.7e-12
    
    
   1
    
   
  
 
     gi|118479464|BALH_3885|Y
  3.7e-12
    
    
   1
    
   
  
 
     gi|217961783|BCAH187_A44
  3.7e-12
    
    
   1
    
   
  
 
     gi|47529811|GBAA4515|YP_
  3.7e-12
    
    
   1
    
   
  
 
     gi|49187190|BAS4194|YP_0
  3.7e-12
    
    
   1
    
   
  
 
     gi|225866273|BCA_4403|YP
  3.7e-12
    
    
   1
    
   
  
 
     gi|227817068|BAMEG_4554|
  3.7e-12
    
    
   1
    
   
  
 
     gi|49481307|BT9727_4032|
  3.7e-12
    
    
   1
    
   
  
 
     gi|297584635|Bsel_2346|Y
  3.7e-12
    
    
   1
    
   
  
 
     gi|295706627|BMD_4528|YP
  3.7e-12
    
    
   1
    
   
  
 
     gi|297529352|GC56T3_1008
  3.7e-12
    
    
   1
    
   
  
 
     gi|261417657|GYMC61_0157
  3.7e-12
    
    
   1
    
   
  
 
     gi|56963455|ABC1690|YP_1
  3.7e-12
    
    
   1
    
   
  
 
     gi|239827752|GWCH70_2414
  3.7e-12
    
    
   1
    
   
  
 
     gi|229916320|EAT1b_0589|
  3.7e-12
    
    
   1
    
   
  
 
     gi|288555754|BpOF4_13730
  3.7e-12
    
    
   1
    
   
  
 
     gi|169829158|Bsph_3702|Y
  3.7e-12
    
    
   1
    
   
  
 
     gi|301055785|BACI_c42620
  3.7e-12
    
    
   1
    
   
  
 
     gi|172056869|Exig_0832|Y
  3.7e-12
    
    
   1
    
   
  
 
     gi|261407775|GYMC10_3981
  3.7e-12
    
    
   1
    
   
  
 
     gi|261405623|GYMC10_1774
  3.7e-12
    
    
   1
    
   
  
 
     gi|23099399|OB1944|NP_69
  3.7e-12
    
    
   1
    
   
  
 
     gi|294501280|BMQ_4542|YP
  3.7e-12
    
    
   1
    
   
  
 
     gi|138896056|GTNG_2419|Y
  3.7e-12
    
    
   1
    
   
  
 
     gi|15613939|BH1376|NP_24
  3.7e-12
    
    
   1
    
   
  
 
     gi|296504786|BMB171_C395
  3.7e-12
    
    
   1
    
   
  
 
     gi|297582645|Bsel_0319|Y
  5e-12
    
    
   1
    
   
  
 
     gi|294500199|BMQ_3443|YP
  6.7e-12
    
    
   1
    
   
  
 
     gi|294497070|BMQ_0235|YP
  7.7e-12
    
    
   1
    
   
  
 
     gi|295702435|BMD_0229|YP
  7.7e-12
    
    
   1
    
   
  
 
     gi|295695448|Btus_0783|Y
  8.9e-12
    
    
   1
    
   
  
 
     gi|218232670|BCB4264_A10
  1.2e-11
    
    
   1
    
   
  
 
     gi|218896055|BCG9842_B42
  1.2e-11
    
    
   1
    
   
  
 
     gi|225862940|BCA_1029|YP
  1.2e-11
    
    
   1
    
   
  
 
     gi|227816152|BAMEG_3579|
  1.2e-11
    
    
   1
    
   
  
 
     gi|229604478|BAA_1086|YP
  1.2e-11
    
    
   1
    
   
  
 
     gi|30261116|BA0992|NP_84
  1.2e-11
    
    
   1
    
   
  
 
     gi|47526271|GBAA0992|YP_
  1.2e-11
    
    
   1
    
   
  
 
     gi|118476614|BALH_0889|Y
  1.2e-11
    
    
   1
    
   
  
 
     gi|52144331|BCZK0896|YP_
  1.2e-11
    
    
   1
    
   
  
 
     gi|218902186|BCAH820_106
  1.2e-11
    
    
   1
    
   
  
 
     gi|30019159|BC1004|NP_83
  1.2e-11
    
    
   1
    
   
  
 
     gi|222094730|BCQ_1068|YP
  1.2e-11
    
    
   1
    
   
  
 
     gi|217958581|BCAH187_A11
  1.2e-11
    
    
   1
    
   
  
 
     gi|49480184|BT9727_0913|
  1.2e-11
    
    
   1
    
   
  
 
     gi|49183950|BAS0928|YP_0
  1.2e-11
    
    
   1
    
   
  
 
     gi|42780162|BCE_1086|NP_
  1.2e-11
    
    
   1
    
   
  
 
     gi|163938900|BcerKBAB4_0
  1.2e-11
    
    
   1
    
   
  
 
     gi|301052635|BACI_c10250
  1.2e-11
    
    
   1
    
   
  
 
     gi|296501716|BMB171_C087
  1.2e-11
    
    
   1
    
   
  
 
     gi|152976319|Bcer98_2607
  1.4e-11
    
    
   1
    
   
  
 
     gi|169829726|Bsph_4295|Y
  1.4e-11
    
    
   1
    
   
  
 
     gi|52784380|BLi00560|YP_
  1.6e-11
    
    
   1
    
   
  
 
     gi|52079008|BL02208|YP_0
  1.6e-11
    
    
   1
    
   
  
 
     gi|23099294|OB1839|NP_69
  1.6e-11
    
    
   1
    
   
  
 
     gi|169829306|Bsph_3856|Y
  2.3e-11
    
    
   1
    
   
  
 
     gi|163940286|BcerKBAB4_2
  2.6e-11
    
    
   1
    
   
  
 
     gi|255767137|BSU04730|NP
  3e-11
    
    
   1
    
   
  
 
     gi|154686834|RBAM_024040
  3e-11
    
    
   1
    
   
  
 
     gi|154684976|RBAM_005070
  3e-11
    
    
   1
    
   
  
 
     gi|157691239|BPUM_0446|Y
  3e-11
    
    
   1
    
   
  
 
     gi|42780207|BCE_1131|NP_
  3.4e-11
    
    
   1
    
   
  
 
     gi|217958624|BCAH187_A12
  3.4e-11
    
    
   1
    
   
  
 
     gi|222094769|BCQ_1107|YP
  3.4e-11
    
    
   1
    
   
  
 
     gi|163938949|BcerKBAB4_0
  3.4e-11
    
    
   1
    
   
  
 
     gi|52144286|BCZK0942|YP_
  3.4e-11
    
    
   1
    
   
  
 
     gi|23099454|OB1999|NP_69
  3.4e-11
    
    
   1
    
   
  
 
     gi|225864482|BCA_2587|YP
  3.9e-11
    
    
   1
    
   
  
 
     gi|118477905|BALH_2250|Y
  3.9e-11
    
    
   1
    
   
  
 
     gi|42781595|BCE_2534|NP_
  3.9e-11
    
    
   1
    
   
  
 
     gi|217959986|BCAH187_A25
  3.9e-11
    
    
   1
    
   
  
 
     gi|218903633|BCAH820_251
  3.9e-11
    
    
   1
    
   
  
 
     gi|52142997|BCZK2243|YP_
  3.9e-11
    
    
   1
    
   
  
 
     gi|218231851|BCB4264_A24
  3.9e-11
    
    
   1
    
   
  
 
     gi|30020599|BC2469|NP_83
  3.9e-11
    
    
   1
    
   
  
 
     gi|222096073|BCQ_2413|YP
  3.9e-11
    
    
   1
    
   
  
 
     gi|49479242|BT9727_2287|
  3.9e-11
    
    
   1
    
   
  
 
     gi|218897485|BCG9842_B28
  3.9e-11
    
    
   1
    
   
  
 
     gi|52787786|BLi04109|YP_
  3.9e-11
    
    
   1
    
   
  
 
     gi|301054038|BACI_c24720
  3.9e-11
    
    
   1
    
   
  
 
     gi|261409476|GYMC10_5703
  3.9e-11
    
    
   1
    
   
  
 
     gi|296503062|BMB171_C223
  3.9e-11
    
    
   1
    
   
  
 
     gi|52082396|BL00940|YP_0
  3.9e-11
    
    
   1
    
   
  
 
     gi|261405673|GYMC10_1824
  5e-11
    
    
   1
    
   
  
 
     gi|288554854|BpOF4_09205
  5e-11
    
    
   1
    
   
  
 
     gi|49476853|BT9727_0558|
  8.2e-11
    
    
   1
    
   
  
 
     gi|169827019|Bsph_1443|Y
  8.2e-11
    
    
   1
    
   
  
 
     gi|118476646|BALH_0922|Y
  1.3e-10
    
    
   1
    
   
  
 
     gi|30261155|BA1032|NP_84
  1.3e-10
    
    
   1
    
   
  
 
     gi|218902228|BCAH820_111
  1.3e-10
    
    
   1
    
   
  
 
     gi|225862980|BCA_1069|YP
  1.3e-10
    
    
   1
    
   
  
 
     gi|49477062|BT9727_0953|
  1.3e-10
    
    
   1
    
   
  
 
     gi|227816114|BAMEG_3540|
  1.3e-10
    
    
   1
    
   
  
 
     gi|47777889|GBAA1032|YP_
  1.3e-10
    
    
   1
    
   
  
 
     gi|218231966|BCB4264_A10
  1.3e-10
    
    
   1
    
   
  
 
     gi|218896095|BCG9842_B42
  1.3e-10
    
    
   1
    
   
  
 
     gi|229604778|BAA_1124|YP
  1.3e-10
    
    
   1
    
   
  
 
     gi|49183986|BAS0964|YP_0
  1.3e-10
    
    
   1
    
   
  
 
     gi|301052678|BACI_c10700
  1.3e-10
    
    
   1
    
   
  
 
     gi|296501752|BMB171_C091
  1.3e-10
    
    
   1
    
   
  
 
     gi|212638144|Aflv_0295|Y
  1.5e-10
    
    
   1
    
   
  
 
     gi|56962590|ABC0816|YP_1
  1.5e-10
    
    
   1
    
   
  
 
     gi|295696809|Btus_2226|Y
  1.5e-10
    
    
   1
    
   
  
 
     gi|16077241|BSU01730|NP_
  1.9e-10
    
    
   1
    
   
  
 
     gi|52784027|BLi00199|YP_
  1.9e-10
    
    
   1
    
   
  
 
     gi|154684695|RBAM_002260
  1.9e-10
    
    
   1
    
   
  
 
     gi|157690957|BPUM_0160|Y
  1.9e-10
    
    
   1
    
   
  
 
     gi|52078665|BL02699|YP_0
  1.9e-10
    
    
   1
    
   
  
 
     gi|261409365|GYMC10_5592
  1.9e-10
    
    
   1
    
   
  
 
     gi|217958232|BCAH187_A07
  2.1e-10
    
    
   1
    
   
  
 
     gi|52786190|BLi02449|YP_
  2.1e-10
    
    
   1
    
   
  
 
     gi|157692810|BPUM_2042|Y
  2.1e-10
    
    
   1
    
   
  
 
     gi|52080819|BL00651|YP_0
  2.1e-10
    
    
   1
    
   
  
 
     gi|239827551|GWCH70_2200
  2.6e-10
    
    
   1
    
   
  
 
     gi|49480428|BT9727_5044|
  2.9e-10
    
    
   1
    
   
  
 
     gi|49188198|BAS5212|YP_0
  2.9e-10
    
    
   1
    
   
  
 
     gi|218906540|BCAH820_545
  2.9e-10
    
    
   1
    
   
  
 
     gi|222097738|BCQ_4079|YP
  2.9e-10
    
    
   1
    
   
  
 
     gi|225867344|BCA_5512|YP
  2.9e-10
    
    
   1
    
   
  
 
     gi|227818128|BAMEG_5654|
  2.9e-10
    
    
   1
    
   
  
 
     gi|217962852|BCAH187_A55
  2.9e-10
    
    
   1
    
   
  
 
     gi|42784537|BCE_5492|NP_
  2.9e-10
    
    
   1
    
   
  
 
     gi|218900489|BCG9842_B54
  2.9e-10
    
    
   1
    
   
  
 
     gi|118480394|BALH_4859|Y
  2.9e-10
    
    
   1
    
   
  
 
     gi|52140200|BCZK5060|YP_
  2.9e-10
    
    
   1
    
   
  
 
     gi|30023393|BC5363|NP_83
  2.9e-10
    
    
   1
    
   
  
 
     gi|30265385|BA5610|NP_84
  2.9e-10
    
    
   1
    
   
  
 
     gi|229601723|BAA_5636|YP
  2.9e-10
    
    
   1
    
   
  
 
     gi|222098834|BCQ_5203|YP
  2.9e-10
    
    
   1
    
   
  
 
     gi|47530932|GBAA5610|YP_
  2.9e-10
    
    
   1
    
   
  
 
     gi|301056831|BACI_c53580
  2.9e-10
    
    
   1
    
   
  
 
     gi|296505785|BMB171_C495
  2.9e-10
    
    
   1
    
   
  
 
     gi|261406063|GYMC10_2217
  3.3e-10
    
    
   1
    
   
  
 
     gi|30019269|BC1114|NP_83
  3.7e-10
    
    
   1
    
   
  
 
     gi|30261223|BA1113|NP_84
  3.7e-10
    
    
   1
    
   
  
 
     gi|49184055|BAS1035|YP_0
  3.7e-10
    
    
   1
    
   
  
 
     gi|218896153|BCG9842_B41
  3.7e-10
    
    
   1
    
   
  
 
     gi|47526385|GBAA1113|YP_
  3.7e-10
    
    
   1
    
   
  
 
     gi|218902311|BCAH820_119
  3.7e-10
    
    
   1
    
   
  
 
     gi|229602397|BAA_1198|YP
  3.7e-10
    
    
   1
    
   
  
 
     gi|225863070|BCA_1159|YP
  3.7e-10
    
    
   1
    
   
  
 
     gi|163939015|BcerKBAB4_1
  3.7e-10
    
    
   1
    
   
  
 
     gi|227816040|BAMEG_3466|
  3.7e-10
    
    
   1
    
   
  
 
     gi|222094839|BCQ_1177|YP
  3.7e-10
    
    
   1
    
   
  
 
     gi|218232533|BCB4264_A11
  3.7e-10
    
    
   1
    
   
  
 
     gi|217958692|BCAH187_A12
  3.7e-10
    
    
   1
    
   
  
 
     gi|42780290|BCE_1215|NP_
  3.7e-10
    
    
   1
    
   
  
 
     gi|52144214|BCZK1013|YP_
  3.7e-10
    
    
   1
    
   
  
 
     gi|49477092|BT9727_1012|
  3.7e-10
    
    
   1
    
   
  
 
     gi|138893826|GTNG_0147|Y
  3.7e-10
    
    
   1
    
   
  
 
     gi|301052754|BACI_c11460
  3.7e-10
    
    
   1
    
   
  
 
     gi|15613092|BH0529|NP_24
  3.7e-10
    
    
   1
    
   
  
 
     gi|261404455|GYMC10_0586
  3.7e-10
    
    
   1
    
   
  
 
     gi|261408152|GYMC10_4361
  3.7e-10
    
    
   1
    
   
  
 
     gi|169827149|Bsph_1579|Y
  3.7e-10
    
    
   1
    
   
  
 
     gi|227815066|BAMEG_2477|
  4.1e-10
    
    
   1
    
   
  
 
     gi|49184977|BAS1966|YP_0
  4.1e-10
    
    
   1
    
   
  
 
     gi|229602619|BAA_2180|YP
  4.1e-10
    
    
   1
    
   
  
 
     gi|30262135|BA2114|NP_84
  4.1e-10
    
    
   1
    
   
  
 
     gi|30020245|BC2108|NP_83
  4.1e-10
    
    
   1
    
   
  
 
     gi|47527408|GBAA2114|YP_
  4.1e-10
    
    
   1
    
   
  
 
     gi|218232753|BCB4264_A21
  4.1e-10
    
    
   1
    
   
  
 
     gi|296502728|BMB171_C189
  4.1e-10
    
    
   1
    
   
  
 
     gi|157692327|BPUM_1546|Y
  4.6e-10
    
    
   1
    
   
  
 
     gi|261406105|GYMC10_2260
  4.6e-10
    
    
   1
    
   
  
 
     gi|295695216|Btus_0545|Y
  4.6e-10
    
    
   1
    
   
  
 
     gi|212637996|Aflv_0147|Y
  5.1e-10
    
    
   1
    
   
  
 
     gi|239825735|GWCH70_0154
  5.1e-10
    
    
   1
    
   
  
 
     gi|52785627|BLi01868|YP_
  5.1e-10
    
    
   1
    
   
  
 
     gi|52080250|BL01246|YP_0
  5.1e-10
    
    
   1
    
   
  
 
     gi|261406462|GYMC10_2625
  5.6e-10
    
    
   1
    
   
  
 
     gi|23097683|OB0228|NP_69
  5.6e-10
    
    
   1
    
   
  
 
     gi|218233984|BCB4264_A53
  6.3e-10
    
    
   1
    
   
  
 
     gi|225867222|BCA_5390|YP
  6.3e-10
    
    
   1
    
   
  
 
     gi|30023283|BC5251|NP_83
  6.3e-10
    
    
   1
    
   
  
 
     gi|227814691|BAMEG_2101|
  6.3e-10
    
    
   1
    
   
  
 
     gi|227818013|BAMEG_5539|
  6.3e-10
    
    
   1
    
   
  
 
     gi|30265276|BA5493|NP_84
  6.3e-10
    
    
   1
    
   
  
 
     gi|47527789|GBAA2502|YP_
  6.3e-10
    
    
   1
    
   
  
 
     gi|217962737|BCAH187_A54
  6.3e-10
    
    
   1
    
   
  
 
     gi|222098712|BCQ_5081|YP
  6.3e-10
    
    
   1
    
   
  
 
     gi|229603576|BAA_5520|YP
  6.3e-10
    
    
   1
    
   
  
 
     gi|49481227|BT9727_4932|
  6.3e-10
    
    
   1
    
   
  
 
     gi|163942935|BcerKBAB4_5
  6.3e-10
    
    
   1
    
   
  
 
     gi|218900375|BCG9842_B55
  6.3e-10
    
    
   1
    
   
  
 
     gi|49185332|BAS2323|YP_0
  6.3e-10
    
    
   1
    
   
  
 
     gi|30262499|BA2502|NP_84
  6.3e-10
    
    
   1
    
   
  
 
     gi|42784415|BCE_5370|NP_
  6.3e-10
    
    
   1
    
   
  
 
     gi|52140312|BCZK4947|YP_
  6.3e-10
    
    
   1
    
   
  
 
     gi|47530811|GBAA5493|YP_
  6.3e-10
    
    
   1
    
   
  
 
     gi|49188088|BAS5102|YP_0
  6.3e-10
    
    
   1
    
   
  
 
     gi|229604897|BAA_2557|YP
  6.3e-10
    
    
   1
    
   
  
 
     gi|295704249|BMD_2121|YP
  6.3e-10
    
    
   1
    
   
  
 
     gi|296505675|BMB171_C484
  6.3e-10
    
    
   1
    
   
  
 
     gi|294498927|BMQ_2164|YP
  6.3e-10
    
    
   1
    
   
  
 
     gi|301056717|BACI_c52440
  6.3e-10
    
    
   1
    
   
  
 
     gi|15613235|BH0672|NP_24
  6.3e-10
    
    
   1
    
   
  
 
     gi|288556053|BpOF4_15235
  7e-10
    
    
   1
    
   
  
 
     gi|227814227|BAMEG_1635|
  7.7e-10
    
    
   1
    
   
  
 
     gi|49185764|BAS2758|YP_0
  7.7e-10
    
    
   1
    
   
  
 
     gi|218904082|BCAH820_296
  7.7e-10
    
    
   1
    
   
  
 
     gi|161486563|BA2970|NP_8
  7.7e-10
    
    
   1
    
   
  
 
     gi|229602282|BAA_3022|YP
  7.7e-10
    
    
   1
    
   
  
 
     gi|161611198|GBAA2970|YP
  7.7e-10
    
    
   1
    
   
  
 
     gi|15615924|BH3362|NP_24
  7.7e-10
    
    
   1
    
   
  
 
     gi|49478105|BT9727_3026|
  8.6e-10
    
    
   1
    
   
  
 
     gi|218904418|BCAH820_330
  8.6e-10
    
    
   1
    
   
  
 
     gi|52142270|BCZK2972|YP_
  8.6e-10
    
    
   1
    
   
  
 
     gi|47528609|GBAA3324|YP_
  8.6e-10
    
    
   1
    
   
  
 
     gi|225865247|BCA_3356|YP
  8.6e-10
    
    
   1
    
   
  
 
     gi|227813895|BAMEG_1301|
  8.6e-10
    
    
   1
    
   
  
 
     gi|229604117|BAA_3359|YP
  8.6e-10
    
    
   1
    
   
  
 
     gi|49186087|BAS3082|YP_0
  8.6e-10
    
    
   1
    
   
  
 
     gi|30263235|BA3324|NP_84
  8.6e-10
    
    
   1
    
   
  
 
     gi|163940993|BcerKBAB4_3
  8.6e-10
    
    
   1
    
   
  
 
     gi|301054766|BACI_c32210
  8.6e-10
    
    
   1
    
   
  
 
     gi|56420261|GK1726|YP_14
  9.5e-10
    
    
   1
    
   
  
 
     gi|23100547|OB3092|NP_69
  9.5e-10
    
    
   1
    
   
  
 
     gi|56964615|ABC2851|YP_1
  9.5e-10
    
    
   1
    
   
  
 
     gi|56418685|GK0150|YP_14
  1.1e-09
    
    
   1
    
   
  
 
     gi|16078017|BSU09520|NP_
  1.1e-09
    
    
   1
    
   
  
 
     gi|288553039|BpOF4_00065
  1.1e-09
    
    
   1
    
   
  
 
     gi|52784792|BLi01020|YP_
  1.1e-09
    
    
   1
    
   
  
 
     gi|154686555|RBAM_021240
  1.1e-09
    
    
   1
    
   
  
 
     gi|261417650|GYMC61_0150
  1.1e-09
    
    
   1
    
   
  
 
     gi|52079432|BL02851|YP_0
  1.1e-09
    
    
   1
    
   
  
 
     gi|157691684|BPUM_0902|Y
  1.1e-09
    
    
   1
    
   
  
 
     gi|154685410|RBAM_009760
  1.1e-09
    
    
   1
    
   
  
 
     gi|297528524|GC56T3_0148
  1.1e-09
    
    
   1
    
   
  
 
     gi|138895828|GTNG_2187|Y
  1.1e-09
    
    
   1
    
   
  
 
     gi|16078710|BSU16470|NP_
  1.2e-09
    
    
   1
    
   
  
 
     gi|154686064|RBAM_016310
  1.2e-09
    
    
   1
    
   
  
 
     gi|294509181|BMQ_pBM5009
  1.2e-09
    
    
   1
    
   
  
 
     gi|261404422|GYMC10_0553
  1.3e-09
    
    
   1
    
   
  
 
     gi|217959830|BCAH187_A24
  1.4e-09
    
    
   1
    
   
  
 
     gi|222095915|BCQ_2255|YP
  1.4e-09
    
    
   1
    
   
  
 
     gi|56420789|GK2254|YP_14
  1.4e-09
    
    
   1
    
   
  
 
     gi|261417910|GYMC61_0428
  1.4e-09
    
    
   1
    
   
  
 
     gi|297529579|GC56T3_1250
  1.4e-09
    
    
   1
    
   
  
 
     gi|261408790|GYMC10_5011
  1.6e-09
    
    
   1
    
   
  
 
     gi|52143316|BCZK1921|YP_
  1.7e-09
    
    
   1
    
   
  
 
     gi|118477553|BALH_1878|Y
  1.7e-09
    
    
   1
    
   
  
 
     gi|222095751|BCQ_2091|YP
  1.7e-09
    
    
   1
    
   
  
 
     gi|42781261|BCE_2195|NP_
  1.7e-09
    
    
   1
    
   
  
 
     gi|163939932|BcerKBAB4_1
  1.7e-09
    
    
   1
    
   
  
 
     gi|217959667|BCAH187_A22
  1.7e-09
    
    
   1
    
   
  
 
     gi|49477520|BT9727_1945|
  1.7e-09
    
    
   1
    
   
  
 
     gi|225864098|BCA_2202|YP
  1.7e-09
    
    
   1
    
   
  
 
     gi|218903264|BCAH820_214
  1.7e-09
    
    
   1
    
   
  
 
     gi|218897120|BCG9842_B31
  1.7e-09
    
    
   1
    
   
  
 
     gi|301053658|BACI_c20780
  1.7e-09
    
    
   1
    
   
  
 
     gi|15616194|BH3632|NP_24
  1.7e-09
    
    
   1
    
   
  
 
     gi|138894123|GTNG_0449|Y
  2.1e-09
    
    
   1
    
   
  
 
     gi|288555447|BpOF4_12185
  2.1e-09
    
    
   1
    
   
  
 
     gi|15614994|BH2431|NP_24
  2.3e-09
    
    
   1
    
   
  
 
     gi|30262705|BA2732|NP_84
  2.6e-09
    
    
   1
    
   
  
 
     gi|227814455|BAMEG_1863|
  2.6e-09
    
    
   1
    
   
  
 
     gi|49185553|BAS2545|YP_0
  2.6e-09
    
    
   1
    
   
  
 
     gi|52142774|BCZK2466|YP_
  2.6e-09
    
    
   1
    
   
  
 
     gi|118478099|BALH_2454|Y
  2.6e-09
    
    
   1
    
   
  
 
     gi|152977601|Bcer98_3939
  2.6e-09
    
    
   1
    
   
  
 
     gi|218903856|BCAH820_274
  2.6e-09
    
    
   1
    
   
  
 
     gi|229603088|BAA_2796|YP
  2.6e-09
    
    
   1
    
   
  
 
     gi|47528023|GBAA2732|YP_
  2.6e-09
    
    
   1
    
   
  
 
     gi|49477843|BT9727_2501|
  2.6e-09
    
    
   1
    
   
  
 
     gi|225864707|BCA_2814|YP
  2.6e-09
    
    
   1
    
   
  
 
     gi|222096241|BCQ_2581|YP
  2.6e-09
    
    
   1
    
   
  
 
     gi|301054256|BACI_c26970
  2.6e-09
    
    
   1
    
   
  
 
     gi|261409947|GYMC10_6177
  2.6e-09
    
    
   1
    
   
  
 
     gi|157691378|BPUM_0588|Y
  2.6e-09
    
    
   1
    
   
  
 
     gi|172056180|Exig_0136|Y
  2.6e-09
    
    
   1
    
   
  
 
     gi|217957742|BCAH187_A02
  3.1e-09
    
    
   1
    
   
  
 
     gi|229601254|BAA_0200|YP
  3.1e-09
    
    
   1
    
   
  
 
     gi|52145050|BCZK0162|YP_
  3.1e-09
    
    
   1
    
   
  
 
     gi|49183204|BAS0171|YP_0
  3.1e-09
    
    
   1
    
   
  
 
     gi|227812842|BAMEG_0200|
  3.1e-09
    
    
   1
    
   
  
 
     gi|225862220|BCA_0212|YP
  3.1e-09
    
    
   1
    
   
  
 
     gi|163938169|BcerKBAB4_0
  3.1e-09
    
    
   1
    
   
  
 
     gi|222093937|BCQ_0193|YP
  3.1e-09
    
    
   1
    
   
  
 
     gi|47525427|GBAA0169|YP_
  3.1e-09
    
    
   1
    
   
  
 
     gi|30260357|BA0169|NP_84
  3.1e-09
    
    
   1
    
   
  
 
     gi|218901372|BCAH820_019
  3.1e-09
    
    
   1
    
   
  
 
     gi|118475937|BALH_0169|Y
  3.1e-09
    
    
   1
    
   
  
 
     gi|169829988|Bsph_4570|Y
  3.1e-09
    
    
   1
    
   
  
 
     gi|42779274|BCE_0193|NP_
  3.1e-09
    
    
   1
    
   
  
 
     gi|288556517|BpOF4_17595
  3.1e-09
    
    
   1
    
   
  
 
     gi|169827960|Bsph_2435|Y
  3.1e-09
    
    
   1
    
   
  
 
     gi|261404968|GYMC10_1112
  3.4e-09
    
    
   1
    
   
  
 
     gi|261406319|GYMC10_2476
  3.7e-09
    
    
   1
    
   
  
 
     gi|218896046|BCG9842_B42
  4.1e-09
    
    
   1
    
   
  
 
     gi|218233874|BCB4264_A10
  4.1e-09
    
    
   1
    
   
  
 
     gi|163941054|BcerKBAB4_3
  4.1e-09
    
    
   1
    
   
  
 
     gi|296501706|BMB171_C086
  4.1e-09
    
    
   1
    
   
  
 
     gi|218232506|BCB4264_A54
  4.5e-09
    
    
   1
    
   
  
 
     gi|296506459|BMB171_P007
  4.5e-09
    
    
   1
    
   
  
 
     gi|67078088|pE33L466_021
  4.5e-09
    
    
   1
    
   
  
 
     gi|261406207|GYMC10_2363
  5.4e-09
    
    
   1
    
   
  
 
     gi|294498777|BMQ_2014|YP
  6.5e-09
    
    
   1
    
   
  
 
     gi|294497028|BMQ_0193|YP
  6.5e-09
    
    
   1
    
   
  
 
     gi|295704098|BMD_1970|YP
  6.5e-09
    
    
   1
    
   
  
 
     gi|295702393|BMD_0187|YP
  6.5e-09
    
    
   1
    
   
  
 
     gi|255767497|BSU23100|NP
  7.7e-09
    
    
   1
    
   
  
 
     gi|157691562|BPUM_0780|Y
  7.7e-09
    
    
   1
    
   
  
 
     gi|294509114|BMQ_pBM5002
  7.7e-09
    
    
   1
    
   
  
 
     gi|227813117|BAMEG_0515|
  8.4e-09
    
    
   1
    
   
  
 
     gi|229600567|BAA_4139|YP
  8.4e-09
    
    
   1
    
   
  
 
     gi|47529410|GBAA4115|YP_
  8.4e-09
    
    
   1
    
   
  
 
     gi|30263977|BA4115|NP_84
  8.4e-09
    
    
   1
    
   
  
 
     gi|49186821|BAS3823|YP_0
  8.4e-09
    
    
   1
    
   
  
 
     gi|218906428|BCAH820_534
  9.2e-09
    
    
   1
    
   
  
 
     gi|261407370|GYMC10_3567
  9.2e-09
    
    
   1
    
   
  
 
     gi|15614178|BH1615|NP_24
  9.2e-09
    
    
   1
    
   
  
 
     gi|154685892|RBAM_014590
  1.2e-08
    
    
   1
    
   
  
 
     gi|261407639|GYMC10_3840
  1.2e-08
    
    
   1
    
   
  
 
     gi|229917320|EAT1b_1595|
  1.3e-08
    
    
   1
    
   
  
 
     gi|56962021|ABC0239|YP_1
  1.3e-08
    
    
   1
    
   
  
 
     gi|23098724|OB1269|NP_69
  1.4e-08
    
    
   1
    
   
  
 
     gi|15612826|BH0263|NP_24
  1.5e-08
    
    
   1
    
   
  
 
     gi|23100811|OB3356|NP_69
  1.7e-08
    
    
   1
    
   
  
 
     gi|15615679|BH3117|NP_24
  1.7e-08
    
    
   1
    
   
  
 
     gi|152977451|Bcer98_3783
  1.8e-08
    
    
   1
    
   
  
 
     gi|138894766|GTNG_1100|Y
  1.8e-08
    
    
   1
    
   
  
 
     gi|294497918|BMQ_1151|YP
  1.8e-08
    
    
   1
    
   
  
 
     gi|295703274|BMD_1138|YP
  1.8e-08
    
    
   1
    
   
  
 
     gi|56963737|ABC1972|YP_1
  1.8e-08
    
    
   1
    
   
  
 
     gi|261406097|GYMC10_2252
  2e-08
    
    
   1
    
   
  
 
     gi|288554775|BpOF4_08800
  2.2e-08
    
    
   1
    
   
  
 
     gi|297582837|Bsel_0515|Y
  2.2e-08
    
    
   1
    
   
  
 
     gi|56419781|GK1246|YP_14
  2.5e-08
    
    
   1
    
   
  
 
     gi|261419446|GYMC61_2030
  2.5e-08
    
    
   1
    
   
  
 
     gi|52080075|BL02968|YP_0
  2.5e-08
    
    
   1
    
   
  
 
     gi|52785449|BLi01689|YP_
  2.5e-08
    
    
   1
    
   
  
 
     gi|218848158|BCG9842_003
  2.5e-08
    
    
   1
    
   
  
 
     gi|297530579|GC56T3_2307
  2.5e-08
    
    
   1
    
   
  
 
     gi|49185294|BAS2285|YP_0
  2.8e-08
    
    
   1
    
   
  
 
     gi|227814737|BAMEG_2147|
  2.8e-08
    
    
   1
    
   
  
 
     gi|30020517|BC2386|NP_83
  2.8e-08
    
    
   1
    
   
  
 
     gi|30262453|BA2454|NP_84
  2.8e-08
    
    
   1
    
   
  
 
     gi|47527745|GBAA2454|YP_
  2.8e-08
    
    
   1
    
   
  
 
     gi|52143032|BCZK2206|YP_
  2.8e-08
    
    
   1
    
   
  
 
     gi|49477707|BT9727_2248|
  2.8e-08
    
    
   1
    
   
  
 
     gi|217959947|BCAH187_A25
  2.8e-08
    
    
   1
    
   
  
 
     gi|118477845|BALH_2188|Y
  2.8e-08
    
    
   1
    
   
  
 
     gi|218233483|BCB4264_A24
  2.8e-08
    
    
   1
    
   
  
 
     gi|225864413|BCA_2518|YP
  2.8e-08
    
    
   1
    
   
  
 
     gi|218903588|BCAH820_247
  2.8e-08
    
    
   1
    
   
  
 
     gi|222096033|BCQ_2373|YP
  2.8e-08
    
    
   1
    
   
  
 
     gi|229601988|BAA_2511|YP
  2.8e-08
    
    
   1
    
   
  
 
     gi|261405039|GYMC10_1184
  2.8e-08
    
    
   1
    
   
  
 
     gi|16080921|BSU38700|NP_
  2.8e-08
    
    
   1
    
   
  
 
     gi|296502978|BMB171_C214
  2.8e-08
    
    
   1
    
   
  
 
     gi|301053967|BACI_c23980
  2.8e-08
    
    
   1
    
   
  
 
     gi|16078537|BSU14730|NP_
  3e-08
    
    
   1
    
   
  
 
     gi|172056208|Exig_0164|Y
  3e-08
    
    
   1
    
   
  
 
     gi|15615785|BH3223|NP_24
  3e-08
    
    
   1
    
   
  
 
     gi|23098753|OB1298|NP_69
  3.5e-08
    
    
   1
    
   
  
 
     gi|261406442|GYMC10_2604
  3.5e-08
    
    
   1
    
   
  
 
     gi|297582696|Bsel_0370|Y
  3.5e-08
    
    
   1
    
   
  
 
     gi|229917220|EAT1b_1495|
  3.5e-08
    
    
   1
    
   
  
 
     gi|212639788|Aflv_1962|Y
  3.8e-08
    
    
   1
    
   
  
 
     gi|239827399|GWCH70_2031
  3.8e-08
    
    
   1
    
   
  
 
     gi|297584063|Bsel_1770|Y
  4.1e-08
    
    
   1
    
   
  
 
     gi|157694260|BPUM_3514|Y
  4.1e-08
    
    
   1
    
   
  
 
     gi|49184672|BAS1658|YP_0
  4.4e-08
    
    
   1
    
   
  
 
     gi|163939640|BcerKBAB4_1
  4.4e-08
    
    
   1
    
   
  
 
     gi|222095458|BCQ_1798|YP
  4.4e-08
    
    
   1
    
   
  
 
     gi|42780931|BCE_1861|NP_
  4.4e-08
    
    
   1
    
   
  
 
     gi|217959319|BCAH187_A19
  4.4e-08
    
    
   1
    
   
  
 
     gi|47527079|GBAA1789|YP_
  4.4e-08
    
    
   1
    
   
  
 
     gi|225863696|BCA_1798|YP
  4.4e-08
    
    
   1
    
   
  
 
     gi|30261838|BA1789|NP_84
  4.4e-08
    
    
   1
    
   
  
 
     gi|218902955|BCAH820_183
  4.4e-08
    
    
   1
    
   
  
 
     gi|49481088|BT9727_1636|
  4.4e-08
    
    
   1
    
   
  
 
     gi|218896772|BCG9842_B35
  4.4e-08
    
    
   1
    
   
  
 
     gi|227815388|BAMEG_2801|
  4.4e-08
    
    
   1
    
   
  
 
     gi|118477262|BALH_1573|Y
  4.4e-08
    
    
   1
    
   
  
 
     gi|229602836|BAA_1861|YP
  4.4e-08
    
    
   1
    
   
  
 
     gi|52143630|BCZK1605|YP_
  4.4e-08
    
    
   1
    
   
  
 
     gi|301053373|BACI_c17850
  4.4e-08
    
    
   1
    
   
  
 
     gi|295696140|Btus_1522|Y
  4.4e-08
    
    
   1
    
   
  
 
     gi|295694973|Btus_0293|Y
  4.8e-08
    
    
   1
    
   
  
 
     gi|163942349|BcerKBAB4_4
  5.6e-08
    
    
   1
    
   
  
 
     gi|138894925|GTNG_1263|Y
  5.6e-08
    
    
   1
    
   
  
 
     gi|261407955|GYMC10_4162
  6.1e-08
    
    
   1
    
   
  
 
     gi|295696987|Btus_2417|Y
  6.5e-08
    
    
   1
    
   
  
 
     gi|218905848|BCAH820_473
  7.1e-08
    
    
   1
    
   
  
 
     gi|217962100|BCAH187_A47
  7.1e-08
    
    
   1
    
   
  
 
     gi|49478708|BT9727_4352|
  7.1e-08
    
    
   1
    
   
  
 
     gi|222098082|BCQ_4424|YP
  7.1e-08
    
    
   1
    
   
  
 
     gi|225866598|BCA_4732|YP
  7.1e-08
    
    
   1
    
   
  
 
     gi|118479762|BALH_4199|Y
  7.1e-08
    
    
   1
    
   
  
 
     gi|294500913|BMQ_4167|YP
  7.1e-08
    
    
   1
    
   
  
 
     gi|295706259|BMD_4154|YP
  7.1e-08
    
    
   1
    
   
  
 
     gi|295705020|BMD_2904|YP
  7.1e-08
    
    
   1
    
   
  
 
     gi|294499631|BMQ_2875|YP
  7.1e-08
    
    
   1
    
   
  
 
     gi|169827296|Bsph_1728|Y
  7.6e-08
    
    
   1
    
   
  
 
     gi|169826467|Bsph_0879|Y
  9.5e-08
    
    
   1
    
   
  
 
     gi|23100228|OB2773|NP_69
  9.5e-08
    
    
   1
    
   
  
 
     gi|295695225|Btus_0554|Y
  1.1e-07
    
    
   1
    
   
  
 
     gi|261409914|GYMC10_6143
  1.1e-07
    
    
   1
    
   
  
 
     gi|295705907|BMD_3799|YP
  1.3e-07
    
    
   1
    
   
  
 
     gi|294500554|BMQ_3807|YP
  1.3e-07
    
    
   1
    
   
  
 
     gi|239826640|GWCH70_1138
  1.3e-07
    
    
   1
    
   
  
 
     gi|172058539|Exig_2532|Y
  1.4e-07
    
    
   1
    
   
  
 
     gi|297582728|Bsel_0402|Y
  1.6e-07
    
    
   1
    
   
  
 
     gi|23099037|OB1582|NP_69
  1.8e-07
    
    
   1
    
   
  
 
     gi|56965809|ABC4051|YP_1
  1.8e-07
    
    
   1
    
   
  
 
     gi|157694452|BPUM_3710|Y
  2.1e-07
    
    
   1
    
   
  
 
     gi|261405575|GYMC10_1726
  2.1e-07
    
    
   1
    
   
  
 
     gi|261409324|GYMC10_5549
  2.1e-07
    
    
   1
    
   
  
 
     gi|297582431|Bsel_0096|Y
  2.4e-07
    
    
   1
    
   
  
 
     gi|56421959|GK3424|YP_14
  2.6e-07
    
    
   1
    
   
  
 
     gi|261420836|GYMC61_3488
  2.6e-07
    
    
   1
    
   
  
 
     gi|42780194|BCE_1118|NP_
  2.6e-07
    
    
   1
    
   
  
 
     gi|261404205|GYMC10_0333
  2.6e-07
    
    
   1
    
   
  
 
     gi|297531625|GC56T3_3410
  2.6e-07
    
    
   1
    
   
  
 
     gi|169826213|Bsph_0618|Y
  2.6e-07
    
    
   1
    
   
  
 
     gi|138897001|GTNG_3372|Y
  2.8e-07
    
    
   1
    
   
  
 
     gi|47530207|GBAA4913|YP_
  3e-07
    
    
   1
    
   
  
 
     gi|222098134|BCQ_4476|YP
  3e-07
    
    
   1
    
   
  
 
     gi|218233191|BCB4264_A47
  3e-07
    
    
   1
    
   
  
 
     gi|30264734|BA4913|NP_84
  3e-07
    
    
   1
    
   
  
 
     gi|42783844|BCE_4798|NP_
  3e-07
    
    
   1
    
   
  
 
     gi|217962148|BCAH187_A47
  3e-07
    
    
   1
    
   
  
 
     gi|227817453|BAMEG_4945|
  3e-07
    
    
   1
    
   
  
 
     gi|52140841|BCZK4409|YP_
  3e-07
    
    
   1
    
   
  
 
     gi|218905890|BCAH820_477
  3e-07
    
    
   1
    
   
  
 
     gi|229604251|BAA_4924|YP
  3e-07
    
    
   1
    
   
  
 
     gi|49187552|BAS4558|YP_0
  3e-07
    
    
   1
    
   
  
 
     gi|118479803|BALH_4240|Y
  3e-07
    
    
   1
    
   
  
 
     gi|301056170|BACI_c46590
  3e-07
    
    
   1
    
   
  
 
     gi|49478801|BT9727_4602|
  3.4e-07
    
    
   1
    
   
  
 
     gi|163942610|BcerKBAB4_4
  3.4e-07
    
    
   1
    
   
  
 
     gi|295694956|Btus_0276|Y
  3.4e-07
    
    
   1
    
   
  
 
     gi|229917493|EAT1b_1768|
  3.4e-07
    
    
   1
    
   
  
 
     gi|169827905|Bsph_2380|Y
  3.7e-07
    
    
   1
    
   
  
 
     gi|229916805|EAT1b_1078|
  3.9e-07
    
    
   1
    
   
  
 
     gi|169828654|Bsph_3171|Y
  3.9e-07
    
    
   1
    
   
  
 
     gi|261407440|GYMC10_3639
  4.2e-07
    
    
   1
    
   
  
 
     gi|163940540|BcerKBAB4_2
  4.8e-07
    
    
   1
    
   
  
 
     gi|261409306|GYMC10_5531
  4.8e-07
    
    
   1
    
   
  
 
     gi|261404261|GYMC10_0389
  5.1e-07
    
    
   1
    
   
  
 
     gi|169829956|Bsph_4538|Y
  5.1e-07
    
    
   1
    
   
  
 
     gi|163943299|BcerKBAB4_5
  5.8e-07
    
    
   1
    
   
  
 
     gi|152975676|Bcer98_1911
  5.8e-07
    
    
   1
    
   
  
 
     gi|212639538|Aflv_1712|Y
  5.8e-07
    
    
   1
    
   
  
 
     gi|261404314|GYMC10_0444
  6.2e-07
    
    
   1
    
   
  
 
     gi|15613183|BH0620|NP_24
  6.7e-07
    
    
   1
    
   
  
 
     gi|288555440|BpOF4_12150
  7.6e-07
    
    
   1
    
   
  
 
     gi|294500314|BMQ_3558|YP
  7.6e-07
    
    
   1
    
   
  
 
     gi|295705661|BMD_3546|YP
  7.6e-07
    
    
   1
    
   
  
 
     gi|16079766|BSU27120|NP_
  1e-06
    
    
   1
    
   
  
 
     gi|154685119|RBAM_006640
  1e-06
    
    
   1
    
   
  
 
     gi|52079036|BL05045|YP_0
  1.1e-06
    
    
   1
    
   
  
 
     gi|52784408|BLi00595|YP_
  1.1e-06
    
    
   1
    
   
  
 
     gi|218900006|BCG9842_B02
  1.4e-06
    
    
   1
    
   
  
 
     gi|23098703|OB1248|NP_69
  1.5e-06
    
    
   1
    
   
  
 
     gi|169829522|Bsph_4082|Y
  2.2e-06
    
    
   1
    
   
  
 
     gi|295705993|BMD_3885|YP
  2.5e-06
    
    
   1
    
   
  
 
     gi|261404792|GYMC10_0928
  2.6e-06
    
    
   1
    
   
  
 
     gi|239826131|GWCH70_0591
  3.5e-06
    
    
   1
    
   
  
 
     gi|163940227|BcerKBAB4_2
  4.1e-06
    
    
   1
    
   
  
 
     gi|288554248|BpOF4_06145
  6.5e-06
    
    
   1
    
   
  
 
     gi|15615778|BH3216|NP_24
  8.1e-06
    
    
   1
    
   
  
 
     gi|163939522|BcerKBAB4_1
  1e-05
    
    
   1
    
   
  
 
     gi|218234226|BCB4264_A16
  1e-05
    
    
   1
    
   
  
 
     gi|23099768|OB2313|NP_69
  1.2e-05
    
    
   1
    
   
  
 
     gi|169826679|Bsph_1097|Y
  1.4e-05
    
    
   1
    
   
  
 
     gi|169829980|Bsph_4562|Y
  4e-05
    
    
   1
    
   
  
 
     gi|15616444|BH3882|NP_24
  5e-05
    
    
   1
    
   
  
 
     gi|15614589|BH2026|NP_24
  0.00016
    
    
   1
    
   
  
 
     gi|49479940|BT9727_1587|
  0.0011
    
    
   1
    
   
  
 
     gi|163940708|BcerKBAB4_2
  0.0017
    
    
   1
    
   
  
 
     gi|16079737|BSU26840|NP_
  0.0024
    
    
   1
    
   
  
  SCALE
     
     | 
     | 
     | 
     | 
     | 
     | 
     | 
     | 
     | 
     | 
     | 
     | 
     | 
     | 
     | 
     | 
     | 
     | 
     | 
     | 
     | 
     | 
     | 
     | 
     | 
     | 
     | 
     | 
     | 
     | 
     | 
     | 
     | 
    1 
     25 
     50 
     75 
     100 
     125 
     150 
     175 
     200 
     225 
     250 
     275 
     300 
     325 
     350 
     375 
     400 
     425 
     450 
     475 
     500 
     525 
     550 
     575 
     600 
     625 
     650 
     675 
     700 
     725 
     750 
     775 
     800 
   
 

    
      	Motif 1 in BLOCKS format  
    
 
 
 
 
  
 to  BLOCKS multiple alignment processor.  
    
      	Motif 1 position-specific scoring matrix  
    
 
 

    
      	Motif 1 position-specific probability matrix  
    
 
 

    
      	Motif 1 regular expression  
    
[ED]D[LI][VI]QE[GT]FI[GK]LYK[AS][ILV]

 


Time 7149.97 secs.

 

    
   
     P  
     N        MOTIF  2       width = 13         sites = 298        llr = 7045        E-value = 8.4e-1697 
    
 
    Simplified  A  : : 1 : : : : 1 : 3 1 : a
    pos.-specific  C  : : : : : : : : : : : : :
    probability  D  : 6 : : : 2 : : : 1 : : :
    matrix  E  : : : 2 : : : : : 1 : : :
    F  7 : : : 1 : 1 : 7 : : 2 :
    G  : : : 2 : 5 : : : : : : :
    H  : : : : : : : : : : : : :
    I  : : : : 1 : 1 : : : : : :
    K  : : : 1 5 1 1 7 : : : : :
    L  : : 3 : : : 1 : 3 1 : : :
    M  : : : : : : : : : : : : :
    N  : 1 : 1 : 1 1 : : : : : :
    P  : : 3 : : : : : : : 1 : :
    Q  : : : : 1 : : : : : : : :
    R  : : : 1 1 : : : : : : : :
    S  : 2 : 3 : : : 1 : 4 1 : :
    T  : : : : : : 1 : : : 8 : :
    V  : : : : : : 3 : : : : : :
    W  : : : : : : : : : : : : :
    Y  3 : 3 : 1 : 1 : : : : 8 :
  . 
               bits      7.3              
                      6.6              
                      5.9              
                      5.1              
      Information   4.4               
      content   3.7                   
    (34.1 bits) 2.9                    
                      2.2                        
                      1.5                           
                      0.7                           
                      0.0    
  . 
    Multilevel                 F      D      Y      S      K      G      V      K      F      S      T      Y      A   
    consensus                 Y      P    G            L    A      F    
    sequence                      L                      
                                                    
                                                    

  . 
  NAME       START   P-VALUE           SITES   
  gi|261407775|GYMC10_3981    123  1.91e-16 
    NMGLIKAVEK     F      D      Y      S      K      G      F      K      F      S      T      Y      A       TWWIRQAITR 
  gi|218232261|BCB4264_A44    182  2.30e-15 
    NMGLIKAVEK     F      D      Y      R      K      G      F      K      F      S      T      Y      A       TWWIRQAITR 
  gi|52141207|BCZK4042|YP_    180  2.30e-15 
    NMGLIKAVEK     F      D      Y      R      K      G      F      K      F      S      T      Y      A       TWWIRQAITR 
  gi|42783418|BCE_4372|NP_    182  2.30e-15 
    NMGLIKAVEK     F      D      Y      R      K      G      F      K      F      S      T      Y      A       TWWIRQAITR 
  gi|30264362|BA4515|NP_84    180  2.30e-15 
    NMGLIKAVEK     F      D      Y      R      K      G      F      K      F      S      T      Y      A       TWWIRQAITR 
  gi|222097738|BCQ_4079|YP    182  2.30e-15 
    NMGLIKAGEK     F      D      Y      R      K      G      F      K      F      S      T      Y      A       TWWIRQAITR 
  gi|218899458|BCG9842_B08    182  2.30e-15 
    NMGLIKAVEK     F      D      Y      R      K      G      F      K      F      S      T      Y      A       TWWIRQAITR 
  gi|218905429|BCAH820_431    180  2.30e-15 
    NMGLIKAVEK     F      D      Y      R      K      G      F      K      F      S      T      Y      A       TWWIRQAITR 
  gi|229601154|BAA_4536|YP    180  2.30e-15 
    NMGLIKAVEK     F      D      Y      R      K      G      F      K      F      S      T      Y      A       TWWIRQAITR 
  gi|30022370|BC4289|NP_83    182  2.30e-15 
    NMGLIKAVEK     F      D      Y      R      K      G      F      K      F      S      T      Y      A       TWWIRQAITR 
  gi|152976724|Bcer98_3019    180  2.30e-15 
    NMGLIKAVEK     F      D      Y      R      K      G      F      K      F      S      T      Y      A       TWWIRQAITR 
  gi|163942050|BcerKBAB4_4    182  2.30e-15 
    NMGLIKAVEK     F      D      Y      R      K      G      F      K      F      S      T      Y      A       TWWIRQAITR 
  gi|118479464|BALH_3885|Y    180  2.30e-15 
    NMGLIKAVEK     F      D      Y      R      K      G      F      K      F      S      T      Y      A       TWWIRQAITR 
  gi|217961783|BCAH187_A44    182  2.30e-15 
    NMGLIKAVEK     F      D      Y      R      K      G      F      K      F      S      T      Y      A       TWWIRQAITR 
  gi|47529811|GBAA4515|YP_    180  2.30e-15 
    NMGLIKAVEK     F      D      Y      R      K      G      F      K      F      S      T      Y      A       TWWIRQAITR 
  gi|49187190|BAS4194|YP_0    180  2.30e-15 
    NMGLIKAVEK     F      D      Y      R      K      G      F      K      F      S      T      Y      A       TWWIRQAITR 
  gi|225866273|BCA_4403|YP    180  2.30e-15 
    NMGLIKAVEK     F      D      Y      R      K      G      F      K      F      S      T      Y      A       TWWIRQAITR 
  gi|227817068|BAMEG_4554|    180  2.30e-15 
    NMGLIKAVEK     F      D      Y      R      K      G      F      K      F      S      T      Y      A       TWWIRQAITR 
  gi|49481307|BT9727_4032|    180  2.30e-15 
    NMGLIKAVEK     F      D      Y      R      K      G      F      K      F      S      T      Y      A       TWWIRQAITR 
  gi|169829158|Bsph_3702|Y    182  2.30e-15 
    NMGLIKAVEK     F      D      Y      R      K      G      F      K      F      S      T      Y      A       TWWIRQAITR 
  gi|301055785|BACI_c42620    182  2.30e-15 
    NMGLIKAVEK     F      D      Y      R      K      G      F      K      F      S      T      Y      A       TWWIRQAITR 
  gi|23099399|OB1944|NP_69    187  2.30e-15 
    NMGLIKAVEK     F      D      Y      R      K      G      F      K      F      S      T      Y      A       TWWIRQAITR 
  gi|296504786|BMB171_C395    182  2.30e-15 
    NMGLIKAVEK     F      D      Y      R      K      G      F      K      F      S      T      Y      A       TWWIRQAITR 
  gi|297584635|Bsel_2346|Y    178  3.67e-15 
    NMGLIKAVEK     F      D      Y      N      K      G      F      K      F      S      T      Y      A       TWWIRQAITR 
  gi|15613939|BH1376|NP_24    179  3.67e-15 
    NMGLIKAVEK     F      D      Y      N      K      G      F      K      F      S      T      Y      A       TWWIRQAITR 
  gi|261405623|GYMC10_1774    184  9.16e-15 
    NMGLIKAVEK     F      D      Y      K      K      G      F      K      F      S      T      Y      A       TWWIRQAITR 
  gi|56421017|GK2482|YP_14    182  1.41e-14 
    NMGLIKAVEK     F      D      Y      R      K      G      Y      K      F      S      T      Y      A       TWWIRQAITR 
  gi|255767573|BSU25200|NP    178  1.41e-14 
    NMGLMKAVEK     F      D      Y      R      K      G      Y      K      F      S      T      Y      A       TWWIRQAITR 
  gi|212638699|Aflv_0856|Y    179  1.41e-14 
    NMGLIKAVEK     F      D      Y      R      K      G      Y      K      F      S      T      Y      A       TWWIRQAITR 
  gi|295706627|BMD_4528|YP    184  1.41e-14 
    NMGLIKAVEK     F      D      Y      R      K      G      Y      K      F      S      T      Y      A       TWWIRQAITR 
  gi|297529352|GC56T3_1008    182  1.41e-14 
    NMGLIKAVEK     F      D      Y      R      K      G      Y      K      F      S      T      Y      A       TWWIRQAITR 
  gi|261417657|GYMC61_0157    182  1.41e-14 
    NMGLIKAVEK     F      D      Y      R      K      G      Y      K      F      S      T      Y      A       TWWIRQAITR 
  gi|239827752|GWCH70_2414    181  1.41e-14 
    NMGLIKAVEK     F      D      Y      R      K      G      Y      K      F      S      T      Y      A       TWWIRQAITR 
  gi|157693020|BPUM_2253|Y    180  1.41e-14 
    NMGLMKAVEK     F      D      Y      R      K      G      Y      K      F      S      T      Y      A       TWWIRQAITR 
  gi|154686781|RBAM_023510    180  1.41e-14 
    NMGLMKAVEK     F      D      Y      R      K      G      Y      K      F      S      T      Y      A       TWWIRQAITR 
  gi|163119552|BL03682|YP_    180  1.41e-14 
    NMGLMKAVEK     F      D      Y      R      K      G      Y      K      F      S      T      Y      A       TWWIRQAITR 
  gi|52786447|BLi02712|YP_    180  1.41e-14 
    NMGLMKAVEK     F      D      Y      R      K      G      Y      K      F      S      T      Y      A       TWWIRQAITR 
  gi|294501280|BMQ_4542|YP    184  1.41e-14 
    NMGLIKAVEK     F      D      Y      R      K      G      Y      K      F      S      T      Y      A       TWWIRQAITR 
  gi|138896056|GTNG_2419|Y    182  1.41e-14 
    NMGLIKAVEK     F      D      Y      R      K      G      Y      K      F      S      T      Y      A       TWWIRQAITR 
  gi|288555754|BpOF4_13730    179  1.79e-14 
    NMGLIKAVEK     F      D      Y      E      K      G      Y      K      F      S      T      Y      A       TWWIRQAITR 
  gi|56963455|ABC1690|YP_1    180  2.16e-14 
    NMGLIKAVEK     F      D      Y      D      K      G      F      K      F      S      T      Y      A       TWWIRQAITR 
  gi|212639646|Aflv_1820|Y    82  3.93e-14 
    CIGLMKSIDN     F      D      L      S      Q      N      V      K      F      S      T      Y      A       VPMIIGEIRR 
  gi|42782996|BCE_3948|NP_    112  3.93e-14 
    CIGLMKSIDN     F      D      L      G      Q      N      V      K      F      S      T      Y      A       VPMIIGEIRR 
  gi|218233406|BCB4264_A40    82  3.93e-14 
    CIGLMKSIDN     F      D      L      G      Q      N      V      K      F      S      T      Y      A       VPMIIGEIRR 
  gi|227813187|BAMEG_0585|    82  3.93e-14 
    CIGLMKSIDN     F      D      L      G      Q      N      V      K      F      S      T      Y      A       VPMIIGEIRR 
  gi|163941643|BcerKBAB4_3    115  3.93e-14 
    CIGLMKSIDN     F      D      L      G      Q      N      V      K      F      S      T      Y      A       VPMIIGEIRR 
  gi|152976264|Bcer98_2552    82  3.93e-14 
    CIGLMKSIDN     F      D      L      S      Q      N      V      K      F      S      T      Y      A       VPMIIGEIRR 
  gi|49478441|BT9727_3645|    112  3.93e-14 
    CIGLMKSIDN     F      D      L      G      Q      N      V      K      F      S      T      Y      A       VPMIIGEIRR 
  gi|30263905|BA4042|NP_84    82  3.93e-14 
    CIGLMKSIDN     F      D      L      G      Q      N      V      K      F      S      T      Y      A       VPMIIGEIRR 
  gi|217961324|BCAH187_A39    82  3.93e-14 
    CIGLMKSIDN     F      D      L      G      Q      N      V      K      F      S      T      Y      A       VPMIIGEIRR 
  gi|229602177|BAA_4068|YP    82  3.93e-14 
    CIGLMKSIDN     F      D      L      G      Q      N      V      K      F      S      T      Y      A       VPMIIGEIRR 
  gi|30021992|BC3903|NP_83    82  3.93e-14 
    CIGLMKSIDN     F      D      L      G      Q      N      V      K      F      S      T      Y      A       VPMIIGEIRR 
  gi|218905033|BCAH820_391    82  3.93e-14 
    CIGLMKSIDN     F      D      L      G      Q      N      V      K      F      S      T      Y      A       VPMIIGEIRR 
  gi|225865885|BCA_4007|YP    82  3.93e-14 
    CIGLMKSIDN     F      D      L      G      Q      N      V      K      F      S      T      Y      A       VPMIIGEIRR 
  gi|47778237|GBAA4042|YP_    82  3.93e-14 
    CIGLMKSIDN     F      D      L      G      Q      N      V      K      F      S      T      Y      A       VPMIIGEIRR 
  gi|222097349|BCQ_3689|YP    112  3.93e-14 
    CIGLMKSIDN     F      D      L      G      Q      N      V      K      F      S      T      Y      A       VPMIIGEIRR 
  gi|49186753|BAS3754|YP_0    112  3.93e-14 
    CIGLMKSIDN     F      D      L      G      Q      N      V      K      F      S      T      Y      A       VPMIIGEIRR 
  gi|218899057|BCG9842_B12    82  3.93e-14 
    CIGLMKSIDN     F      D      L      G      Q      N      V      K      F      S      T      Y      A       VPMIIGEIRR 
  gi|52141585|BCZK3662|YP_    112  3.93e-14 
    CIGLMKSIDN     F      D      L      G      Q      N      V      K      F      S      T      Y      A       VPMIIGEIRR 
  gi|118479123|BALH_3533|Y    112  3.93e-14 
    CIGLMKSIDN     F      D      L      G      Q      N      V      K      F      S      T      Y      A       VPMIIGEIRR 
  gi|301055394|BACI_c38590    112  3.93e-14 
    CIGLMKSIDN     F      D      L      G      Q      N      V      K      F      S      T      Y      A       VPMIIGEIRR 
  gi|288553150|BpOF4_00620    82  3.93e-14 
    CIGLMKSIDN     F      D      L      S      Q      N      V      K      F      S      T      Y      A       VPMIIGEIRR 
  gi|295706362|BMD_4257|YP    82  3.93e-14 
    CIGLMKSIDN     F      D      L      S      Q      N      V      K      F      S      T      Y      A       VPMIIGEIRR 
  gi|56964115|ABC2350|YP_1    82  3.93e-14 
    CIGLMKSIDN     F      D      L      S      Q      N      V      K      F      S      T      Y      A       VPMIIGEIRR 
  gi|295696452|Btus_1846|Y    82  3.93e-14 
    CIGLMKAIDN     F      D      L      G      Q      N      V      K      F      S      T      Y      A       VPMIIGEIRR 
  gi|296504397|BMB171_C356    82  3.93e-14 
    CIGLMKSIDN     F      D      L      G      Q      N      V      K      F      S      T      Y      A       VPMIIGEIRR 
  gi|261405674|GYMC10_1825    83  3.93e-14 
    CIGLMKAIDN     F      D      L      S      Q      N      V      K      F      S      T      Y      A       VPMIIGEIRR 
  gi|294501013|BMQ_4269|YP    82  3.93e-14 
    CIGLMKSIDN     F      D      L      S      Q      N      V      K      F      S      T      Y      A       VPMIIGEIRR 
  gi|15615117|BH2554|NP_24    82  3.93e-14 
    CIGLMKSIDN     F      D      L      S      Q      N      V      K      F      S      T      Y      A       VPMIIGEIRR 
  gi|218905210|BCAH820_409    77  5.77e-14 
    CIGLLKSVDK     F      D      L      S      F      D      V      K      F      S      T      Y      A       VPMIIGEIQR 
  gi|217961566|BCAH187_A42    77  5.77e-14 
    CIGLLKSVDK     F      D      L      S      F      D      V      K      F      S      T      Y      A       VPMIIGEIQR 
  gi|52141420|BCZK3829|YP_    77  5.77e-14 
    CIGLLKSVDK     F      D      L      S      F      D      V      K      F      S      T      Y      A       VPMIIGEIQR 
  gi|42783188|BCE_4142|NP_    77  5.77e-14 
    CIGLLKSVDK     F      D      L      S      F      D      V      K      F      S      T      Y      A       VPMIIGEIQR 
  gi|47529588|GBAA4294|YP_    77  5.77e-14 
    CIGLLKSVDK     F      D      L      S      F      D      V      K      F      S      T      Y      A       VPMIIGEIQR 
  gi|227816852|BAMEG_4334|    77  5.77e-14 
    CIGLLKSVDK     F      D      L      S      F      D      V      K      F      S      T      Y      A       VPMIIGEIQR 
  gi|118479276|BALH_3690|Y    77  5.77e-14 
    CIGLLKSVDK     F      D      L      S      F      D      V      K      F      S      T      Y      A       VPMIIGEIQR 
  gi|225866059|BCA_4185|YP    77  5.77e-14 
    CIGLLKSVDK     F      D      L      S      F      D      V      K      F      S      T      Y      A       VPMIIGEIQR 
  gi|218899234|BCG9842_B10    77  5.77e-14 
    CIGLLKSVDK     F      D      L      S      F      D      V      K      F      S      T      Y      A       VPMIIGEIQR 
  gi|30264150|BA4294|NP_84    77  5.77e-14 
    CIGLLKSVDK     F      D      L      S      F      D      V      K      F      S      T      Y      A       VPMIIGEIQR 
  gi|49478518|BT9727_3813|    77  5.77e-14 
    CIGLLKSVDK     F      D      L      S      F      D      V      K      F      S      T      Y      A       VPMIIGEIQR 
  gi|218234550|BCB4264_A41    77  5.77e-14 
    CIGLLKSVDK     F      D      L      S      F      D      V      K      F      S      T      Y      A       VPMIIGEIQR 
  gi|222097523|BCQ_3863|YP    77  5.77e-14 
    CIGLLKSVDK     F      D      L      S      F      D      V      K      F      S      T      Y      A       VPMIIGEIQR 
  gi|49186981|BAS3983|YP_0    77  5.77e-14 
    CIGLLKSVDK     F      D      L      S      F      D      V      K      F      S      T      Y      A       VPMIIGEIQR 
  gi|30022159|BC4072|NP_83    77  5.77e-14 
    CIGLLKSVDK     F      D      L      S      F      D      V      K      F      S      T      Y      A       VPMIIGEIQR 
  gi|163941815|BcerKBAB4_3    77  5.77e-14 
    CIGLLKSVDK     F      D      L      S      F      D      V      K      F      S      T      Y      A       VPMIIGEIQR 
  gi|152976482|Bcer98_2771    77  5.77e-14 
    CIGLLKSVDK     F      D      L      S      F      D      V      K      F      S      T      Y      A       VPMIIGEIQR 
  gi|229603990|BAA_4316|YP    77  5.77e-14 
    CIGLLKSVDK     F      D      L      S      F      D      V      K      F      S      T      Y      A       VPMIIGEIQR 
  gi|301055569|BACI_c40400    77  5.77e-14 
    CIGLLKSVDK     F      D      L      S      F      D      V      K      F      S      T      Y      A       VPMIIGEIQR 
  gi|296504567|BMB171_C373    77  5.77e-14 
    CIGLLKSVDK     F      D      L      S      F      D      V      K      F      S      T      Y      A       VPMIIGEIQR 
  gi|172056869|Exig_0832|Y    168  8.31e-14 
    NMGLIKAVEK     F      D      Y      T      K      G      Y      K      F      S      T      Y      A       TWWIRQAITR 
  gi|212638845|Aflv_1004|Y    73  9.85e-14 
    CIGLMKSVDK     F      D      L      S      Y      D      V      K      F      S      T      Y      A       VPMILGEIQR 
  gi|16078597|BSU15330|NP_    83  9.85e-14 
    CIGLMKSIDN     F      D      L      S      H      N      V      K      F      S      T      Y      A       VPMIIGEIRR 
  gi|56420843|GK2308|YP_14    75  9.85e-14 
    CIGLLKSVDK     F      D      L      S      Y      D      V      K      F      S      T      Y      A       VPMIIGEIQR 
  gi|288555973|BpOF4_14835    79  9.85e-14 
    CIGLIKSVDK     F      D      L      S      Y      D      V      K      F      S      T      Y      A       VPMIIGEIQR 
  gi|157692844|BPUM_2076|Y    77  9.85e-14 
    CIGLLKSVDK     F      D      L      S      Y      D      V      K      F      S      T      Y      A       VPMIIGEIQR 
  gi|169827282|Bsph_1714|Y    7  9.85e-14 
    MKSVDK     F      D      L      S      Y      D      V      K      F      S      T      Y      A       VPMIIGEIQR 
  gi|15614101|BH1538|NP_24    77  9.85e-14 
    CIGLIKSVDK     F      D      L      S      Y      D      V      K      F      S      T      Y      A       VPMIIGEIQR 
  gi|294501135|BMQ_4391|YP    77  9.85e-14 
    CIGLLKSVDK     F      D      L      S      Y      D      V      K      F      S      T      Y      A       VPMIIGEIQR 
  gi|52785510|BLi01751|YP_    83  9.85e-14 
    CIGLMKSIDN     F      D      L      S      H      N      V      K      F      S      T      Y      A       VPMIIGEIRR 
  gi|295706482|BMD_4377|YP    77  9.85e-14 
    CIGLLKSVDK     F      D      L      S      Y      D      V      K      F      S      T      Y      A       VPMIIGEIQR 
  gi|56963560|ABC1795|YP_1    77  9.85e-14 
    CIGLIKSVDK     F      D      L      S      Y      D      V      K      F      S      T      Y      A       VPMIIGEIQR 
  gi|154685949|RBAM_015160    83  9.85e-14 
    CIGLMKSIDN     F      D      L      S      H      N      V      K      F      S      T      Y      A       VPMIIGEIRR 
  gi|297529524|GC56T3_1195    77  9.85e-14 
    CIGLLKSVDK     F      D      L      S      Y      D      V      K      F      S      T      Y      A       VPMIIGEIQR 
  gi|239827600|GWCH70_2250    75  9.85e-14 
    CIGLLKSVDK     F      D      L      S      Y      D      V      K      F      S      T      Y      A       VPMIIGEIQR 
  gi|138895880|GTNG_2239|Y    77  9.85e-14 
    CIGLLKSVDK     F      D      L      S      Y      D      V      K      F      S      T      Y      A       VPMIIGEIQR 
  gi|295696440|Btus_1834|Y    77  9.85e-14 
    CIGLMKAIDK     F      D      L      S      Y      D      V      K      F      S      T      Y      A       VPMIIGEIQR 
  gi|261417856|GYMC61_0374    77  9.85e-14 
    CIGLLKSVDK     F      D      L      S      Y      D      V      K      F      S      T      Y      A       VPMIIGEIQR 
  gi|23099294|OB1839|NP_69    75  9.85e-14 
    SIGLIKSIDK     F      D      L      S      Y      D      V      K      F      S      T      Y      A       VPMIIGEIQR 
  gi|261405990|GYMC10_2143    76  9.85e-14 
    CIGLLKSVDK     F      D      L      S      Y      D      V      K      F      S      T      Y      A       VPMIIGEIQR 
  gi|52080136|BL02256|YP_0    83  9.85e-14 
    CIGLMKSIDN     F      D      L      S      H      N      V      K      F      S      T      Y      A       VPMIIGEIRR 
  gi|229916320|EAT1b_0589|    171  1.96e-13 
    NMGLIKAVEK     F      D      Y      M      K      G      F      K      F      S      T      Y      A       TWWIRQAITR 
  gi|56419663|GK1128|YP_14    82  2.73e-13 
    CIGLMKSIDN     F      D      L      N      Q      N      V      K      F      S      T      Y      A       VPMIIGEIRR 
  gi|297530705|GC56T3_2445    82  2.73e-13 
    CIGLMKSIDN     F      D      L      N      Q      N      V      K      F      S      T      Y      A       VPMIIGEIRR 
  gi|239826533|GWCH70_1031    82  2.73e-13 
    CIGLMKSIDN     F      D      L      N      Q      N      V      K      F      S      T      Y      A       VPMIIGEIRR 
  gi|138894663|GTNG_0993|Y    87  2.73e-13 
    CIGLMKSIDN     F      D      L      N      Q      N      V      K      F      S      T      Y      A       VPMIIGEIRR 
  gi|261419325|GYMC61_1901    82  2.73e-13 
    CIGLMKSIDN     F      D      L      N      Q      N      V      K      F      S      T      Y      A       VPMIIGEIRR 
  gi|56421070|GK2535|YP_14    94  3.75e-13 
    TIGLIKAIES     Y      S      P      G      K      G      T      K      L      A      T      Y      A       ARCIENEILM 
  gi|261418447|GYMC61_0982    102  3.75e-13 
    TIGLIKAIES     Y      S      P      G      K      G      T      K      L      A      T      Y      A       ARCIENEILM 
  gi|239827809|GWCH70_2471    94  3.75e-13 
    TIGLIKAIES     Y      S      P      G      K      G      T      K      L      A      T      Y      A       ARCIENEILM 
  gi|297529299|GC56T3_0955    102  3.75e-13 
    TIGLIKAIES     Y      S      P      G      K      G      T      K      L      A      T      Y      A       ARCIENEILM 
  gi|295695448|Btus_0783|Y    186  1.42e-12 
    NLGLIKAVEK     F      D      F      R      K      G      F      K      F      S      T      Y      A       TWWIRQAITR 
  gi|138896107|GTNG_2470|Y    94  1.86e-12 
    TIGLIKAIES     Y      S      P      N      K      G      T      K      L      A      T      Y      A       ARCIENEILM 
  gi|56419781|GK1246|YP_14    71  3.59e-12 
    LVGLYDALEK     F      D      P      S      R      D      L      K      F      D      T      Y      A       SFRIRGAILD 
  gi|212639538|Aflv_1712|Y    61  3.59e-12 
    LLGLYDALEK     F      D      P      S      R      D      L      K      F      D      T      Y      A       SFRIRGAILD 
  gi|16078710|BSU16470|NP_    70  3.59e-12 
    MLGLYDALEK     F      D      P      S      R      D      L      K      F      D      T      Y      A       SFRIRGAIID 
  gi|138894766|GTNG_1100|Y    73  3.59e-12 
    LVGLYDALEK     F      D      P      S      R      D      L      K      F      D      T      Y      A       SFRIRGAILD 
  gi|261419446|GYMC61_2030    71  3.59e-12 
    LVGLYDALEK     F      D      P      S      R      D      L      K      F      D      T      Y      A       SFRIRGAILD 
  gi|261405673|GYMC10_1824    103  3.59e-12 
    AIGLIKAVNT     F      D      P      E      K      K      I      K      L      A      T      Y      A       SRCIENEILM 
  gi|154686064|RBAM_016310    70  3.59e-12 
    MLGLYDALEK     F      D      P      G      R      D      L      K      F      D      T      Y      A       SFRIRGAIID 
  gi|157692327|BPUM_1546|Y    70  3.59e-12 
    MLGLYDALEK     F      D      P      G      R      D      L      K      F      D      T      Y      A       SFRIRGAIID 
  gi|23099037|OB1582|NP_69    71  3.59e-12 
    ILGLYDALKK     F      D      P      S      R      D      L      K      F      D      T      Y      A       SFRIKGSIMD 
  gi|52785627|BLi01868|YP_    70  3.59e-12 
    MLGLYDALEK     F      D      P      G      R      D      L      K      F      D      T      Y      A       SFRIRGAIID 
  gi|52080250|BL01246|YP_0    70  3.59e-12 
    MLGLYDALEK     F      D      P      G      R      D      L      K      F      D      T      Y      A       SFRIRGAIID 
  gi|297530579|GC56T3_2307    71  3.59e-12 
    LVGLYDALEK     F      D      P      S      R      D      L      K      F      D      T      Y      A       SFRIRGAILD 
  gi|239826640|GWCH70_1138    71  3.59e-12 
    LMGLYDALEK     F      D      P      S      R      D      L      K      F      D      T      Y      A       SFRIRGAIFD 
  gi|229603939|BAA_4584|YP    93  4.61e-12 
    TIGLIKAIES     Y      S      A      G      K      G      T      K      L      A      T      Y      A       ARCIENEILM 
  gi|49481469|BT9727_4074|    93  4.61e-12 
    TIGLIKAIES     Y      S      A      G      K      G      T      K      L      A      T      Y      A       ARCIENEILM 
  gi|49187232|BAS4236|YP_0    93  4.61e-12 
    TIGLIKAIES     Y      S      A      G      K      G      T      K      L      A      T      Y      A       ARCIENEILM 
  gi|222097784|BCQ_4125|YP    119  4.61e-12 
    TIGLIKAIES     Y      S      A      G      K      G      T      K      L      A      T      Y      A       ARCIENEILM 
  gi|218905533|BCAH820_441    93  4.61e-12 
    TIGLIKAIES     Y      S      A      G      K      G      T      K      L      A      T      Y      A       ARCIENEILM 
  gi|227817116|BAMEG_4602|    93  4.61e-12 
    TIGLIKAIES     Y      S      A      G      K      G      T      K      L      A      T      Y      A       ARCIENEILM 
  gi|217961829|BCAH187_A44    93  4.61e-12 
    TIGLIKAIES     Y      S      A      G      K      G      T      K      L      A      T      Y      A       ARCIENEILM 
  gi|225866320|BCA_4450|YP    93  4.61e-12 
    TIGLIKAIES     Y      S      A      G      K      G      T      K      L      A      T      Y      A       ARCIENEILM 
  gi|163942095|BcerKBAB4_4    93  4.61e-12 
    TIGLIKAIES     Y      S      A      G      K      G      T      K      L      A      T      Y      A       ARCIENEILM 
  gi|218231149|BCB4264_A44    93  4.61e-12 
    TIGLIKAIES     Y      S      A      G      K      G      T      K      L      A      T      Y      A       ARCIENEILM 
  gi|218899507|BCG9842_B07    93  4.61e-12 
    TIGLIKAIES     Y      S      A      G      K      G      T      K      L      A      T      Y      A       ARCIENEILM 
  gi|118479504|BALH_3926|Y    127  4.61e-12 
    TIGLIKAIES     Y      S      A      G      K      G      T      K      L      A      T      Y      A       ARCIENEILM 
  gi|30022417|BC4336|NP_83    96  4.61e-12 
    TIGLIKAIES     Y      S      A      G      K      G      T      K      L      A      T      Y      A       ARCIENEILM 
  gi|152976769|Bcer98_3065    93  4.61e-12 
    TIGLIKAIES     Y      S      A      G      K      G      T      K      L      A      T      Y      A       ARCIENEILM 
  gi|30264411|BA4566|NP_84    93  4.61e-12 
    TIGLIKAIES     Y      S      A      G      K      G      T      K      L      A      T      Y      A       ARCIENEILM 
  gi|42783467|BCE_4421|NP_    121  4.61e-12 
    TIGLIKAIES     Y      S      A      G      K      G      T      K      L      A      T      Y      A       ARCIENEILM 
  gi|52141167|BCZK4084|YP_    93  4.61e-12 
    TIGLIKAIES     Y      S      A      G      K      G      T      K      L      A      T      Y      A       ARCIENEILM 
  gi|47529862|GBAA4566|YP_    93  4.61e-12 
    TIGLIKAIES     Y      S      A      G      K      G      T      K      L      A      T      Y      A       ARCIENEILM 
  gi|154686834|RBAM_024040    94  4.61e-12 
    TIGLIKGIES     Y      S      A      G      K      G      T      K      L      A      T      Y      A       ARCIENEILM 
  gi|157692208|BPUM_1427|Y    83  4.61e-12 
    CIGLMKSIDN     F      D      L      S      H      N      V      R      F      S      T      Y      A       VPMIIGEIRR 
  gi|52080862|BL00778|YP_0    77  4.61e-12 
    CIGLLKSVDK     F      D      L      S      Y      D      V      R      F      S      T      Y      A       VPMIIGEIQR 
  gi|301055830|BACI_c43070    37  4.61e-12 
    TIGLIKAIES     Y      S      A      G      K      G      T      K      L      A      T      Y      A       ARCIENEILM 
  gi|23098931|OB1476|NP_69    83  4.61e-12 
    CIGLMKSIDN     F      D      L      S      H      N      V      R      F      S      T      Y      A       VPMIIGEIRR 
  gi|52786234|BLi02495|YP_    77  4.61e-12 
    CIGLLKSVDK     F      D      L      S      Y      D      V      R      F      S      T      Y      A       VPMIIGEIQR 
  gi|52081127|BL02107|YP_0    94  4.61e-12 
    TIGLIKAIES     Y      S      A      G      K      G      T      K      L      A      T      Y      A       ARCIENEILM 
  gi|296504831|BMB171_C400    52  4.61e-12 
    TIGLIKAIES     Y      S      A      G      K      G      T      K      L      A      T      Y      A       ARCIENEILM 
  gi|52786504|BLi02769|YP_    94  4.61e-12 
    TIGLIKAIES     Y      S      A      G      K      G      T      K      L      A      T      Y      A       ARCIENEILM 
  gi|154686587|RBAM_021560    77  4.61e-12 
    CIGLLKSVDK     F      D      L      S      Y      D      V      R      F      S      T      Y      A       VPMIIGEIQR 
  gi|229918653|EAT1b_2940|    67  5.21e-12 
    YMGLYDALMK     Y      D      P      E      H      N      N      K      F      D      T      Y      A       AFRIRGAIID 
  gi|294500913|BMQ_4167|YP    71  6.64e-12 
    LIGLYDALEK     F      D      Y      G      R      D      L      K      F      D      T      Y      A       SFRIRGAIID 
  gi|295706259|BMD_4154|YP    71  6.64e-12 
    LIGLYDALEK     F      D      Y      G      R      D      L      K      F      D      T      Y      A       SFRIRGAIID 
  gi|255767354|BSU15320|NP    102  9.47e-12 
    TIGLIKAVNT     F      N      P      E      K      K      I      K      L      A      T      Y      A       SRCIENEILM 
  gi|227813186|BAMEG_0584|    102  9.47e-12 
    TIGLIKAVNT     F      N      P      E      K      K      I      K      L      A      T      Y      A       SRCIENEILM 
  gi|152976265|Bcer98_2553    102  9.47e-12 
    TIGLIKAVNT     F      N      P      E      K      K      I      K      L      A      T      Y      A       SRCIENEILM 
  gi|218899058|BCG9842_B12    102  9.47e-12 
    TIGLIKAVNT     F      N      P      E      K      K      I      K      L      A      T      Y      A       SRCIENEILM 
  gi|218905035|BCAH820_391    102  9.47e-12 
    TIGLIKAVNT     F      N      P      E      K      K      I      K      L      A      T      Y      A       SRCIENEILM 
  gi|30263906|BA4043|NP_84    102  9.47e-12 
    TIGLIKAVNT     F      N      P      E      K      K      I      K      L      A      T      Y      A       SRCIENEILM 
  gi|225865886|BCA_4008|YP    102  9.47e-12 
    TIGLIKAVNT     F      N      P      E      K      K      I      K      L      A      T      Y      A       SRCIENEILM 
  gi|47529336|GBAA4043|YP_    102  9.47e-12 
    TIGLIKAVNT     F      N      P      E      K      K      I      K      L      A      T      Y      A       SRCIENEILM 
  gi|212639647|Aflv_1821|Y    102  9.47e-12 
    TIGLIKAVNT     F      N      P      E      K      K      I      K      L      A      T      Y      A       SRCIENEILM 
  gi|52141583|BCZK3663|YP_    102  9.47e-12 
    TIGLIKAVNT     F      N      P      E      K      K      I      K      L      A      T      Y      A       SRCIENEILM 
  gi|42782997|BCE_3949|NP_    102  9.47e-12 
    TIGLIKAVNT     F      N      P      E      K      K      I      K      L      A      T      Y      A       SRCIENEILM 
  gi|217961326|BCAH187_A39    102  9.47e-12 
    TIGLIKAVNT     F      N      P      E      K      K      I      K      L      A      T      Y      A       SRCIENEILM 
  gi|222097350|BCQ_3690|YP    102  9.47e-12 
    TIGLIKAVNT     F      N      P      E      K      K      I      K      L      A      T      Y      A       SRCIENEILM 
  gi|229601447|BAA_4069|YP    102  9.47e-12 
    TIGLIKAVNT     F      N      P      E      K      K      I      K      L      A      T      Y      A       SRCIENEILM 
  gi|30021993|BC3904|NP_83    102  9.47e-12 
    TIGLIKAVNT     F      N      P      E      K      K      I      K      L      A      T      Y      A       SRCIENEILM 
  gi|162382776|BALH_3534|Y    102  9.47e-12 
    TIGLIKAVNT     F      N      P      E      K      K      I      K      L      A      T      Y      A       SRCIENEILM 
  gi|218234749|BCB4264_A40    102  9.47e-12 
    TIGLIKAVNT     F      N      P      E      K      K      I      K      L      A      T      Y      A       SRCIENEILM 
  gi|49186754|BAS3755|YP_0    102  9.47e-12 
    TIGLIKAVNT     F      N      P      E      K      K      I      K      L      A      T      Y      A       SRCIENEILM 
  gi|163941644|BcerKBAB4_3    102  9.47e-12 
    TIGLIKAVNT     F      N      P      E      K      K      I      K      L      A      T      Y      A       SRCIENEILM 
  gi|49478442|BT9727_3646|    102  9.47e-12 
    TIGLIKAVNT     F      N      P      E      K      K      I      K      L      A      T      Y      A       SRCIENEILM 
  gi|56419662|GK1127|YP_14    102  9.47e-12 
    TIGLIKAVNT     F      N      P      E      K      K      I      K      L      A      T      Y      A       SRCIENEILM 
  gi|294501014|BMQ_4270|YP    102  9.47e-12 
    TIGLIKAVNT     F      N      P      E      K      K      I      K      L      A      T      Y      A       SRCIENEILM 
  gi|157692207|BPUM_1426|Y    102  9.47e-12 
    TIGLIKAVNT     F      N      P      E      K      K      I      K      L      A      T      Y      A       SRCIENEILM 
  gi|15615119|BH2556|NP_24    100  9.47e-12 
    TIGLIKAVNT     F      N      P      E      K      K      I      K      L      A      T      Y      A       SRCIENEILM 
  gi|296504398|BMB171_C356    102  9.47e-12 
    TIGLIKAVNT     F      N      P      E      K      K      I      K      L      A      T      Y      A       SRCIENEILM 
  gi|52785509|BLi01750|YP_    102  9.47e-12 
    TIGLIKAVNT     F      N      P      E      K      K      I      K      L      A      T      Y      A       SRCIENEILM 
  gi|154685948|RBAM_015150    102  9.47e-12 
    TIGLIKAVNT     F      N      P      E      K      K      I      K      L      A      T      Y      A       SRCIENEILM 
  gi|52080135|BL02255|YP_0    102  9.47e-12 
    TIGLIKAVNT     F      N      P      E      K      K      I      K      L      A      T      Y      A       SRCIENEILM 
  gi|288553151|BpOF4_00625    100  9.47e-12 
    TIGLIKAVNT     F      N      P      E      K      K      I      K      L      A      T      Y      A       SRCIENEILM 
  gi|138894662|GTNG_0992|Y    102  9.47e-12 
    TIGLIKAVNT     F      N      P      E      K      K      I      K      L      A      T      Y      A       SRCIENEILM 
  gi|23098930|OB1475|NP_69    102  9.47e-12 
    TIGLIKAVNT     F      N      P      E      K      K      I      K      L      A      T      Y      A       SRCIENEILM 
  gi|239826532|GWCH70_1030    102  9.47e-12 
    TIGLIKAVNT     F      N      P      E      K      K      I      K      L      A      T      Y      A       SRCIENEILM 
  gi|56964116|ABC2351|YP_1    100  9.47e-12 
    TIGLIKAVNT     F      N      P      E      K      K      I      K      L      A      T      Y      A       SRCIENEILM 
  gi|297530706|GC56T3_2446    102  9.47e-12 
    TIGLIKAVNT     F      N      P      E      K      K      I      K      L      A      T      Y      A       SRCIENEILM 
  gi|295706363|BMD_4258|YP    102  9.47e-12 
    TIGLIKAVNT     F      N      P      E      K      K      I      K      L      A      T      Y      A       SRCIENEILM 
  gi|261419324|GYMC61_1900    102  9.47e-12 
    TIGLIKAVNT     F      N      P      E      K      K      I      K      L      A      T      Y      A       SRCIENEILM 
  gi|301055395|BACI_c38600    101  9.47e-12 
    TIGLIKAVNT     F      N      P      E      K      K      I      K      L      A      T      Y      A       SRCIENEILM 
  gi|212638647|Aflv_0804|Y    94  1.06e-11 
    TIGLIKAIES     Y      S      T      G      K      G      T      K      L      A      T      Y      A       ARCIENEIFM 
  gi|15613848|BH1285|NP_24    94  2.30e-11 
    TIGLIKAIES     F      S      E      G      K      G      T      K      L      A      T      Y      A       ARCIENEILM 
  gi|169827020|Bsph_1444|Y    81  2.56e-11 
    CIGLMKAIDH     F      D      L      K      H      N      V      R      F      S      T      Y      A       VPMIIGEIRR 
  gi|294501329|BMQ_4591|YP    94  2.85e-11 
    TIGLIKAIES     Y      S      Q      G      K      G      T      K      L      A      T      Y      A       ARCIENEILM 
  gi|295706676|BMD_4577|YP    94  2.85e-11 
    TIGLIKAIES     Y      S      Q      G      K      G      T      K      L      A      T      Y      A       ARCIENEILM 
  gi|138897001|GTNG_3372|Y    75  3.16e-11 
    RLSFLRLLEH     Y      D      P      N      Q      G      T      S      F      E      S      Y      A       MKSLIGYMKN 
  gi|288553039|BpOF4_00065    71  3.16e-11 
    MLGLYDALEK     F      N      P      N      R      D      L      K      F      D      T      Y      A       SFRVRGAIID 
  gi|157693076|BPUM_2309|Y    142  3.16e-11 
    TIGLIKAIES     Y      S      S      G      K      G      T      K      L      A      T      Y      A       ARCIENEILM 
  gi|16079402|BSU23450|NP_    77  3.50e-11 
    CIGLLKSVDK     F      D      L      T      Y      D      V      R      F      S      T      Y      A       VPMIIGEIQR 
  gi|23099454|OB1999|NP_69    94  3.50e-11 
    TIGLIKGVES     F      S      T      D      K      G      T      K      L      A      T      Y      A       ARCIENEILM 
  gi|296506459|BMB171_P007    114  3.87e-11 
    NEGMLRAMED     F      N      P      D      L      G      Y      C      F      S      T      Y      A       FWWIQKSMLG 
  gi|67078088|pE33L466_021    114  3.87e-11 
    NEGMLRAMED     F      N      P      D      L      G      Y      C      F      S      T      Y      A       FWWIKKRMLG 
  gi|261420836|GYMC61_3488    73  4.74e-11 
    RLSFLRLLDH     Y      D      P      K      Q      G      T      S      F      E      S      Y      A       MKSLIGYMKN 
  gi|297531625|GC56T3_3410    73  4.74e-11 
    RLSFLRLLDH     Y      D      P      K      Q      G      T      S      F      E      S      Y      A       MKSLIGYMKN 
  gi|288555607|BpOF4_12995    94  4.74e-11 
    TIGLIKAIES     Y      S      D      G      K      G      T      K      L      A      T      Y      A       ARCIENEILM 
  gi|56963382|ABC1617|YP_1    94  5.23e-11 
    TIGLIKAIES     Y      S      E      G      K      G      T      K      L      A      T      Y      A       ARCIENEILM 
  gi|295695806|Btus_1170|Y    94  5.77e-11 
    TIGLIKAIES     F      Q      P      H      K      G      T      K      L      A      T      Y      A       ARCIENEILM 
  gi|15614994|BH2431|NP_24    71  7.00e-11 
    MLGLLDALEK     F      N      P      D      R      D      L      K      F      D      T      Y      A       SFRVRGAILD 
  gi|297582861|Bsel_0539|Y    81  7.00e-11 
    MIGLLAALRR     F      D      P      S      F      G      R      S      F      E      S      F      A       VPTIVGEIKR 
  gi|172057862|Exig_1853|Y    67  9.31e-11 
    MMGLYDALQK     F      D      Q      N      H      N      N      K      F      D      T      Y      A       AFRIRGAILD 
  gi|261405539|GYMC10_1690    94  1.12e-10 
    TIGLIKAIES     F      R      P      N      K      G      T      K      L      A      T      F      A       ARCIENEILM 
  gi|169827019|Bsph_1443|Y    66  1.23e-10 
    SIGLIKAIET     F      N      T      D      K      N      I      K      L      A      T      Y      A       SRCIENEILM 
  gi|56421959|GK3424|YP_14    73  1.47e-10 
    RLSFLRLLDH     Y      D      P      S      Q      G      A      S      F      E      S      Y      A       MKSLIGYMKN 
  gi|218232670|BCB4264_A10    74  1.47e-10 
    MLGLLGAIRR     Y      D      Y      S      I      G      N      A      F      E      P      F      A       IPTIVGEIKK 
  gi|218896055|BCG9842_B42    74  1.47e-10 
    MLGLLGAIRR     Y      D      Y      S      I      G      N      A      F      E      P      F      A       IPTIVGEIKK 
  gi|225862940|BCA_1029|YP    74  1.47e-10 
    MLGLLGAIRR     Y      D      Y      S      I      G      N      A      F      E      P      F      A       IPTIVGEIKK 
  gi|227816152|BAMEG_3579|    74  1.47e-10 
    MLGLLGAIRR     Y      D      Y      S      I      G      N      A      F      E      P      F      A       IPTIVGEIKK 
  gi|229604478|BAA_1086|YP    74  1.47e-10 
    MLGLLGAIRR     Y      D      Y      S      I      G      N      A      F      E      P      F      A       IPTIVGEIKK 
  gi|30261116|BA0992|NP_84    74  1.47e-10 
    MLGLLGAIRR     Y      D      Y      S      I      G      N      A      F      E      P      F      A       IPTIVGEIKK 
  gi|47526271|GBAA0992|YP_    74  1.47e-10 
    MLGLLGAIRR     Y      D      Y      S      I      G      N      A      F      E      P      F      A       IPTIVGEIKK 
  gi|118476614|BALH_0889|Y    75  1.47e-10 
    MLGLLGAIRR     Y      D      Y      S      I      G      N      A      F      E      P      F      A       IPTIVGEIKK 
  gi|52144331|BCZK0896|YP_    75  1.47e-10 
    MLGLLGAIRR     Y      D      Y      S      I      G      N      A      F      E      P      F      A       IPTIVGEIKK 
  gi|218902186|BCAH820_106    74  1.47e-10 
    MLGLLGAIRR     Y      D      Y      S      I      G      N      A      F      E      P      F      A       IPTIVGEIKK 
  gi|30019159|BC1004|NP_83    75  1.47e-10 
    MLGLLGAIRR     Y      D      Y      S      I      G      N      A      F      E      P      F      A       IPTIVGEIKK 
  gi|222094730|BCQ_1068|YP    74  1.47e-10 
    MLGLLGAIRR     Y      D      Y      S      I      G      N      A      F      E      P      F      A       IPTIVGEIKK 
  gi|217958581|BCAH187_A11    74  1.47e-10 
    MLGLLGAIRR     Y      D      Y      S      I      G      N      A      F      E      P      F      A       IPTIVGEIKK 
  gi|49480184|BT9727_0913|    75  1.47e-10 
    MLGLLGAIRR     Y      D      Y      S      I      G      N      A      F      E      P      F      A       IPTIVGEIKK 
  gi|49183950|BAS0928|YP_0    75  1.47e-10 
    MLGLLGAIRR     Y      D      Y      S      I      G      N      A      F      E      P      F      A       IPTIVGEIKK 
  gi|42780162|BCE_1086|NP_    74  1.47e-10 
    MLGLLGAIRR     Y      D      Y      S      I      G      N      A      F      E      P      F      A       IPTIVGEIKK 
  gi|163938900|BcerKBAB4_0    74  1.47e-10 
    MLGLLGAIRR     Y      D      Y      S      I      G      N      A      F      E      P      F      A       IPTIVGEIKK 
  gi|301052635|BACI_c10250    75  1.47e-10 
    MLGLLGAIRR     Y      D      Y      S      I      G      N      A      F      E      P      F      A       IPTIVGEIKK 
  gi|296501716|BMB171_C087    74  1.47e-10 
    MLGLLGAIRR     Y      D      Y      S      I      G      N      A      F      E      P      F      A       IPTIVGEIKK 
  gi|229917800|EAT1b_2078|    75  1.76e-10 
    MIGLLAALRR     F      D      P      E      F      G      R      S      F      E      S      F      A       VPTIIGEIKR 
  gi|152976319|Bcer98_2607    64  4.11e-10 
    MIALIKAREQ     F      K      P      E      M      G      F      K      F      L      T      F      A       VPKIIGEIGN 
  gi|261407955|GYMC10_4162    73  4.46e-10 
    VMGLIDAIEK     F      D      Y      K      R      G      L      Q      F      E      T      Y      A       SWRVRGAILD 
  gi|218848158|BCG9842_003    89  4.46e-10 
    TIGLMKGLED     Y      D      Y      T      K      G      N      K      L      S      T      Y      I       TWWIRQRISK 
  gi|23098085|OB0630|NP_69    75  5.24e-10 
    MIGLLAAVRR     Y      D      P      T      Y      G      K      S      F      E      S      F      A       IPTIIGEIKR 
  gi|295696453|Btus_1847|Y    105  5.68e-10 
    TIGLIKAVNT     F      D      P      Q      K      R      I      K      L      A      T      Y      A       SRCIENEILM 
  gi|169829726|Bsph_4295|Y    74  6.15e-10 
    MLGLLGAIRR     F      D      P      N      V      G      R      S      F      E      A      F      A       VPTIVGEIKR 
  gi|169827149|Bsph_1579|Y    86  6.15e-10 
    MIGLFDALNK     F      D      I      N      R      D      L      K      F      D      T      Y      A       SFRVRGAIID 
  gi|154684976|RBAM_005070    74  6.66e-10 
    MIGLLGAIKR     Y      D      P      E      V      G      K      S      F      E      A      F      A       IPTIIGEIKR 
  gi|15613092|BH0529|NP_24    74  6.66e-10 
    MVGLLAAFER     Y      D      P      K      Y      G      R      S      F      E      S      F      A       VPTIIGEIKR 
  gi|294497070|BMQ_0235|YP    74  6.66e-10 
    MLGLLGAIRR     Y      D      A      S      F      G      R      S      F      E      S      F      A       VPTIIGEIKR 
  gi|295702435|BMD_0229|YP    74  6.66e-10 
    MLGLLGAIRR     Y      D      A      S      F      G      R      S      F      E      S      F      A       VPTIIGEIKR 
  gi|30263387|BA3483|NP_84    72  7.78e-10 
    LFAFNEAIEQ     Y      S      Y      K      K      G      K      S      F      L      A      F      A       DLLIKRDVID 
  gi|118478695|BALH_3081|Y    80  7.78e-10 
    LFAFNEAIEK     Y      S      Y      K      K      G      K      S      F      L      A      F      A       DLLIKRDVID 
  gi|49481728|BT9727_3204|    72  7.78e-10 
    LFAFNEAIEQ     Y      S      Y      K      K      G      K      S      F      L      A      F      A       DLLIKRDVID 
  gi|222096892|BCQ_3232|YP    67  7.78e-10 
    LFAFNEAIEQ     Y      S      Y      K      K      G      K      S      F      L      A      F      A       DLLIKRDVID 
  gi|52142105|BCZK3137|YP_    72  7.78e-10 
    LFAFNEAIEQ     Y      S      Y      K      K      G      K      S      F      L      A      F      A       DLLIKRDVID 
  gi|42782494|BCE_3441|NP_    67  7.78e-10 
    LFAFNEAIEQ     Y      S      Y      K      K      G      K      S      F      L      A      F      A       DLLIKRDVID 
  gi|163941043|BcerKBAB4_3    72  7.78e-10 
    LFAFNEAIEQ     Y      S      Y      K      K      G      K      S      F      L      A      F      A       DLLIKRDVID 
  gi|225865393|BCA_3504|YP    71  7.78e-10 
    LFAFNEAIEK     Y      S      Y      K      K      G      K      S      F      L      A      F      A       DLLIKRDVID 
  gi|49186236|BAS3231|YP_0    72  7.78e-10 
    LFAFNEAIEQ     Y      S      Y      K      K      G      K      S      F      L      A      F      A       DLLIKRDVID 
  gi|218904561|BCAH820_344    67  7.78e-10 
    LFAFNEAIEQ     Y      S      Y      K      K      G      K      S      F      L      A      F      A       DLLIKRDVID 
  gi|47778165|GBAA3483|YP_    72  7.78e-10 
    LFAFNEAIEQ     Y      S      Y      K      K      G      K      S      F      L      A      F      A       DLLIKRDVID 
  gi|227813739|BAMEG_1143|    67  7.78e-10 
    LFAFNEAIEQ     Y      S      Y      K      K      G      K      S      F      L      A      F      A       DLLIKRDVID 
  gi|229601980|BAA_3517|YP    67  7.78e-10 
    LFAFNEAIEQ     Y      S      Y      K      K      G      K      S      F      L      A      F      A       DLLIKRDVID 
  gi|301054926|BACI_c33840    72  7.78e-10 
    LFAFNEAIEQ     Y      S      Y      K      K      G      K      S      F      L      A      F      A       DLLIKRDVID 
  gi|218231054|BCB4264_A34    71  9.80e-10 
    LFAFNEAIEQ     Y      S      Y      T      K      G      K      S      F      L      A      F      A       DLLIKRDVID 
  gi|218898488|BCG9842_B18    71  9.80e-10 
    LFAFNEAIEQ     Y      S      Y      T      K      G      K      S      F      L      A      F      A       DLLIKRDVID 
  gi|30021529|BC3426|NP_83    71  9.80e-10 
    LFAFNEAIEQ     Y      S      Y      T      K      G      K      S      F      L      A      F      A       DLLIKRDVID 
  gi|296503941|BMB171_C311    67  9.80e-10 
    LFAFNEAIEQ     Y      S      Y      T      K      G      K      S      F      L      A      F      A       DLLIKRDVID 
  gi|52784380|BLi00560|YP_    75  1.33e-09 
    MLGLLGAIKR     Y      D      P      D      V      G      K      S      F      E      A      F      A       IPTIIGEIKR 
  gi|52079008|BL02208|YP_0    74  1.33e-09 
    MLGLLGAIKR     Y      D      P      D      V      G      K      S      F      E      A      F      A       IPTIIGEIKR 
  gi|227813117|BAMEG_0515|    71  2.74e-09 
    MLGLVTARRD     F      K      T      E      L      G      F      K      F      T      T      F      A       VPRILGYIRK 
  gi|229600567|BAA_4139|YP    71  2.74e-09 
    MLGLVTARRD     F      K      T      E      L      G      F      K      F      T      T      F      A       VPRILGYIRK 
  gi|47529410|GBAA4115|YP_    56  2.74e-09 
    MLGLVTARRD     F      K      T      E      L      G      F      K      F      T      T      F      A       VPRILGYIRK 
  gi|30263977|BA4115|NP_84    56  2.74e-09 
    MLGLVTARRD     F      K      T      E      L      G      F      K      F      T      T      F      A       VPRILGYIRK 
  gi|49186821|BAS3823|YP_0    71  2.74e-09 
    MLGLVTARRD     F      K      T      E      L      G      F      K      F      T      T      F      A       VPRILGYIRK 
  gi|288554854|BpOF4_09205    74  2.74e-09 
    MVGLLAALTR     F      D      S      E      F      G      R      S      F      E      S      F      A       VPTIVGEIKR 
  gi|169829306|Bsph_3856|Y    87  4.47e-09 
    TIGLMKAVES     Y      T      P      D      K      K      T      R      L      A      T      Y      A       ARCIENEILM 
  gi|255767137|BSU04730|NP    74  4.79e-09 
    MIGLLGAIKR     Y      D      P      V      V      G      K      S      F      E      A      F      A       IPTIIGEIKR 
  gi|261409324|GYMC10_5549    74  5.49e-09 
    QMALIRLLQQ     Y      D      I      S      L      G      I      P      F      E      P      Y      A       MKSMIGHMKN 
  gi|172058807|Exig_2804|Y    75  5.87e-09 
    MIGLLAALRR     F      D      K      E      F      G      R      S      F      E      S      F      A       VPTIIGEIKR 
  gi|157691239|BPUM_0446|Y    74  6.28e-09 
    MIGLLGAIKR     Y      D      P      L      V      G      K      S      F      E      A      F      A       IPTIIGEIKR 
  gi|56964009|ABC2244|YP_1    66  6.28e-09 
    LSGLLDAIAK     F      D      S      K      R      A      L      K      F      D      T      Y      A       SFRIRGAIID 
  gi|138894489|GTNG_0819|Y    72  8.20e-09 
    LIAFNEAIEK     Y      A      P      H      K      G      G      S      F      L      S      F      A       ELLIKRRLID 
  gi|297584063|Bsel_1770|Y    72  1.07e-08 
    LMGLFDALEK     F      D      R      K      R      E      L      K      F      D      T      Y      A       SFRIRGAIID 
  gi|56419467|GK0932|YP_14    72  1.90e-08 
    LIAFNEAIEK     Y      A      L      H      K      G      G      S      F      L      S      F      A       ELLIKRRLID 
  gi|56962590|ABC0816|YP_1    74  2.45e-08 
    MVGLLAAMKR     Y      N      E      D      Y      G      R      S      F      E      S      F      A       VPTVVGEIKR 
  gi|295696140|Btus_1522|Y    54  2.45e-08 
    MLGLLEAARR     Y      D      P      G      Q      E      T      P      F      E      V      F      A       RLRIRGAMID 
  gi|239826355|GWCH70_0832    73  3.77e-08 
    LIAFNEAIEK     Y      A      P      H      K      G      G      S      F      I      S      F      A       ELLIKRRLID 
  gi|56964615|ABC2851|YP_1    54  8.21e-08 
    TIGLINAVEN     Y      E      N      K      K      G      I      T      F      A      A      Y      A       VPVIRRSMLK 
  gi|295695629|Btus_0984|Y    76  1.10e-07 
    LAAFNEAIDR     Y      D      G      N      R      G      A      S      F      L      G      F      A       DTVIRRRLID 
  gi|297585316|Bsel_3049|Y    54  8.92e-05 
    GMVALFDAWQ     R      F      D      G      A      K      G      A      F      P      S      Y      V       RLYIHGRMCN 
 
    
      	Motif 2 block diagrams  
    
 
  Name Lowest p-value    Motifs
 
     gi|261407775|GYMC10_3981
  1.9e-16
    
    
   2
    
   
  
 
     gi|218232261|BCB4264_A44
  2.3e-15
    
    
   2
    
   
  
 
     gi|52141207|BCZK4042|YP_
  2.3e-15
    
    
   2
    
   
  
 
     gi|42783418|BCE_4372|NP_
  2.3e-15
    
    
   2
    
   
  
 
     gi|30264362|BA4515|NP_84
  2.3e-15
    
    
   2
    
   
  
 
     gi|222097738|BCQ_4079|YP
  2.3e-15
    
    
   2
    
   
  
 
     gi|218899458|BCG9842_B08
  2.3e-15
    
    
   2
    
   
  
 
     gi|218905429|BCAH820_431
  2.3e-15
    
    
   2
    
   
  
 
     gi|229601154|BAA_4536|YP
  2.3e-15
    
    
   2
    
   
  
 
     gi|30022370|BC4289|NP_83
  2.3e-15
    
    
   2
    
   
  
 
     gi|152976724|Bcer98_3019
  2.3e-15
    
    
   2
    
   
  
 
     gi|163942050|BcerKBAB4_4
  2.3e-15
    
    
   2
    
   
  
 
     gi|118479464|BALH_3885|Y
  2.3e-15
    
    
   2
    
   
  
 
     gi|217961783|BCAH187_A44
  2.3e-15
    
    
   2
    
   
  
 
     gi|47529811|GBAA4515|YP_
  2.3e-15
    
    
   2
    
   
  
 
     gi|49187190|BAS4194|YP_0
  2.3e-15
    
    
   2
    
   
  
 
     gi|225866273|BCA_4403|YP
  2.3e-15
    
    
   2
    
   
  
 
     gi|227817068|BAMEG_4554|
  2.3e-15
    
    
   2
    
   
  
 
     gi|49481307|BT9727_4032|
  2.3e-15
    
    
   2
    
   
  
 
     gi|169829158|Bsph_3702|Y
  2.3e-15
    
    
   2
    
   
  
 
     gi|301055785|BACI_c42620
  2.3e-15
    
    
   2
    
   
  
 
     gi|23099399|OB1944|NP_69
  2.3e-15
    
    
   2
    
   
  
 
     gi|296504786|BMB171_C395
  2.3e-15
    
    
   2
    
   
  
 
     gi|297584635|Bsel_2346|Y
  3.7e-15
    
    
   2
    
   
  
 
     gi|15613939|BH1376|NP_24
  3.7e-15
    
    
   2
    
   
  
 
     gi|261405623|GYMC10_1774
  9.2e-15
    
    
   2
    
   
  
 
     gi|56421017|GK2482|YP_14
  1.4e-14
    
    
   2
    
   
  
 
     gi|255767573|BSU25200|NP
  1.4e-14
    
    
   2
    
   
  
 
     gi|212638699|Aflv_0856|Y
  1.4e-14
    
    
   2
    
   
  
 
     gi|295706627|BMD_4528|YP
  1.4e-14
    
    
   2
    
   
  
 
     gi|297529352|GC56T3_1008
  1.4e-14
    
    
   2
    
   
  
 
     gi|261417657|GYMC61_0157
  1.4e-14
    
    
   2
    
   
  
 
     gi|239827752|GWCH70_2414
  1.4e-14
    
    
   2
    
   
  
 
     gi|157693020|BPUM_2253|Y
  1.4e-14
    
    
   2
    
   
  
 
     gi|154686781|RBAM_023510
  1.4e-14
    
    
   2
    
   
  
 
     gi|163119552|BL03682|YP_
  1.4e-14
    
    
   2
    
   
  
 
     gi|52786447|BLi02712|YP_
  1.4e-14
    
    
   2
    
   
  
 
     gi|294501280|BMQ_4542|YP
  1.4e-14
    
    
   2
    
   
  
 
     gi|138896056|GTNG_2419|Y
  1.4e-14
    
    
   2
    
   
  
 
     gi|288555754|BpOF4_13730
  1.8e-14
    
    
   2
    
   
  
 
     gi|56963455|ABC1690|YP_1
  2.2e-14
    
    
   2
    
   
  
 
     gi|212639646|Aflv_1820|Y
  3.9e-14
    
    
   2
    
   
  
 
     gi|42782996|BCE_3948|NP_
  3.9e-14
    
    
   2
    
   
  
 
     gi|218233406|BCB4264_A40
  3.9e-14
    
    
   2
    
   
  
 
     gi|227813187|BAMEG_0585|
  3.9e-14
    
    
   2
    
   
  
 
     gi|163941643|BcerKBAB4_3
  3.9e-14
    
    
   2
    
   
  
 
     gi|152976264|Bcer98_2552
  3.9e-14
    
    
   2
    
   
  
 
     gi|49478441|BT9727_3645|
  3.9e-14
    
    
   2
    
   
  
 
     gi|30263905|BA4042|NP_84
  3.9e-14
    
    
   2
    
   
  
 
     gi|217961324|BCAH187_A39
  3.9e-14
    
    
   2
    
   
  
 
     gi|229602177|BAA_4068|YP
  3.9e-14
    
    
   2
    
   
  
 
     gi|30021992|BC3903|NP_83
  3.9e-14
    
    
   2
    
   
  
 
     gi|218905033|BCAH820_391
  3.9e-14
    
    
   2
    
   
  
 
     gi|225865885|BCA_4007|YP
  3.9e-14
    
    
   2
    
   
  
 
     gi|47778237|GBAA4042|YP_
  3.9e-14
    
    
   2
    
   
  
 
     gi|222097349|BCQ_3689|YP
  3.9e-14
    
    
   2
    
   
  
 
     gi|49186753|BAS3754|YP_0
  3.9e-14
    
    
   2
    
   
  
 
     gi|218899057|BCG9842_B12
  3.9e-14
    
    
   2
    
   
  
 
     gi|52141585|BCZK3662|YP_
  3.9e-14
    
    
   2
    
   
  
 
     gi|118479123|BALH_3533|Y
  3.9e-14
    
    
   2
    
   
  
 
     gi|301055394|BACI_c38590
  3.9e-14
    
    
   2
    
   
  
 
     gi|288553150|BpOF4_00620
  3.9e-14
    
    
   2
    
   
  
 
     gi|295706362|BMD_4257|YP
  3.9e-14
    
    
   2
    
   
  
 
     gi|56964115|ABC2350|YP_1
  3.9e-14
    
    
   2
    
   
  
 
     gi|295696452|Btus_1846|Y
  3.9e-14
    
    
   2
    
   
  
 
     gi|296504397|BMB171_C356
  3.9e-14
    
    
   2
    
   
  
 
     gi|261405674|GYMC10_1825
  3.9e-14
    
    
   2
    
   
  
 
     gi|294501013|BMQ_4269|YP
  3.9e-14
    
    
   2
    
   
  
 
     gi|15615117|BH2554|NP_24
  3.9e-14
    
    
   2
    
   
  
 
     gi|218905210|BCAH820_409
  5.8e-14
    
    
   2
    
   
  
 
     gi|217961566|BCAH187_A42
  5.8e-14
    
    
   2
    
   
  
 
     gi|52141420|BCZK3829|YP_
  5.8e-14
    
    
   2
    
   
  
 
     gi|42783188|BCE_4142|NP_
  5.8e-14
    
    
   2
    
   
  
 
     gi|47529588|GBAA4294|YP_
  5.8e-14
    
    
   2
    
   
  
 
     gi|227816852|BAMEG_4334|
  5.8e-14
    
    
   2
    
   
  
 
     gi|118479276|BALH_3690|Y
  5.8e-14
    
    
   2
    
   
  
 
     gi|225866059|BCA_4185|YP
  5.8e-14
    
    
   2
    
   
  
 
     gi|218899234|BCG9842_B10
  5.8e-14
    
    
   2
    
   
  
 
     gi|30264150|BA4294|NP_84
  5.8e-14
    
    
   2
    
   
  
 
     gi|49478518|BT9727_3813|
  5.8e-14
    
    
   2
    
   
  
 
     gi|218234550|BCB4264_A41
  5.8e-14
    
    
   2
    
   
  
 
     gi|222097523|BCQ_3863|YP
  5.8e-14
    
    
   2
    
   
  
 
     gi|49186981|BAS3983|YP_0
  5.8e-14
    
    
   2
    
   
  
 
     gi|30022159|BC4072|NP_83
  5.8e-14
    
    
   2
    
   
  
 
     gi|163941815|BcerKBAB4_3
  5.8e-14
    
    
   2
    
   
  
 
     gi|152976482|Bcer98_2771
  5.8e-14
    
    
   2
    
   
  
 
     gi|229603990|BAA_4316|YP
  5.8e-14
    
    
   2
    
   
  
 
     gi|301055569|BACI_c40400
  5.8e-14
    
    
   2
    
   
  
 
     gi|296504567|BMB171_C373
  5.8e-14
    
    
   2
    
   
  
 
     gi|172056869|Exig_0832|Y
  8.3e-14
    
    
   2
    
   
  
 
     gi|212638845|Aflv_1004|Y
  9.9e-14
    
    
   2
    
   
  
 
     gi|16078597|BSU15330|NP_
  9.9e-14
    
    
   2
    
   
  
 
     gi|56420843|GK2308|YP_14
  9.9e-14
    
    
   2
    
   
  
 
     gi|288555973|BpOF4_14835
  9.9e-14
    
    
   2
    
   
  
 
     gi|157692844|BPUM_2076|Y
  9.9e-14
    
    
   2
    
   
  
 
     gi|169827282|Bsph_1714|Y
  9.9e-14
    
    
   2
    
   
  
 
     gi|15614101|BH1538|NP_24
  9.9e-14
    
    
   2
    
   
  
 
     gi|294501135|BMQ_4391|YP
  9.9e-14
    
    
   2
    
   
  
 
     gi|52785510|BLi01751|YP_
  9.9e-14
    
    
   2
    
   
  
 
     gi|295706482|BMD_4377|YP
  9.9e-14
    
    
   2
    
   
  
 
     gi|56963560|ABC1795|YP_1
  9.9e-14
    
    
   2
    
   
  
 
     gi|154685949|RBAM_015160
  9.9e-14
    
    
   2
    
   
  
 
     gi|297529524|GC56T3_1195
  9.9e-14
    
    
   2
    
   
  
 
     gi|239827600|GWCH70_2250
  9.9e-14
    
    
   2
    
   
  
 
     gi|138895880|GTNG_2239|Y
  9.9e-14
    
    
   2
    
   
  
 
     gi|295696440|Btus_1834|Y
  9.9e-14
    
    
   2
    
   
  
 
     gi|261417856|GYMC61_0374
  9.9e-14
    
    
   2
    
   
  
 
     gi|23099294|OB1839|NP_69
  9.9e-14
    
    
   2
    
   
  
 
     gi|261405990|GYMC10_2143
  9.9e-14
    
    
   2
    
   
  
 
     gi|52080136|BL02256|YP_0
  9.9e-14
    
    
   2
    
   
  
 
     gi|229916320|EAT1b_0589|
  2e-13
    
    
   2
    
   
  
 
     gi|56419663|GK1128|YP_14
  2.7e-13
    
    
   2
    
   
  
 
     gi|297530705|GC56T3_2445
  2.7e-13
    
    
   2
    
   
  
 
     gi|239826533|GWCH70_1031
  2.7e-13
    
    
   2
    
   
  
 
     gi|138894663|GTNG_0993|Y
  2.7e-13
    
    
   2
    
   
  
 
     gi|261419325|GYMC61_1901
  2.7e-13
    
    
   2
    
   
  
 
     gi|56421070|GK2535|YP_14
  3.7e-13
    
    
   2
    
   
  
 
     gi|261418447|GYMC61_0982
  3.7e-13
    
    
   2
    
   
  
 
     gi|239827809|GWCH70_2471
  3.7e-13
    
    
   2
    
   
  
 
     gi|297529299|GC56T3_0955
  3.7e-13
    
    
   2
    
   
  
 
     gi|295695448|Btus_0783|Y
  1.4e-12
    
    
   2
    
   
  
 
     gi|138896107|GTNG_2470|Y
  1.9e-12
    
    
   2
    
   
  
 
     gi|56419781|GK1246|YP_14
  3.6e-12
    
    
   2
    
   
  
 
     gi|212639538|Aflv_1712|Y
  3.6e-12
    
    
   2
    
   
  
 
     gi|16078710|BSU16470|NP_
  3.6e-12
    
    
   2
    
   
  
 
     gi|138894766|GTNG_1100|Y
  3.6e-12
    
    
   2
    
   
  
 
     gi|261419446|GYMC61_2030
  3.6e-12
    
    
   2
    
   
  
 
     gi|261405673|GYMC10_1824
  3.6e-12
    
    
   2
    
   
  
 
     gi|154686064|RBAM_016310
  3.6e-12
    
    
   2
    
   
  
 
     gi|157692327|BPUM_1546|Y
  3.6e-12
    
    
   2
    
   
  
 
     gi|23099037|OB1582|NP_69
  3.6e-12
    
    
   2
    
   
  
 
     gi|52785627|BLi01868|YP_
  3.6e-12
    
    
   2
    
   
  
 
     gi|52080250|BL01246|YP_0
  3.6e-12
    
    
   2
    
   
  
 
     gi|297530579|GC56T3_2307
  3.6e-12
    
    
   2
    
   
  
 
     gi|239826640|GWCH70_1138
  3.6e-12
    
    
   2
    
   
  
 
     gi|229603939|BAA_4584|YP
  4.6e-12
    
    
   2
    
   
  
 
     gi|49481469|BT9727_4074|
  4.6e-12
    
    
   2
    
   
  
 
     gi|49187232|BAS4236|YP_0
  4.6e-12
    
    
   2
    
   
  
 
     gi|222097784|BCQ_4125|YP
  4.6e-12
    
    
   2
    
   
  
 
     gi|218905533|BCAH820_441
  4.6e-12
    
    
   2
    
   
  
 
     gi|227817116|BAMEG_4602|
  4.6e-12
    
    
   2
    
   
  
 
     gi|217961829|BCAH187_A44
  4.6e-12
    
    
   2
    
   
  
 
     gi|225866320|BCA_4450|YP
  4.6e-12
    
    
   2
    
   
  
 
     gi|163942095|BcerKBAB4_4
  4.6e-12
    
    
   2
    
   
  
 
     gi|218231149|BCB4264_A44
  4.6e-12
    
    
   2
    
   
  
 
     gi|218899507|BCG9842_B07
  4.6e-12
    
    
   2
    
   
  
 
     gi|118479504|BALH_3926|Y
  4.6e-12
    
    
   2
    
   
  
 
     gi|30022417|BC4336|NP_83
  4.6e-12
    
    
   2
    
   
  
 
     gi|152976769|Bcer98_3065
  4.6e-12
    
    
   2
    
   
  
 
     gi|30264411|BA4566|NP_84
  4.6e-12
    
    
   2
    
   
  
 
     gi|42783467|BCE_4421|NP_
  4.6e-12
    
    
   2
    
   
  
 
     gi|52141167|BCZK4084|YP_
  4.6e-12
    
    
   2
    
   
  
 
     gi|47529862|GBAA4566|YP_
  4.6e-12
    
    
   2
    
   
  
 
     gi|154686834|RBAM_024040
  4.6e-12
    
    
   2
    
   
  
 
     gi|157692208|BPUM_1427|Y
  4.6e-12
    
    
   2
    
   
  
 
     gi|52080862|BL00778|YP_0
  4.6e-12
    
    
   2
    
   
  
 
     gi|301055830|BACI_c43070
  4.6e-12
    
    
   2
    
   
  
 
     gi|23098931|OB1476|NP_69
  4.6e-12
    
    
   2
    
   
  
 
     gi|52786234|BLi02495|YP_
  4.6e-12
    
    
   2
    
   
  
 
     gi|52081127|BL02107|YP_0
  4.6e-12
    
    
   2
    
   
  
 
     gi|296504831|BMB171_C400
  4.6e-12
    
    
   2
    
   
  
 
     gi|52786504|BLi02769|YP_
  4.6e-12
    
    
   2
    
   
  
 
     gi|154686587|RBAM_021560
  4.6e-12
    
    
   2
    
   
  
 
     gi|229918653|EAT1b_2940|
  5.2e-12
    
    
   2
    
   
  
 
     gi|294500913|BMQ_4167|YP
  6.6e-12
    
    
   2
    
   
  
 
     gi|295706259|BMD_4154|YP
  6.6e-12
    
    
   2
    
   
  
 
     gi|255767354|BSU15320|NP
  9.5e-12
    
    
   2
    
   
  
 
     gi|227813186|BAMEG_0584|
  9.5e-12
    
    
   2
    
   
  
 
     gi|152976265|Bcer98_2553
  9.5e-12
    
    
   2
    
   
  
 
     gi|218899058|BCG9842_B12
  9.5e-12
    
    
   2
    
   
  
 
     gi|218905035|BCAH820_391
  9.5e-12
    
    
   2
    
   
  
 
     gi|30263906|BA4043|NP_84
  9.5e-12
    
    
   2
    
   
  
 
     gi|225865886|BCA_4008|YP
  9.5e-12
    
    
   2
    
   
  
 
     gi|47529336|GBAA4043|YP_
  9.5e-12
    
    
   2
    
   
  
 
     gi|212639647|Aflv_1821|Y
  9.5e-12
    
    
   2
    
   
  
 
     gi|52141583|BCZK3663|YP_
  9.5e-12
    
    
   2
    
   
  
 
     gi|42782997|BCE_3949|NP_
  9.5e-12
    
    
   2
    
   
  
 
     gi|217961326|BCAH187_A39
  9.5e-12
    
    
   2
    
   
  
 
     gi|222097350|BCQ_3690|YP
  9.5e-12
    
    
   2
    
   
  
 
     gi|229601447|BAA_4069|YP
  9.5e-12
    
    
   2
    
   
  
 
     gi|30021993|BC3904|NP_83
  9.5e-12
    
    
   2
    
   
  
 
     gi|162382776|BALH_3534|Y
  9.5e-12
    
    
   2
    
   
  
 
     gi|218234749|BCB4264_A40
  9.5e-12
    
    
   2
    
   
  
 
     gi|49186754|BAS3755|YP_0
  9.5e-12
    
    
   2
    
   
  
 
     gi|163941644|BcerKBAB4_3
  9.5e-12
    
    
   2
    
   
  
 
     gi|49478442|BT9727_3646|
  9.5e-12
    
    
   2
    
   
  
 
     gi|56419662|GK1127|YP_14
  9.5e-12
    
    
   2
    
   
  
 
     gi|294501014|BMQ_4270|YP
  9.5e-12
    
    
   2
    
   
  
 
     gi|157692207|BPUM_1426|Y
  9.5e-12
    
    
   2
    
   
  
 
     gi|15615119|BH2556|NP_24
  9.5e-12
    
    
   2
    
   
  
 
     gi|296504398|BMB171_C356
  9.5e-12
    
    
   2
    
   
  
 
     gi|52785509|BLi01750|YP_
  9.5e-12
    
    
   2
    
   
  
 
     gi|154685948|RBAM_015150
  9.5e-12
    
    
   2
    
   
  
 
     gi|52080135|BL02255|YP_0
  9.5e-12
    
    
   2
    
   
  
 
     gi|288553151|BpOF4_00625
  9.5e-12
    
    
   2
    
   
  
 
     gi|138894662|GTNG_0992|Y
  9.5e-12
    
    
   2
    
   
  
 
     gi|23098930|OB1475|NP_69
  9.5e-12
    
    
   2
    
   
  
 
     gi|239826532|GWCH70_1030
  9.5e-12
    
    
   2
    
   
  
 
     gi|56964116|ABC2351|YP_1
  9.5e-12
    
    
   2
    
   
  
 
     gi|297530706|GC56T3_2446
  9.5e-12
    
    
   2
    
   
  
 
     gi|295706363|BMD_4258|YP
  9.5e-12
    
    
   2
    
   
  
 
     gi|261419324|GYMC61_1900
  9.5e-12
    
    
   2
    
   
  
 
     gi|301055395|BACI_c38600
  9.5e-12
    
    
   2
    
   
  
 
     gi|212638647|Aflv_0804|Y
  1.1e-11
    
    
   2
    
   
  
 
     gi|15613848|BH1285|NP_24
  2.3e-11
    
    
   2
    
   
  
 
     gi|169827020|Bsph_1444|Y
  2.6e-11
    
    
   2
    
   
  
 
     gi|294501329|BMQ_4591|YP
  2.8e-11
    
    
   2
    
   
  
 
     gi|295706676|BMD_4577|YP
  2.8e-11
    
    
   2
    
   
  
 
     gi|138897001|GTNG_3372|Y
  3.2e-11
    
    
   2
    
   
  
 
     gi|288553039|BpOF4_00065
  3.2e-11
    
    
   2
    
   
  
 
     gi|157693076|BPUM_2309|Y
  3.2e-11
    
    
   2
    
   
  
 
     gi|16079402|BSU23450|NP_
  3.5e-11
    
    
   2
    
   
  
 
     gi|23099454|OB1999|NP_69
  3.5e-11
    
    
   2
    
   
  
 
     gi|296506459|BMB171_P007
  3.9e-11
    
    
   2
    
   
  
 
     gi|67078088|pE33L466_021
  3.9e-11
    
    
   2
    
   
  
 
     gi|261420836|GYMC61_3488
  4.7e-11
    
    
   2
    
   
  
 
     gi|297531625|GC56T3_3410
  4.7e-11
    
    
   2
    
   
  
 
     gi|288555607|BpOF4_12995
  4.7e-11
    
    
   2
    
   
  
 
     gi|56963382|ABC1617|YP_1
  5.2e-11
    
    
   2
    
   
  
 
     gi|295695806|Btus_1170|Y
  5.8e-11
    
    
   2
    
   
  
 
     gi|15614994|BH2431|NP_24
  7e-11
    
    
   2
    
   
  
 
     gi|297582861|Bsel_0539|Y
  7e-11
    
    
   2
    
   
  
 
     gi|172057862|Exig_1853|Y
  9.3e-11
    
    
   2
    
   
  
 
     gi|261405539|GYMC10_1690
  1.1e-10
    
    
   2
    
   
  
 
     gi|169827019|Bsph_1443|Y
  1.2e-10
    
    
   2
    
   
  
 
     gi|56421959|GK3424|YP_14
  1.5e-10
    
    
   2
    
   
  
 
     gi|218232670|BCB4264_A10
  1.5e-10
    
    
   2
    
   
  
 
     gi|218896055|BCG9842_B42
  1.5e-10
    
    
   2
    
   
  
 
     gi|225862940|BCA_1029|YP
  1.5e-10
    
    
   2
    
   
  
 
     gi|227816152|BAMEG_3579|
  1.5e-10
    
    
   2
    
   
  
 
     gi|229604478|BAA_1086|YP
  1.5e-10
    
    
   2
    
   
  
 
     gi|30261116|BA0992|NP_84
  1.5e-10
    
    
   2
    
   
  
 
     gi|47526271|GBAA0992|YP_
  1.5e-10
    
    
   2
    
   
  
 
     gi|118476614|BALH_0889|Y
  1.5e-10
    
    
   2
    
   
  
 
     gi|52144331|BCZK0896|YP_
  1.5e-10
    
    
   2
    
   
  
 
     gi|218902186|BCAH820_106
  1.5e-10
    
    
   2
    
   
  
 
     gi|30019159|BC1004|NP_83
  1.5e-10
    
    
   2
    
   
  
 
     gi|222094730|BCQ_1068|YP
  1.5e-10
    
    
   2
    
   
  
 
     gi|217958581|BCAH187_A11
  1.5e-10
    
    
   2
    
   
  
 
     gi|49480184|BT9727_0913|
  1.5e-10
    
    
   2
    
   
  
 
     gi|49183950|BAS0928|YP_0
  1.5e-10
    
    
   2
    
   
  
 
     gi|42780162|BCE_1086|NP_
  1.5e-10
    
    
   2
    
   
  
 
     gi|163938900|BcerKBAB4_0
  1.5e-10
    
    
   2
    
   
  
 
     gi|301052635|BACI_c10250
  1.5e-10
    
    
   2
    
   
  
 
     gi|296501716|BMB171_C087
  1.5e-10
    
    
   2
    
   
  
 
     gi|229917800|EAT1b_2078|
  1.8e-10
    
    
   2
    
   
  
 
     gi|152976319|Bcer98_2607
  4.1e-10
    
    
   2
    
   
  
 
     gi|261407955|GYMC10_4162
  4.5e-10
    
    
   2
    
   
  
 
     gi|218848158|BCG9842_003
  4.5e-10
    
    
   2
    
   
  
 
     gi|23098085|OB0630|NP_69
  5.2e-10
    
    
   2
    
   
  
 
     gi|295696453|Btus_1847|Y
  5.7e-10
    
    
   2
    
   
  
 
     gi|169829726|Bsph_4295|Y
  6.1e-10
    
    
   2
    
   
  
 
     gi|169827149|Bsph_1579|Y
  6.1e-10
    
    
   2
    
   
  
 
     gi|154684976|RBAM_005070
  6.7e-10
    
    
   2
    
   
  
 
     gi|15613092|BH0529|NP_24
  6.7e-10
    
    
   2
    
   
  
 
     gi|294497070|BMQ_0235|YP
  6.7e-10
    
    
   2
    
   
  
 
     gi|295702435|BMD_0229|YP
  6.7e-10
    
    
   2
    
   
  
 
     gi|30263387|BA3483|NP_84
  7.8e-10
    
    
   2
    
   
  
 
     gi|118478695|BALH_3081|Y
  7.8e-10
    
    
   2
    
   
  
 
     gi|49481728|BT9727_3204|
  7.8e-10
    
    
   2
    
   
  
 
     gi|222096892|BCQ_3232|YP
  7.8e-10
    
    
   2
    
   
  
 
     gi|52142105|BCZK3137|YP_
  7.8e-10
    
    
   2
    
   
  
 
     gi|42782494|BCE_3441|NP_
  7.8e-10
    
    
   2
    
   
  
 
     gi|163941043|BcerKBAB4_3
  7.8e-10
    
    
   2
    
   
  
 
     gi|225865393|BCA_3504|YP
  7.8e-10
    
    
   2
    
   
  
 
     gi|49186236|BAS3231|YP_0
  7.8e-10
    
    
   2
    
   
  
 
     gi|218904561|BCAH820_344
  7.8e-10
    
    
   2
    
   
  
 
     gi|47778165|GBAA3483|YP_
  7.8e-10
    
    
   2
    
   
  
 
     gi|227813739|BAMEG_1143|
  7.8e-10
    
    
   2
    
   
  
 
     gi|229601980|BAA_3517|YP
  7.8e-10
    
    
   2
    
   
  
 
     gi|301054926|BACI_c33840
  7.8e-10
    
    
   2
    
   
  
 
     gi|218231054|BCB4264_A34
  9.8e-10
    
    
   2
    
   
  
 
     gi|218898488|BCG9842_B18
  9.8e-10
    
    
   2
    
   
  
 
     gi|30021529|BC3426|NP_83
  9.8e-10
    
    
   2
    
   
  
 
     gi|296503941|BMB171_C311
  9.8e-10
    
    
   2
    
   
  
 
     gi|52784380|BLi00560|YP_
  1.3e-09
    
    
   2
    
   
  
 
     gi|52079008|BL02208|YP_0
  1.3e-09
    
    
   2
    
   
  
 
     gi|227813117|BAMEG_0515|
  2.7e-09
    
    
   2
    
   
  
 
     gi|229600567|BAA_4139|YP
  2.7e-09
    
    
   2
    
   
  
 
     gi|47529410|GBAA4115|YP_
  2.7e-09
    
    
   2
    
   
  
 
     gi|30263977|BA4115|NP_84
  2.7e-09
    
    
   2
    
   
  
 
     gi|49186821|BAS3823|YP_0
  2.7e-09
    
    
   2
    
   
  
 
     gi|288554854|BpOF4_09205
  2.7e-09
    
    
   2
    
   
  
 
     gi|169829306|Bsph_3856|Y
  4.5e-09
    
    
   2
    
   
  
 
     gi|255767137|BSU04730|NP
  4.8e-09
    
    
   2
    
   
  
 
     gi|261409324|GYMC10_5549
  5.5e-09
    
    
   2
    
   
  
 
     gi|172058807|Exig_2804|Y
  5.9e-09
    
    
   2
    
   
  
 
     gi|157691239|BPUM_0446|Y
  6.3e-09
    
    
   2
    
   
  
 
     gi|56964009|ABC2244|YP_1
  6.3e-09
    
    
   2
    
   
  
 
     gi|138894489|GTNG_0819|Y
  8.2e-09
    
    
   2
    
   
  
 
     gi|297584063|Bsel_1770|Y
  1.1e-08
    
    
   2
    
   
  
 
     gi|56419467|GK0932|YP_14
  1.9e-08
    
    
   2
    
   
  
 
     gi|56962590|ABC0816|YP_1
  2.4e-08
    
    
   2
    
   
  
 
     gi|295696140|Btus_1522|Y
  2.4e-08
    
    
   2
    
   
  
 
     gi|239826355|GWCH70_0832
  3.8e-08
    
    
   2
    
   
  
 
     gi|56964615|ABC2851|YP_1
  8.2e-08
    
    
   2
    
   
  
 
     gi|295695629|Btus_0984|Y
  1.1e-07
    
    
   2
    
   
  
 
     gi|297585316|Bsel_3049|Y
  8.9e-05
    
    
   2
    
   
  
  SCALE
     
     | 
     | 
     | 
     | 
     | 
     | 
     | 
     | 
     | 
     | 
     | 
     | 
     | 
     | 
     | 
    1 
     25 
     50 
     75 
     100 
     125 
     150 
     175 
     200 
     225 
     250 
     275 
     300 
     325 
     350 
   
 

    
      	Motif 2 in BLOCKS format  
    
 
 
 
 
  
 to  BLOCKS multiple alignment processor.  
    
      	Motif 2 position-specific scoring matrix  
    
 
 

    
      	Motif 2 position-specific probability matrix  
    
 
 

    
      	Motif 2 regular expression  
    
[FY]D[YPL][SG]KGVK[FL][SA]T[YF]A

 


Time 11415.77 secs.

 

    
   
     P  
     N        MOTIF  3       width = 19         sites = 271        llr = 9203        E-value = 6.0e-2558 
    
 
    Simplified  A  : : 6 : 1 : : : : : 2 : : : : : : 2 1
    pos.-specific  C  : : : : : : : : : : : : : : : : : : :
    probability  D  2 : : 2 : : : : : : : : : : : : : : :
    matrix  E  8 : : 4 2 : : : : : 2 : : : : : 8 : :
    F  : : : : : 2 : : : : : : : : : : : : :
    G  : : 4 : : : 9 : : : : : : : : : : : :
    H  : : : : : : : : : : : 1 : : : : 1 : :
    I  : 3 : : : 2 : 7 : : : : 4 : : 5 : : :
    K  : : : 3 : : : : : : : : : : : : 1 6 4
    L  : : : : 1 6 : 1 : : : : : : : 5 : : :
    M  : : : : 1 : : : : : 1 : : : : : : : :
    N  : : : : : : : : : : : : : : : : : : :
    P  : : : : : : : : : : : : : : : : : : :
    Q  : : : : : : : : : 6 : 3 : : 3 : 1 : 1
    R  : : : : 2 : : : : 3 : 3 : 2 7 : : 1 3
    S  : : : : : : : : 8 : 4 : : 8 : : : 1 1
    T  : 1 : : : : : : 2 1 : : : : : : : : :
    V  : 6 : : 2 : : 2 : : 1 : 6 : : : : : :
    W  : : : : : : : : : : : : : : : : : : :
    Y  : : : : : : : : : : : 3 : : : : : : :
  . 
               bits      7.3                    
                      6.6                    
                      5.9                    
                      5.1                    
      Information   4.4                     
      content   3.7                        
    (49.0 bits) 2.9                           
                      2.2                                     
                      1.5                                       
                      0.7                                       
                      0.0    
  . 
    Multilevel                 E      V      A      E      R      L      G      I      S      Q      S      Q      V      S      R      L      E      K      K   
    consensus                 D    I    G    K    E    I          R      Y    I      Q    I        R  
    sequence                          V                R                
                                                                
                                                                

  . 
  NAME       START   P-VALUE           SITES   
  gi|227813186|BAMEG_0584|    208  1.18e-19 
    LAGGEEKTQK     D      V      A      D      M      L      G      I      S      Q      S      Y      I      S      R      L      E      K      R       IIKRLRKEFN 
  gi|152976265|Bcer98_2553    208  1.18e-19 
    LAGGEEKTQK     D      V      A      D      M      L      G      I      S      Q      S      Y      I      S      R      L      E      K      R       IIKRLRKEFN 
  gi|218899058|BCG9842_B12    208  1.18e-19 
    LAGGEEKTQK     D      V      A      D      M      L      G      I      S      Q      S      Y      I      S      R      L      E      K      R       IIKRLRKEFN 
  gi|218905035|BCAH820_391    208  1.18e-19 
    LAGGEEKTQK     D      V      A      D      M      L      G      I      S      Q      S      Y      I      S      R      L      E      K      R       IIKRLRKEFN 
  gi|30263906|BA4043|NP_84    208  1.18e-19 
    LAGGEEKTQK     D      V      A      D      M      L      G      I      S      Q      S      Y      I      S      R      L      E      K      R       IIKRLRKEFN 
  gi|225865886|BCA_4008|YP    208  1.18e-19 
    LAGGEEKTQK     D      V      A      D      M      L      G      I      S      Q      S      Y      I      S      R      L      E      K      R       IIKRLRKEFN 
  gi|47529336|GBAA4043|YP_    208  1.18e-19 
    LAGGEEKTQK     D      V      A      D      M      L      G      I      S      Q      S      Y      I      S      R      L      E      K      R       IIKRLRKEFN 
  gi|52141583|BCZK3663|YP_    208  1.18e-19 
    LAGGEEKTQK     D      V      A      D      M      L      G      I      S      Q      S      Y      I      S      R      L      E      K      R       IIKRLRKEFN 
  gi|42782997|BCE_3949|NP_    208  1.18e-19 
    LAGGEEKTQK     D      V      A      D      M      L      G      I      S      Q      S      Y      I      S      R      L      E      K      R       IIKRLRKEFN 
  gi|217961326|BCAH187_A39    208  1.18e-19 
    LAGGEEKTQK     D      V      A      D      M      L      G      I      S      Q      S      Y      I      S      R      L      E      K      R       IIKRLRKEFN 
  gi|222097350|BCQ_3690|YP    208  1.18e-19 
    LAGGEEKTQK     D      V      A      D      M      L      G      I      S      Q      S      Y      I      S      R      L      E      K      R       IIKRLRKEFN 
  gi|229601447|BAA_4069|YP    208  1.18e-19 
    LAGGEEKTQK     D      V      A      D      M      L      G      I      S      Q      S      Y      I      S      R      L      E      K      R       IIKRLRKEFN 
  gi|30021993|BC3904|NP_83    208  1.18e-19 
    LAGGEEKTQK     D      V      A      D      M      L      G      I      S      Q      S      Y      I      S      R      L      E      K      R       IIKRLRKEFN 
  gi|162382776|BALH_3534|Y    208  1.18e-19 
    LAGGEEKTQK     D      V      A      D      M      L      G      I      S      Q      S      Y      I      S      R      L      E      K      R       IIKRLRKEFN 
  gi|218234749|BCB4264_A40    208  1.18e-19 
    LAGGEEKTQK     D      V      A      D      M      L      G      I      S      Q      S      Y      I      S      R      L      E      K      R       IIKRLRKEFN 
  gi|49186754|BAS3755|YP_0    208  1.18e-19 
    LAGGEEKTQK     D      V      A      D      M      L      G      I      S      Q      S      Y      I      S      R      L      E      K      R       IIKRLRKEFN 
  gi|163941644|BcerKBAB4_3    208  1.18e-19 
    LAGGEEKTQK     D      V      A      D      M      L      G      I      S      Q      S      Y      I      S      R      L      E      K      R       IIKRLRKEFN 
  gi|49478442|BT9727_3646|    208  1.18e-19 
    LAGGEEKTQK     D      V      A      D      M      L      G      I      S      Q      S      Y      I      S      R      L      E      K      R       IIKRLRKEFN 
  gi|294501014|BMQ_4270|YP    208  1.18e-19 
    LAGGEEKTQK     D      V      A      D      M      L      G      I      S      Q      S      Y      I      S      R      L      E      K      R       IIKRLRKEFN 
  gi|157692207|BPUM_1426|Y    208  1.18e-19 
    LVGGEEKTQK     D      V      A      D      M      L      G      I      S      Q      S      Y      I      S      R      L      E      K      R       IIKRLQKEFN 
  gi|296504398|BMB171_C356    208  1.18e-19 
    LAGGEEKTQK     D      V      A      D      M      L      G      I      S      Q      S      Y      I      S      R      L      E      K      R       IIKRLRKEFN 
  gi|52785509|BLi01750|YP_    208  1.18e-19 
    LVGGEEKTQK     D      V      A      D      M      L      G      I      S      Q      S      Y      I      S      R      L      E      K      R       IIKRLRKEFN 
  gi|52080135|BL02255|YP_0    208  1.18e-19 
    LVGGEEKTQK     D      V      A      D      M      L      G      I      S      Q      S      Y      I      S      R      L      E      K      R       IIKRLRKEFN 
  gi|288553151|BpOF4_00625    206  1.18e-19 
    LAGEEEKTQK     D      V      A      D      M      L      G      I      S      Q      S      Y      I      S      R      L      E      K      R       IIKRLQKEFN 
  gi|56964116|ABC2351|YP_1    206  1.18e-19 
    LAGEEEKTQK     D      V      A      D      M      L      G      I      S      Q      S      Y      I      S      R      L      E      K      R       IIKRLQKEFS 
  gi|295706363|BMD_4258|YP    208  1.18e-19 
    LAGGEEKTQK     D      V      A      D      M      L      G      I      S      Q      S      Y      I      S      R      L      E      K      R       IIKRLRKEFN 
  gi|301055395|BACI_c38600    207  1.18e-19 
    LAGGEEKTQK     D      V      A      D      M      L      G      I      S      Q      S      Y      I      S      R      L      E      K      R       IIKRLRKEFN 
  gi|16079402|BSU23450|NP_    223  1.59e-19 
    LRYYKDQTQS     E      V      A      E      R      L      G      I      S      Q      V      Q      V      S      R      L      E      K      K       ILKQIKVQMD 
  gi|157692844|BPUM_2076|Y    223  1.59e-19 
    LRYYKDQTQS     E      V      A      E      R      L      G      I      S      Q      V      Q      V      S      R      L      E      K      K       ILKQIQMQMD 
  gi|56963560|ABC1795|YP_1    223  1.59e-19 
    LRYYKDQTQS     E      V      A      E      R      L      G      I      S      Q      V      Q      V      S      R      L      E      K      K       ILEQMKETMS 
  gi|23098930|OB1475|NP_69    208  1.59e-19 
    LIGEEEKTQK     D      V      A      D      M      L      G      I      S      Q      S      Y      I      S      R      L      E      K      K       IIRRLKKEFN 
  gi|23099294|OB1839|NP_69    220  1.59e-19 
    LRYYKDKTQT     E      V      A      E      R      L      G      I      S      Q      V      Q      V      S      R      L      E      K      K       ILEDMKKNMD 
  gi|154686587|RBAM_021560    223  1.59e-19 
    LRYYKDQTQS     E      V      A      E      R      L      G      I      S      Q      V      Q      V      S      R      L      E      K      K       ILKQIKVQMD 
  gi|52080862|BL00778|YP_0    223  2.13e-19 
    LRYYKDKTQS     E      V      A      D      R      L      G      I      S      Q      V      Q      V      S      R      L      E      K      K       ILKQIKNQMD 
  gi|52786234|BLi02495|YP_    223  2.13e-19 
    LRYYKDKTQS     E      V      A      D      R      L      G      I      S      Q      V      Q      V      S      R      L      E      K      K       ILKQIKNQMD 
  gi|295696440|Btus_1834|Y    223  2.13e-19 
    LRFFRDKTQS     E      V      A      E      V      L      G      I      S      Q      V      Q      V      S      R      L      E      K      K       ILNRIRRQLL 
  gi|218905210|BCAH820_409    223  8.00e-19 
    LRYYKDQTQS     E      V      A      E      R      I      G      I      S      Q      V      Q      V      S      R      L      E      K      K       ILKQMKDRID 
  gi|217961566|BCAH187_A42    223  8.00e-19 
    LRYYKDQTQS     E      V      A      E      R      I      G      I      S      Q      V      Q      V      S      R      L      E      K      K       ILKQMKDRID 
  gi|52141420|BCZK3829|YP_    223  8.00e-19 
    LRYYKDQTQS     E      V      A      E      R      I      G      I      S      Q      V      Q      V      S      R      L      E      K      K       ILKQMKDRID 
  gi|42783188|BCE_4142|NP_    223  8.00e-19 
    LRYYKDQTQS     E      V      A      E      R      I      G      I      S      Q      V      Q      V      S      R      L      E      K      K       ILKQMKDRID 
  gi|47529588|GBAA4294|YP_    223  8.00e-19 
    LRYYKDQTQS     E      V      A      E      R      I      G      I      S      Q      V      Q      V      S      R      L      E      K      K       ILKQMKDRID 
  gi|227816852|BAMEG_4334|    223  8.00e-19 
    LRYYKDQTQS     E      V      A      E      R      I      G      I      S      Q      V      Q      V      S      R      L      E      K      K       ILKQMKDRID 
  gi|118479276|BALH_3690|Y    223  8.00e-19 
    LRYYKDQTQS     E      V      A      E      R      I      G      I      S      Q      V      Q      V      S      R      L      E      K      K       ILKQMKDRID 
  gi|225866059|BCA_4185|YP    223  8.00e-19 
    LRYYKDQTQS     E      V      A      E      R      I      G      I      S      Q      V      Q      V      S      R      L      E      K      K       ILKQMKDRID 
  gi|218899234|BCG9842_B10    223  8.00e-19 
    LRYYKDQTQS     E      V      A      E      R      I      G      I      S      Q      V      Q      V      S      R      L      E      K      K       ILKQMKDRID 
  gi|30264150|BA4294|NP_84    223  8.00e-19 
    LRYYKDQTQS     E      V      A      E      R      I      G      I      S      Q      V      Q      V      S      R      L      E      K      K       ILKQMKDRID 
  gi|49478518|BT9727_3813|    223  8.00e-19 
    LRYYKDQTQS     E      V      A      E      R      I      G      I      S      Q      V      Q      V      S      R      L      E      K      K       ILKQMKDRID 
  gi|218234550|BCB4264_A41    223  8.00e-19 
    LRYYKDQTQS     E      V      A      E      R      I      G      I      S      Q      V      Q      V      S      R      L      E      K      K       ILKQMKDRID 
  gi|222097523|BCQ_3863|YP    223  8.00e-19 
    LRYYKDQTQS     E      V      A      E      R      I      G      I      S      Q      V      Q      V      S      R      L      E      K      K       ILKQMKDRID 
  gi|49186981|BAS3983|YP_0    223  8.00e-19 
    LRYYKDQTQS     E      V      A      E      R      I      G      I      S      Q      V      Q      V      S      R      L      E      K      K       ILKQMKDRID 
  gi|30022159|BC4072|NP_83    223  8.00e-19 
    LRYYKDQTQS     E      V      A      E      R      I      G      I      S      Q      V      Q      V      S      R      L      E      K      K       ILKQMKDRID 
  gi|163941815|BcerKBAB4_3    223  8.00e-19 
    LRYYKDQTQS     E      V      A      E      R      I      G      I      S      Q      V      Q      V      S      R      L      E      K      K       ILKQMKDRID 
  gi|152976482|Bcer98_2771    223  8.00e-19 
    LRYYKDQTQS     E      V      A      E      R      I      G      I      S      Q      V      Q      V      S      R      L      E      K      K       ILKQMKDRID 
  gi|229603990|BAA_4316|YP    223  8.00e-19 
    LRYYKDQTQS     E      V      A      E      R      I      G      I      S      Q      V      Q      V      S      R      L      E      K      K       ILKQMKDRID 
  gi|301055569|BACI_c40400    223  8.00e-19 
    LRYYKDQTQS     E      V      A      E      R      I      G      I      S      Q      V      Q      V      S      R      L      E      K      K       ILKQMKDRID 
  gi|296504567|BMB171_C373    223  8.00e-19 
    LRYYKDQTQS     E      V      A      E      R      I      G      I      S      Q      V      Q      V      S      R      L      E      K      K       ILKQMKDRID 
  gi|56421070|GK2535|YP_14    200  2.49e-18 
    LGRQREKTQR     E      I      A      K      E      L      G      I      S      R      S      Y      V      S      R      I      E      K      R       ALMKMFHEFY 
  gi|154686834|RBAM_024040    200  2.49e-18 
    LDLKKEKTQR     E      I      A      K      E      L      G      I      S      R      S      Y      V      S      R      I      E      K      R       ALMKMFHEFY 
  gi|138896107|GTNG_2470|Y    200  2.49e-18 
    LGRQREKTQR     E      I      A      K      E      L      G      I      S      R      S      Y      V      S      R      I      E      K      R       ALMKMFHEFY 
  gi|261418447|GYMC61_0982    208  2.49e-18 
    LGRQREKTQR     E      I      A      K      E      L      G      I      S      R      S      Y      V      S      R      I      E      K      R       ALMKMFHEFY 
  gi|239827809|GWCH70_2471    200  2.49e-18 
    LDMQQEKTQR     E      I      A      K      E      L      G      I      S      R      S      Y      V      S      R      I      E      K      R       ALMKMFHEFY 
  gi|157693076|BPUM_2309|Y    248  2.49e-18 
    LDLKKEKTQR     E      I      A      K      E      L      G      I      S      R      S      Y      V      S      R      I      E      K      R       ALMKMFHEFS 
  gi|52081127|BL02107|YP_0    200  2.49e-18 
    LDLKKEKTQR     E      I      A      K      E      L      G      I      S      R      S      Y      V      S      R      I      E      K      R       ALMKMFHEFY 
  gi|261405539|GYMC10_1690    201  2.49e-18 
    EAGGEERTQR     E      I      A      K      E      L      G      I      S      R      S      Y      V      S      R      I      E      K      R       ALMKLYHEFY 
  gi|295695806|Btus_1170|Y    200  2.49e-18 
    LQNGEEKTQR     E      I      A      E      E      L      G      I      S      R      S      Y      V      S      R      I      E      K      R       ALMKLLHDLR 
  gi|15613848|BH1285|NP_24    200  2.49e-18 
    LDLEEERTQR     E      I      A      K      E      L      G      I      S      R      S      Y      V      S      R      I      E      K      R       ALMKLFHEFY 
  gi|297529299|GC56T3_0955    208  2.49e-18 
    LGRQREKTQR     E      I      A      K      E      L      G      I      S      R      S      Y      V      S      R      I      E      K      R       ALMKMFHEFY 
  gi|52786504|BLi02769|YP_    200  2.49e-18 
    LDLKKEKTQR     E      I      A      K      E      L      G      I      S      R      S      Y      V      S      R      I      E      K      R       ALMKMFHEFY 
  gi|163119564|BL05276|YP_    35  2.49e-18 
    LDLKKEKTQR     E      I      A      K      E      L      G      I      S      R      S      Y      V      S      R      I      E      K      R       ALMKVFHEFY 
  gi|15614101|BH1538|NP_24    223  3.08e-18 
    LRYYKDQTQS     E      V      A      Q      R      L      G      I      S      Q      V      Q      V      S      R      L      E      K      K       ILETMKANIA 
  gi|229603939|BAA_4584|YP    199  3.78e-18 
    LGLDKEKTQR     E      I      A      K      A      L      G      I      S      R      S      Y      V      S      R      I      E      K      R       ALMKMFHEFV 
  gi|49481469|BT9727_4074|    199  3.78e-18 
    LGLDKEKTQR     E      I      A      K      A      L      G      I      S      R      S      Y      V      S      R      I      E      K      R       ALMKMFHEFV 
  gi|49187232|BAS4236|YP_0    199  3.78e-18 
    LGLDKEKTQR     E      I      A      K      A      L      G      I      S      R      S      Y      V      S      R      I      E      K      R       ALMKMFHEFV 
  gi|222097784|BCQ_4125|YP    225  3.78e-18 
    LGLDKEKTQR     E      I      A      K      A      L      G      I      S      R      S      Y      V      S      R      I      E      K      R       ALMKMFHEFV 
  gi|227817116|BAMEG_4602|    199  3.78e-18 
    LGLDKEKTQR     E      I      A      K      A      L      G      I      S      R      S      Y      V      S      R      I      E      K      R       ALMKMFHEFV 
  gi|217961829|BCAH187_A44    199  3.78e-18 
    LGLDKEKTQR     E      I      A      K      A      L      G      I      S      R      S      Y      V      S      R      I      E      K      R       ALMKMFHEFV 
  gi|225866320|BCA_4450|YP    199  3.78e-18 
    LGLDKEKTQR     E      I      A      K      A      L      G      I      S      R      S      Y      V      S      R      I      E      K      R       ALMKMFHEFV 
  gi|163942095|BcerKBAB4_4    199  3.78e-18 
    LGLDKEKTQR     E      I      A      K      A      L      G      I      S      R      S      Y      V      S      R      I      E      K      R       ALMKMFHEFV 
  gi|218231149|BCB4264_A44    199  3.78e-18 
    LGLDKEKTQR     E      I      A      K      A      L      G      I      S      R      S      Y      V      S      R      I      E      K      R       ALMKMFHEFV 
  gi|218899507|BCG9842_B07    199  3.78e-18 
    LGLDKEKTQR     E      I      A      K      A      L      G      I      S      R      S      Y      V      S      R      I      E      K      R       ALMKMFHEFV 
  gi|118479504|BALH_3926|Y    233  3.78e-18 
    LGLDKEKTQR     E      I      A      K      A      L      G      I      S      R      S      Y      V      S      R      I      E      K      R       ALMKMFHEFV 
  gi|30022417|BC4336|NP_83    202  3.78e-18 
    LGLDKEKTQR     E      I      A      K      A      L      G      I      S      R      S      Y      V      S      R      I      E      K      R       ALMKMFHEFV 
  gi|152976769|Bcer98_3065    199  3.78e-18 
    LGLDKEKTQR     E      I      A      K      A      L      G      I      S      R      S      Y      V      S      R      I      E      K      R       ALMKMFHEFV 
  gi|30264411|BA4566|NP_84    199  3.78e-18 
    LGLDKEKTQR     E      I      A      K      A      L      G      I      S      R      S      Y      V      S      R      I      E      K      R       ALMKMFHEFV 
  gi|42783467|BCE_4421|NP_    227  3.78e-18 
    LGLDKEKTQR     E      I      A      K      A      L      G      I      S      R      S      Y      V      S      R      I      E      K      R       ALMKMFHEFV 
  gi|52141167|BCZK4084|YP_    199  3.78e-18 
    LGLDKEKTQR     E      I      A      K      A      L      G      I      S      R      S      Y      V      S      R      I      E      K      R       ALMKMFHEFV 
  gi|47529862|GBAA4566|YP_    199  3.78e-18 
    LGLDKEKTQR     E      I      A      K      A      L      G      I      S      R      S      Y      V      S      R      I      E      K      R       ALMKMFHEFV 
  gi|218905476|BCAH820_436    62  3.78e-18 
    LGLDKEKTQR     E      I      A      K      A      L      G      I      S      R      S      Y      V      S      R      I      E      K      R       ALMKMFHEFV 
  gi|301055830|BACI_c43070    143  3.78e-18 
    LGLDKEKTQR     E      I      A      K      A      L      G      I      S      R      S      Y      V      S      R      I      E      K      R       ALMKMFHEFV 
  gi|296504831|BMB171_C400    158  3.78e-18 
    LGLDKEKTQR     E      I      A      K      A      L      G      I      S      R      S      Y      V      S      R      I      E      K      R       ALMKMFHEFV 
  gi|212639647|Aflv_1821|Y    208  4.61e-18 
    LSGGEEKTQK     D      V      A      D      L      L      G      I      S      Q      S      Y      I      S      R      L      E      K      R       IIKRLRKEFN 
  gi|56419662|GK1127|YP_14    208  4.61e-18 
    LSGGEEKTQK     D      V      A      D      L      L      G      I      S      Q      S      Y      I      S      R      L      E      K      R       IIKRLRKEFN 
  gi|295696453|Btus_1847|Y    211  4.61e-18 
    LAGGQEMTQK     D      V      A      D      L      L      G      I      S      Q      S      Y      I      S      R      L      E      K      R       IIKRLRKEFN 
  gi|261405673|GYMC10_1824    209  4.61e-18 
    LQDGEEKTQK     D      V      A      D      L      L      G      I      S      Q      S      Y      I      S      R      L      E      K      R       IIKRLRKEFN 
  gi|15615119|BH2556|NP_24    206  4.61e-18 
    LAGGEEKTQK     D      V      A      D      L      L      G      I      S      Q      S      Y      I      S      R      L      E      K      R       IIKRLQKEFN 
  gi|138894662|GTNG_0992|Y    208  4.61e-18 
    LSGGEEKTQK     D      V      A      D      L      L      G      I      S      Q      S      Y      I      S      R      L      E      K      R       IIKRLRKEFN 
  gi|239826532|GWCH70_1030    208  4.61e-18 
    LSGGEEKTQK     D      V      A      D      L      L      G      I      S      Q      S      Y      I      S      R      L      E      K      R       IIKRLRKEFN 
  gi|297530706|GC56T3_2446    208  4.61e-18 
    LSGGEEKTQK     D      V      A      D      L      L      G      I      S      Q      S      Y      I      S      R      L      E      K      R       IIKRLRKEFN 
  gi|261419324|GYMC61_1900    208  4.61e-18 
    LSGGEEKTQK     D      V      A      D      L      L      G      I      S      Q      S      Y      I      S      R      L      E      K      R       IIKRLRKEFN 
  gi|56420843|GK2308|YP_14    221  6.78e-18 
    LRYYKDQTQS     E      V      A      S      R      L      G      I      S      Q      V      Q      V      S      R      L      E      K      K       ILQHIKDKMD 
  gi|297529524|GC56T3_1195    223  6.78e-18 
    LRYYKDQTQS     E      V      A      S      R      L      G      I      S      Q      V      Q      V      S      R      L      E      K      K       ILQHIKDKMD 
  gi|239827600|GWCH70_2250    221  6.78e-18 
    LRYYKDQTQS     E      V      A      S      R      L      G      I      S      Q      V      Q      V      S      R      L      E      K      K       ILQQIKERMD 
  gi|261417856|GYMC61_0374    223  6.78e-18 
    LRYYKDQTQS     E      V      A      S      R      L      G      I      S      Q      V      Q      V      S      R      L      E      K      K       ILQHIKDKMD 
  gi|255767354|BSU15320|NP    208  9.78e-18 
    LVGEEEKTQK     D      V      A      D      M      M      G      I      S      Q      S      Y      I      S      R      L      E      K      R       IIKRLRKEFN 
  gi|212638845|Aflv_1004|Y    218  9.78e-18 
    LRYYKDQTQS     E      V      A      A      R      L      G      I      S      Q      V      Q      V      S      R      L      E      K      K       ILQQMKARMD 
  gi|294501135|BMQ_4391|YP    223  9.78e-18 
    LRYYKDQTQS     E      V      A      A      R      L      G      I      S      Q      V      Q      V      S      R      L      E      K      K       ILKQMKLHMN 
  gi|295706482|BMD_4377|YP    223  9.78e-18 
    LRYYKDQTQS     E      V      A      A      R      L      G      I      S      Q      V      Q      V      S      R      L      E      K      K       ILKQMKLHMN 
  gi|154685948|RBAM_015150    208  9.78e-18 
    LVGEEEKTQK     D      V      A      D      M      M      G      I      S      Q      S      Y      I      S      R      L      E      K      R       IIKRLRKEFN 
  gi|138895880|GTNG_2239|Y    223  9.78e-18 
    LRYYKDQTQS     E      V      A      A      R      L      G      I      S      Q      V      Q      V      S      R      L      E      K      K       ILQHIKEKMD 
  gi|42782996|BCE_3948|NP_    260  1.65e-17 
    KRFFQGKTQM     E      V      A      E      E      I      G      I      S      Q      A      Q      V      S      R      L      E      K      S       AIKQMNKTIQ 
  gi|218233406|BCB4264_A40    230  1.65e-17 
    KRFFQGKTQM     E      V      A      E      E      I      G      I      S      Q      A      Q      V      S      R      L      E      K      S       AIKQMNKTIQ 
  gi|227813187|BAMEG_0585|    230  1.65e-17 
    KRFFQGKTQM     E      V      A      E      E      I      G      I      S      Q      A      Q      V      S      R      L      E      K      S       AIKQMNKTIQ 
  gi|163941643|BcerKBAB4_3    263  1.65e-17 
    KRFFQGKTQM     E      V      A      E      E      I      G      I      S      Q      A      Q      V      S      R      L      E      K      S       AIKQMNKTIQ 
  gi|152976264|Bcer98_2552    230  1.65e-17 
    KRFFQGKTQM     E      V      A      E      E      I      G      I      S      Q      A      Q      V      S      R      L      E      K      S       AIKQMNKTIQ 
  gi|49478441|BT9727_3645|    260  1.65e-17 
    KRFFQGKTQM     E      V      A      E      E      I      G      I      S      Q      A      Q      V      S      R      L      E      K      S       AIKQMNKTIQ 
  gi|30263905|BA4042|NP_84    230  1.65e-17 
    KRFFQGKTQM     E      V      A      E      E      I      G      I      S      Q      A      Q      V      S      R      L      E      K      S       AIKQMNKTIQ 
  gi|217961324|BCAH187_A39    230  1.65e-17 
    KRFFQGKTQM     E      V      A      E      E      I      G      I      S      Q      A      Q      V      S      R      L      E      K      S       AIKQMNKTIQ 
  gi|229602177|BAA_4068|YP    230  1.65e-17 
    KRFFQGKTQM     E      V      A      E      E      I      G      I      S      Q      A      Q      V      S      R      L      E      K      S       AIKQMNKTIQ 
  gi|30021992|BC3903|NP_83    230  1.65e-17 
    KRFFQGKTQM     E      V      A      E      E      I      G      I      S      Q      A      Q      V      S      R      L      E      K      S       AIKQMNKTIQ 
  gi|218905033|BCAH820_391    230  1.65e-17 
    KRFFQGKTQM     E      V      A      E      E      I      G      I      S      Q      A      Q      V      S      R      L      E      K      S       AIKQMNKTIQ 
  gi|225865885|BCA_4007|YP    230  1.65e-17 
    KRFFQGKTQM     E      V      A      E      E      I      G      I      S      Q      A      Q      V      S      R      L      E      K      S       AIKQMNKTIQ 
  gi|47778237|GBAA4042|YP_    230  1.65e-17 
    KRFFQGKTQM     E      V      A      E      E      I      G      I      S      Q      A      Q      V      S      R      L      E      K      S       AIKQMNKTIQ 
  gi|222097349|BCQ_3689|YP    260  1.65e-17 
    KRFFQGKTQM     E      V      A      E      E      I      G      I      S      Q      A      Q      V      S      R      L      E      K      S       AIKQMNKTIQ 
  gi|49186753|BAS3754|YP_0    260  1.65e-17 
    KRFFQGKTQM     E      V      A      E      E      I      G      I      S      Q      A      Q      V      S      R      L      E      K      S       AIKQMNKTIQ 
  gi|218899057|BCG9842_B12    230  1.65e-17 
    KRFFQGKTQM     E      V      A      E      E      I      G      I      S      Q      A      Q      V      S      R      L      E      K      S       AIKQMNKTIQ 
  gi|52141585|BCZK3662|YP_    260  1.65e-17 
    KRFFQGKTQM     E      V      A      E      E      I      G      I      S      Q      A      Q      V      S      R      L      E      K      S       AIKQMNKTIQ 
  gi|118479123|BALH_3533|Y    260  1.65e-17 
    KRFFQGKTQM     E      V      A      E      E      I      G      I      S      Q      A      Q      V      S      R      L      E      K      S       AIKQMNKTIQ 
  gi|301055394|BACI_c38590    260  1.65e-17 
    KRFFQGKTQM     E      V      A      E      E      I      G      I      S      Q      A      Q      V      S      R      L      E      K      S       AIKQMNKTIQ 
  gi|295696452|Btus_1846|Y    230  1.65e-17 
    MRFFEGKTQM     E      V      A      E      E      I      G      I      S      Q      A      Q      V      S      R      L      E      K      S       AIQHMQKFIK 
  gi|296504397|BMB171_C356    230  1.65e-17 
    KRFFQGKTQM     E      V      A      E      E      I      G      I      S      Q      A      Q      V      S      R      L      E      K      S       AIKQMNKTIQ 
  gi|169827019|Bsph_1443|Y    172  1.65e-17 
    LNGKVEMTQK     E      V      A      D      H      L      G      I      S      Q      S      Y      I      S      R      L      E      K      K       IIQDLRENLN 
  gi|261405674|GYMC10_1825    231  1.94e-17 
    MRFYEGKTQM     E      V      A      D      E      I      G      I      S      Q      A      Q      V      S      R      L      E      K      S       AIKQMQKHVK 
  gi|212639646|Aflv_1820|Y    230  2.68e-17 
    KRFFQGKTQM     E      V      A      E      E      I      G      I      S      Q      A      Q      V      S      R      L      E      K      A       AIKQMNKNIQ 
  gi|56419663|GK1128|YP_14    230  2.68e-17 
    KRFFQGKTQM     E      V      A      E      E      I      G      I      S      Q      A      Q      V      S      R      L      E      K      A       AIRQMNKNIQ 
  gi|16078597|BSU15330|NP_    231  2.68e-17 
    KRFFQGKTQM     E      V      A      E      E      I      G      I      S      Q      A      Q      V      S      R      L      E      K      A       AIKQMNKNIH 
  gi|297530705|GC56T3_2445    230  2.68e-17 
    KRFFQGKTQM     E      V      A      E      E      I      G      I      S      Q      A      Q      V      S      R      L      E      K      A       AIRQMNKNIQ 
  gi|157692208|BPUM_1427|Y    231  2.68e-17 
    KRFFQGKTQM     E      V      A      E      E      I      G      I      S      Q      A      Q      V      S      R      L      E      K      A       AIKQMNKNIF 
  gi|239826533|GWCH70_1031    230  2.68e-17 
    KRFFQGKTQM     E      V      A      E      E      I      G      I      S      Q      A      Q      V      S      R      L      E      K      A       AIRQMNKNIQ 
  gi|295706362|BMD_4257|YP    230  2.68e-17 
    KRFFQGKTQM     E      V      A      E      E      I      G      I      S      Q      A      Q      V      S      R      L      E      K      A       AIKQMNKNIQ 
  gi|138894663|GTNG_0993|Y    235  2.68e-17 
    KRFFQGKTQM     E      V      A      E      E      I      G      I      S      Q      A      Q      V      S      R      L      E      K      A       AIRQMNKNIQ 
  gi|154685949|RBAM_015160    231  2.68e-17 
    KRFFQGKTQM     E      V      A      E      E      I      G      I      S      Q      A      Q      V      S      R      L      E      K      A       AIKQMNKNIH 
  gi|294501013|BMQ_4269|YP    230  2.68e-17 
    KRFFQGKTQM     E      V      A      E      E      I      G      I      S      Q      A      Q      V      S      R      L      E      K      A       AIKQMNKNIQ 
  gi|15615117|BH2554|NP_24    232  2.68e-17 
    MRFYQGKTQM     E      V      A      E      E      I      G      I      S      Q      A      Q      V      S      R      L      E      K      A       AIQQMSKHAQ 
  gi|261419325|GYMC61_1901    230  2.68e-17 
    KRFFQGKTQM     E      V      A      E      E      I      G      I      S      Q      A      Q      V      S      R      L      E      K      A       AIRQMNKNIQ 
  gi|288555973|BpOF4_14835    225  3.13e-17 
    LRYYKDQTQS     E      V      A      T      R      L      G      I      S      Q      V      Q      V      S      R      L      E      K      K       ILEQMKEVMG 
  gi|288553150|BpOF4_00620    232  3.13e-17 
    MRFFQGKTQM     E      V      A      D      E      I      G      I      S      Q      A      Q      V      S      R      L      E      K      A       AIQQMNKHAQ 
  gi|23098931|OB1476|NP_69    231  3.13e-17 
    KRFFQGKTQM     E      V      A      D      E      I      G      I      S      Q      A      Q      V      S      R      L      E      K      A       AISQMNKQMF 
  gi|52785510|BLi01751|YP_    231  3.13e-17 
    KRFFQGKTQM     E      V      A      D      E      I      G      I      S      Q      A      Q      V      S      R      L      E      K      A       AIKQMNKNIH 
  gi|261405990|GYMC10_2143    222  3.13e-17 
    LRYYRDQTQS     E      V      A      T      R      L      G      I      S      Q      V      Q      V      S      R      L      E      K      K       ILQSIRNEIA 
  gi|52080136|BL02256|YP_0    231  3.13e-17 
    KRFFQGKTQM     E      V      A      D      E      I      G      I      S      Q      A      Q      V      S      R      L      E      K      A       AIKQMNKNIH 
  gi|288555607|BpOF4_12995    200  4.92e-17 
    LDMEDELTQR     E      I      A      K      K      L      G      I      S      R      S      Y      V      S      R      I      E      K      R       ALMKLFHEFY 
  gi|52784380|BLi00560|YP_    227  6.54e-17 
    LTYIQNKSQK     E      T      G      E      M      L      G      I      S      Q      M      H      V      S      R      L      Q      R      K       AVKRLREALA 
  gi|52079008|BL02208|YP_0    226  6.54e-17 
    LTYIQNKSQK     E      T      G      E      M      L      G      I      S      Q      M      H      V      S      R      L      Q      R      K       AVKRLREALA 
  gi|294501329|BMQ_4591|YP    200  1.45e-16 
    LDLQDEKTQR     E      I      A      K      E      L      N      I      S      R      S      Y      V      S      R      I      E      K      R       ALMKMFHEFY 
  gi|56963382|ABC1617|YP_1    200  1.45e-16 
    LNMEEERTQR     E      I      A      K      E      L      N      I      S      R      S      Y      V      S      R      I      E      K      R       ALMKLFHEFY 
  gi|295706676|BMD_4577|YP    200  1.45e-16 
    LDLQDEKTQR     E      I      A      K      E      L      N      I      S      R      S      Y      V      S      R      I      E      K      R       ALMKMFHEFY 
  gi|169827282|Bsph_1714|Y    153  1.86e-16 
    LRYFSDCTQT     E      I      A      N      R      L      G      I      S      Q      V      Q      V      S      R      L      E      K      K       ILAQLKSWMA 
  gi|56964115|ABC2350|YP_1    232  3.36e-16 
    MRFFQGKTQM     E      V      A      E      E      I      G      I      S      Q      A      Q      V      S      R      L      E      K      G       AIQQMNKHIE 
  gi|15613092|BH0529|NP_24    226  3.36e-16 
    CTYFEHLSQK     E      T      G      H      R      L      G      I      S      Q      M      H      V      S      R      L      Q      R      R       ALKKLRDSIR 
  gi|23098085|OB0630|NP_69    227  3.76e-16 
    CTYFKNMSQK     E      T      G      E      L      L      G      I      S      Q      M      H      V      S      R      L      Q      R      R       SLRKLREAIQ 
  gi|229917800|EAT1b_2078|    227  3.76e-16 
    CAYYKNMSQK     E      T      G      E      L      L      G      I      S      Q      M      H      V      S      R      L      Q      R      R       ALEKLREAIK 
  gi|172058807|Exig_2804|Y    227  3.76e-16 
    CAYYRNMSQK     E      T      G      E      L      L      G      I      S      Q      M      H      V      S      R      L      Q      R      R       ALQKLREAIK 
  gi|157691239|BPUM_0446|Y    226  4.69e-16 
    LTYLQNKSQK     E      T      G      D      L      L      G      I      S      Q      M      H      V      S      R      L      Q      R      K       AVKKLRDALS 
  gi|169829306|Bsph_3856|Y    193  5.82e-16 
    LNQQDALTQK     E      I      A      A      R      L      N      I      S      R      S      Y      V      S      R      I      E      K      R       ALIKLYQLYK 
  gi|218232670|BCB4264_A10    226  6.47e-16 
    YIFGENLNQK     D      T      G      E      R      L      G      I      S      Q      M      H      V      S      R      I      K      R      Q       AISKLKQAAF 
  gi|218896055|BCG9842_B42    226  6.47e-16 
    YIFGENLNQK     D      T      G      E      R      L      G      I      S      Q      M      H      V      S      R      I      K      R      Q       AISKLKQAAF 
  gi|225862940|BCA_1029|YP    226  6.47e-16 
    YIFEENLNQK     D      T      G      E      R      L      G      I      S      Q      M      H      V      S      R      I      K      R      Q       AISKLKQAAF 
  gi|227816152|BAMEG_3579|    226  6.47e-16 
    YIFEENLNQK     D      T      G      E      R      L      G      I      S      Q      M      H      V      S      R      I      K      R      Q       AISKLKQAAF 
  gi|229604478|BAA_1086|YP    226  6.47e-16 
    YIFEENLNQK     D      T      G      E      R      L      G      I      S      Q      M      H      V      S      R      I      K      R      Q       AISKLKQAAF 
  gi|30261116|BA0992|NP_84    226  6.47e-16 
    YIFEENLNQK     D      T      G      E      R      L      G      I      S      Q      M      H      V      S      R      I      K      R      Q       AISKLKQAAF 
  gi|47526271|GBAA0992|YP_    226  6.47e-16 
    YIFEENLNQK     D      T      G      E      R      L      G      I      S      Q      M      H      V      S      R      I      K      R      Q       AISKLKQAAF 
  gi|118476614|BALH_0889|Y    227  6.47e-16 
    YIFEENLNQK     D      T      G      E      R      L      G      I      S      Q      M      H      V      S      R      I      K      R      Q       AISKLKQAAF 
  gi|52144331|BCZK0896|YP_    227  6.47e-16 
    YIFEENLNQK     D      T      G      E      R      L      G      I      S      Q      M      H      V      S      R      I      K      R      Q       AISKLKQAAF 
  gi|218902186|BCAH820_106    226  6.47e-16 
    YIFEENLNQK     D      T      G      E      R      L      G      I      S      Q      M      H      V      S      R      I      K      R      Q       AISKLKQAAF 
  gi|30019159|BC1004|NP_83    227  6.47e-16 
    YIFGENLNQK     D      T      G      E      R      L      G      I      S      Q      M      H      V      S      R      I      K      R      Q       AISKLKQAAF 
  gi|222094730|BCQ_1068|YP    226  6.47e-16 
    YIFGENLNQK     D      T      G      E      R      L      G      I      S      Q      M      H      V      S      R      I      K      R      Q       AISKLKQAAF 
  gi|217958581|BCAH187_A11    226  6.47e-16 
    YIFGENLNQK     D      T      G      E      R      L      G      I      S      Q      M      H      V      S      R      I      K      R      Q       AISKLKQAAF 
  gi|49480184|BT9727_0913|    227  6.47e-16 
    YIFEENLNQK     D      T      G      E      R      L      G      I      S      Q      M      H      V      S      R      I      K      R      Q       AISKLKQAAF 
  gi|49183950|BAS0928|YP_0    227  6.47e-16 
    YIFEENLNQK     D      T      G      E      R      L      G      I      S      Q      M      H      V      S      R      I      K      R      Q       AISKLKQAAF 
  gi|42780162|BCE_1086|NP_    226  6.47e-16 
    YIFEENLNQK     D      T      G      E      R      L      G      I      S      Q      M      H      V      S      R      I      K      R      Q       AISKLKQAAF 
  gi|163938900|BcerKBAB4_0    226  6.47e-16 
    YIFGENLNQK     D      T      G      E      R      L      G      I      S      Q      M      H      V      S      R      I      K      R      Q       AISKLKQAAF 
  gi|301052635|BACI_c10250    227  6.47e-16 
    YIFEENLNQK     D      T      G      E      R      L      G      I      S      Q      M      H      V      S      R      I      K      R      Q       AISKLKQAAF 
  gi|296501716|BMB171_C087    226  6.47e-16 
    YIFGENLNQK     D      T      G      E      R      L      G      I      S      Q      M      H      V      S      R      I      K      R      Q       AISKLKQAAF 
  gi|255767137|BSU04730|NP    226  7.18e-16 
    LTYIQNKSQK     E      T      G      D      I      L      G      I      S      Q      M      H      V      S      R      L      Q      R      K       AVKKLREALI 
  gi|154684976|RBAM_005070    226  7.18e-16 
    LTFVQNKSQK     E      T      G      D      I      L      G      I      S      Q      M      H      V      S      R      L      Q      R      K       AVKKLREALI 
  gi|169827020|Bsph_1444|Y    229  7.18e-16 
    KRFYYGETQT     E      I      A      K      E      L      G      I      S      Q      A      Q      I      S      R      L      E      K      N       AIETMQKDYK 
  gi|56962590|ABC0816|YP_1    226  9.75e-16 
    CTYYENLSQK     E      T      G      E      K      L      G      I      S      Q      M      H      V      S      R      L      Q      R      R       ALQKLRDSIR 
  gi|288554854|BpOF4_09205    226  9.75e-16 
    LTYYDNLSQK     E      T      G      E      K      L      G      I      S      Q      M      H      V      S      R      L      Q      R      R       ALQKLRDSIK 
  gi|23099454|OB1999|NP_69    200  9.75e-16 
    LNDREEMTQR     E      I      A      K      K      L      N      I      S      R      S      Y      V      S      R      I      E      K      R       ALMKVFHEYY 
  gi|297582861|Bsel_0539|Y    233  1.45e-15 
    LTYFENMSQK     E      T      G      E      Q      L      G      I      S      Q      M      H      V      S      R      L      Q      R      R       ALQKLKDSIR 
  gi|218232261|BCB4264_A44    338  4.40e-15 
    LDDGRTRTLE     E      V      G      K      V      F      G      V      T      R      E      R      I      R      Q      I      E      A      K       ALRKLRHPSR 
  gi|52141207|BCZK4042|YP_    336  4.40e-15 
    LDDGRTRTLE     E      V      G      K      V      F      G      V      T      R      E      R      I      R      Q      I      E      A      K       ALRKLRHPSR 
  gi|42783418|BCE_4372|NP_    338  4.40e-15 
    LDDGRTRTLE     E      V      G      K      V      F      G      V      T      R      E      R      I      R      Q      I      E      A      K       ALRKLRHPSR 
  gi|30264362|BA4515|NP_84    336  4.40e-15 
    LDDGRTRTLE     E      V      G      K      V      F      G      V      T      R      E      R      I      R      Q      I      E      A      K       ALRKLRHPSR 
  gi|222097738|BCQ_4079|YP    338  4.40e-15 
    LDDGRTRTLE     E      V      G      K      V      F      G      V      T      R      E      R      I      R      Q      I      E      A      K       ALRKLRHPSR 
  gi|218899458|BCG9842_B08    338  4.40e-15 
    LDDGRTRTLE     E      V      G      K      V      F      G      V      T      R      E      R      I      R      Q      I      E      A      K       ALRKLRHPSR 
  gi|218905429|BCAH820_431    336  4.40e-15 
    LDDGRTRTLE     E      V      G      K      V      F      G      V      T      R      E      R      I      R      Q      I      E      A      K       ALRKLRHPSR 
  gi|229601154|BAA_4536|YP    336  4.40e-15 
    LDDGRTRTLE     E      V      G      K      V      F      G      V      T      R      E      R      I      R      Q      I      E      A      K       ALRKLRHPSR 
  gi|30022370|BC4289|NP_83    338  4.40e-15 
    LDDGRTRTLE     E      V      G      K      V      F      G      V      T      R      E      R      I      R      Q      I      E      A      K       ALRKLRHPSR 
  gi|152976724|Bcer98_3019    336  4.40e-15 
    LDDGRTRTLE     E      V      G      K      V      F      G      V      T      R      E      R      I      R      Q      I      E      A      K       ALRKLRHPSR 
  gi|163942050|BcerKBAB4_4    338  4.40e-15 
    LDDGRTRTLE     E      V      G      K      V      F      G      V      T      R      E      R      I      R      Q      I      E      A      K       ALRKLRHPSR 
  gi|56421017|GK2482|YP_14    338  4.40e-15 
    LDDGRTRTLE     E      V      G      K      V      F      G      V      T      R      E      R      I      R      Q      I      E      A      K       ALRKLRHPSR 
  gi|255767573|BSU25200|NP    334  4.40e-15 
    LDDGRTRTLE     E      V      G      K      V      F      G      V      T      R      E      R      I      R      Q      I      E      A      K       ALRKLRHPSR 
  gi|212638699|Aflv_0856|Y    335  4.40e-15 
    LDDGRTRTLE     E      V      G      K      V      F      G      V      T      R      E      R      I      R      Q      I      E      A      K       ALRKLRHPSR 
  gi|118479464|BALH_3885|Y    336  4.40e-15 
    LDDGRTRTLE     E      V      G      K      V      F      G      V      T      R      E      R      I      R      Q      I      E      A      K       ALRKLRHPSR 
  gi|217961783|BCAH187_A44    338  4.40e-15 
    LDDGRTRTLE     E      V      G      K      V      F      G      V      T      R      E      R      I      R      Q      I      E      A      K       ALRKLRHPSR 
  gi|47529811|GBAA4515|YP_    336  4.40e-15 
    LDDGRTRTLE     E      V      G      K      V      F      G      V      T      R      E      R      I      R      Q      I      E      A      K       ALRKLRHPSR 
  gi|49187190|BAS4194|YP_0    336  4.40e-15 
    LDDGRTRTLE     E      V      G      K      V      F      G      V      T      R      E      R      I      R      Q      I      E      A      K       ALRKLRHPSR 
  gi|225866273|BCA_4403|YP    336  4.40e-15 
    LDDGRTRTLE     E      V      G      K      V      F      G      V      T      R      E      R      I      R      Q      I      E      A      K       ALRKLRHPSR 
  gi|227817068|BAMEG_4554|    336  4.40e-15 
    LDDGRTRTLE     E      V      G      K      V      F      G      V      T      R      E      R      I      R      Q      I      E      A      K       ALRKLRHPSR 
  gi|49481307|BT9727_4032|    336  4.40e-15 
    LDDGRTRTLE     E      V      G      K      V      F      G      V      T      R      E      R      I      R      Q      I      E      A      K       ALRKLRHPSR 
  gi|297584635|Bsel_2346|Y    334  4.40e-15 
    LDDGRTRTLE     E      V      G      K      V      F      G      V      T      R      E      R      I      R      Q      I      E      A      K       ALRKLRHPSR 
  gi|295706627|BMD_4528|YP    340  4.40e-15 
    LDDGRTRTLE     E      V      G      K      V      F      G      V      T      R      E      R      I      R      Q      I      E      A      K       ALRKLRHPSR 
  gi|297529352|GC56T3_1008    338  4.40e-15 
    LDDGRTRTLE     E      V      G      K      V      F      G      V      T      R      E      R      I      R      Q      I      E      A      K       ALRKLRHPSR 
  gi|261417657|GYMC61_0157    338  4.40e-15 
    LDDGRTRTLE     E      V      G      K      V      F      G      V      T      R      E      R      I      R      Q      I      E      A      K       ALRKLRHPSR 
  gi|56963455|ABC1690|YP_1    336  4.40e-15 
    LDDGRTRTLE     E      V      G      K      V      F      G      V      T      R      E      R      I      R      Q      I      E      A      K       ALRKLRHPSR 
  gi|239827752|GWCH70_2414    337  4.40e-15 
    LDDGRTRTLE     E      V      G      K      V      F      G      V      T      R      E      R      I      R      Q      I      E      A      K       ALRKLRHPSR 
  gi|229916320|EAT1b_0589|    327  4.40e-15 
    LDDGRTRTLE     E      V      G      K      V      F      G      V      T      R      E      R      I      R      Q      I      E      A      K       ALRKLRHPSR 
  gi|157693020|BPUM_2253|Y    336  4.40e-15 
    LDDGRTRTLE     E      V      G      K      V      F      G      V      T      R      E      R      I      R      Q      I      E      A      K       ALRKLRHPSR 
  gi|154686781|RBAM_023510    336  4.40e-15 
    LDDGRTRTLE     E      V      G      K      V      F      G      V      T      R      E      R      I      R      Q      I      E      A      K       ALRKLRHPSR 
  gi|288555754|BpOF4_13730    335  4.40e-15 
    LDDGRTRTLE     E      V      G      K      V      F      G      V      T      R      E      R      I      R      Q      I      E      A      K       ALRKLRHPSR 
  gi|169829158|Bsph_3702|Y    338  4.40e-15 
    LDDGRTRTLE     E      V      G      K      V      F      G      V      T      R      E      R      I      R      Q      I      E      A      K       ALRKLRHPSR 
  gi|301055785|BACI_c42620    338  4.40e-15 
    LDDGRTRTLE     E      V      G      K      V      F      G      V      T      R      E      R      I      R      Q      I      E      A      K       ALRKLRHPSR 
  gi|163119552|BL03682|YP_    336  4.40e-15 
    LDDGRTRTLE     E      V      G      K      V      F      G      V      T      R      E      R      I      R      Q      I      E      A      K       ALRKLRHPSR 
  gi|172056869|Exig_0832|Y    324  4.40e-15 
    LDDGRTRTLE     E      V      G      K      V      F      G      V      T      R      E      R      I      R      Q      I      E      A      K       ALRKLRHPSR 
  gi|52786447|BLi02712|YP_    336  4.40e-15 
    LDDGRTRTLE     E      V      G      K      V      F      G      V      T      R      E      R      I      R      Q      I      E      A      K       ALRKLRHPSR 
  gi|261405623|GYMC10_1774    340  4.40e-15 
    LDDGRTRTLE     E      V      G      K      V      F      G      V      T      R      E      R      I      R      Q      I      E      A      K       ALRKLRHPSR 
  gi|23099399|OB1944|NP_69    343  4.40e-15 
    LDDGRTRTLE     E      V      G      K      V      F      G      V      T      R      E      R      I      R      Q      I      E      A      K       ALRKLRHPSR 
  gi|294501280|BMQ_4542|YP    340  4.40e-15 
    LDDGRTRTLE     E      V      G      K      V      F      G      V      T      R      E      R      I      R      Q      I      E      A      K       ALRKLRHPSR 
  gi|138896056|GTNG_2419|Y    338  4.40e-15 
    LDDGRTRTLE     E      V      G      K      V      F      G      V      T      R      E      R      I      R      Q      I      E      A      K       ALRKLRHPSR 
  gi|295695448|Btus_0783|Y    342  4.40e-15 
    LDDGRTRTLE     E      V      G      K      V      F      G      V      T      R      E      R      I      R      Q      I      E      A      K       ALRKLRHPSR 
  gi|15613939|BH1376|NP_24    335  4.40e-15 
    LDDGRTRTLE     E      V      G      K      V      F      G      V      T      R      E      R      I      R      Q      I      E      A      K       ALRKLRHPSR 
  gi|296504786|BMB171_C395    338  4.40e-15 
    LDDGRTRTLE     E      V      G      K      V      F      G      V      T      R      E      R      I      R      Q      I      E      A      K       ALRKLRHPSR 
  gi|172057862|Exig_1853|Y    219  6.27e-15 
    LFYFEELTLT     E      I      G      E      V      L      G      L      S      T      S      R      I      S      Q      I      H      S      K       ALRKLKQALG 
  gi|169829726|Bsph_4295|Y    226  9.66e-15 
    LTYLEQLSQK     E      A      G      E      Q      L      G      I      S      Q      M      H      V      S      R      I      Q      R      K       AIKKLQEAIL 
  gi|294497070|BMQ_0235|YP    226  1.15e-14 
    YTFIENLSQK     D      A      G      E      K      L      G      I      S      Q      M      H      V      S      R      L      Q      R      R       ALDKLRTAAR 
  gi|295702435|BMD_0229|YP    226  1.15e-14 
    YTFIENLSQK     D      A      G      E      K      L      G      I      S      Q      M      H      V      S      R      L      Q      R      R       ALDKLRTAAR 
  gi|261407775|GYMC10_3981    279  1.25e-14 
    LEDGRTRTLE     E      V      G      Q      V      F      G      V      T      R      E      R      I      R      Q      I      E      A      K       ALRKLRHPSR 
  gi|239826640|GWCH70_1138    223  3.12e-14 
    LFYKEELTFT     E      I      G      E      I      L      G      L      S      T      S      R      I      S      Q      L      H      S      K       AIFKLRKILE 
  gi|294500913|BMQ_4167|YP    222  4.31e-14 
    LFYKEELTFT     E      I      A      Q      L      L      G      L      S      T      S      R      I      S      Q      I      H      S      K       ALFKMRTSLS 
  gi|295706259|BMD_4154|YP    222  4.31e-14 
    LFYKEELTFT     E      I      A      Q      L      L      G      L      S      T      S      R      I      S      Q      I      H      S      K       ALFKMRTSLS 
  gi|295696140|Btus_1522|Y    206  5.06e-14 
    LHYIEGLSFR     E      V      A      A      V      L      G      V      T      P      A      R      V      S      Q      L      H      S      K       ALLRLRSRLA 
  gi|261409324|GYMC10_5549    224  8.77e-14 
    LAFQEGQSQR     A      I      A      Q      R      L      G      V      S      Q      M      S      V      S      R      I      Q      K      R       ATEKLKQIMS 
  gi|56421959|GK3424|YP_14    224  1.02e-13 
    AVFRRGETQR     S      L      A      E      R      L      G      V      S      Q      M      T      I      S      R      I      Q      K      R       AIAKLKRQLA 
  gi|138897001|GTNG_3372|Y    226  1.02e-13 
    AVFRRGETQR     S      L      A      K      R      L      G      V      S      Q      M      T      I      S      R      I      Q      K      R       AIEKLKRQLA 
  gi|261420836|GYMC61_3488    224  1.02e-13 
    AIFRRGETQR     S      L      A      E      R      L      G      V      S      Q      M      T      I      S      R      I      Q      K      R       AIAKLKRQLA 
  gi|239827399|GWCH70_2031    797  1.02e-13 
    FMTGEEMTLQ     E      I      G      E      L      F      G      V      S      R      E      R      I      R      Q      I      E      N      K       ALKHLKKCPA 
  gi|16078710|BSU16470|NP_    222  1.29e-13 
    LFYKEELTLT     E      I      G      Q      V      L      N      L      S      T      S      R      I      S      Q      I      H      S      K       ALFKLKNLLE 
  gi|154686064|RBAM_016310    222  1.29e-13 
    LFYKEELTLT     E      I      G      Q      V      L      N      L      S      T      S      R      I      S      Q      I      H      S      K       ALFKLKHLLE 
  gi|297531625|GC56T3_3410    224  1.29e-13 
    AIFRRGETQR     A      L      A      E      R      L      G      V      S      Q      M      T      I      S      R      I      Q      K      R       AIAKLKRELA 
  gi|52785627|BLi01868|YP_    222  1.29e-13 
    LFYKEELTLT     E      I      G      Q      V      L      N      L      S      T      S      R      I      S      Q      I      H      S      K       ALLKLKHLLD 
  gi|52080250|BL01246|YP_0    222  1.29e-13 
    LFYKEELTLT     E      I      G      Q      V      L      N      L      S      T      S      R      I      S      Q      I      H      S      K       ALLKLKHLLD 
  gi|157692327|BPUM_1546|Y    222  1.39e-13 
    LFYKEELTLT     E      I      G      H      V      L      N      L      S      T      S      R      I      S      Q      I      H      S      K       ALFKLKHLLD 
  gi|212639538|Aflv_1712|Y    212  1.61e-13 
    LFYKEELTFT     E      I      G      H      I      M      G      L      S      T      S      R      I      S      Q      I      H      A      K       ALFKMRKLIE 
  gi|23099037|OB1582|NP_69    225  1.61e-13 
    LFYNEELTFT     E      I      G      Q      V      V      G      L      T      T      S      R      I      S      Q      I      H      K      R       SIFKLKKTLE 
  gi|169827149|Bsph_1579|Y    237  2.71e-13 
    LFYTEELTLT     E      I      G      E      M      L      E      L      S      T      S      R      I      S      Q      I      H      S      K       ALLKLRKLLS 
  gi|229918653|EAT1b_2940|    219  3.13e-13 
    LFYFDELTLT     E      I      G      E      V      L      E      L      S      T      S      R      I      S      Q      I      H      S      K       ALKTLKHALG 
  gi|297584063|Bsel_1770|Y    224  3.36e-13 
    LFYYEELTLT     E      I      G      E      A      L      S      L      S      T      S      R      I      S      Q      I      H      S      K       AVYKLQQQLH 
  gi|56419781|GK1246|YP_14    220  3.88e-13 
    LFYKEELTFT     E      I      G      S      L      L      H      L      S      T      S      R      I      S      Q      I      H      A      K       ALWKLRRFFE 
  gi|261419446|GYMC61_2030    220  3.88e-13 
    LFYKEELTFT     E      I      G      S      L      L      H      L      S      T      S      R      I      S      Q      I      H      A      K       ALWKLRRFFE 
  gi|297530579|GC56T3_2307    220  3.88e-13 
    LFYKEELTFT     E      I      G      S      L      L      H      L      S      T      S      R      I      S      Q      I      H      A      K       ALWKLRRFFE 
  gi|288553039|BpOF4_00065    228  4.80e-13 
    LFYFDELTLT     E      I      G      H      I      L      N      L      S      T      S      R      I      S      Q      I      H      S      K       ALFRMNQLIN 
  gi|56964009|ABC2244|YP_1    220  7.31e-13 
    LSYYEELSLT     E      I      G      R      I      L      N      L      S      T      S      R      I      S      Q      I      H      A      R       AIAKLRKGLQ 
  gi|138894766|GTNG_1100|Y    222  1.55e-12 
    LFYKEELTFT     E      I      G      S      I      L      Q      L      S      T      S      R      I      S      Q      I      H      A      K       ALWKLRRFFA 
  gi|218848158|BCG9842_003    253  1.55e-12 
    FDDGIELTLE     D      T      A      R      I      L      G      V      T      R      E      R      V      Q      Q      I      E      K      K       AIEKMKQRLS 
  gi|261407955|GYMC10_4162    224  2.16e-12 
    LLYYEDLSLS     E      I      A      E      V      M      S      L      S      P      S      R      I      S      Q      L      H      S      K       AILRLRGALD 
  gi|15614994|BH2431|NP_24    224  2.81e-12 
    LFYFEELTLT     E      I      G      Q      I      L      K      L      S      T      S      R      I      S      Q      I      H      S      K       ALFRLQQALK 
  gi|157691589|BPUM_0807|Y    154  8.34e-12 
    HIYVEGQTMK     E      I      A      D      R      L      G      E      T      R      Q      N      I      S      N      I      H      K      K       ALNKLKEETG 
  gi|296506459|BMB171_P007    274  1.75e-11 
    LLDGKVYLHK     E      I      A      K      A      L      M      I      T      T      E      R      V      S      Q      I      K      D      E       AIKKLKKCQH 
  gi|67078088|pE33L466_021    274  2.51e-11 
    LLDGKVYLHK     E      I      A      K      A      L      M      I      T      T      E      R      V      S      Q      I      K      E      E       AIKKLKKCDY 
  gi|49477012|BT9727_0859|    166  1.77e-10 
    LIYIVNYNNK     E      V      A      K      L      L      G      E      S      E      Q      T      V      S      Y      N      H      K      K       AIKKLRNSMN 
 
    
      	Motif 3 block diagrams  
    
 
  Name Lowest p-value    Motifs
 
     gi|227813186|BAMEG_0584|
  1.2e-19
    
    
   3
    
   
  
 
     gi|152976265|Bcer98_2553
  1.2e-19
    
    
   3
    
   
  
 
     gi|218899058|BCG9842_B12
  1.2e-19
    
    
   3
    
   
  
 
     gi|218905035|BCAH820_391
  1.2e-19
    
    
   3
    
   
  
 
     gi|30263906|BA4043|NP_84
  1.2e-19
    
    
   3
    
   
  
 
     gi|225865886|BCA_4008|YP
  1.2e-19
    
    
   3
    
   
  
 
     gi|47529336|GBAA4043|YP_
  1.2e-19
    
    
   3
    
   
  
 
     gi|52141583|BCZK3663|YP_
  1.2e-19
    
    
   3
    
   
  
 
     gi|42782997|BCE_3949|NP_
  1.2e-19
    
    
   3
    
   
  
 
     gi|217961326|BCAH187_A39
  1.2e-19
    
    
   3
    
   
  
 
     gi|222097350|BCQ_3690|YP
  1.2e-19
    
    
   3
    
   
  
 
     gi|229601447|BAA_4069|YP
  1.2e-19
    
    
   3
    
   
  
 
     gi|30021993|BC3904|NP_83
  1.2e-19
    
    
   3
    
   
  
 
     gi|162382776|BALH_3534|Y
  1.2e-19
    
    
   3
    
   
  
 
     gi|218234749|BCB4264_A40
  1.2e-19
    
    
   3
    
   
  
 
     gi|49186754|BAS3755|YP_0
  1.2e-19
    
    
   3
    
   
  
 
     gi|163941644|BcerKBAB4_3
  1.2e-19
    
    
   3
    
   
  
 
     gi|49478442|BT9727_3646|
  1.2e-19
    
    
   3
    
   
  
 
     gi|294501014|BMQ_4270|YP
  1.2e-19
    
    
   3
    
   
  
 
     gi|157692207|BPUM_1426|Y
  1.2e-19
    
    
   3
    
   
  
 
     gi|296504398|BMB171_C356
  1.2e-19
    
    
   3
    
   
  
 
     gi|52785509|BLi01750|YP_
  1.2e-19
    
    
   3
    
   
  
 
     gi|52080135|BL02255|YP_0
  1.2e-19
    
    
   3
    
   
  
 
     gi|288553151|BpOF4_00625
  1.2e-19
    
    
   3
    
   
  
 
     gi|56964116|ABC2351|YP_1
  1.2e-19
    
    
   3
    
   
  
 
     gi|295706363|BMD_4258|YP
  1.2e-19
    
    
   3
    
   
  
 
     gi|301055395|BACI_c38600
  1.2e-19
    
    
   3
    
   
  
 
     gi|16079402|BSU23450|NP_
  1.6e-19
    
    
   3
    
   
  
 
     gi|157692844|BPUM_2076|Y
  1.6e-19
    
    
   3
    
   
  
 
     gi|56963560|ABC1795|YP_1
  1.6e-19
    
    
   3
    
   
  
 
     gi|23098930|OB1475|NP_69
  1.6e-19
    
    
   3
    
   
  
 
     gi|23099294|OB1839|NP_69
  1.6e-19
    
    
   3
    
   
  
 
     gi|154686587|RBAM_021560
  1.6e-19
    
    
   3
    
   
  
 
     gi|52080862|BL00778|YP_0
  2.1e-19
    
    
   3
    
   
  
 
     gi|52786234|BLi02495|YP_
  2.1e-19
    
    
   3
    
   
  
 
     gi|295696440|Btus_1834|Y
  2.1e-19
    
    
   3
    
   
  
 
     gi|218905210|BCAH820_409
  8e-19
    
    
   3
    
   
  
 
     gi|217961566|BCAH187_A42
  8e-19
    
    
   3
    
   
  
 
     gi|52141420|BCZK3829|YP_
  8e-19
    
    
   3
    
   
  
 
     gi|42783188|BCE_4142|NP_
  8e-19
    
    
   3
    
   
  
 
     gi|47529588|GBAA4294|YP_
  8e-19
    
    
   3
    
   
  
 
     gi|227816852|BAMEG_4334|
  8e-19
    
    
   3
    
   
  
 
     gi|118479276|BALH_3690|Y
  8e-19
    
    
   3
    
   
  
 
     gi|225866059|BCA_4185|YP
  8e-19
    
    
   3
    
   
  
 
     gi|218899234|BCG9842_B10
  8e-19
    
    
   3
    
   
  
 
     gi|30264150|BA4294|NP_84
  8e-19
    
    
   3
    
   
  
 
     gi|49478518|BT9727_3813|
  8e-19
    
    
   3
    
   
  
 
     gi|218234550|BCB4264_A41
  8e-19
    
    
   3
    
   
  
 
     gi|222097523|BCQ_3863|YP
  8e-19
    
    
   3
    
   
  
 
     gi|49186981|BAS3983|YP_0
  8e-19
    
    
   3
    
   
  
 
     gi|30022159|BC4072|NP_83
  8e-19
    
    
   3
    
   
  
 
     gi|163941815|BcerKBAB4_3
  8e-19
    
    
   3
    
   
  
 
     gi|152976482|Bcer98_2771
  8e-19
    
    
   3
    
   
  
 
     gi|229603990|BAA_4316|YP
  8e-19
    
    
   3
    
   
  
 
     gi|301055569|BACI_c40400
  8e-19
    
    
   3
    
   
  
 
     gi|296504567|BMB171_C373
  8e-19
    
    
   3
    
   
  
 
     gi|56421070|GK2535|YP_14
  2.5e-18
    
    
   3
    
   
  
 
     gi|154686834|RBAM_024040
  2.5e-18
    
    
   3
    
   
  
 
     gi|138896107|GTNG_2470|Y
  2.5e-18
    
    
   3
    
   
  
 
     gi|261418447|GYMC61_0982
  2.5e-18
    
    
   3
    
   
  
 
     gi|239827809|GWCH70_2471
  2.5e-18
    
    
   3
    
   
  
 
     gi|157693076|BPUM_2309|Y
  2.5e-18
    
    
   3
    
   
  
 
     gi|52081127|BL02107|YP_0
  2.5e-18
    
    
   3
    
   
  
 
     gi|261405539|GYMC10_1690
  2.5e-18
    
    
   3
    
   
  
 
     gi|295695806|Btus_1170|Y
  2.5e-18
    
    
   3
    
   
  
 
     gi|15613848|BH1285|NP_24
  2.5e-18
    
    
   3
    
   
  
 
     gi|297529299|GC56T3_0955
  2.5e-18
    
    
   3
    
   
  
 
     gi|52786504|BLi02769|YP_
  2.5e-18
    
    
   3
    
   
  
 
     gi|163119564|BL05276|YP_
  2.5e-18
    
    
   3
    
   
  
 
     gi|15614101|BH1538|NP_24
  3.1e-18
    
    
   3
    
   
  
 
     gi|229603939|BAA_4584|YP
  3.8e-18
    
    
   3
    
   
  
 
     gi|49481469|BT9727_4074|
  3.8e-18
    
    
   3
    
   
  
 
     gi|49187232|BAS4236|YP_0
  3.8e-18
    
    
   3
    
   
  
 
     gi|222097784|BCQ_4125|YP
  3.8e-18
    
    
   3
    
   
  
 
     gi|227817116|BAMEG_4602|
  3.8e-18
    
    
   3
    
   
  
 
     gi|217961829|BCAH187_A44
  3.8e-18
    
    
   3
    
   
  
 
     gi|225866320|BCA_4450|YP
  3.8e-18
    
    
   3
    
   
  
 
     gi|163942095|BcerKBAB4_4
  3.8e-18
    
    
   3
    
   
  
 
     gi|218231149|BCB4264_A44
  3.8e-18
    
    
   3
    
   
  
 
     gi|218899507|BCG9842_B07
  3.8e-18
    
    
   3
    
   
  
 
     gi|118479504|BALH_3926|Y
  3.8e-18
    
    
   3
    
   
  
 
     gi|30022417|BC4336|NP_83
  3.8e-18
    
    
   3
    
   
  
 
     gi|152976769|Bcer98_3065
  3.8e-18
    
    
   3
    
   
  
 
     gi|30264411|BA4566|NP_84
  3.8e-18
    
    
   3
    
   
  
 
     gi|42783467|BCE_4421|NP_
  3.8e-18
    
    
   3
    
   
  
 
     gi|52141167|BCZK4084|YP_
  3.8e-18
    
    
   3
    
   
  
 
     gi|47529862|GBAA4566|YP_
  3.8e-18
    
    
   3
    
   
  
 
     gi|218905476|BCAH820_436
  3.8e-18
    
    
   3
    
   
  
 
     gi|301055830|BACI_c43070
  3.8e-18
    
    
   3
    
   
  
 
     gi|296504831|BMB171_C400
  3.8e-18
    
    
   3
    
   
  
 
     gi|212639647|Aflv_1821|Y
  4.6e-18
    
    
   3
    
   
  
 
     gi|56419662|GK1127|YP_14
  4.6e-18
    
    
   3
    
   
  
 
     gi|295696453|Btus_1847|Y
  4.6e-18
    
    
   3
    
   
  
 
     gi|261405673|GYMC10_1824
  4.6e-18
    
    
   3
    
   
  
 
     gi|15615119|BH2556|NP_24
  4.6e-18
    
    
   3
    
   
  
 
     gi|138894662|GTNG_0992|Y
  4.6e-18
    
    
   3
    
   
  
 
     gi|239826532|GWCH70_1030
  4.6e-18
    
    
   3
    
   
  
 
     gi|297530706|GC56T3_2446
  4.6e-18
    
    
   3
    
   
  
 
     gi|261419324|GYMC61_1900
  4.6e-18
    
    
   3
    
   
  
 
     gi|56420843|GK2308|YP_14
  6.8e-18
    
    
   3
    
   
  
 
     gi|297529524|GC56T3_1195
  6.8e-18
    
    
   3
    
   
  
 
     gi|239827600|GWCH70_2250
  6.8e-18
    
    
   3
    
   
  
 
     gi|261417856|GYMC61_0374
  6.8e-18
    
    
   3
    
   
  
 
     gi|255767354|BSU15320|NP
  9.8e-18
    
    
   3
    
   
  
 
     gi|212638845|Aflv_1004|Y
  9.8e-18
    
    
   3
    
   
  
 
     gi|294501135|BMQ_4391|YP
  9.8e-18
    
    
   3
    
   
  
 
     gi|295706482|BMD_4377|YP
  9.8e-18
    
    
   3
    
   
  
 
     gi|154685948|RBAM_015150
  9.8e-18
    
    
   3
    
   
  
 
     gi|138895880|GTNG_2239|Y
  9.8e-18
    
    
   3
    
   
  
 
     gi|42782996|BCE_3948|NP_
  1.6e-17
    
    
   3
    
   
  
 
     gi|218233406|BCB4264_A40
  1.6e-17
    
    
   3
    
   
  
 
     gi|227813187|BAMEG_0585|
  1.6e-17
    
    
   3
    
   
  
 
     gi|163941643|BcerKBAB4_3
  1.6e-17
    
    
   3
    
   
  
 
     gi|152976264|Bcer98_2552
  1.6e-17
    
    
   3
    
   
  
 
     gi|49478441|BT9727_3645|
  1.6e-17
    
    
   3
    
   
  
 
     gi|30263905|BA4042|NP_84
  1.6e-17
    
    
   3
    
   
  
 
     gi|217961324|BCAH187_A39
  1.6e-17
    
    
   3
    
   
  
 
     gi|229602177|BAA_4068|YP
  1.6e-17
    
    
   3
    
   
  
 
     gi|30021992|BC3903|NP_83
  1.6e-17
    
    
   3
    
   
  
 
     gi|218905033|BCAH820_391
  1.6e-17
    
    
   3
    
   
  
 
     gi|225865885|BCA_4007|YP
  1.6e-17
    
    
   3
    
   
  
 
     gi|47778237|GBAA4042|YP_
  1.6e-17
    
    
   3
    
   
  
 
     gi|222097349|BCQ_3689|YP
  1.6e-17
    
    
   3
    
   
  
 
     gi|49186753|BAS3754|YP_0
  1.6e-17
    
    
   3
    
   
  
 
     gi|218899057|BCG9842_B12
  1.6e-17
    
    
   3
    
   
  
 
     gi|52141585|BCZK3662|YP_
  1.6e-17
    
    
   3
    
   
  
 
     gi|118479123|BALH_3533|Y
  1.6e-17
    
    
   3
    
   
  
 
     gi|301055394|BACI_c38590
  1.6e-17
    
    
   3
    
   
  
 
     gi|295696452|Btus_1846|Y
  1.6e-17
    
    
   3
    
   
  
 
     gi|296504397|BMB171_C356
  1.6e-17
    
    
   3
    
   
  
 
     gi|169827019|Bsph_1443|Y
  1.6e-17
    
    
   3
    
   
  
 
     gi|261405674|GYMC10_1825
  1.9e-17
    
    
   3
    
   
  
 
     gi|212639646|Aflv_1820|Y
  2.7e-17
    
    
   3
    
   
  
 
     gi|56419663|GK1128|YP_14
  2.7e-17
    
    
   3
    
   
  
 
     gi|16078597|BSU15330|NP_
  2.7e-17
    
    
   3
    
   
  
 
     gi|297530705|GC56T3_2445
  2.7e-17
    
    
   3
    
   
  
 
     gi|157692208|BPUM_1427|Y
  2.7e-17
    
    
   3
    
   
  
 
     gi|239826533|GWCH70_1031
  2.7e-17
    
    
   3
    
   
  
 
     gi|295706362|BMD_4257|YP
  2.7e-17
    
    
   3
    
   
  
 
     gi|138894663|GTNG_0993|Y
  2.7e-17
    
    
   3
    
   
  
 
     gi|154685949|RBAM_015160
  2.7e-17
    
    
   3
    
   
  
 
     gi|294501013|BMQ_4269|YP
  2.7e-17
    
    
   3
    
   
  
 
     gi|15615117|BH2554|NP_24
  2.7e-17
    
    
   3
    
   
  
 
     gi|261419325|GYMC61_1901
  2.7e-17
    
    
   3
    
   
  
 
     gi|288555973|BpOF4_14835
  3.1e-17
    
    
   3
    
   
  
 
     gi|288553150|BpOF4_00620
  3.1e-17
    
    
   3
    
   
  
 
     gi|23098931|OB1476|NP_69
  3.1e-17
    
    
   3
    
   
  
 
     gi|52785510|BLi01751|YP_
  3.1e-17
    
    
   3
    
   
  
 
     gi|261405990|GYMC10_2143
  3.1e-17
    
    
   3
    
   
  
 
     gi|52080136|BL02256|YP_0
  3.1e-17
    
    
   3
    
   
  
 
     gi|288555607|BpOF4_12995
  4.9e-17
    
    
   3
    
   
  
 
     gi|52784380|BLi00560|YP_
  6.5e-17
    
    
   3
    
   
  
 
     gi|52079008|BL02208|YP_0
  6.5e-17
    
    
   3
    
   
  
 
     gi|294501329|BMQ_4591|YP
  1.5e-16
    
    
   3
    
   
  
 
     gi|56963382|ABC1617|YP_1
  1.5e-16
    
    
   3
    
   
  
 
     gi|295706676|BMD_4577|YP
  1.5e-16
    
    
   3
    
   
  
 
     gi|169827282|Bsph_1714|Y
  1.9e-16
    
    
   3
    
   
  
 
     gi|56964115|ABC2350|YP_1
  3.4e-16
    
    
   3
    
   
  
 
     gi|15613092|BH0529|NP_24
  3.4e-16
    
    
   3
    
   
  
 
     gi|23098085|OB0630|NP_69
  3.8e-16
    
    
   3
    
   
  
 
     gi|229917800|EAT1b_2078|
  3.8e-16
    
    
   3
    
   
  
 
     gi|172058807|Exig_2804|Y
  3.8e-16
    
    
   3
    
   
  
 
     gi|157691239|BPUM_0446|Y
  4.7e-16
    
    
   3
    
   
  
 
     gi|169829306|Bsph_3856|Y
  5.8e-16
    
    
   3
    
   
  
 
     gi|218232670|BCB4264_A10
  6.5e-16
    
    
   3
    
   
  
 
     gi|218896055|BCG9842_B42
  6.5e-16
    
    
   3
    
   
  
 
     gi|225862940|BCA_1029|YP
  6.5e-16
    
    
   3
    
   
  
 
     gi|227816152|BAMEG_3579|
  6.5e-16
    
    
   3
    
   
  
 
     gi|229604478|BAA_1086|YP
  6.5e-16
    
    
   3
    
   
  
 
     gi|30261116|BA0992|NP_84
  6.5e-16
    
    
   3
    
   
  
 
     gi|47526271|GBAA0992|YP_
  6.5e-16
    
    
   3
    
   
  
 
     gi|118476614|BALH_0889|Y
  6.5e-16
    
    
   3
    
   
  
 
     gi|52144331|BCZK0896|YP_
  6.5e-16
    
    
   3
    
   
  
 
     gi|218902186|BCAH820_106
  6.5e-16
    
    
   3
    
   
  
 
     gi|30019159|BC1004|NP_83
  6.5e-16
    
    
   3
    
   
  
 
     gi|222094730|BCQ_1068|YP
  6.5e-16
    
    
   3
    
   
  
 
     gi|217958581|BCAH187_A11
  6.5e-16
    
    
   3
    
   
  
 
     gi|49480184|BT9727_0913|
  6.5e-16
    
    
   3
    
   
  
 
     gi|49183950|BAS0928|YP_0
  6.5e-16
    
    
   3
    
   
  
 
     gi|42780162|BCE_1086|NP_
  6.5e-16
    
    
   3
    
   
  
 
     gi|163938900|BcerKBAB4_0
  6.5e-16
    
    
   3
    
   
  
 
     gi|301052635|BACI_c10250
  6.5e-16
    
    
   3
    
   
  
 
     gi|296501716|BMB171_C087
  6.5e-16
    
    
   3
    
   
  
 
     gi|255767137|BSU04730|NP
  7.2e-16
    
    
   3
    
   
  
 
     gi|154684976|RBAM_005070
  7.2e-16
    
    
   3
    
   
  
 
     gi|169827020|Bsph_1444|Y
  7.2e-16
    
    
   3
    
   
  
 
     gi|56962590|ABC0816|YP_1
  9.8e-16
    
    
   3
    
   
  
 
     gi|288554854|BpOF4_09205
  9.8e-16
    
    
   3
    
   
  
 
     gi|23099454|OB1999|NP_69
  9.8e-16
    
    
   3
    
   
  
 
     gi|297582861|Bsel_0539|Y
  1.4e-15
    
    
   3
    
   
  
 
     gi|218232261|BCB4264_A44
  4.4e-15
    
    
   3
    
   
  
 
     gi|52141207|BCZK4042|YP_
  4.4e-15
    
    
   3
    
   
  
 
     gi|42783418|BCE_4372|NP_
  4.4e-15
    
    
   3
    
   
  
 
     gi|30264362|BA4515|NP_84
  4.4e-15
    
    
   3
    
   
  
 
     gi|222097738|BCQ_4079|YP
  4.4e-15
    
    
   3
    
   
  
 
     gi|218899458|BCG9842_B08
  4.4e-15
    
    
   3
    
   
  
 
     gi|218905429|BCAH820_431
  4.4e-15
    
    
   3
    
   
  
 
     gi|229601154|BAA_4536|YP
  4.4e-15
    
    
   3
    
   
  
 
     gi|30022370|BC4289|NP_83
  4.4e-15
    
    
   3
    
   
  
 
     gi|152976724|Bcer98_3019
  4.4e-15
    
    
   3
    
   
  
 
     gi|163942050|BcerKBAB4_4
  4.4e-15
    
    
   3
    
   
  
 
     gi|56421017|GK2482|YP_14
  4.4e-15
    
    
   3
    
   
  
 
     gi|255767573|BSU25200|NP
  4.4e-15
    
    
   3
    
   
  
 
     gi|212638699|Aflv_0856|Y
  4.4e-15
    
    
   3
    
   
  
 
     gi|118479464|BALH_3885|Y
  4.4e-15
    
    
   3
    
   
  
 
     gi|217961783|BCAH187_A44
  4.4e-15
    
    
   3
    
   
  
 
     gi|47529811|GBAA4515|YP_
  4.4e-15
    
    
   3
    
   
  
 
     gi|49187190|BAS4194|YP_0
  4.4e-15
    
    
   3
    
   
  
 
     gi|225866273|BCA_4403|YP
  4.4e-15
    
    
   3
    
   
  
 
     gi|227817068|BAMEG_4554|
  4.4e-15
    
    
   3
    
   
  
 
     gi|49481307|BT9727_4032|
  4.4e-15
    
    
   3
    
   
  
 
     gi|297584635|Bsel_2346|Y
  4.4e-15
    
    
   3
    
   
  
 
     gi|295706627|BMD_4528|YP
  4.4e-15
    
    
   3
    
   
  
 
     gi|297529352|GC56T3_1008
  4.4e-15
    
    
   3
    
   
  
 
     gi|261417657|GYMC61_0157
  4.4e-15
    
    
   3
    
   
  
 
     gi|56963455|ABC1690|YP_1
  4.4e-15
    
    
   3
    
   
  
 
     gi|239827752|GWCH70_2414
  4.4e-15
    
    
   3
    
   
  
 
     gi|229916320|EAT1b_0589|
  4.4e-15
    
    
   3
    
   
  
 
     gi|157693020|BPUM_2253|Y
  4.4e-15
    
    
   3
    
   
  
 
     gi|154686781|RBAM_023510
  4.4e-15
    
    
   3
    
   
  
 
     gi|288555754|BpOF4_13730
  4.4e-15
    
    
   3
    
   
  
 
     gi|169829158|Bsph_3702|Y
  4.4e-15
    
    
   3
    
   
  
 
     gi|301055785|BACI_c42620
  4.4e-15
    
    
   3
    
   
  
 
     gi|163119552|BL03682|YP_
  4.4e-15
    
    
   3
    
   
  
 
     gi|172056869|Exig_0832|Y
  4.4e-15
    
    
   3
    
   
  
 
     gi|52786447|BLi02712|YP_
  4.4e-15
    
    
   3
    
   
  
 
     gi|261405623|GYMC10_1774
  4.4e-15
    
    
   3
    
   
  
 
     gi|23099399|OB1944|NP_69
  4.4e-15
    
    
   3
    
   
  
 
     gi|294501280|BMQ_4542|YP
  4.4e-15
    
    
   3
    
   
  
 
     gi|138896056|GTNG_2419|Y
  4.4e-15
    
    
   3
    
   
  
 
     gi|295695448|Btus_0783|Y
  4.4e-15
    
    
   3
    
   
  
 
     gi|15613939|BH1376|NP_24
  4.4e-15
    
    
   3
    
   
  
 
     gi|296504786|BMB171_C395
  4.4e-15
    
    
   3
    
   
  
 
     gi|172057862|Exig_1853|Y
  6.3e-15
    
    
   3
    
   
  
 
     gi|169829726|Bsph_4295|Y
  9.7e-15
    
    
   3
    
   
  
 
     gi|294497070|BMQ_0235|YP
  1.1e-14
    
    
   3
    
   
  
 
     gi|295702435|BMD_0229|YP
  1.1e-14
    
    
   3
    
   
  
 
     gi|261407775|GYMC10_3981
  1.2e-14
    
    
   3
    
   
  
 
     gi|239826640|GWCH70_1138
  3.1e-14
    
    
   3
    
   
  
 
     gi|294500913|BMQ_4167|YP
  4.3e-14
    
    
   3
    
   
  
 
     gi|295706259|BMD_4154|YP
  4.3e-14
    
    
   3
    
   
  
 
     gi|295696140|Btus_1522|Y
  5.1e-14
    
    
   3
    
   
  
 
     gi|261409324|GYMC10_5549
  8.8e-14
    
    
   3
    
   
  
 
     gi|56421959|GK3424|YP_14
  1e-13
    
    
   3
    
   
  
 
     gi|138897001|GTNG_3372|Y
  1e-13
    
    
   3
    
   
  
 
     gi|261420836|GYMC61_3488
  1e-13
    
    
   3
    
   
  
 
     gi|239827399|GWCH70_2031
  1e-13
    
    
   3
    
   
  
 
     gi|16078710|BSU16470|NP_
  1.3e-13
    
    
   3
    
   
  
 
     gi|154686064|RBAM_016310
  1.3e-13
    
    
   3
    
   
  
 
     gi|297531625|GC56T3_3410
  1.3e-13
    
    
   3
    
   
  
 
     gi|52785627|BLi01868|YP_
  1.3e-13
    
    
   3
    
   
  
 
     gi|52080250|BL01246|YP_0
  1.3e-13
    
    
   3
    
   
  
 
     gi|157692327|BPUM_1546|Y
  1.4e-13
    
    
   3
    
   
  
 
     gi|212639538|Aflv_1712|Y
  1.6e-13
    
    
   3
    
   
  
 
     gi|23099037|OB1582|NP_69
  1.6e-13
    
    
   3
    
   
  
 
     gi|169827149|Bsph_1579|Y
  2.7e-13
    
    
   3
    
   
  
 
     gi|229918653|EAT1b_2940|
  3.1e-13
    
    
   3
    
   
  
 
     gi|297584063|Bsel_1770|Y
  3.4e-13
    
    
   3
    
   
  
 
     gi|56419781|GK1246|YP_14
  3.9e-13
    
    
   3
    
   
  
 
     gi|261419446|GYMC61_2030
  3.9e-13
    
    
   3
    
   
  
 
     gi|297530579|GC56T3_2307
  3.9e-13
    
    
   3
    
   
  
 
     gi|288553039|BpOF4_00065
  4.8e-13
    
    
   3
    
   
  
 
     gi|56964009|ABC2244|YP_1
  7.3e-13
    
    
   3
    
   
  
 
     gi|138894766|GTNG_1100|Y
  1.5e-12
    
    
   3
    
   
  
 
     gi|218848158|BCG9842_003
  1.5e-12
    
    
   3
    
   
  
 
     gi|261407955|GYMC10_4162
  2.2e-12
    
    
   3
    
   
  
 
     gi|15614994|BH2431|NP_24
  2.8e-12
    
    
   3
    
   
  
 
     gi|157691589|BPUM_0807|Y
  8.3e-12
    
    
   3
    
   
  
 
     gi|296506459|BMB171_P007
  1.7e-11
    
    
   3
    
   
  
 
     gi|67078088|pE33L466_021
  2.5e-11
    
    
   3
    
   
  
 
     gi|49477012|BT9727_0859|
  1.8e-10
    
    
   3
    
   
  
  SCALE
     
     | 
     | 
     | 
     | 
     | 
     | 
     | 
     | 
     | 
     | 
     | 
     | 
     | 
     | 
     | 
     | 
     | 
     | 
     | 
     | 
     | 
     | 
     | 
     | 
     | 
     | 
     | 
     | 
     | 
     | 
     | 
     | 
     | 
    1 
     25 
     50 
     75 
     100 
     125 
     150 
     175 
     200 
     225 
     250 
     275 
     300 
     325 
     350 
     375 
     400 
     425 
     450 
     475 
     500 
     525 
     550 
     575 
     600 
     625 
     650 
     675 
     700 
     725 
     750 
     775 
     800 
   
 

    
      	Motif 3 in BLOCKS format  
    
 
 
 
 
  
 to  BLOCKS multiple alignment processor.  
    
      	Motif 3 position-specific scoring matrix  
    
 
 

    
      	Motif 3 position-specific probability matrix  
    
 
 

    
      	Motif 3 regular expression  
    
[ED][VI][AG][EK][REV][LI]GIS[QR]S[QYR][VI]S[RQ][LI]EK[KR]

 


Time 15539.35 secs.

 

    
   
     P  
     N        MOTIF  4       width = 11         sites = 255        llr = 5312        E-value = 1.4e-1163 
    
 
    Simplified  A  : : : : 1 : : 5 : : :
    pos.-specific  C  : : : : : : : : : : :
    probability  D  : : : : : : : : : : :
    matrix  E  : : : : : : : : : : :
    F  : : : 1 1 : : : : : 7
    G  : : : : : 1 : : : : :
    H  : : : : : 1 : : : : :
    I  1 : : : : : 6 2 : : :
    K  1 : : : : : : : 4 3 :
    L  6 : a : 2 : : : : : 1
    M  1 : : : : : : : : : :
    N  : 1 : : : : : : : : :
    P  : : : : : : : : : : :
    Q  : : : : : : : : 4 : :
    R  : 9 : : : : : : 2 7 :
    S  : : : : : 6 : : : : :
    T  : : : : : : : : : : :
    V  : : : 9 3 : 3 3 : : :
    W  : : : : 2 : : : : : :
    Y  : : : : 1 2 : : 1 : 3
  . 
               bits      7.3            
                      6.6            
                      5.9            
                      5.1            
      Information   4.4            
      content   3.7              
    (30.1 bits) 2.9                   
                      2.2                      
                      1.5                       
                      0.7                       
                      0.0    
  . 
    Multilevel                 L      R      L      V      V      S      I      A      K      R      F   
    consensus                             V    V    Q    K    Y  
    sequence                                      
                                                
                                                

  . 
  NAME       START   P-VALUE           SITES   
  gi|218905210|BCAH820_409    42  2.40e-13 
    QARDTIVQSN     M      R      L      V      W      S      V      V      Q      R      F       LNRGYEPDDL 
  gi|217961566|BCAH187_A42    42  2.40e-13 
    QARDTIVQSN     M      R      L      V      W      S      V      V      Q      R      F       LNRGYEPDDL 
  gi|52141420|BCZK3829|YP_    42  2.40e-13 
    QARDTIVQSN     M      R      L      V      W      S      V      V      Q      R      F       LNRGYEPDDL 
  gi|212638845|Aflv_1004|Y    38  2.40e-13 
    QARDEIIEKN     M      R      L      V      W      S      V      V      Q      R      F       LNRGYEPEDL 
  gi|42783188|BCE_4142|NP_    42  2.40e-13 
    QARDTIVQSN     M      R      L      V      W      S      V      V      Q      R      F       LNRGYEPDDL 
  gi|47529588|GBAA4294|YP_    42  2.40e-13 
    QARDTIVQSN     M      R      L      V      W      S      V      V      Q      R      F       LNRGYEPDDL 
  gi|227816852|BAMEG_4334|    42  2.40e-13 
    QARDTIVQSN     M      R      L      V      W      S      V      V      Q      R      F       LNRGYEPDDL 
  gi|118479276|BALH_3690|Y    42  2.40e-13 
    QARDTIVQSN     M      R      L      V      W      S      V      V      Q      R      F       LNRGYEPDDL 
  gi|225866059|BCA_4185|YP    42  2.40e-13 
    QARDTIVQSN     M      R      L      V      W      S      V      V      Q      R      F       LNRGYEPDDL 
  gi|16079402|BSU23450|NP_    42  2.40e-13 
    QARDLLIEKN     M      R      L      V      W      S      V      V      Q      R      F       LNRGYEPDDL 
  gi|218899234|BCG9842_B10    42  2.40e-13 
    QARDTIVQSN     M      R      L      V      W      S      V      V      Q      R      F       LNRGYEPDDL 
  gi|30264150|BA4294|NP_84    42  2.40e-13 
    QARDTIVQSN     M      R      L      V      W      S      V      V      Q      R      F       LNRGYEPDDL 
  gi|49478518|BT9727_3813|    42  2.40e-13 
    QARDTIVQSN     M      R      L      V      W      S      V      V      Q      R      F       LNRGYEPDDL 
  gi|218234550|BCB4264_A41    42  2.40e-13 
    QARDTIVQSN     M      R      L      V      W      S      V      V      Q      R      F       LNRGYEPDDL 
  gi|222097523|BCQ_3863|YP    42  2.40e-13 
    QARDTIVQSN     M      R      L      V      W      S      V      V      Q      R      F       LNRGYEPDDL 
  gi|49186981|BAS3983|YP_0    42  2.40e-13 
    QARDTIVQSN     M      R      L      V      W      S      V      V      Q      R      F       LNRGYEPDDL 
  gi|30022159|BC4072|NP_83    42  2.40e-13 
    QARDTIVQSN     M      R      L      V      W      S      V      V      Q      R      F       LNRGYEPDDL 
  gi|163941815|BcerKBAB4_3    42  2.40e-13 
    QARDTIVQSN     M      R      L      V      W      S      V      V      Q      R      F       LNRGYEPDDL 
  gi|152976482|Bcer98_2771    42  2.40e-13 
    GARDTIVQSN     M      R      L      V      W      S      V      V      Q      R      F       LNRGYEPDDL 
  gi|229603990|BAA_4316|YP    42  2.40e-13 
    QARDTIVQSN     M      R      L      V      W      S      V      V      Q      R      F       LNRGYEPDDL 
  gi|56420843|GK2308|YP_14    40  2.40e-13 
    EARDEIIEKN     M      R      L      V      W      S      V      V      Q      R      F       LNRGYEADDL 
  gi|157692844|BPUM_2076|Y    42  2.40e-13 
    QARDLLVEKN     M      R      L      V      W      S      V      V      Q      R      F       LNRGYEPDDL 
  gi|52080862|BL00778|YP_0    42  2.40e-13 
    KARDLLIEKN     M      R      L      V      W      S      V      V      Q      R      F       LNRGYEPDDL 
  gi|301055569|BACI_c40400    42  2.40e-13 
    QARDTIVQSN     M      R      L      V      W      S      V      V      Q      R      F       LNRGYEPDDL 
  gi|296504567|BMB171_C373    42  2.40e-13 
    QARDTIVQSN     M      R      L      V      W      S      V      V      Q      R      F       LNRGYEPDDL 
  gi|294501135|BMQ_4391|YP    42  2.40e-13 
    IARDTIVQKN     M      R      L      V      W      S      V      V      Q      R      F       INRGYEPDDL 
  gi|295706482|BMD_4377|YP    42  2.40e-13 
    IARDTIVQKN     M      R      L      V      W      S      V      V      Q      R      F       INRGYEPDDL 
  gi|52786234|BLi02495|YP_    42  2.40e-13 
    KARDLLIEKN     M      R      L      V      W      S      V      V      Q      R      F       LNRGYEPDDL 
  gi|297529524|GC56T3_1195    42  2.40e-13 
    EARDEIIEKN     M      R      L      V      W      S      V      V      Q      R      F       LNRGYEADDL 
  gi|239827600|GWCH70_2250    40  2.40e-13 
    EARDEIIEKN     M      R      L      V      W      S      V      V      Q      R      F       LNRGYEPDDL 
  gi|138895880|GTNG_2239|Y    42  2.40e-13 
    EARDEIIEKN     M      R      L      V      W      S      V      V      Q      R      F       LNRGYEADDL 
  gi|261417856|GYMC61_0374    42  2.40e-13 
    EARDEIIEKN     M      R      L      V      W      S      V      V      Q      R      F       LNRGYEADDL 
  gi|154686587|RBAM_021560    42  2.40e-13 
    QARDLLIEKN     M      R      L      V      W      S      V      V      Q      R      F       LNRGYEPDDL 
  gi|218232261|BCB4264_A44    147  6.17e-12 
    EAKRRLAEAN     L      R      L      V      V      S      I      A      K      R      Y       VGRGMLFLDL 
  gi|52141207|BCZK4042|YP_    145  6.17e-12 
    EAKRRLAEAN     L      R      L      V      V      S      I      A      K      R      Y       VGRGMLFLDL 
  gi|42783418|BCE_4372|NP_    147  6.17e-12 
    EAKRRLAEAN     L      R      L      V      V      S      I      A      K      R      Y       VGRGMLFLDL 
  gi|30264362|BA4515|NP_84    145  6.17e-12 
    EAKRRLAEAN     L      R      L      V      V      S      I      A      K      R      Y       VGRGMLFLDL 
  gi|222097738|BCQ_4079|YP    147  6.17e-12 
    EAKRRLAEAN     L      R      L      V      V      S      I      A      K      R      Y       VGRGMLFLDL 
  gi|218899458|BCG9842_B08    147  6.17e-12 
    EAKRRLAEAN     L      R      L      V      V      S      I      A      K      R      Y       VGRGMLFLDL 
  gi|218905429|BCAH820_431    145  6.17e-12 
    EAKRRLAEAN     L      R      L      V      V      S      I      A      K      R      Y       VGRGMLFLDL 
  gi|229601154|BAA_4536|YP    145  6.17e-12 
    EAKRRLAEAN     L      R      L      V      V      S      I      A      K      R      Y       VGRGMLFLDL 
  gi|30022370|BC4289|NP_83    147  6.17e-12 
    EAKRRLAEAN     L      R      L      V      V      S      I      A      K      R      Y       VGRGMLFLDL 
  gi|152976724|Bcer98_3019    145  6.17e-12 
    EAKRRLAEAN     L      R      L      V      V      S      I      A      K      R      Y       VGRGMLFLDL 
  gi|163942050|BcerKBAB4_4    147  6.17e-12 
    EAKRRLAEAN     L      R      L      V      V      S      I      A      K      R      Y       VGRGMLFLDL 
  gi|56421017|GK2482|YP_14    147  6.17e-12 
    EAKRRLTEAN     L      R      L      V      V      S      I      A      K      R      Y       VGRGMLFLDL 
  gi|255767573|BSU25200|NP    143  6.17e-12 
    ESKRRLAEAN     L      R      L      V      V      S      I      A      K      R      Y       VGRGMLFLDL 
  gi|212638699|Aflv_0856|Y    144  6.17e-12 
    EAKRRLAEAN     L      R      L      V      V      S      I      A      K      R      Y       VGRGMLFLDL 
  gi|118479464|BALH_3885|Y    145  6.17e-12 
    EAKRRLAEAN     L      R      L      V      V      S      I      A      K      R      Y       VGRGMLFLDL 
  gi|217961783|BCAH187_A44    147  6.17e-12 
    EAKRRLAEAN     L      R      L      V      V      S      I      A      K      R      Y       VGRGMLFLDL 
  gi|47529811|GBAA4515|YP_    145  6.17e-12 
    EAKRRLAEAN     L      R      L      V      V      S      I      A      K      R      Y       VGRGMLFLDL 
  gi|49187190|BAS4194|YP_0    145  6.17e-12 
    EAKRRLAEAN     L      R      L      V      V      S      I      A      K      R      Y       VGRGMLFLDL 
  gi|225866273|BCA_4403|YP    145  6.17e-12 
    EAKRRLAEAN     L      R      L      V      V      S      I      A      K      R      Y       VGRGMLFLDL 
  gi|227817068|BAMEG_4554|    145  6.17e-12 
    EAKRRLAEAN     L      R      L      V      V      S      I      A      K      R      Y       VGRGMLFLDL 
  gi|49481307|BT9727_4032|    145  6.17e-12 
    EAKRRLAEAN     L      R      L      V      V      S      I      A      K      R      Y       VGRGMLFLDL 
  gi|297584635|Bsel_2346|Y    143  6.17e-12 
    EAKRRLAEAN     L      R      L      V      V      S      I      A      K      R      Y       VGRGMLFLDL 
  gi|295706627|BMD_4528|YP    149  6.17e-12 
    EAKRRLAEAN     L      R      L      V      V      S      I      A      K      R      Y       VGRGMLFLDL 
  gi|297529352|GC56T3_1008    147  6.17e-12 
    EAKRRLTEAN     L      R      L      V      V      S      I      A      K      R      Y       VGRGMLFLDL 
  gi|261417657|GYMC61_0157    147  6.17e-12 
    EAKRRLTEAN     L      R      L      V      V      S      I      A      K      R      Y       VGRGMLFLDL 
  gi|56963455|ABC1690|YP_1    145  6.17e-12 
    EAKKRLAEAN     L      R      L      V      V      S      I      A      K      R      Y       VGRGMLFLDL 
  gi|239827752|GWCH70_2414    146  6.17e-12 
    EAKRRLTEAN     L      R      L      V      V      S      I      A      K      R      Y       VGRGMLFLDL 
  gi|229916320|EAT1b_0589|    136  6.17e-12 
    EAKKRLAEAN     L      R      L      V      V      S      I      A      K      R      Y       VGRGMLFLDL 
  gi|157693020|BPUM_2253|Y    145  6.17e-12 
    ESKRRLAEAN     L      R      L      V      V      S      I      A      K      R      Y       VGRGMLFLDL 
  gi|154686781|RBAM_023510    145  6.17e-12 
    ESKRRLAEAN     L      R      L      V      V      S      I      A      K      R      Y       VGRGMLFLDL 
  gi|288555754|BpOF4_13730    144  6.17e-12 
    EAKRRLAEAN     L      R      L      V      V      S      I      A      K      R      Y       VGRGMLFLDL 
  gi|169829158|Bsph_3702|Y    147  6.17e-12 
    EARKRLAEAN     L      R      L      V      V      S      I      A      K      R      Y       VGRGMLFLDL 
  gi|301055785|BACI_c42620    147  6.17e-12 
    EAKRRLAEAN     L      R      L      V      V      S      I      A      K      R      Y       VGRGMLFLDL 
  gi|163119552|BL03682|YP_    145  6.17e-12 
    ESKRRLAEAN     L      R      L      V      V      S      I      A      K      R      Y       VGRGMLFLDL 
  gi|172056869|Exig_0832|Y    133  6.17e-12 
    EAKKRLAEAN     L      R      L      V      V      S      I      A      K      R      Y       VGRGMLFLDL 
  gi|52786447|BLi02712|YP_    145  6.17e-12 
    ESKRRLAEAN     L      R      L      V      V      S      I      A      K      R      Y       VGRGMLFLDL 
  gi|261405623|GYMC10_1774    149  6.17e-12 
    EAKRRLAEAN     L      R      L      V      V      S      I      A      K      R      Y       VGRGMLFLDL 
  gi|23099399|OB1944|NP_69    152  6.17e-12 
    EAKRRLAEAN     L      R      L      V      V      S      I      A      K      R      Y       VGRGMLFLDL 
  gi|294501280|BMQ_4542|YP    149  6.17e-12 
    EAKRRLAEAN     L      R      L      V      V      S      I      A      K      R      Y       VGRGMLFLDL 
  gi|138896056|GTNG_2419|Y    147  6.17e-12 
    EAKRRLTEAN     L      R      L      V      V      S      I      A      K      R      Y       VGRGMLFLDL 
  gi|295695448|Btus_0783|Y    151  6.17e-12 
    EAKAALIKAN     L      R      L      V      V      S      I      A      K      R      Y       VGRGMLFLDL 
  gi|15613939|BH1376|NP_24    144  6.17e-12 
    EAKRRLAEAN     L      R      L      V      V      S      I      A      K      R      Y       VGRGMLFLDL 
  gi|296504786|BMB171_C395    147  6.17e-12 
    EAKRRLAEAN     L      R      L      V      V      S      I      A      K      R      Y       VGRGMLFLDL 
  gi|23099294|OB1839|NP_69    40  7.52e-12 
    SARDRLVEKN     I      R      L      V      W      S      V      V      Q      R      F       INRGYDPDDL 
  gi|261405990|GYMC10_2143    41  7.52e-12 
    VARDTLVNCN     I      R      L      V      W      S      V      V      Q      R      F       MNRGYEPDDL 
  gi|261407775|GYMC10_3981    88  1.22e-11 
    EAKRRLAEAN     L      R      L      V      V      S      I      A      R      R      Y       AGRGMQFLDL 
  gi|169827019|Bsph_1443|Y    31  1.22e-11 
    RARDTLIERN     L      R      L      V      V      Y      I      A      R      R      F       DNTGTPIEDL 
  gi|288555973|BpOF4_14835    44  2.86e-11 
    QARDLIVNRN     T      R      L      V      W      S      V      V      Q      R      F       MNRGYEADDL 
  gi|15614101|BH1538|NP_24    42  2.86e-11 
    EARDQIVSRN     T      R      L      V      W      S      V      V      Q      R      F       LNRGYEADDL 
  gi|56963560|ABC1795|YP_1    42  2.86e-11 
    EARDSIVNHN     T      R      L      V      W      S      V      V      Q      R      F       LNRGYEADDL 
  gi|212639646|Aflv_1820|Y    47  3.66e-11 
    SAREKLITGN     L      R      L      V      L      S      V      I      Q      R      F       NNRGEYVDDL 
  gi|42782996|BCE_3948|NP_    77  3.66e-11 
    SAREKLVNGN     L      R      L      V      L      S      V      I      Q      R      F       NNRGEYVDDL 
  gi|218233406|BCB4264_A40    47  3.66e-11 
    SAREKLVNGN     L      R      L      V      L      S      V      I      Q      R      F       NNRGEYVDDL 
  gi|227813187|BAMEG_0585|    47  3.66e-11 
    SAREKLVNGN     L      R      L      V      L      S      V      I      Q      R      F       NNRGEYVDDL 
  gi|163941643|BcerKBAB4_3    80  3.66e-11 
    SAREKLVNGN     L      R      L      V      L      S      V      I      Q      R      F       NNRGEYVDDL 
  gi|152976264|Bcer98_2552    47  3.66e-11 
    SAREKLVNGN     L      R      L      V      L      S      V      I      Q      R      F       NNRGEFVDDL 
  gi|56419663|GK1128|YP_14    47  3.66e-11 
    EAREKLVNGN     L      R      L      V      L      S      V      I      Q      R      F       NNRGEFVDDL 
  gi|49478441|BT9727_3645|    77  3.66e-11 
    SAREKLVNGN     L      R      L      V      L      S      V      I      Q      R      F       NNRGEYVDDL 
  gi|30263905|BA4042|NP_84    47  3.66e-11 
    SAREKLVNGN     L      R      L      V      L      S      V      I      Q      R      F       NNRGEYVDDL 
  gi|217961324|BCAH187_A39    47  3.66e-11 
    SAREKLVNGN     L      R      L      V      L      S      V      I      Q      R      F       NNRGEYVDDL 
  gi|229602177|BAA_4068|YP    47  3.66e-11 
    SAREKLVNGN     L      R      L      V      L      S      V      I      Q      R      F       NNRGEYVDDL 
  gi|30021992|BC3903|NP_83    47  3.66e-11 
    SAREKLVNGN     L      R      L      V      L      S      V      I      Q      R      F       NNRGEYVDDL 
  gi|218905033|BCAH820_391    47  3.66e-11 
    SAREKLVNGN     L      R      L      V      L      S      V      I      Q      R      F       NNRGEYVDDL 
  gi|225865885|BCA_4007|YP    47  3.66e-11 
    SAREKLVNGN     L      R      L      V      L      S      V      I      Q      R      F       NNRGEYVDDL 
  gi|47778237|GBAA4042|YP_    47  3.66e-11 
    SAREKLVNGN     L      R      L      V      L      S      V      I      Q      R      F       NNRGEYVDDL 
  gi|222097349|BCQ_3689|YP    77  3.66e-11 
    SAREKLVNGN     L      R      L      V      L      S      V      I      Q      R      F       NNRGEYVDDL 
  gi|49186753|BAS3754|YP_0    77  3.66e-11 
    SAREKLVNGN     L      R      L      V      L      S      V      I      Q      R      F       NNRGEYVDDL 
  gi|16078597|BSU15330|NP_    48  3.66e-11 
    SAREKLVNGN     L      R      L      V      L      S      V      I      Q      R      F       NNRGEYVDDL 
  gi|218899057|BCG9842_B12    47  3.66e-11 
    SAREKLVNGN     L      R      L      V      L      S      V      I      Q      R      F       NNRGEYVDDL 
  gi|52141585|BCZK3662|YP_    77  3.66e-11 
    SAREKLVNGN     L      R      L      V      L      S      V      I      Q      R      F       NNRGEYVDDL 
  gi|118479123|BALH_3533|Y    77  3.66e-11 
    SAREKLVNGN     L      R      L      V      L      S      V      I      Q      R      F       NNRGEYVDDL 
  gi|297530705|GC56T3_2445    47  3.66e-11 
    EAREKLVNGN     L      R      L      V      L      S      V      I      Q      R      F       NNRGEFVDDL 
  gi|301055394|BACI_c38590    77  3.66e-11 
    SAREKLVNGN     L      R      L      V      L      S      V      I      Q      R      F       NNRGEYVDDL 
  gi|157692208|BPUM_1427|Y    48  3.66e-11 
    TAREKLVNGN     L      R      L      V      L      S      V      I      Q      R      F       NNRGEYVDDL 
  gi|239826533|GWCH70_1031    47  3.66e-11 
    EAREKLVNGN     L      R      L      V      L      S      V      I      Q      R      F       NNRGEFVDDL 
  gi|288553150|BpOF4_00620    47  3.66e-11 
    SAREVLVNGN     L      R      L      V      L      S      V      I      Q      R      F       NNRGEYVDDL 
  gi|295706362|BMD_4257|YP    47  3.66e-11 
    TAREKLVSGN     L      R      L      V      L      S      V      I      Q      R      F       NNRGEFVDDL 
  gi|56964115|ABC2350|YP_1    47  3.66e-11 
    PARETLVNGN     L      R      L      V      L      S      V      I      Q      R      F       NNRGENVDDL 
  gi|138894663|GTNG_0993|Y    52  3.66e-11 
    EAREKLVNGN     L      R      L      V      L      S      V      I      Q      R      F       NNRGEFVDDL 
  gi|23098931|OB1476|NP_69    48  3.66e-11 
    TAREELVNGN     L      R      L      V      L      S      V      I      Q      R      F       NNRGEYVDDL 
  gi|52785510|BLi01751|YP_    48  3.66e-11 
    TAREKLVNGN     L      R      L      V      L      S      V      I      Q      R      F       NNRGEYVDDL 
  gi|295696452|Btus_1846|Y    47  3.66e-11 
    SARERLIHGN     L      R      L      V      L      S      V      I      Q      R      F       NNRGENVDDL 
  gi|154685949|RBAM_015160    48  3.66e-11 
    SAREKLVNGN     L      R      L      V      L      S      V      I      Q      R      F       NNRGEYVDDL 
  gi|296504397|BMB171_C356    47  3.66e-11 
    SAREKLVNGN     L      R      L      V      L      S      V      I      Q      R      F       NNRGEYVDDL 
  gi|261405674|GYMC10_1825    48  3.66e-11 
    SAREKLVNGN     L      R      L      V      L      S      V      I      Q      R      F       NNRGEFVDDL 
  gi|294501013|BMQ_4269|YP    47  3.66e-11 
    TAREKLVSGN     L      R      L      V      L      S      V      I      Q      R      F       NNRGEFVDDL 
  gi|15615117|BH2554|NP_24    47  3.66e-11 
    SARETLVNGN     L      R      L      V      L      S      V      I      Q      R      F       NNRGEYVDDL 
  gi|261419325|GYMC61_1901    47  3.66e-11 
    EAREKLVNGN     L      R      L      V      L      S      V      I      Q      R      F       NNRGEFVDDL 
  gi|52080136|BL02256|YP_0    48  3.66e-11 
    TAREKLVNGN     L      R      L      V      L      S      V      I      Q      R      F       NNRGEYVDDL 
  gi|255767354|BSU15320|NP    67  4.73e-11 
    AARAILIERN     L      R      L      V      V      Y      I      A      R      K      F       ENTGINIEDL 
  gi|227813186|BAMEG_0584|    67  4.73e-11 
    AARSLLIERN     L      R      L      V      V      Y      I      A      R      K      F       ENTGINIEDL 
  gi|152976265|Bcer98_2553    67  4.73e-11 
    AARSLLIERN     L      R      L      V      V      Y      I      A      R      K      F       ENTGINIEDL 
  gi|218899058|BCG9842_B12    67  4.73e-11 
    AARSLLIERN     L      R      L      V      V      Y      I      A      R      K      F       ENTGINIEDL 
  gi|218905035|BCAH820_391    67  4.73e-11 
    AARSLLIERN     L      R      L      V      V      Y      I      A      R      K      F       ENTGINIEDL 
  gi|30263906|BA4043|NP_84    67  4.73e-11 
    AARSLLIERN     L      R      L      V      V      Y      I      A      R      K      F       ENTGINIEDL 
  gi|225865886|BCA_4008|YP    67  4.73e-11 
    AARSLLIERN     L      R      L      V      V      Y      I      A      R      K      F       ENTGINIEDL 
  gi|47529336|GBAA4043|YP_    67  4.73e-11 
    AARSLLIERN     L      R      L      V      V      Y      I      A      R      K      F       ENTGINIEDL 
  gi|212639647|Aflv_1821|Y    67  4.73e-11 
    TARSLLIERN     L      R      L      V      V      Y      I      A      R      K      F       ENTGINIEDL 
  gi|52141583|BCZK3663|YP_    67  4.73e-11 
    AARSLLIERN     L      R      L      V      V      Y      I      A      R      K      F       ENTGINIEDL 
  gi|42782997|BCE_3949|NP_    67  4.73e-11 
    AARSLLIERN     L      R      L      V      V      Y      I      A      R      K      F       ENTGINIEDL 
  gi|217961326|BCAH187_A39    67  4.73e-11 
    AARSLLIERN     L      R      L      V      V      Y      I      A      R      K      F       ENTGINIEDL 
  gi|222097350|BCQ_3690|YP    67  4.73e-11 
    AARSLLIERN     L      R      L      V      V      Y      I      A      R      K      F       ENTGINIEDL 
  gi|229601447|BAA_4069|YP    67  4.73e-11 
    AARSLLIERN     L      R      L      V      V      Y      I      A      R      K      F       ENTGINIEDL 
  gi|30021993|BC3904|NP_83    67  4.73e-11 
    AARSLLIERN     L      R      L      V      V      Y      I      A      R      K      F       ENTGINIEDL 
  gi|162382776|BALH_3534|Y    67  4.73e-11 
    AARSLLIERN     L      R      L      V      V      Y      I      A      R      K      F       ENTGINIEDL 
  gi|218234749|BCB4264_A40    67  4.73e-11 
    AARSLLIERN     L      R      L      V      V      Y      I      A      R      K      F       ENTGINIEDL 
  gi|49186754|BAS3755|YP_0    67  4.73e-11 
    AARSLLIERN     L      R      L      V      V      Y      I      A      R      K      F       ENTGINIEDL 
  gi|163941644|BcerKBAB4_3    67  4.73e-11 
    AARSMLIERN     L      R      L      V      V      Y      I      A      R      K      F       ENTGINIEDL 
  gi|49478442|BT9727_3646|    67  4.73e-11 
    AARSLLIERN     L      R      L      V      V      Y      I      A      R      K      F       ENTGINIEDL 
  gi|56419662|GK1127|YP_14    67  4.73e-11 
    TARSLLIERN     L      R      L      V      V      Y      I      A      R      K      F       ENTGIHIEDL 
  gi|294501014|BMQ_4270|YP    67  4.73e-11 
    AARSLLIERN     L      R      L      V      V      Y      I      A      R      K      F       ENTGINIEDL 
  gi|157692207|BPUM_1426|Y    67  4.73e-11 
    TARAILIERN     L      R      L      V      V      Y      I      A      R      K      F       ENTGINIEDL 
  gi|295696453|Btus_1847|Y    70  4.73e-11 
    SVRSTLIERN     L      R      L      V      V      Y      I      A      R      K      F       ENTGIYIEDL 
  gi|261405673|GYMC10_1824    68  4.73e-11 
    ATRAMLIERN     L      R      L      V      V      Y      I      A      R      K      F       ENTGINIEDL 
  gi|15615119|BH2556|NP_24    65  4.73e-11 
    AVRSMLIERN     L      R      L      V      V      Y      I      A      R      K      F       ENTGINIEDL 
  gi|296504398|BMB171_C356    67  4.73e-11 
    AARSLLIERN     L      R      L      V      V      Y      I      A      R      K      F       ENTGINIEDL 
  gi|52785509|BLi01750|YP_    67  4.73e-11 
    AARAILIERN     L      R      L      V      V      Y      I      A      R      K      F       ENTGINIEDL 
  gi|154685948|RBAM_015150    67  4.73e-11 
    AARAILIERN     L      R      L      V      V      Y      I      A      R      K      F       ENTGINIEDL 
  gi|52080135|BL02255|YP_0    67  4.73e-11 
    AARAILIERN     L      R      L      V      V      Y      I      A      R      K      F       ENTGINIEDL 
  gi|288553151|BpOF4_00625    65  4.73e-11 
    AVRSMLIERN     L      R      L      V      V      Y      I      A      R      K      F       ENTGINIEDL 
  gi|138894662|GTNG_0992|Y    67  4.73e-11 
    TARSLLIERN     L      R      L      V      V      Y      I      A      R      K      F       ENTGIHIEDL 
  gi|23098930|OB1475|NP_69    67  4.73e-11 
    AARAMLIERN     L      R      L      V      V      Y      I      A      R      K      F       ENTGINIEDL 
  gi|239826532|GWCH70_1030    67  4.73e-11 
    TARSLLIERN     L      R      L      V      V      Y      I      A      R      K      F       ENTGINIEDL 
  gi|56964116|ABC2351|YP_1    65  4.73e-11 
    AVRSVLIERN     L      R      L      V      V      Y      I      A      R      K      F       ENTGINIEDL 
  gi|297530706|GC56T3_2446    67  4.73e-11 
    TARSLLIERN     L      R      L      V      V      Y      I      A      R      K      F       ENTGIHIEDL 
  gi|295706363|BMD_4258|YP    67  4.73e-11 
    AARSLLIERN     L      R      L      V      V      Y      I      A      R      K      F       ENTGINIEDL 
  gi|261419324|GYMC61_1900    67  4.73e-11 
    TARSLLIERN     L      R      L      V      V      Y      I      A      R      K      F       ENTGIHIEDL 
  gi|301055395|BACI_c38600    66  4.73e-11 
    AARSLLIERN     L      R      L      V      V      Y      I      A      R      K      F       ENTGINIEDL 
  gi|229603939|BAA_4584|YP    58  5.37e-11 
    QARNLLIEHN     L      R      L      V      A      H      I      V      K      K      F       ENTGEDAEDL 
  gi|49481469|BT9727_4074|    58  5.37e-11 
    QARNLLIEHN     L      R      L      V      A      H      I      V      K      K      F       ENTGEDAEDL 
  gi|49187232|BAS4236|YP_0    58  5.37e-11
[truncated: 606,789 more chars]
