# Supplementary figures and images for: Whole-genome phylogenies of the family Bacillaceae and expansion of the sigma factor gene family in the Bacillus cereus species-group
Source: BMC Genomics. 2011 Aug 24;12:430. doi: 10.1186/1471-2164-12-430 (PMC3171730; doi:10.1186/1471-2164-12-430)

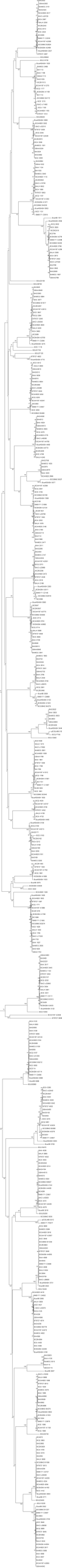

0.2

Supplement: Additional file 4 — Results of phylogenetic analysis of the sigma factors identified in Additional file 2. Phylogenetic analysis utilized the neighbor-joining algorithm of MEGA (see text). [file 1471-2164-12-430-S4.PDF]
